# Silence as relational adaptation: Strategic communicative practices of Chinese partners in Sino-Western intercultural marriages

**DOI:** 10.3389/fpsyg.2026.1957138

**Published:** 2026-10-16

**Authors:** Hongya Fan, Weiqian Xiang

**Affiliations:** School of Foreign Languages, Shanxi University, Taiyuan, Shanxi, China

**Keywords:** Chinese partners, intercultural communication, intercultural marriage, proceduralized grounded theory, relational adaptation, silence

## Abstract

**Introduction:**

Silence is a pervasive yet understudied communicative practice in intercultural intimate relationships. Existing research has primarily examined silence in public and institutional contexts, while limited attention has been paid to how silence functions within intercultural marriages as a relational and adaptive practice. This study explores how Chinese partners in Sino-Western intercultural marriages experience, enact, and negotiate silence during everyday interactions.

**Methods:**

Employing a proceduralized grounded theory approach, this study integrates qualitative data from semi-structured interviews, video observations, and textual materials. Through iterative open, axial, and selective coding procedures, a processual model was developed to explain the emergence, transformation, and consequences of Chinese partners’ silence in intercultural marital contexts.

**Results:**

The findings reveal that Chinese partners’ silence operates as a dynamic communicative adaptation mechanism rather than a passive withdrawal from interaction. Silence evolves through a processual trajectory of antecedents, manifestations, adjustments, and consequences. Triggered by cross-cultural divergences, situational norm conflicts, and L2 pragmatic constraints, silence manifests in distinct intimacy-maintaining, boundary-preserving, and mutuality-enhancing orientations. Furthermore, two adjustment pathways—proactive individual adjustment and collaborative dyadic adjustment—enable partners to transform silence from a potential source of misunderstanding into a constructive relational resource. These adaptive processes contribute to the regulation of marital interactions, renegotiation of relational power dynamics, and enhancement of intercultural understanding.

**Discussion:**

This study reconceptualizes silence as an agentive interpersonal strategy through which Chinese partners actively manage cultural differences, emotional expression, and relational boundaries in intercultural marriages. By highlighting the adaptive and relational functions of silence, this study extends psychological understandings of intercultural communication and intimate relationship adjustment, demonstrating how nonverbal practices contribute to the maintenance and development of intercultural marital relationships.

## 1 Introduction

China’s growing economic integration with the world has contributed to a steady increase in cross-border romantic relationships and intercultural marriages (Nehring and Wang, 2016; Barabantseva and Grillot, 2019; Sier and Barabantseva, 2026). In 2024, 61,900 marriages involving foreign nationals or residents from Hong Kong, Macao, and Taiwan were registered in China (National Bureau of Statistics of China, 2025). Unlike monocultural couples, intercultural couples are more prone to friction and conflict due to the inherent challenges of integrating different cultural backgrounds (Janicka, 2025). These differences are particularly visible in language use (Li, 2026), through which partners negotiate personal identities, enact cultural hybridity, adjust relational boundaries, and cultivate a shared couple culture (Piller, 2002; Pietikäinen, 2017). Silence, as a non-verbal behavior, is an important but understudied part of this process. In intimate relationships, silence can be used to resolve marital conflicts and maintain the image of oneself and their spouses (Cheng and Tardy, 2009). Yet there are significant differences in the interpretation and perception of silence across cultures (Ling, 2003; Nakane, 2007; Sun, 2016; Hayati and Sinha, 2024).

In many Chinese and other East Asian cultures, silence has traditionally been endowed with pro-social values as a pragmatic tool to express respect, preserve face, and maintain interpersonal harmony; on the contrary, in Western cultures (especially in English-speaking countries), silence is more likely to be interpreted as embarrassment, disapproval, unfriendliness, or incompetence (Lemak, 2012; Quan, 2015; Hayati and Sinha, 2024; Kelly et al., 2026). Such mismatches may cause cognitive bias in Sino-Western intercultural marriages, lead to pragmatic misunderstandings, intensify relational conflicts, and affect the sustainable development of marital relationships. Although existing research has explored the connotation characteristics and cultural specificity of silence from the perspective of intercultural communication, the analytical frameworks mainly focus on public or institutional fields such as education and the workplace (Hwang et al., 2002; Jackson, 2002; Verouden et al., 2018), while the private field of marriage and intimacy has received little attention. In addition, studies on intercultural intimate relationships have widely explored value differences, conflict management, communication patterns, and relationship satisfaction (Hiew et al., 2015a, b; Halford et al., 2018a, b; Sun and Yuan, 2021; Li, 2026), with a primary focus on explicit verbal interaction but limited attention to silence as a non-verbal behavior. Despite a small body of literature on silence in intimate relationships (Cheng and Tardy, 2009; Pietikäinen, 2018; Weinstein et al., 2024), few studies have centered on Chinese partners, leaving the dynamic, processual mechanisms underlying how their silent behaviors emerge and evolve underexplored.

Building on these research gaps, this study adopts proceduralized grounded theory (Strauss and Corbin, 1998) to investigate Chinese partners’ silence in Sino-Western intercultural marriages. By integrating three types of qualitative data—semi-structured interviews, online video observations, and relevant textual materials—this study develops a processual model explaining how silence emerges, functions, and transforms within intercultural marital interactions. Rather than conceptualizing silence as a stable cultural disposition or a simple absence of speech, this study views it as a dynamic communicative practice whose meanings and functions are continuously negotiated through relational encounters, cultural differences, and situational demands.

This study extends existing research on silence beyond predominantly public and institutional settings by exploring its role in the private domain of intercultural intimate relationships. It advances a process-oriented understanding of silence by revealing how cultural expectations, relational dynamics, and linguistic constraints jointly shape the emergence and transformation of silent practices over time. By demonstrating how Chinese partners actively employ silence as an agentive communicative resource to regulate conflicts, negotiate interpersonal boundaries, and construct shared meanings, this study contributes to research on intercultural communication, relationship psychology, and interpersonal adaptation. More broadly, it illuminates the psychological and relational processes through which individuals navigate cultural divergences and develop adaptive interaction patterns in intercultural marriages.

## 2 Literature review

### 2.1 Intercultural silence: theoretical dimensions and institutional settings

Regarding the intercultural dimension of silence, the academic community has accumulated a large number of literatures, among which comparative studies between Eastern and Western cultures are the most prominent (Murayama, 1995; Gu, 2004; Nakane, 2007; Jones, 2011; Quan, 2015). These studies mainly examine the value orientations, interpretive rationales, and interactional consequences of silence. Based on Hall’s (1976) high- and low-context framework and Hofstede’s (1980) cultural dimensions theory, the research direction establishes different cultural differences. In general, Western individualistic and low-context cultures attach importance to explicit verbal articulation, and often interpret silence as rejection, indifference, anger, or hostility; while collectivistic and high-context cultures in the East or Asia rely heavily on implicit non-verbal cues, usually giving silence positive meanings such as wisdom and respect, and utilizing silence to avoid conflicts, prevent embarrassment, maintain interpersonal harmony, and convey subtle emotions (Turner, 2012; Guo, 2020; Dodu-Savca, 2022; Gutiérrez and Paniagua, 2024). From the perspective of politeness theory (Brown and Levinson, 1987), silence is a strategy to maintain face and show politeness, which is used to alleviate face-threatening acts (FTA); however, when the normative context requires active verbal participation in communication, silence is easily misunderstood, which ultimately leads to intercultural pragmatic failures (Nakane, 2006). These different orientations can be traced back to foundational philosophical and rhetorical traditions. Western thought influenced by Aristotelian and Ciceronian traditions promoted the Greek and Roman persuasive rhetoric, regarded language as the main tool for discovering truth, and advocated rational eloquence. On the contrary, classical Chinese Confucianism and Taoism traditionally valued harmony and tranquility, downplayed overt disputation, and regarded silence as an authentic and appropriate way of interaction (Gu, 2004; Li, 2007). Contemporary academic research transcends this binary opposition between words and silence, and further emphasizes the positivity contained in Eastern silence. For instance, Yuan (2021) pointed out through the investigation of early historical and philosophical dialogue that silence in early Chinese discourse is rarely a natural or spontaneous phenomenon; rather, it is a highly strategic and context-dependent mode of communication utilized for political persuasion and performative protests, proving just as argumentative and conventional as oral expression.

These theoretical insights and cultural differences have been empirically clarified and verified across two main institutional settings of education and the workplace. Educational research has widely focused on the causes and functions of classroom silence among East Asian L2 learners, identifying cultural-psychological mechanisms (e.g., authority orientation, face concerns and kiasuism) as well as situational and individual variables (e.g., pedagogical style, language proficiency, and communication anxiety) (Cheng, 2000; Hwang et al., 2002; Jackson, 2002). Because Western classrooms frequently misunderstand silence as insufficient ability or low participation, interactive tension and cultural stereotypes are often reinforced (Zhu and Bresnahan, 2018). Ali (2025) challenged these assumptions, re-examined Chinese students’ silence in Western universities, and pointed out that silence can serve as a dynamic communicative behavior and a tool for critical reflection. Ali deeply analyzed the multi-dimensional cultural, linguistic, pedagogical, and social driving factors behind silence, and put forward empirical-based pedagogical strategies for culturally responsive and non-exclusive teaching in multicultural classrooms. In organizational studies, research on employee silence confirms the intercultural applicability of silence motives and explores their configurations with power distance and institutional collectivism (Knoll et al., 2021). In addition, research on intercultural professional collaborations showed that differences in the tolerance and interpretation of Chinese silence in diverse cultures will significantly affect the effectiveness of trust-building and collaboration (Spencer-Oatey and Xing, 2005; Verouden et al., 2018). Taken together, previous studies showed that Chinese silence is not a mere symptom of communicative breakdown, but rather a manageable, strategic, albeit highly misinterpretable, interactional resource. Extending this line of inquiry, Fan and Xiang (2026) further demonstrated that Chinese silence in intercultural communication is not a fixed cultural disposition or passive absence, but a dynamic process shaped by cultural predispositions, contextual negotiations, strategic enactment, and subsequent habitualization. The findings revealed the agentive and adaptive nature of Chinese silence; however, how such silent practices are experienced, negotiated, and transformed within high-intimacy relational contexts, particularly intercultural marriages, remains underexplored.

### 2.2 Intercultural marital relationships: explicit conflicts, communication patterns, and conflict management

The research on Sino-Western intercultural marriage mainly focuses on the conflicts caused by cultural differences, and examines the static differences in values and dynamic interactions. There are significant differences in the dynamics of marriage on the individualism–collectivism spectrum: Western relationships prioritize individual autonomy, personal goals, and explicit emotional intimacy; in contrast, Chinese marriages are traditionally regarded as a union embedded within extensive kinship networks, where partners often dilute individual or couple-level desires to fulfill their collective familial responsibilities and maintain the integration of the extended family (Williamson et al., 2012; Halford et al., 2018c; Lam et al., 2016). Utilizing a mixed-methods design, Young (2012) elaborated on these cultural differences and found that the conflict in the Sino-American transnational marriage is mainly concentrated in the dimensions of linguistic communication, acculturation, religious beliefs, in-laws, political views, consumption habits, and children’s upbringing, and noted that the degree of Chinese partners’ adaptation to Western culture is positively related to their marital satisfaction. Hiew et al. (2015c) developed the Chinese-Western Intercultural Couple Standards Scale (CWICSS), which provided psychometric support for these different cultural norms. The study revealed that although the two cultural groups also attach importance to the “couple bond,” Chinese partners show a significantly stronger sense of identity with the “family responsibility” standards (e.g., extended family and relationship harmony).

In the field of dynamic interaction, communication patterns and conflict management are the main research focuses. Christensen et al. (2006) confirmed the cross-cultural consistency of the “demand/withdraw” pattern, pointing out its robust negative correlation with relationship satisfaction. Zeng’s (2014) further cross-cultural comparative research revealed that this pattern varies across socio-cultural backgrounds and the urban–rural divide, with socio-economic factors such as education and income having a notable impact on power relationships and conflict progression in interaction. At the level of micro-interaction, emerging studies have explored silence in marital conflicts. For instance, based on the self-construal theory, Cheng and Tardy (2009) observed that Taiwanese (characterized by higher interdependent self-construal) tend to maintain the face of their spouses through silence, while Americans (characterized by higher independent self-construal) are more likely to employ silence to safeguard their own image. Adopting conversation analysis, Pietikäinen (2018) revealed the polyfunctional nature of silence manifested as withholding a response during conflicts between intercultural couples using English as a lingua franca (ELF), showing that such silence is not a passive lack of speech, but an active strategy for interactive meaning-taking.

In addition to focusing on the micro-interaction at the level of linguistic contact, a broader line of inquiry explored the overall picture of emotional and pragmatic landscapes in intercultural romantic relationships. Dewaele and Salomidou (2017) employed a large-scale mixed-methods approach to argue that while language and cultural differences initially bring linguistic and psychological challenges to emotional communication in the foreign language (LX), these obstacles are rarely permanent; rather, they fade within months through affective socialization, and the partner’s language gradually evolves into the “language of the heart.” Dewaele (2018) further refined these interactional dynamics from the perspective of pragmatics, systematically summarized the difficulties in emotional communication into distinct pragmalinguistic and sociopragmatic dimensions, and concluded that despite pragmatic problems during relational inception, long-term relational engagement promotes emotional acculturation and alleviates communicative friction over time.

In summary, although the extant literature provided valuable insights into the understanding of intercultural silence and marital friction, significant empirical and theoretical gaps remain. First, the research on intercultural silence was mostly limited to the public or institutional field, leaving its factors, manifestations, and consequences in the private, high-stakes realm of marital intimacy largely undertheorized. Second, the research on intercultural marital communication focused on overt conflicts and explicit verbal exchange, ignoring silence as an implicit but universal communicative practice. Finally, existing studies on silence in intimate relationships (e.g., Cheng and Tardy, 2009; Pietikäinen, 2018) rarely privileged the subjective experience of Chinese partners, and failed to capture the underlying processual mechanisms through which their non-verbal behaviors dynamically unfold. To address these limitations, this study adopts proceduralized grounded theory, and examines the implicit interactional logic of Chinese partners’ silence in intercultural marriages through qualitative data obtained from semi-structured interviews, online video observations, and textual materials. The following two research questions are proposed: (1) In Sino-Western intercultural marriages, how do Chinese partners experience and enact silence as an implicit communicative practice? (2) In Sino-Western intercultural marriages, how does Chinese partners’ silence evolve dynamically?

## 3 Methodology

### 3.1 Method

In order to capture the subtle and microscopic interactional dynamics of silence in Sino-Western intercultural marriages, this study adopts the proceduralized grounded theory proposed by Strauss and Corbin (1998). Chinese partners’ silence in intercultural marriages is unlikely to be a fixed communicative behavior with a single cultural meaning. Rather, it may emerge from the interaction between culturally shaped expectations, relational dynamics, situational pressures, and partners’ ongoing attempts to manage conflict and preserve the relationship. The proceduralized grounded theory is particularly applicable here to understand this process and trace how silent behaviors emerge, are interpreted, and subsequently shape further interaction. The method is characterized by rigorous bottom-up inductive reasoning, allowing categories and generative mechanisms to emerge directly from raw data. In the end, its systematic focus on condition-action-consequence sequences facilitates constructing a dynamic, processual model through the three consecutive stages of open coding, axial coding, and selective coding.

### 3.2 Data collection

This study collected qualitative data from three different sources. First, semi-structured interviews were conducted with 25 Chinese participants in Sino-Western intercultural marriages. The study combined the principles of purposive sampling, snowball sampling, and maximum variance to ensure that the sample covers diverse demographic characteristics such as gender, marriage duration, spouse’s nationality, and geographical location (see Table 1). Each interview lasted 40 to 50 minutes, focusing on cross-cultural conflict experiences, the contexts and manifestations of silence, the internal psychological process of the participants during silence, and the subsequent coping strategies. With participants’ informed consent, interviews were recorded and transcribed verbatim. To enhance the transparency and verifiability of the qualitative analysis, the interview transcripts were subsequently subjected to systematic anonymization and de-identification. The anonymized and de-identified transcripts were translated into English, resulting in approximately 130,000-word English-language interview transcripts. Second, to complement the interview accounts, we collected 20 public interaction videos posted by intercultural couples on mainstream social media platforms, including Xiaohongshu (also known as rednote) and TikTok. Observational notes specifically documented instances of silence along with accompanying non-verbal cues (e.g., body movements, facial expressions, and partner feedback). We explicitly acknowledged that public social media content may represent curated digital narratives tailored for audience engagement, which may reflect selective self-presentation rather than unscripted daily interactions. Accordingly, these video data were analyzed primarily as performative representations of intercultural communication and were systematically triangulated with our interview findings. Third, supplementary textual materials were collected for comparative analysis. The corpus contained 20 academic publications (journal papers and monographs covering intercultural communication, marital sociology, and silence studies) and 10 journalistic reports or social commentaries on intercultural marriages.

**Table 1:** Demographic information of participants (*N* = 25).

Characteristics		Sample size	Proportion (%)
Gender	Male	6	24
	Female	19	76
Marital duration	Less than 3 years	9	36
	4–10 years	12	48
	More than 10 years	4	16
Spouse nationality	United Kingdom	12	48
	United States	8	32
	Australia	5	20
Geographical location	China	15	60
	English-speaking countries	10	40
Parenthood status	With children	19	76
	Without children	6	24

### 3.3 Data analysis

Following the iterative, constant comparative principle of grounded theory, data analysis was conducted concurrently with data collection. In the open coding stage, we analyzed the interview transcripts, observation notes, and textual materials line by line, conceptualized the behavioral characteristics, psychological intentions, and situational cues related to silence, and summarized them as initial concepts and categories. In the axial coding stage, data were systematically organized using Strauss and Corbin’s (1998) paradigm model. The structural tool interconnects categories through dimensions of conditions, actions/interactions, and consequences, thus realizing the integration of structure and processes. Based on this paradigm, we clarified the boundaries, attributes, and dimensional variations of each subcategory, and further refined them into the main categories. In the selective coding stage, major categories were integrated around a single core category to finalize the processual theoretical model. Throughout the analytical process, research memos were systematically maintained to record analytical insights, theoretical intuitions, and comparisons between categories to safeguard the conceptual depth of the data interpretation. Theoretical sampling was continuously employed in the analytical process until theoretical saturation was reached (Turner and Astin, 2021). To ensure structural validation, about 75% of the raw data were randomly assigned to the initial coding corpus, while the remaining 25% were reserved for independent saturation testing to verify the stability and integrity of the final model.

## 4 Results

### 4.1 Open coding

Open coding is the analytical process of identifying concepts, exploring their properties and dimensions in data, and investigating dimensional variations of generated categories (Strauss and Corbin, 1998). Three sets of raw data were thoroughly analyzed in this research, consisting of 18 semi-structured interview transcripts, 15 video observation records, and 22 relevant textual materials. A total of 92 initial concepts and 20 categories were identified via constant comparison and inductive analysis. Representative open coding results are illustrated in Table 2.

**Table 2:** Open coding examples in qualitative data analysis.

Categories	Concepts	Original statements
F1 Divergences in cultural values	A1 Perceived collectivist-individualist divergence	I7 “In China, we naturally value family harmony and try to accommodate every family member’s feelings. But people from Western countries prioritize individual choice and personal freedom. Because of this clash in mindsets, I usually choose to stay silent to cool down conflicts, rather than destroying our relationship through constant arguing.”
	A2 Different high- and low-context communicative cultures	I12 “Westerners are used to laying everything out on the table and speaking explicitly, whereas we Chinese rely heavily on the immediate context and what goes unsaid. So as the Chinese spouse, I sometimes prefer to express my stance through silence. There is actually a lot of unspoken meaning embedded within that silence.”
	A3 Religious discrepancy	I3 “I’m an atheist, but my American husband is a devout Catholic. He goes to church every week and wants our child to be baptized. I wasn’t really on board, so he thinks I disrespect his faith. Since then, whenever the topic comes up, I just keep my mouth shut to avoid arguments.”
……		
F3 Divergences in familial norms	A8 Differences in the domestic division of labor	T9 Western culture advocates equal division of housework, whereas Eastern culture still retains traditional gender-based role allocation. Such cross-cultural friction frequently triggers domestic disputes, and Chinese partners often resort to silence to evade direct confrontations.
	A9 Cross-cultural lifestyle mismatches	I15 “There’s still a massive gap between our daily habits in a Sino-American household. I’m used to hot meals and warm water, but he cannot survive without salads and iced drinks for every single meal. I keep an early schedule, but he often stays up late hanging out. After countless fruitless attempts to compromise, I just stop nagging him and accept these cultural gaps quietly, sticking to our respective routines.”
	A10 Polarized financial philosophies	I1 “I wanted to save money to buy a house for our kids and support my aging parents, but he believed in living for the moment and spending freely on travel. After trying to talk to him so many times with zero results, I gave up further persuasion and saved money privately, taking silence as a way to prevent clashes over financial ideas.”
……		
F7 Conflict-avoidant silence	A25 Avoiding value-based disputes	I10 “He always says that people should live for themselves first, which completely goes against my family-first belief. But I’ve never argued with him about it. It’s hard to bridge the ideological gap between Chinese and British cultures anyway, so choosing not to poke at it through silence maintains our domestic harmony far better than trying to prove who’s right.”
	A26 Shifting sensitive topics	I5 “Whenever my husband starts talking about Chinese politics, I simply do not bite. By staying silent and shifting the focus away from sensitive topics, I avoid offending him without letting things get awkward.”
	A27 Creating temporal and spatial buffers	I18 “We fell out over parenting styles. I then stayed alone in the study for half an hour. Cultural divergence calls for calm reflection. Silence gives both sides breathing space and prevents hurtful words spoken out of impulse, which makes subsequent communication much smoother.”
……		
F11 Defensive silence	A44 Mitigating emotional distress	I4 “When he openly criticized Chinese-style parenting as being too restrictive in front of his friends, I did not push back. Arguing would only make him dig his heels in his prejudices, and it would end up hurting my mood.”
	A45 Alleviating negative evaluation	I8 “He always frowns at the smoke and grease from Chinese cooking and complains that it is unhygienic. I did not stop the work at hand and did not respond to him. I just turned on the range hood silently. I used silence to resolve his negative opinions on my culinary culture.”
	A46 Safeguarding psychological boundaries	T17 In intercultural households, the silence of the Chinese partner often functions as a psychological boundary-defense mechanism. When facing the Western spouse’s intrusion into their cultural habits or privacy, the Chinese partner uses silence to draw a clear line, preventing their cultural identity from being overly compromised or policed.
……		
F14 Affective-expressive silence	A60 Embracing affection through shyness	O6 When the British husband said “I love you” to his Chinese wife, she instantly blushed, covered her face, and buried her head in shyness. After a few seconds of silence, she bashfully whispered back, “Love you too.”
	A61 Conveying affection through reserved intimacy	I13 “My wife always complains that I’m terrible at expressing my love. But I just cannot wear my heart on my sleeve or be as outwardly passionate as Westerners are. I’m much more reserved. Even though I do not say the words, my love for her is woven into everything I do for her.”
	A62 Signaling dependence through implicit reliance	I2 “Living in Australia, I really struggle with the local accent, so I always feel anxious running errands alone. But I do not like to keep asking him for help verbally. Instead, whenever I have to go somewhere, I’ll just quietly sit next to him until he offers to come along. This kind of restrained reliance fits perfectly with Chinese implicit communication without making him feel burdened.”
……		
F16 Proactive individual adjustment	A70 Clarifying silence intentions	I11 “During the first few years of our marriage, I used to shut down and stay silent a lot. But now, I’ve become much more proactive in explaining the reasons and meanings behind my silence so he is not left guessing.”
	A71 Regulating silence duration	I16 “I know I have a tendency to lapse into habitual silence, but now I control my silence length and avoid prolonged reticence. For example, when we are discussing something important, I’ll tell him, ‘Give me five minutes to think,’ so he does not feel like I’m just avoiding the issue.”
	A72 Supplementing with nonverbal communication	I9 “Sometimes my English fails to capture exactly how I feel, so I’ll use gestures, eye contact, and facial expressions to show him that I’m not angry. Gradually, he gets used to this and patiently waits for me to voice my thoughts.”
……		
F17 Collaborative dyadic adjustment	A78 Forging a shared interpretation of silence	I20 “We’ve made a mutual pact: brief silence simply means I’m processing my thoughts, whereas prolonged silence paired with a dark expression indicates bad moods. Now, he rarely misinterprets my reflective silence as indifference or emotional evasion.”
	A79 Co-constructing relational ground rules for silence	I19 “We agreed to break the ice actively if silence lasts over ten minutes after quarrels, so as to eliminate misunderstandings and prolonged cold wars.”
	A80 Engaging in reciprocal cultural learning	I14 “I find myself going silent much less often now. Based on mutual understanding and respect, my partner and I are actively trying to integrate two cultures and embrace differences. We learn and compromise on dietary habits, celebrate festivals from both countries, and share personal demands and feelings candidly.”
……		
F20 Progression of cultural integration	A91 Deepened cultural inclusiveness	I22 “He failed to understand my silence at first, but over time, he came to realize that it’s just my unique way of communicating. We’ve both learned to put ourselves in each other’s shoes, deeply embracing and respecting each other’s cultural backgrounds and communicative styles.”
	A92 Achievement of cultural negotiation	T5 Silence creates a cooling-off period for cross-cultural negotiation and avoids emotional confrontation. It enables both partners to view cultural discrepancies rationally and seek balance on core demands, eventually reaching mutually acceptable negotiation outcomes that respect both cultures.

I – Semi-structured interviews; O – Video observations; T – Textual artifacts.

### 4.2 Axial coding

Axial coding focuses on exploring the interconnections among categories derived from open coding, aiming to develop and relate major categories to facilitate theoretical development (Strauss and Corbin, 1998). Guided by the conditions-actions/interactions-consequences paradigm, 20 categories obtained in the prior step were abstracted into four major categories. The detailed results are presented in Table 3.

**Table 3:** Categories and major categories of axial coding.

Major categories	Categories	Connotations
Z1 Antecedents of silence	F1 Divergences in cultural values F2 Divergences in marital values F3 Divergences in familial norms F4 Divergences in gendered interaction patterns F5 Constraints in pragmalinguistic competence F6 Deficiencies in sociopragmatic adaptation	Divergences in cultural values and marital values constitute deep-seated cognitive discrepancies, serving as the foundational root of conflict in intercultural interactions. Meanwhile, divergences in familial norms and gendered interaction patterns represent manifest situational norm conflicts, expressing themselves as behavioral distinctions in daily interactions. Furthermore, constraints in pragmalinguistic competence and deficiencies in sociopragmatic adaptation function as rigid, second-language instrumental barriers, presenting objective impediments to communicative practices. These three sets of antecedents exert a compounding effect through three pathways—cognitive underpinnings, interactional rules, and expressive conditions—jointly elevating the probability of silence among Chinese partners.
Z2 Manifestations of silence	F7 Conflict-avoidant silence F8 Forbearing-acquiescent silence F9 Intergenerational-accommodating silence F10 Culturally anchored silence F11 Defensive silence F12 Adversarial silence F13 Tacit silence F14 Affective-expressive silence F15 Digital-buffered silence	Intimacy-maintaining silence, primarily manifested through conflict-avoidant, forbearing-acquiescent, and intergenerational-accommodating forms, aims to sustain relational stability. Conversely, boundary-preserving silence, encompassing culturally anchored, defensive, and adversarial typologies, is fundamentally driven by the preservation of one’s cultural identity and individual boundaries. Mutuality-enhancing silence, specifically observed in tacit, affective-expressive, and digital-buffered forms, represents an interactive endeavor toward reciprocal and bilateral adaptation. The above categories cover the major coping modes ranging from concession and resistance to collaboration.
Z3 Adjustment strategies for silence	F16 Proactive individual adjustment F17 Collaborative dyadic adjustment	Proactive individual adjustment denotes cognitive and behavioral modifications initiated by Chinese partners at the personal level, whereas collaborative dyadic adjustment involves coordination grounded in mutual understanding and communication. While the former emphasizes autonomous rectification, the latter underscores dyadic synergy. Complementing one another, these dual pathways dictate whether silence can successfully be converted into a controllable and constructive communicative resource.
Z4 Consequences of silence	F18 Regulation of marital relationships F19 Reconstruction of power dynamics F20 Progression of cultural integration	The consequences of silence exhibit a clear hierarchical progression. At the micro level, silence shifts the emotional climate and interactional rhythms within the intimate space, thereby steering the regulation of marital relationships. At the meso level, silence catalyzes the tacit reshaping of domestic power dynamics and role positioning. At the macro level, through protracted interactions, silence actively participates in cultural inclusiveness and negotiation, enabling both partners to gradually cultivate a resilient relational matrix following the collision of cultural divergences, ultimately advancing the progression of cultural integration.

### 4.3 Selective coding

Selective coding serves to integrate and refine categories, through which formal theoretical frameworks are developed by focusing all categorized research findings on a core category (Strauss and Corbin, 1998). In this study, “the dynamic adaptation mechanism of Chinese partners’ silence in Sino-Western intercultural marriages” was identified as the core category. By integrating the findings from open and axial coding, this study further clarified the relationships among categories and developed a processual theoretical model, as presented in Figure 1. The model follows a structured storyline characterized by the sequential trajectory of antecedents–manifestations–adjustments–consequences. In the micro-domain of Sino-Western intercultural marriages, when faced with the communication tension caused by a triad of antecedents—value misalignments, situational norm conflicts, and L2 pragmatic barriers—Chinese partners will exercise agency by strategically employing silence across three functional orientations. These encompass intimacy-maintaining forms aimed at preserving the domestic order, boundary-preserving forms centered on establishing personal cultural and emotional boundaries, and mutuality-enhancing forms committed to optimizing intercultural communication channels. As silent practices may generate misunderstanding or relational distance when their meanings remain ambiguous, both partners engage in dynamic adjustment processes to negotiate and reconstruct the meanings and functions of silence. These adjustments occur through two complementary pathways: proactive individual adjustment, involving personal reflection and communicative adaptation, and collaborative dyadic adjustment, involving mutual interpretation, shared rule-making, and relational coordination. Ultimately, these adaptive silent practices reshape marital interactions by influencing relationship regulation, interpersonal power negotiation, and processes of cultural integration within intercultural marriages.

**Figure 1:**
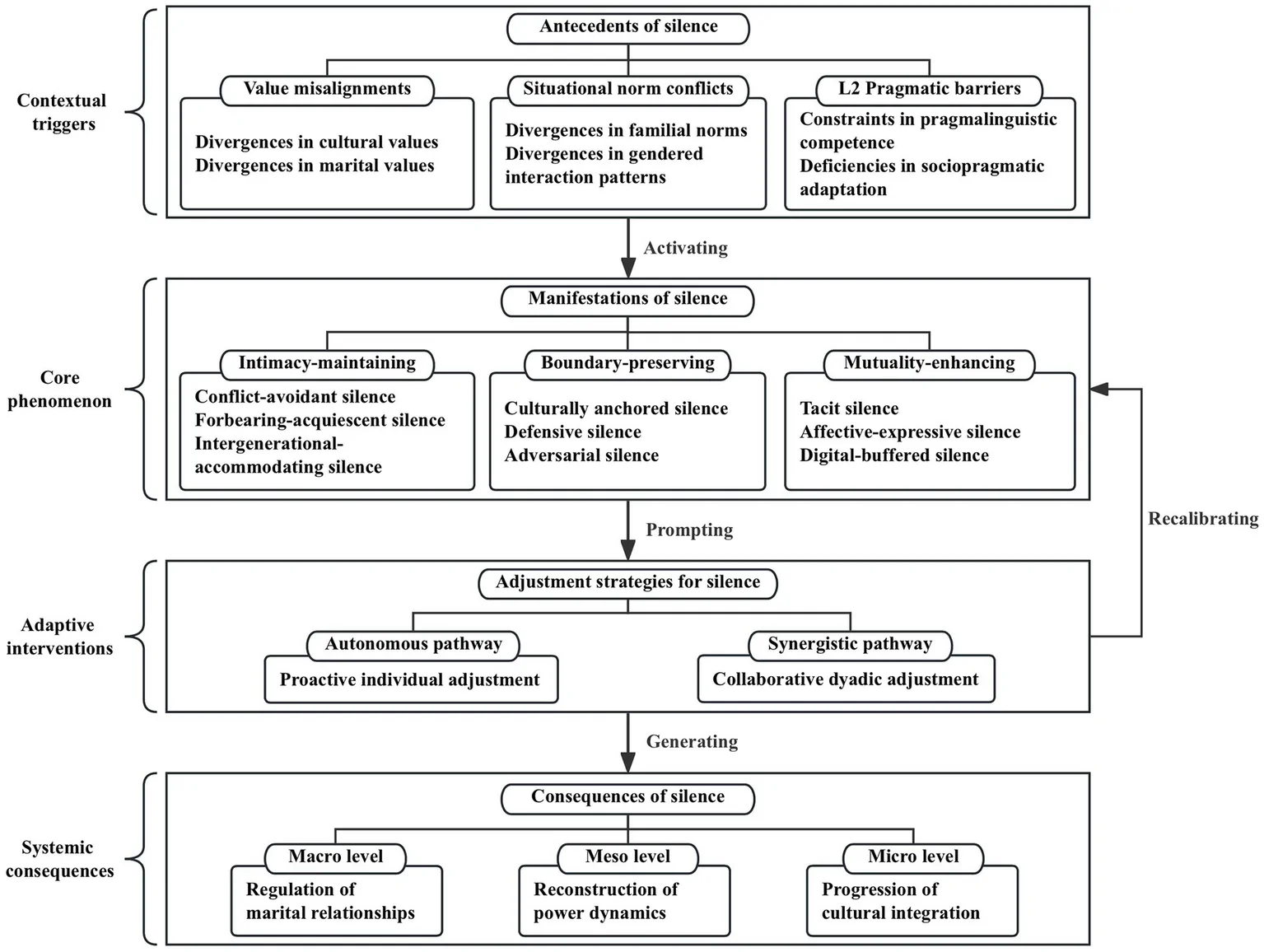
A Model of the dynamic adaptation mechanism of Chinese partners’ silence in Sino-Western intercultural marriages.

### 4.4 Theoretical saturation test

Theoretical saturation refers to the developmental point of category formation, where continuous coding procedures no longer produce new attributes, dimensions, or associations between categories (Strauss and Corbin, 1998). After completing all the coding work, this study carried out a theoretical saturation test. Roughly a quarter of the data from each of the three data sources was randomly reserved as an independent sample for recoding to check whether new concepts, categories, or associations have emerged. The results indicated that all newly generated coding units can be incorporated into the existing model, and no new categories or relational structures were detected, which confirmed that the theoretical model built has reached theoretical saturation.

## 5 Discussion

### 5.1 Antecedents of silence

Antecedents of silence serve as the contextual triggers that activate and shape Chinese partners’ silence. As shown in Figure 1, these antecedents do not exist in isolation; rather, they manifest as an interconnected chain of tensions encompassing structural value misalignments, situational norm conflicts, and linguistic-pragmatic constraints. Together, they position silence as the default interactive coping strategy during intercultural marital friction.

#### 5.1.1 Value misalignments: deep-seated cognitive underpinnings

The core of these antecedents lies in deep-rooted divergences in cultural and marital values. In daily marital life, Chinese partners often experience tensions when family-oriented responsibility encounters Western partners’ stronger emphasis on individual autonomy. As one participant explained:

“In China, we naturally value family harmony and try to accommodate every family member’s feelings. But people from Western countries prioritize individual choice and personal freedom. Because of this clash in mindsets, I usually choose to stay silent to cool down conflicts, rather than destroying our relationship through constant arguing.” (I7)

Similar tensions emerge from differences in communicative expectations. While Western partners may favor explicit verbal expression, Chinese partners may rely more heavily on contextual and implicit meanings, sometimes using silence to signal a stance without initiating direct confrontation:

“Westerners are used to laying everything out on the table and speaking explicitly, whereas we Chinese rely heavily on the immediate context and what goes unsaid. So as the Chinese spouse, I sometimes prefer to express my stance through silence. There is actually a lot of unspoken meaning embedded within that silence.” (I12)

Such value misalignments also become salient in ideologically sensitive domains such as religion and childrearing. For example, one participant described remaining silent when her husband repeatedly raised the issue of baptizing their child:

“I’m an atheist, but my American husband is a devout Catholic. He goes to church every week and wants our child to be baptized. I wasn’t really on board, so he thinks I disrespect his faith. Since then, whenever the topic comes up, I just keep my mouth shut to avoid arguments.” (I3)

These core conceptual conflicts extend directly into marital value systems, where Chinese partners experience fierce friction over extended kinship obligations and strong parenting expectations. The Confucian classic motto, “to govern the state, one must first regulate one’s own family,” emphasizes the primacy of family responsibility, which is in stark contrast to the Western concept of emphasizing personal boundaries and egalitarian negotiation. Moreover, traditional gender role expectations encapsulated in “men ruling the public sphere and women managing the domestic domain” expanded kinship obligations rooted in filial piety and family status, and parents’ high expectations for their children’s achievements, often clash with Western ideals of gender equality, nuclear family priority, and children’s independent development. As predicted by relevant research, Chinese people are more in agreement with the standard of family responsibility and the traditional concept of gender roles than Westerners (Marshall, 2008; Hiew et al., 2015a). This misalignment coincides with Hofstede’s (1980) cultural dimensions theory and Hall’s (1976) high-context communication paradigm. In China’s collectivist society, personal interests are often subordinate to collective well-being and family harmony; as the basic unit of this collective structure, the family imposes a shared obligation on both spouses to maintain family harmony and collective honor (Liao, 2015). In addition, high-context communication in Chinese culture conveys unstated implications, with non-verbal cues functioning as an indispensable part of the whole information transmission (Würtz, 2005).

However, these cultural orientations alone do not determine whether partners remain silent. Rather, the present findings suggest that cultural orientations become consequential when they encounter competing relational expectations within intercultural marriages. This pattern resonates with Baxter and Montgomery’s (1996) relational dialectics perspective, which conceptualizes intimate relationships as sites of persistent tensions between seemingly contradictory needs, such as autonomy and connection, openness and privacy, and individual expression and relational continuity. In the present study, the conflict between family-oriented relational obligations and individual autonomy creates precisely such a dialectical tension. Silence consequently emerges not simply as a culturally predetermined response, but as a communicative resource through which Chinese partners negotiate competing relational demands while attempting to preserve both relational continuity and personal positions.

The family-centered relational logic is not unique to intimate interactions but constitutes a foundational discursive framework that runs through Chinese social and political communication. As Li and Shang (2026) demonstrate, family metaphors in Chinese domestic narratives are anchored in a vertical logic of belonging, responsibility, and family-state isomorphism, whereas in global cross-cultural narratives, markers of hierarchy and bloodline are strategically downplayed in favor of horizontal framings of cooperation and solidarity. This macro-level discursive adaptation mirrors the micro-level dynamics observed in intercultural marriages: Chinese partners uphold family-oriented values internally while resorting to silence as a cautious, low-conflict mode of expression when engaging with Western partners, so as to avoid cultural friction.

#### 5.1.2 Situational norm conflicts: interactional rule contradictions

Situational norm conflicts are manifested as behavioral friction in daily interaction, mainly concentrated around the division of household labor, lifestyle habits, and financial philosophies. First, when Western expectations of reciprocal participation collide with the deep-rooted division of gender roles, Chinese partners often choose to remain silent to avoid exhausting conflicts (T9). Second, cross-cultural lifestyle mismatches—spanning physiological rhythms, daily routines, and dietary habits—generate continuous, subtle friction in everyday cohabitation. As one participant shared:

“There’s still a massive gap between our daily habits in a Sino-American household. I’m used to hot meals and warm water, but he cannot survive without salads and iced drinks for every single meal. I keep an early schedule, but he often stays up late hanging out. After countless fruitless attempts to compromise, I just stop nagging him and accept these cultural gaps quietly, sticking to our respective routines.” (I15)

In addition, divergences in financial philosophies frequently trigger severe interactional tension. When negotiations between long-term security and immediate consumption reach an impasse, Chinese partners often withdraw verbally to prevent escalations. One interviewee illustrates this strategic retreat:

“I wanted to save money to buy a house for our kids and support my aging parents, but he believed in living for the moment and spending freely on travel. After trying to talk to him so many times with zero results, I gave up further persuasion and saved money privately, taking silence as a way to prevent clashes over financial ideas” (I1)

The friction within these families is structurally exacerbated by divergences in gender interaction patterns, which are the cornerstone of the rules of daily interaction between husband and wife. This mismatch is reflected in the style of expression: Chinese people tend to be introspective and prudent, which conflicts with the Western norm of rapid verbal turn-taking. More than half of the female participants mentioned that although their silence is intended to express respect or thoughtfulness, Western partners routinely misunderstand it as intentional stonewalling or refusal to engage. As Wang (2014) pointed out, Chinese people, especially Chinese women, who are deeply influenced by reserved cultural dispositions, convey emotions through obscure and intuitive understanding, while Western utilitarian communication prioritizes straightforward verbal expression and tends to misinterpret those subtle and unspoken sentiments. This mismatch has led to a profound disconnect between affective transmission and conflict reconciliation, as the obscure emotional signals sent by Chinese female partners often cannot be responded to by partners.

Importantly, these findings suggest that situational conflicts should not be understood simply as manifestations of cultural difference. The communicative significance of a given conflict is shaped by the relational domain in which it occurs. Bearman and Parigi (2004) argue that conversational topics are structured by relational positions and social-network contexts rather than being randomly distributed across interaction partners. Extending this relational perspective to intercultural marriage, the present study shows that spouses occupy multiple and overlapping relational roles simultaneously: romantic partners, household members, co-parents, financial partners, and mediators of extended-family relationships. Consequently, conflicts over domestic labor, finances, parenting, religion, or kinship carry different relational stakes and may elicit different forms of silence. Silence is therefore topic-sensitive and relationally contingent rather than a uniform cultural response.

#### 5.1.3 L2 pragmatic barriers: expressive condition impediments

Thirdly, L2 pragmatic barriers impose objective constraints on communication practice and shape the expressive environment between partners. The constraints on pragmalinguistic competence stem from the lack of absolute language proficiency, which is mainly manifested in the limited vocabulary and hesitation in the use of syntax. Given that language proficiency is crucial to effective communication in intercultural marriages (Renalds, 2011), these lexical and syntactic limitations directly hinder the accurate communication and interpretation of complex emotional states. This linguistic vulnerability resonates with Dewaele’s (2018) findings that individuals who adopt their partners’ L1 often face greater difficulties in communicating emotions, largely driven by pragmalinguistic limitations such as lexical constraints and misinterpretations of the pragmatic force of expressions. Many interviewees said that it is difficult to express subtle inner feelings immediately when faced with a lack of English vocabulary or the inability to use complex grammar fluently. This difficulty causes linguistic anxiety and cognitive fatigue, making silence an efficient conversational alternative. Concurrently, deficiencies in sociopragmatic adaptation reflect the difficulty of navigating interactional norms, registers, and politeness strategies of specific situations in a L2. Out of concern about unintentionally violating pragmatic conventions, committing FTAs, or causing misunderstandings due to inauthentic expression, Chinese partners may strategically mobilize silence as a means of reducing risks. These barriers are consistent with the sociopragmatic friction identified by Dewaele (2018), wherein cultural variations in the verbal and non-verbal display of emotions, alongside the inability to translate culturally-specific emotion concepts into the LX, constrain LX users. By withholding verbal input, they maintain their sociopragmatic boundaries, turning an instrumental deficit into a structured and defensive interactional resource.

### 5.2 Manifestations of silence

Manifestations of silence include various behavioral practices enacted by Chinese partners in intercultural marital interactions. These manifestations do not represent passive communicative retreat, but agentic deployments in non-verbal space to guide divergent cultural logics, relational strategies, and identity claims. Based on the main behavioral orientations and potential relational motivations, these practices are categorized into three types: intimacy-maintaining, boundary-preserving, and mutuality-enhancing silences. These orientations are dynamic, shifting fluidly in response to evolving conflict intensities and situational pressures.

#### 5.2.1 Intimacy-maintaining silence: altruistic relational preservation

Characterized by altruism, intimacy-maintaining silence prioritizes marital stability, extended family cohesion, and the emotional well-being of the spouse above personal self-expression. This orientation is mainly manifested in conflict-avoidant silence—a deliberate relational protection strategy adopted in deep-rooted cultural divergences.

In the face of competing ideological frameworks, Chinese partners strategically deploy silence to avoid fruitless debate. As one participant reflected:

“He always says that people should live for themselves first, which completely goes against my family-first belief. But I’ve never argued with him about it. It’s hard to bridge the ideological gap between Chinese and British cultures anyway, so choosing not to poke at it through silence maintains our domestic harmony far better than trying to prove who’s right.” (I10)

Similarly, when addressing sensitive topics such as politics or religion, silence serves as a strategic mechanism to redirect interaction without triggering confrontation. As one participant illustrated when discussing conversations about Chinese politics with her Western husband: “Whenever my husband starts talking about Chinese politics, I simply do not bite. By staying silent and shifting the focus away from sensitive topics, I avoid offending him without letting things get awkward.” (I5).

Following intense disputes over deeply ingrained cultural norms, such as child-rearing philosophies, silence operates as a temporal and spatial buffer to mitigate escalation and preserve psychological safety. Reflecting on a heated disagreement over parenting styles, one interviewee explained:

“We fell out over parenting styles. I then stayed alone in the study for half an hour. Cultural divergence calls for calm reflection. Silence gives both sides breathing space and prevents hurtful words spoken out of impulse, which makes subsequent communication much smoother.” (I18)

These patterns resonate with Stafford and Canary’s (1991) conceptualization of relational maintenance as a set of communicative practices through which partners sustain relational commitment and satisfaction. Extending this perspective, the present findings identify silence as an underexamined form of relational maintenance. Rather than relying on explicit reassurance or continuous verbal engagement, participants sometimes withheld speech to prevent escalation, preserve emotional space, and accommodate culturally sensitive differences. Thus, silence does not necessarily represent the absence of relational maintenance; under specific relational conditions, it can constitute maintenance through communicative restraint.

This maintenance orientation also extends to forbearing-acquiescent and intergenerational-accommodating silence. The former involves temporarily suppressing personal preferences to sustain household functioning, while the latter operates across extended kinship networks, where Chinese partners may remain silent toward Western in-laws to respect their spouse’s familial norms and minimize intergenerational friction. Although these practices are partly informed by culturally embedded orientations toward family harmony (Gu, 2004; Lee and Mock, 2005), their relational function is more fundamental: silence becomes a means through which participants prioritize relational continuity over immediate self-expression.

#### 5.2.2 Boundary-preserving silence: self-oriented identity defense

In contrast to relational compliance, boundary-preserving silence adopts a self-oriented attitude as an active defensive strategy to safeguard individuals’ emotional boundaries, cultural integrity, and subjective rights. Within intercultural dynamics often complicated by power asymmetries, this typology converts non-responsiveness into a protective shield. Its core variant, defensive silence, is used to reduce emotional harm, resolve cultural offenses, and protect psychological boundaries. For instance, in the face of criticism of Chinese parenting styles, one interviewee (I4) consciously chose not to respond verbally, because “arguing would only make him dig his heels into his prejudices, and it would end up hurting my mood.” By using silence as a strategic retreat to avoid ineffective conflicts, she minimized emotional consumption and prevented disputes from escalating into cultural antagonism. Moreover, adversarial silence and culturally anchored silence are reinforced variants of this self-oriented boundary-preserving posture. Adversarial silence disrupts Western stereotypes of Eastern passivity by transforming the calculated absence of voice into a poignant, non-cooperative protest that communicates profound dissatisfaction. Simultaneously, culturally anchored silence is the key mechanism for maintaining identity, where silence is deployed to resist cultural offense and assimilation, so that individuals can firmly stand on their own ethnic roots and cultural inheritance.

Whereas intimacy-maintaining silence prioritizes relational continuity, boundary-preserving silence foregrounds the preservation of autonomy and self-integrity. This distinction resonates with Baxter and Montgomery’s (1996) relational dialectics perspective, particularly the persistent tension between connection and autonomy in intimate relationships. We found that silence can operate on both sides of this relational tension: it may be used to protect the relationship from escalation, but it may also be used to resist unwanted relational demands and safeguard the self. This finding complicates culturally deterministic interpretations of Chinese silence as accommodation, compliance, or face-saving. Rather than representing a uniform tendency toward relational submission, silence can constitute an agentic practice through which Chinese partners simultaneously negotiate relational continuity and defend personal and cultural boundaries.

#### 5.2.3 Mutuality-enhancing silence: synergistic relational adaptation

As a manifestation of the evolution of the relationship to the advanced stage, mutuality-enhancing silence focuses more on balancing the needs of both sides to co-create shared interactional meaning, thus promoting relationship adaptation and continuous cultural negotiation. Tacit silence is a hallmark of mature dyadic adaptation, reflecting the unspoken pace between partners and the mutual tolerance of cultural differences. Partners no longer argue about the differences in daily life, but reach a silent compromise, such as silently adapting to different dietary habits or arranging their own holiday activities, making silence an authentic carrier of cultural integration. Furthermore, affective-expressive silence leverages traditional Chinese emotional containment to deliver implicit intimacy and companionship. In the face of Western partners’ straightforward and romantic expressions of love, Chinese partners often replace long verbal confessions with implicit and restrained affective expressions (O6, I13). This low-key interaction integrates emotions into actual actions rather than straightforward words, providing a low-pressure communication practice, which not only respects the personality traits of Chinese partners but also meets the emotional needs of Western partners. Finally, digital-buffered silence adds an adaptive dimension mediated by technology to modern intercultural intimate relationships. By using interface functions such as muting, asynchronous messages, silent likes, or switching communication mediums, Chinese partners can temporarily withdraw from the high-pressure L2 processing environments. This digital buffer alleviates the problems of immediate linguistic anxiety and mismatch of communication rhythm, and maintains the relationship bond through a managed non-presence state, which has become a vital strategy for intercultural relations to adapt in the digital age.

### 5.3 Adjustment strategies for silence

Adjustment strategies for silence function as key adaptive interventions, which aim to deal with the disadvantages of non-verbal non-responsiveness, mainly its susceptibility to misinterpretation and tendency to exacerbate intercultural marital friction. These strategies take the initiative to adjust the direction of the complex manifestations of silence and transform it from a catalyst for conflict into a constructive resource to promote the coordination of relations. These adaptive strategies uphold across two main pathways: proactive individual adjustment and collaborative dyadic adjustment. The former relies on self-optimization to achieve unilateral adjustment, while the latter uses cooperation between partners to forge a bilateral consensus.

#### 5.3.1 The autonomous pathway: proactive individual adjustment

The autonomous pathway entails the unilateral cognitive and behavioral optimization of Chinese partners in terms of communication intentions, behavioral boundaries, and expressions to reduce the ambiguity of silence and make up for L2 deficiencies. One of the main mechanisms in this pathway is meta-communicative elucidation. As an interviewee (I11) said: “During the first few years of our marriage, I used to shut down and stay silent a lot. But now, I’ve become much more proactive in explaining the reasons and meanings behind my silence so he is not left guessing.” This shift reflects a propensity of Chinese partners to actively interpret their non-verbal states to Western spouses, so as to effectively bridge the gap between high- and low-context cultures, and to resolve negative suspicions and unnecessary doubts in advance. Chinese partners also regulate the temporal boundaries of their silence to relieve Western anxiety surrounding relational avoidance. By clearly framing silence as a limited and rational pause, such as requesting “five minutes to think” (I16), they not only obtained the necessary space for cognitive reflection, but also met the needs of low-context culture for explicit verbal signaling. Furthermore, to overcome linguistic-pragmatic constraints, Chinese partners frequently mobilize multimodal compensation strategies. As one participant vividly described:

“Sometimes my English fails to capture exactly how I feel, so I’ll use gestures, eye contact, and facial expressions to show him that I’m not angry. Gradually, he gets used to this and patiently waits for me to voice my thoughts.” (I9)

Such practices help Western partners gradually become familiar with the non-verbal logic of their Chinese spouses, establishing shared interpretive expectations and alleviating communication pressure caused by L2 limitations. Although participants adjusted aspects of their communicative behavior in response to their partners’ expectations, they did not necessarily replace Chinese communicative norms with Western ones. Rather, they recalibrated the timing, duration, explanation, and multimodal signaling of silence according to the evolving needs of the relationship. In this sense, adaptation is better understood as relational recalibration, an ongoing adjustment of communicative practices through which individuals preserve elements of their existing communicative repertoire while making them more intelligible and workable within an intercultural relationship.

In general, these adaptive behaviors of Chinese partners are in line with the core principles of acculturation, which involve communicative adaptation, the acquisition of new cultural practices and languages, as well as the cognitive processing driven by exposure to diverse cultural norms (Renalds, 2011). This proactive alignment with low-context communication expectations resonates with broader empirical findings in cross-cultural relationship management. Specifically, Zhang et al. (2025) find that dialogue expansion—strategies that cite reliable external sources and open negotiable conversational space, thereby enhancing credibility and trust—significantly improves Facebook communication effectiveness, whereas dialogic contraction shows no significant effect. In a similar vein, Chinese spouses’ deployment of meta-communicative elucidation and multimodal signaling serves as an interpersonal form of dialogue expansion, actively opening up interpretive space to mitigate cross-cultural misattribution and cultivate dyadic trust.

#### 5.3.2 The synergistic pathway: collaborative dyadic adjustment

The synergistic pathway shifts the focus of analysis from unilateral response to bilateral coordination. In the process, partners build shared interactional rules based on mutual understanding. This pathway is driven by consensus, joint rule-making, and cultural integration, which together foster relational maturity. To reconcile divergent cultural interpretations of silence, partners collaboratively negotiate its meanings to eliminate cognitive biases. As one interviewee explicitly noted when describing their relational pact: “We’ve made a mutual pact: brief silence simply means I’m processing my thoughts, whereas prolonged silence paired with a dark expression indicates bad moods. Now, he rarely misinterprets my reflective silence as indifference or emotional evasion.” (I20) By establishing this shared benchmark, the couple dismantles culturalist misattributions and prevents Western partners from mistaking reflective silence for hostility or escape. In addition to symbolic alignment, partners establish concrete behavioral protocols to manage post-conflict silence and prevent it from deteriorating into emotional alienation. For instance, couples implement time-bound rules to bridge the gap between Eastern emotional introversion and Western preferences for immediate verbal resolution: “We agreed to break the ice actively if silence lasts over ten minutes after quarrels, so as to eliminate misunderstandings and prolonged cold wars.” (I19) Over time, such sustained bidirectional accommodation across daily routines—from dietary choices to festive celebrations—gradually alleviates the underlying value friction that triggers conflicts. As another participant reflected:

“I find myself going silent much less often now. Based on mutual understanding and respect, my partner and I are actively trying to integrate two cultures and embrace differences. We learn and compromise on dietary habits, celebrate festivals from both countries, and share personal demands and feelings candidly.” (I14)

On the whole, this collaborative trajectory reflects Kim’s (2001, 2008) stress-adaptation-growth dynamic. By systematically resolving the internal imbalance caused by intercultural friction, partners have redefined marital pressure not as a catalyst for retreating avoidance, but as a transformative pivot toward a reciprocal person-environment fit, gradually turning silence into a synchronous and adaptive relational resource.

### 5.4 Consequences of silence

The consequences of silence are the result of a series of chain reactions in the intercultural marital system. Driven by previous adaptive interventions, these results unfold along a progressive continuum, covering three different levels: the regulation of marital relationships, the reconstruction of power dynamics, and the progression of cultural integration. Ultimately, these multi-dimensional shifts have had a profound impact on the long-term stability and evolution of Sino-Western intercultural marriages.

#### 5.4.1 The micro level: regulation of marital relationships

At the micro level, silence plays a crucial role in maintaining relationships, directly shaping and solidifying the couples’ intimate bond. The strategic use of silence is not to intensify conflicts, but to open up a cognitive and behavioral buffer zone to accommodate cross-cultural divergences. For instance, in parenting disputes, the Chinese partner deliberately restrains the immediate oral defense and temporarily conforms to the spouses’ practice. This non-confrontational response not only eases the current tension, but also leaves room for reflection for Western partners to come up with a more collaborative and feasible compromise. Similarly, the harmony of daily life is also maintained through silence, such as silently preparing a double menu to cope with dietary discrepancies, which implicitly guides Western partners to contact and experience Chinese culinary traditions. Furthermore, silence also strengthens the relationship of trust through pragmatic actions. Chinese partners continue to make sustained, quiet investments in dealing with intercultural affairs and family care, gradually establishing a reliable and empathetic image, thus deepening the mutual trust within the dyad.

#### 5.4.2 The meso level: reconstruction of power dynamics

At the meso level, the influence of silence transcends the immediate interactive situation, reshapes internal family power structures, and steers the intercultural household toward more egalitarian patterns. Chinese partners replace straightforward emotional disputes with rational restraint, effectively disrupting the unilateral dominant pattern that often disadvantages minority-culture spouses. This prudent non-responsiveness compels both sides to be restrained in communication, thus establishing a collaborative benchmark for balanced negotiation. At the same time, using silence to adhere to one’s own cultural customs while sticking to the core position is not a submission under subordinate status, but a firm declaration of cultural agency. This steady behavior gradually deconstructs the stereotype formed by Western partners based on their own culture, allowing Chinese spouses to gain greater discursive and negotiation space in core areas such as household decision-making and financial management.

#### 5.4.3 The macro level: progression of cultural integration

At the macro level, silence deeply shapes cultural integration in the marital field by deepening mutual empathy, promoting negotiated consensus, and strengthening cultural identity. It acts as a key symbolic medium to help Western spouses interpret the inherent logic of East Asian communication, thus promoting mutual cultural adaptation between the two sides and eliminating cross-cultural cognitive barriers. As one participant (I22) reflected, although the Western partner could not understand her silence at first, sustained exposure turned it into a recognized and unique way of communication, prompting both of them to “put ourselves in each other’s shoes, deeply embracing and respecting each other’s cultural backgrounds.” This cognitive shift promotes the true acceptance of heterogeneous communicative norms. Silence provides the necessary space for emotional buffering and an opportunity for rational reflection, thus alleviating affective confrontation, enabling partners to reach consensus results, and then naturally integrating the Chinese and Western cultural frameworks to form a synergistic effect. These results complement the perspective that while cross-cultural dyads inevitably encounter episodic emotional difficulties and misunderstandings, they progressively embrace the resulting cultural hybridity developed over time, leveraging deep affective bonds to bridge inherent linguistic gaps and co-create a unique, shared multicultural relationship (Dewaele and Salomidou, 2017; Dewaele, 2018). Ultimately, situating micro-level spousal communication within the broader macro-cultural landscape reveals a shared logic governing Chinese communicative practices across scales: whether at the level of international discursive engagement—where traditional hierarchical paradigms are reshaped into horizontal, cooperative narratives to bridge cross-cultural divides (Li and Shang, 2026; Zhang et al., 2025)—or at the level of interpersonal dynamics, Chinese actors continuously adapt, negotiate, and bridge Eastern and Western communicative frameworks to cultivate sustainable, mutually recognized relational spaces.

## 6 Conclusion

### 6.1 Research findings

This study employs proceduralized grounded theory to develop a processual model of the dynamic adaptation mechanism underlying Chinese partners’ silence in Sino-Western intercultural marriages. The findings reveal that silence is not merely a passive communicative breakdown, but an agentive relational practice shaped by contextual conditions and interactional demands. Triggered by value misalignments, situational norm conflicts, and L2 pragmatic barriers, communication tensions may lead Chinese partners to adopt silent responses as a means of managing relational challenges. As the core phenomenon, these silent practices exhibit a dynamic three-dimensional structure, encompassing intimacy-maintaining silence, boundary-preserving silence, and mutuality-enhancing silence. To address the ambiguity inherent in non-verbal communication, partners engage in adjustment processes via autonomous and synergistic pathways. Through these processes, silence can be transformed from a potential source of misunderstanding into a constructive relational resource that reshapes marital interactions, interpersonal power negotiation, and cultural integration. Overall, Chinese partners’ silence represents a complex relational practice through which individuals balance cultural identities, emotional expression, and adaptation to intercultural intimate relationships.

### 6.2 Research contributions

Theoretically, this study extends research on silence from predominantly public and institutional contexts to the private domain of intercultural intimacy, which remains comparatively underexplored. By revealing how structural conditions, cultural orientations, and individual agency interact in shaping non-verbal communicative practices, this study challenges simplistic interpretations of silence as either avoidance or disengagement. Instead, it conceptualizes silence as a dynamic and context-dependent relational practice that contributes to a deeper understanding of intercultural communication and relationship adaptation. Practically, the findings provide valuable insights for intercultural couples, relationship counselors, and communication practitioners by highlighting the importance of interpreting silence within specific cultural and relational contexts. The proposed framework may inform the development of culturally sensitive counseling approaches and communication training programs aimed at reducing misunderstandings, enhancing empathic understanding, and facilitating more adaptive interaction patterns in intercultural marriages. By recognizing the agentive and relational functions of silence, individuals and practitioners can better support couples in navigating cultural differences and managing interpersonal challenges in increasingly diverse social contexts.

### 6.3 Research limitations and future directions

Several limitations should be acknowledged. First, the sample focused on relatively highly educated Chinese partners, which fails to fully cover the groups in unstable immigration situations; second, the qualitative research design makes the functional weight and universality of the core category impossible to quantify; third, the perspective is relatively single, and does not fully explore the experience of Western partners. Therefore, future research can expand the demographic scope of the sample for comparative analysis between different socio-economic and immigrant classes. In addition, it is recommended to use a mixed-methods research method to verify the structural stability and universality of the conceptual framework. Finally, adopting a bi-directional, fully dyadic perspective will help subsequent researchers deeply analyze the coordinated adaptation mechanism of silence in Sino-Western intercultural marriages, so as to better understand its important role in the construction of intimate relationships.

## Data Availability

The raw data supporting the conclusions of this article will be made available by the authors, without undue reservation.
