# Supplementary material for: Silence as relational adaptation: Strategic communicative practices of Chinese partners in Sino-Western intercultural marriages
Source: Front Psychol. 2026 Oct 16;17:1957138. doi: 10.3389/fpsyg.2026.1957138 (PMC13719481; doi:10.3389/fpsyg.2026.1957138)
Supplement: Supplementary file 1 [file Supplementary_file_1.DOCX]

***Supplementary Material***

# 1 **Supplementary Semi-structured Interview Data**

**I-01: (Female, US partner, with children, married > 10 years)**

Researcher: Looking back on the past decade or so you’ve spent together, what do you think were the most significant impacts of your differing cultural backgrounds, such as values, mindsets, and communication styles, on your intimate relationship?

Interviewee: The impact really permeated every aspect of our lives; there were positive sides, of course, but also plenty of conflicts. There was the fundamental difference in our concepts of family. I have a typical Chinese mindset: I view the “family” as a single unit and the couple as sharing a common interest—what’s yours is mine, and what’s mine is yours. I believe in prioritizing the family’s long-term plans and considering the needs of both children and elderly parents. He, on the other hand, is a classic individualist. He believes that even within a marriage, we are first and foremost two independent individuals. He feels he has the right to control the money he earns and that his private time shouldn’t be encroached upon; he places a strong emphasis on personal boundaries. I’ve gradually come to accept this, acknowledging that everyone needs their own space, but at first, I really struggled to adjust; I often felt like he was keeping me at arm’s length. Then there were our communication styles. I’m used to being indirect, holding back a bit to save face for the other person and leave myself an out. If he did something wrong, I wouldn’t point it out directly; I’d drop hints and wait for him to figure it out himself. But that approach didn’t work on him at all. He told me several times, “If you have something to say, just say it directly. Don’t make me guess. I can’t read your mind.” It went both ways, too; he was very blunt. Sometimes a single remark from him would leave me speechless, yet he’d genuinely wonder, “I was just stating facts. Why are you getting angry?” Then there were our views on spending, child-rearing, and caring for aging parents; cultural differences lay behind almost every major issue. It’s not a matter of who was right or wrong, but when you’re living together, these differences make friction almost inevitable.

Researcher: Among these differences, which one do you find the hardest to reconcile, the one that makes you feel the most helpless?

Interviewee: It has to be our fundamental views on family responsibility, especially regarding the care of elderly parents and child-rearing. To me, supporting one’s parents is a natural duty and a responsibility. I, as an only child, simply have to shoulder it. My parents are back in my hometown, and as they age, their health is declining. I feel I absolutely must save money for their old age; if they were to be hospitalized or need a caregiver down the line, how could I manage without having the funds ready? But he just doesn’t get it. He feels that since his parents have their own pensions and health insurance, they should take care of themselves, or go to a nursing home if necessary, and that their children have no obligation to sacrifice their own quality of life for them. It’s not that he’s unfilial; he buys gifts for my parents on holidays and is patient when visiting them. However, he simply cannot grasp that deeply ingrained Chinese sense of responsibility—the feeling that one must shoulder the burden of one’s parents’ later years. It’s something etched into one’s bones from childhood; you could explain it a hundred times, but he’d only understand the literal words without truly feeling the underlying anxiety and pressure. Arguing about it just becomes exhausting. It’s like talking to a brick wall.

Researcher: Right, let’s get back to the main topic. Do you ever go silent when interacting with your partner or during conflicts? If so, could you describe one or two specific instances that left the strongest impression on you? For example, what was happening, what was the atmosphere like, how long did the silence last, and what was going through your mind when you chose to stay silent?

Interviewee: Oh, definitely, and quite often, too; the longer we’ve been married, the more frequent the silence has become. The instance that stands out most was New Year’s Eve last year. We had planned to settle our accounts and watch the New Year’s gala together after the kids went to sleep; we even had red wine and strawberries set out in the living room. It was quite a nice atmosphere. But then he pulled up the credit card statements for the year, and I saw that we’d spent nearly 150,000 yuan just on travel and outdoor gear. That year, we’d taken trips to Iceland and Japan, plus three or four short camping trips domestically. He’d bought a professional mountaineering tent, a carbon-fiber bicycle, and a full set of skiing equipment—all sorts of things that added up to over a hundred thousand yuan. My temper flared up instantly. I told him we couldn’t keep spending like that: our child would be entering middle school in two years, we hadn’t secured a home in a good school district yet, and we were still far short of the down payment. Plus, my parents were getting older and needed long-term medication for high blood pressure and diabetes; we needed emergency funds in case of a serious illness later on. He couldn’t understand my point at all. He asked why we absolutely had to buy another place when our current home was big enough. He said money is meant for experiencing life. Why wait until we’re old and can barely walk to go out and have fun? We argued a bit, and our voices kept getting louder as the conversation went on. He felt I was constantly anxious about the future, worrying needlessly, and incapable of enjoying life. I, on the other hand, saw him as selfish; he cared only about his own happiness and gave no thought to the family’s long-term needs. At the height of one argument, I suddenly felt drained of all energy, and I didn’t want to say another word. I wanted to save money to buy a house for our kids and support my aging parents, but he believed in living for the moment and spending freely on travel. After trying to talk to him so many times with zero results, I gave up further persuasion and saved money privately, taking silence as a way to prevent clashes over financial ideas.

Researcher: So, you just stopped speaking abruptly at that point? How long did the silence last? What did you do during that time?

Interviewee: Yes, at that moment in the argument, I suddenly felt utterly powerless. I realized that continuing the fight would just mean repeating the same old things. It wouldn’t solve anything. I set my tablet down on the table and, without saying a word to him, turned and walked into the kitchen. I had already washed the dishes earlier that evening, but I took them out and washed them all over again. Then I wiped down the stove, tidied the fridge, and took out food to thaw for the next day. I deliberately dawdled for over an hour. I just didn’t want to go out there and face him. The living room was quiet; he didn’t follow me, and all I could hear was the sound of the New Year’s Eve gala playing on the TV. Standing by the kitchen sink with the water rushing loudly, I felt a chaotic mix of emotions. At first, I was angry. I felt he was so immature; a man in his mid-thirties with absolutely no plan for the future. As I washed, that anger turned into a sense of grievance; I was constantly scrimping and saving, while he was the opposite, spending money recklessly without a second thought. But eventually, all that remained was a feeling of utter powerlessness. We’ve had this conversation countless times; we argue about it every year-end when we take stock of things. Every time I bring up domestic housing prices, the burden of caring for the elderly, or the fierce competition in education, he counters by citing the situation in the US. We go back and forth, but neither of us can convince the other; in the end, it achieves nothing but hurt feelings. At that moment, I just thought: Forget it. There’s no point in saying anything. At least silence wouldn’t escalate the conflict or completely ruin New Year’s Eve.

Researcher: I see. What was the other instance of silence that left a deep impression on you?

Interviewee: There was another time, last year, around the midterms. Our child scored in the seventies in math, and his class ranking dropped by over twenty places. I suggested to my husband that we sign him up for math and English tutoring classes on the weekend; otherwise, he wouldn’t be able to keep up. He blew up right then and there, saying the child was already exhausted from school and should spend weekends playing outdoors or doing sports. He claimed that tutoring classes would rob him of his childhood and turn him into a mindless drone. It was dinnertime, and our son was sitting right there, shoveling down his food. My husband contradicted me right in front of him and even turned to our son and said, “Don’t worry, son. Dad won’t make you take extra classes. We’ll go cycling this weekend instead.” My face fell instantly, but I didn’t want to argue in front of the child, nor did I want him to see us disagreeing and then stop listening to us later on. So, I said nothing; I just kept my head down and finished my meal, then cleared the table and went into the kitchen. I remained silent for the rest of the evening. I barely spoke to him until our son had finished his homework and gone to bed. At one point, he tried to strike up a conversation and asked if I wanted some fruit, but I just shook my head without saying a word.

Researcher: When you chose silence that time, aside from not wanting to argue in front of your child, was there also a feeling that it was simply “impossible to get through to him”?

Interviewee: Absolutely. He’s convinced that “happy education” is the right approach; he thinks the education system here is too cutthroat and stifling—that it’s unnecessary. But he has no concept of what the Gaokao (college entrance exam) is actually like. It’s like thousands of troops trying to cross a single-plank bridge; a difference of just one point can mean the difference between getting in and being left behind by thousands of other students. He hasn’t experienced it himself, nor has he seen anyone around him go to such lengths for their child’s education; he simply tries to apply the American public education model to the reality in China. I’ve explained it to him countless times: we’re ordinary people, our child doesn’t hold foreign citizenship and will have to take the Gaokao (college entrance exam), so if we don’t put in the effort now, it’ll be too late. But he won’t listen; he just accuses me of stoking anxiety. Bringing it up only leads to arguments, so it’s better to say nothing. Besides, if I were to argue with him in front of the child, the child would feel emboldened and become even harder to discipline later on. So, silence was the only option at the time.

Researcher: Aside from those two scenarios, in what other specific situations do you tend to remain silent? For instance, when facing accusations from your partner, discussing sensitive topics, dealing with a clash of cultural values, feeling exhausted, or harboring feelings of grievance or anger?

Interviewee: There are quite a few situations. To sum it up, I choose silence whenever speaking would be futile or counterproductive. First, definitely when there’s a clash of cultural values; whenever the conversation touches on fundamental values, like financial management, elderly care, or child-rearing. I generally avoid deep discussions and simply clam up if we hit a point of disagreement. I know arguing won’t resolve anything; it’s just a waste of energy. I also stay silent when he gets really worked up emotionally. When his temper flares, he becomes very expressive, raising his voice, speaking rapidly, gesticulating wildly, and getting increasingly worked up as he talks. If I were to push back then, I’d just be adding fuel to the fire, making the argument worse. So, I stay quiet when he’s agitated and wait for the intensity to pass before bringing things up calmly. I’m also very prone to silence when the conversation involves our extended families. For example, my mother-in-law came to stay with us for a month last summer, and her lifestyle was completely different from ours. She’d get up after six in the morning and make a racket in the kitchen, insist on keeping all the windows wide open for ventilation, stuff the fridge with frozen foods, and even wash fruit with dish soap. I’m already tired from work, and while I’d love to sleep in on weekends, I was constantly being woken up. Yet, I never said anything to her face; I just bottled it up and stayed silent. I did mention it to him once, asking if he could suggest his mom be a bit quieter in the mornings. He turned right around and told my mother-in-law about it; she even went out of her way to apologize to me. It made me feel incredibly awkward, as if I were being overly critical or unreasonable. After that, I stopped saying anything about the elders’ lifestyle habits; I just put up with it and stayed silent. I also tend to go silent when I feel deeply aggrieved or hurt. For instance, if he says something particularly hurtful that strikes a raw nerve, I feel so choked up inside that I can’t speak; I just want to be alone. It’s not that I don’t want to express my hurt; it’s just that I feel he wouldn’t understand anyway. Instead, he’d likely think I was making a big deal out of nothing or being overly sensitive.

Researcher: Does the opposite ever happen? Are there times when you’re furious and speak up loudly rather than staying silent?

Interviewee: That happens too, but only with minor issues. For example, if he forgets to pick up the kids as promised, or makes a huge mess after drinking—when there’s a clear right and wrong. I get angry and call him out directly. But whenever it involves major issues like cultural differences or conflicting values, I tend to stay silent. Small matters have a clear right and wrong, so they can be sorted out. Big issues don’t; they stem from different upbringings. Arguing won’t lead to a resolution and only hurts the relationship, so I just suppress my feelings and compromise. I also definitely stay silent when others are around to save face for him. If we’re dining with friends and I disagree with something he says, I won’t contradict him on the spot; I’ll just smile and smooth things over, then bring it up privately later. He’s different, though. He speaks his mind freely. He’ll point out my faults right in front of friends. That actually makes me quite uncomfortable, though I’ve never really pressed the issue with him.

Researcher: What do you think is the main reason you choose silence? Is it to avoid arguments, a feeling that things can’t be explained clearly, a desire to maintain harmony, a need to save face (yours or his), the influence of cultural values, the pressure of expressing yourself in the language, or simply not knowing how to respond in the moment?

Interviewee: The reasons are quite complex. It’s a mix of several factors. The core reason is, first of all, that I don’t want to argue; I want to maintain family harmony. I grew up hearing my mother say, “Harmony in the family leads to prosperity in all things.” A home filled with constant bickering looks bad, and the neighbors would laugh at it. Especially after having a child, I really didn’t want him growing up in an environment filled with arguments. My parents fought constantly when I was young; I’d hide in my room crying, feeling utterly miserable. They would even start arguing the night before I had a major exam. So, once I had a child of my own, I vowed never to let him go through that. I’d choose not to speak up or just bite my tongue; taking a step back meant, at the very least, keeping the peace at home. Then there’s the fact that some things are simply impossible to explain, talking about them is futile. There are deep-seated cultural nuances that words alone can’t convey. Take the anxiety about saving money, for instance: I could tell him the price of housing per square meter in Shanghai or the monthly cost of a nursing home, but I couldn’t make him truly feel that deep-seated insecurity—that panic Chinese people feel when they don’t have money in the bank. Having grown up in a country with a robust social welfare system and never having experienced material scarcity, he simply couldn’t grasp that ingrained sense of crisis. Explaining it over and over just felt like talking to a brick wall—a complete waste of breath. Then there’s the issue of “face.” On one hand, I wanted to spare his dignity, avoiding embarrassing him in front of outsiders, our child, or elders. On the other hand, I wanted to save face for myself. If I mentioned wanting to save money for my parents’ retirement and he flatly refused, I’d feel incredibly humiliated and lose face. It was better to say nothing and save the money privately; that way, I could fulfill my filial duty without having to deal with his disapproval. There’s also the language barrier to consider. Even though his Chinese is quite good, there are still hurdles when it comes to expressing complex emotions. If I’m feeling a mix of grievance, anger, and helplessness—an intertwined web of feelings—I might struggle to articulate it clearly even in Chinese, let alone expect him to fully grasp the nuances. Often, the words would be on the tip of my tongue, but I’d swallow them back, feeling he wouldn’t truly understand the sentiment anyway. Finally, silence gives me time to cool off. I have a bit of a quick temper; when I’m angry, I tend to say harsh, hurtful things, whatever stings the most, only to regret it later. Staying silent for a while lets me calm down and think carefully about what should and shouldn’t be said, helping me avoid the damage caused by speaking rashly. Researcher: Among these reasons, if you had to rank them, which one do you consider the most fundamental?

Interviewee: The core issue is definitely the inability to make myself understood—the feeling that speaking up is pointless. If communication actually worked, who would choose silence? When we first got married, I wanted to lay everything out on the table and work through our differences. But after hitting a brick wall time and again, I realized that many of our differences run deep, ingrained by decades of upbringing, and simply cannot be changed. We argued countless times, but aside from eroding our affection for each other, nothing was ever resolved. Eventually, I learned the ropes; I figured out what could be discussed and what was a waste of breath. For the things we couldn’t resolve, I’d just shut up, using silence to keep the peace. To put it bluntly, it’s a kind of reluctant compromise.

Researcher: So, do you think silence might actually be a form of passive resistance, or a silent protest?

Interviewee: There’s a bit of that, sure, but it’s mostly a sense of resignation. For instance, he insisted on spending tens of thousands on outdoor gear; I disagreed but couldn’t stop him, so I just stayed silent, neither showing support nor picking a fight. You could call it a form of protest, I suppose, even though I didn’t physically block him. Then again, maybe it isn’t. I was simply using silence to express my disapproval. But really, it’s more about self-preservation. I didn’t want to get into a heated argument every time, leaving me fuming for ages while he just turned around and forgot all about it. Silence at least spared me some pain; I didn’t have to pin my emotions and expectations on whether or not he would understand me.

Researcher: So, what impact does your silence have on your communication or relationship? Does it maintain harmony and ease conflict, or does it escalate tensions, leave the other person confused or hurt, and create lingering resentment? Could you give some specific examples? Interviewee: It’s definitely a double-edged sword. There are pros and cons. On the positive side, it stops the conflict in its tracks and prevents the argument from spiraling out of control. Take the fight we had on New Year’s Eve, for example: if I’d kept arguing, I might have said something truly hurtful, and the whole holiday would have been ruined. By staying silent, I tamped down the anger; after a few hours, we both cooled off and moved past it. Silence also gives us both a buffer period. When people are angry, they say things out of spite—things they don’t actually mean. Cooling off for a while allows for a much more rational conversation later, without deliberately poking at each other’s sore spots. But the negative effects are obvious, too. For one thing, he misunderstands it as the “silent treatment,” thinking I’m deliberately ignoring or punishing him. He’s told me many times that my silence hurts him more than an actual argument does. During an argument, at least he knows what I’m thinking and where the conflict lies. When I’m silent, he can’t figure out what’s going on in my head; he feels anxious and deeply frustrated. Then there’s the fact that many issues never actually get resolved. They just get buried under the silence and turn into lingering resentment over time. Take the issue of saving money: we’ve never truly reached a consensus. Every time we argue about it, I eventually go silent and we move on, but the problem remains. It’s bound to blow up again the next time we look at our finances. It’s like a stone pressing down on the grass—the surface looks flat, but underneath, it’s constantly digging in and causing discomfort. Another thing is that too much silence creates a growing rift between two people. If you bottle things up long enough, you eventually lose the desire to speak at all. After a while, it feels like there’s nothing left to say. For instance, I never told him about the extra income I earned from side jobs; keeping that to myself created a barrier. I know couples shouldn’t keep secrets, but I also knew that bringing it up would trigger a huge fight. This feeling of hiding things really takes a toll on the relationship, yet I didn’t have a better option.

Researcher: So, looking at the big picture, do you think silence does more harm than good in your relationship, or vice versa?

Interviewee: It depends on the stage of the relationship. In the first few years of marriage, the downsides definitely outweighed the benefits; we didn’t understand each other well yet, so silence led to misunderstandings and actually hurt the relationship more. Now that we’ve been married over a decade and have largely adjusted to one another, he knows my silence isn’t a form of silent treatment or deliberate coldness. It just means I need to cool off or simply don’t want to argue anymore. At this stage, silence does more good than harm; it helps us avoid a lot of pointless bickering. But fundamentally, there are still many unresolved issues; we’ve just learned to work around them. It’s like having an obstacle in the room. Instead of moving it, we’ve gotten used to walking around it and pretending it isn’t there.

Researcher: How does your partner usually react to your silence? Does he ask what’s wrong, stay silent himself, show impatience, or try to draw you out? What do you think of these reactions?

Interviewee: There were definitely misunderstandings, and there still are, though far fewer than before. Early in our marriage, he couldn’t grasp the concept of “Chinese-style silence” at all. Whenever I stopped talking, he’d interpret it as the silent treatment—a way of punishing him. His immediate reaction was to pester me with questions: “What’s wrong?” “Say something.” “What exactly are you angry about?” He’d keep asking until I was completely exasperated. The less I said, the more anxious he became, eventually leading to accusations like, “This kind of silent treatment is so hurtful.” After about five or six years of marriage, and a few major arguments, he gradually figured out the pattern. He realized that I usually go silent when I’m upset over something minor and need time to process it myself, or when there’s a clash of values and I simply don’t want to argue anymore. His reactions are much more mature now. If it’s a minor issue and I go silent, he’ll give me some space at first. After ten or twenty minutes, he might come over and nuzzle up to me, give me a hug from behind, or hand me some cut fruit, using actions to show he wants to make amends. If it’s something more serious and I stay silent for a long time, he does get a bit impatient. He might say, “Can you stop doing this? Let’s just talk it out clearly,” but he doesn’t pressure me the way he used to; he knows that pushing doesn’t work.

Researcher: So, after the silence, is he usually the one to “break the ice” and resume communication?

Interviewee: Most of the time, he’s the one who takes the initiative. I rarely back down first—perhaps it’s that reserve ingrained in Chinese culture; I find it hard to swallow my pride. He has various ways of breaking the ice, but the most common is striking up a conversation about something mundane. For instance, after an hour or two of silence, he might come over and ask, “What do you want for dinner? I’ll make it.” It’s as if nothing happened; he starts with a casual remark, and the conversation flows from there, with neither of us mentioning the earlier conflict. Physical contact is another way. He might walk over and hug me from behind, resting his chin on my shoulder; without a word, I know he’s backing down and trying to make peace. If I don’t push him away, it means my anger has subsided and the matter is considered settled. Sometimes the child acts as a go-between. He might have our child run over and ask, “Mom, Dad says we’re going to the zoo this weekend. Are you coming?” He uses the child to give me a way to step down gracefully. After all, I wouldn’t keep sulking in front of the child.

Researcher: Have there been times when you were the one to break the ice? What were those situations like?

Interviewee: It has happened, though very rarely. Usually, it’s when I realize I was actually in the wrong. For example, once I saw a 20,000-yuan charge on his bank card that he hadn’t told me about. I assumed he’d gone on a shopping spree for gear again, so I gave him the silent treatment for quite a while. Later, I found out he had secretly booked a health check-up package for my parents as a surprise. Realizing I’d misjudged him, I took the initiative to cook his favorite dish—that was my way of making up. Also, if the silence drags on—say, the house has been quiet for the better part of a day, and I feel the atmosphere is too oppressive and bad for the child, I’ll initiate a conversation about something trivial. But the vast majority of the time, he’s the one who backs down first. He’s really good at that; he never gives me the silent treatment indefinitely.

Researcher: So, after the ice is broken, do you go back and talk about the topic that caused the silence in the first place? Or do you just move on?

Interviewee: It depends on the issue. If it’s something trivial, like forgetting a chore or saying the wrong thing, we move on completely once the ice is broken; neither of us brings it up again. But if it’s a major issue, like finances or parenting, we might discuss it a bit more after things cool down, though we stay very restrained and avoid heated arguments. Still, for most matters involving a clash of values, we usually just move past it. We both know deep down that talking about it won’t lead to a resolution and will only cause more unpleasantness.

Researcher: Do you ever explain to your partner later why you went silent? For instance, telling him, “I wasn’t speaking because I needed to calm down, not to give you the silent treatment”? Does that kind of explanation help reduce misunderstandings?

Interviewee: I didn’t do that at all when we were first married. I was young and a bit dramatic back then; I felt that if he understood me, I shouldn’t have to explain, and if he didn’t, explaining wouldn’t help anyway. But after arguing a lot, I realized he genuinely didn’t understand. It wasn’t that he was pretending not to. It was a cultural difference; that way of handling things simply didn’t exist in his mindset. I remember one particularly fierce argument where he asked, with red-rimmed eyes, “What exactly do you want?” I felt incredibly aggrieved, so I sat down and had a long, heart-to-heart talk with him. I explained that Chinese people express emotions differently. When we’re unhappy, we don’t shout or scream; instead, we go quiet. Silence isn’t about deliberately ignoring someone; it’s about processing my own emotions and avoiding saying hurtful things in anger. I told him, “My silence isn’t the silent treatment; it’s just how I handle my emotions. Just like you might go fix your motorcycle in the garage or go for a run when you’re angry, I just need a little time to myself.”

Researcher: How did he react after that explanation? Did it actually help reduce misunderstandings?

Interviewee: It did help. Since then, he doesn’t rush to question me the moment I stop talking, nor does he immediately assume I’m giving him the silent treatment. He gives me space and waits for me to compose myself. There was a time when I fell silent, and he even poured me a glass of hot water and set it nearby, saying, “Take your time thinking; call me when you’re ready to talk.” I was quite surprised by that gesture. But there were limits to that kind of help. He could understand that silence was my way of processing emotions, but he could never grasp why those specific things made me angry or why they mattered so much to me.

Researcher: From a cultural perspective, do you think certain concepts in Chinese culture, such as “harmony brings prosperity to the family,” “think thrice before acting,” or “silence is golden,” or perhaps your upbringing, influenced your choice to remain silent in an intercultural intimate relationship?

Interviewee: That definitely had a significant impact. You could say my way of handling things was shaped by Chinese culture and my family upbringing. Growing up, I constantly heard my parents and grandparents repeating the same message: family harmony is paramount. They believed a household filled with constant bickering was destined to fall apart and would become a laughingstock to outsiders. So, on a subconscious level, I came to view arguing as something bad, something that destroys the family, so I avoided it whenever possible. Silence became my tool for maintaining harmony; even if it was only a superficial peace, it was still better than chaos and screaming matches. My parents taught me from a young age to think before I speak—not to just blurt out whatever came to mind. They warned that talking too much leads to mistakes, offends people, and causes regret over ill-chosen words. This was especially true when angry; they said “trouble comes from the mouth,” so I shouldn’t speak rashly. Consequently, whenever I got emotional, my first instinct was to shut my mouth. If I didn’t speak, I wouldn’t say the wrong thing, wouldn’t hurt others, and wouldn’t invite trouble for myself. Chinese culture emphasizes propriety in speech, considering the other person’s feelings and the context, rather than being blunt or impulsive. Often, when emotions run high, it’s impossible to figure out the right thing to say, so the best approach is to stay silent until I’ve sorted out my thoughts. Sometimes, after thinking it over, I’d decide the issue wasn’t worth bringing up at all, voicing it might only damage the relationship. Then there’s the collectivist mindset. Chinese culture values the “big picture”; for the sake of overall harmony, one might sacrifice personal emotions and feelings. Many times, I chose silence for the sake of the family’s well-being. I could suppress my own grievances, dissatisfaction, and anger; as long as the family remained whole and harmonious, and the children could grow up in a stable environment. That was enough for me. He is completely different; he believes personal feelings are paramount. If you have emotions, you should express them; bottling them up is a form of self-abuse and harms the relationship. He could never understand why one would compromise oneself for the sake of the “bigger picture.” Beyond cultural differences, my upbringing played a huge role. My parents fought constantly when I was little; I’d hide in the closet and cry every time, terrified they’d divorce. I used to think that if they just stopped talking, the fighting would stop too. So, in my own marriage, whenever I sense a conflict brewing, I instinctively clam up, using silence to nip the argument in the bud.

Researcher: Do your close Chinese friends handle conflict with silence in their marriages, too? Comparing your communication style with theirs—that is, friends in non-intercultural marriages—do you think your silence differs from theirs? What are the main differences?

Interviewee: They do it too, especially those married for over a decade; we’re all pretty much the same. We often vent to each other, asking, “What’s the point of arguing with your husband?” You might win the argument, but you lose the emotional connection and end up feeling miserable anyway. It’s better to just say less and enjoy some peace and quiet. The difference is that for them, in same-culture marriages, silence is often a reaction to the fact that speaking up won’t change anything or just leads to more fighting. For me, in an intercultural marriage, silence is often because speaking up means he won’t understand anyway. Fundamentally, though, it’s all about compromise in marriage. It’s all born of resignation.

Researcher: Do you feel that this mindset of prioritizing harmony actually comes at the cost of your own well-being?

Interviewee: It definitely feels like a sacrifice—how could it not? Sometimes, in the quiet of the night, I realize how exhausted I am. I bottle everything up and constantly consider the bigger picture—the elders, the children, my husband’s feelings—while rarely thinking about myself. Sometimes I almost forget what my own temperament used to be like. But then again, that’s just marriage, isn’t it? Intercultural or not, someone in the marriage always has to compromise or yield. And looking at it from the other side, he’s compromising too. He’s adapting to my culture, learning to tolerate my silence, curbing his own blunt temper, and accepting my views on family. We’re accommodating each other; that’s how the relationship grows stronger. Researcher: Do you feel that the frequency or manner in which you use silence in your cross-cultural marriage has changed over the years? How does the present compare to the early days of your marriage?

Interviewee: In the first two years, there was very little silence. Everything was fresh, and our feelings were intense; whenever a problem arose, I wanted to communicate openly, believing that love could bridge any gap. Back then, if we had a conflict, I would patiently explain my perspective, sharing how Chinese people think and describing the social context in China. While there were moments of silence, they were just the kind of sulking a young woman might do when upset—a bit of a huff, and a few soothing words would fix it; I didn’t take it to heart. In the years that followed, the novelty wore off, the mundane realities of daily life set in, and the children were born; a whole host of conflicts erupted at once. We argued countless times and hit so many dead ends. I gradually realized that some things simply couldn’t be explained or resolved. As disappointment accumulated, the silence grew more frequent. Back then, the silence was laced with resentment—a “what’s the point of talking? You wouldn’t understand anyway” kind of sulking. We’d often give each other the silent treatment for days on end; it was emotionally draining. Now, after more than a dozen years of marriage, the silence is less frequent, but its nature has completely changed. It’s a selective silence now. For trivial matters, I speak my mind immediately. I don’t bottle it up. But when it comes to fundamental cultural clashes or issues that simply cannot be resolved, I just stay silent, no arguing, no fuss. There’s no resentment in this silence; it’s a calm, deliberate choice. I know talking won’t help, so I don’t bother; I save my energy for living my life. In the past, when I went silent, I’d ruminate endlessly, feeling increasingly aggrieved. Now, if I go silent, I just get on with things, washing dishes, watching TV series, spending time with the kids, without missing a beat, and my emotions settle quickly. It’s more like a self-protection mechanism I’ve developed over time.

Researcher: Do you think this change stems from you becoming more mature, or from having lowered your expectations for the relationship?

Interviewee: It’s a bit of both. On one hand, I’ve matured; I realize that marriage isn’t a place for debating logic. Many issues don’t have an absolute right or wrong, so there’s no need to fight to the death over them. Daily life isn’t an academic debate where a winner must be declared. On the other hand, my expectations have indeed lowered. When we first got married, I thought we could bridge our cultural differences and become soulmates—that we could talk about anything and face everything together. Now I realize that no matter how deep the bond, it is hard to cross the chasm created by our different upbringings. There are things he will never understand, and I will never be able to change him. With lower expectations come fewer disappointments, and silence becomes a state of calm rather than tension. It’s not that the love is gone; it’s about accepting our differences and acknowledging that marriage inherently involves many unsolvable problems. By being tolerant of each other and learning about one another’s cultures, we’ve found a way to share a quiet, mutual understanding.

Researcher: Alright, that covers just about everything on my list. Is there anything else regarding silence in your cross-cultural marriage, something I didn’t ask about but you feel is important, and you’d like to add?

Interviewee: I’d like to add one point: I hope people won’t immediately label silence in a marriage as “emotional abuse” or the “silent treatment.” Yet, silence in a cross-cultural marriage often isn’t about intentionally hurting the other person or falling out of love; it is a choice made out of necessity, a way of interacting that the couple has worked out together to minimize emotional damage. For me, silence isn’t about avoiding communication; it’s about recognizing that certain conversations are inherently futile. Rather than ending up mutually wounded, it is better to take a step back and use silence to preserve the peace of our home. This is my way of planning for our future and nurturing our relationship. Ultimately, silence in a cross-cultural marriage is a manifestation of the clash between two cultures. My silence embodies Chinese family values and philosophies on life, as well as my upbringing and cultural DNA. His lack of understanding reflects his own cultural background. There is no right or wrong, just difference. I know other binational couples where the woman is foreign and the man is Chinese, and in those cases, it is often the man who goes silent while the woman presses him to speak. So, it really isn’t a personal failing; it stems from different cultural foundations. Looking back over the years, I feel that whether it involves silence or arguments, as long as both partners cherish the home and genuinely want to build a life together, they will eventually find a way of relating that feels comfortable for both. Our life now may have its regrets and barriers, but it also holds a grounded, steady happiness. And for me, that is enough.

**I-02: (Female, Australian partner, without children, married < 3 years)**

Researcher: Alright, I have a general idea of the situation now. Next, I’d like to ask: looking back at your journey from dating to marriage, what do you think were the most significant impacts of your cross-cultural backgrounds, such as differences in values, mindsets, and communication styles, on your relationship?

Interviewee: There are so many—differences pop up in every aspect; it’s hard to sum it up with just one or two examples. The most obvious difference is definitely in communication styles. He’s the type of person who is incredibly direct; his emotions are written all over his face, and he speaks his mind immediately without ever beating around the bush. For instance, if he thinks a dish I cooked is too salty, he’ll just say, “The soup is a bit salty today; use less salt next time.” If he wants a specific gift, he’ll give me a list rather than making me guess. I’m completely different; I tend to hold back a bit of what I want to say. I don’t always voice my emotions directly because I feel that spelling everything out takes the magic away. If someone truly cares about you, they should just understand. When I’m upset, I won’t say, “I’m angry; come comfort me.” Instead, I might talk less, use a flat tone, or just sit aside scrolling through my phone, waiting for him to notice. But he rarely picks up on it; he only realizes something is wrong after my mood has become glaringly obvious, asking belatedly, “Are you upset?” Then there are the clear differences in values—spending habits, for example. He has a typical Western approach to spending: he spends what he earns and saves almost nothing. He maxes out his credit cards every month and pays them off when his paycheck arrives; he buys whatever he likes without thinking about the future. I can’t do that. I was raised with the mindset that “having reserves brings peace of mind.” I need savings to feel secure. I set aside a fixed amount every month, compare prices before buying anything, and absolutely refuse to spend money on things I deem unnecessary. We argued about this a lot when we first got together; he thought I was too stingy and made life too stressful, while I thought he was reckless with money and irresponsible. There are also differences in our views on family. He places a huge emphasis on boundaries. In his view, marriage is a matter between the two of us. It doesn’t really involve our parents, and each of us handles our own family affairs. I, on the other hand, feel that marriage involves both families; I believe the elders should interact, and it’s only natural for the families to help each other out when issues arise. Earlier, when my parents wanted to visit me in Australia, I wanted them to stay at our place, but my husband felt it would be inconvenient and suggested a hotel would be more comfortable. I was furious at the time; I felt he was being too distant, treating my family like strangers rather than family.

Researcher: Could you give a specific example of a small incident that made you strongly feel, “Wow, our cultural differences are really significant”?

Interviewee: One incident stands out vividly. Not long after we started dating, he ran a fever, over 38 degrees, and was completely drained. I was really worried; I brewed ginger soup, sponged him down with warm water to bring his temperature down, and even wanted to take time off work to look after him. But instead, he told me to leave him be, saying he just needed to lie down for a bit. He insisted a cold was a minor issue and didn’t require anyone else’s care. I felt really hurt and aggrieved; I was acting out of genuine concern, yet he seemed so unappreciative.

I only learned later that people here are just like that. They prefer to be alone when sick, feel that being cared for is an imposition on others, and dislike excessive fussing. It’s not just with partners; it’s the same with their parents. Back home, rushing around to care for a sick family member is the most basic form of concern, right? But to him, it felt like an intrusion. There was another time we were dining with his friends, and one of them bluntly said—right in front of everyone—that his new haircut was hideous and didn’t suit him. I was stunned; I thought, “How could you say that to someone’s face? It’s so rude.” But he just laughed along and said, “Yeah, I think they messed it up too,” treating it as no big deal. He later told me that friends should be honest and direct, and that beating around the bush is insincere. That was when I truly realized that the definition of “respect” differs between the two cultures.

Researcher: Did you find it hard to accept these differences when they first came up? Or did things get better as you gradually adapted to each other?

Interviewee: It was definitely hard to accept at first; I’d even start questioning whether he loved me. For instance, if he couldn’t guess why I was upset, I’d think, “If he really cared about me, how could he not understand what I’m thinking?” But after spending more time together, I gradually understood: it wasn’t that he didn’t love me. He simply didn’t have that particular way of thinking. He grew up in an environment where people spoke their minds directly; no one ever taught him to guess what others were thinking. We’ve found a much better rhythm now and have reached compromises on many issues, like finances. We opened a joint account for household expenses and manage the rest of our salaries independently, so we rarely argue about money anymore.

Researcher: Right. Let’s move on to the topic of silence. Do you ever go silent during your daily interactions with your partner or when a conflict arises? Could you describe one or two specific instances of silence that left a strong impression on you, such as what happened, the atmosphere at the time, and how long the silence lasted? What was going through your mind when you chose to remain silent?

Interviewee: It definitely happens, and quite frequently, too; it’s not just an occasional thing. The instance that stands out most was last winter. Well, around July or August, which is winter in Australia. It was my dad’s birthday, so I transferred 2,000 Australian dollars from our joint account to my parents as a gift. It wasn’t a huge amount, just a gesture of affection, and I thought it was perfectly normal, so I didn’t mention it to him beforehand. Later, when he was checking the account transactions, he saw it. After dinner, he brought his tablet over and asked me what the transaction was for. His tone was actually quite calm; he didn’t yell at me. He just said, very seriously, that any expenditure from the joint account—regardless of the amount—should be discussed with the other person first as a sign of mutual respect. As soon as he said that, I was struck speechless. I put down the bowl I was holding, walked into the living room, sat on the sofa, and just sat there with my head down, picking at my phone case without saying a word. I sat there for about twenty minutes. The TV was on—showing the rugby match he usually watches, but neither of us was actually watching it. The living room was incredibly quiet, save for the sound of the TV. At first, he sat next to me and explained that he wasn’t opposed to me giving money to my family; he just felt I should have let him know beforehand. When he saw I wasn’t saying anything, he stopped talking, sighed, and left me alone in the living room.

Researcher: What exactly were you thinking during those twenty minutes? Did you feel aggrieved, did you feel you were in the wrong, or was it something else?

Interviewee: My thoughts were a jumble; a lot of emotions came flooding in all at once. At first, I felt aggrieved. I thought sending money to my parents for their birthdays was only natural. It wasn’t as if I was squandering it, so why did I have to specifically report it? Two thousand Australian dollars isn’t a huge sum; was it really necessary to make such a big deal out of the accounting? At the time, I even felt a bit disheartened, thinking he was drawing too sharp a line between us and didn’t truly regard my parents as family. But on second thought, what he said made sense. The money in the joint account belonged to both of us, so giving a heads-up was indeed the right thing to do; I simply hadn’t considered his feelings. Yet, I couldn’t bring myself to admit I was wrong, nor did I know quite how to say it. Admitting fault would make me look like I was clearly in the wrong, but arguing might just escalate the conflict, turning a minor issue into a major fight.

There was also the issue of language: there were many things I could explain clearly in Chinese but didn’t know how to express in English. The words would be on the tip of my tongue, but I couldn’t organize them properly; I feared that saying them aloud might distort the meaning and lead to even greater misunderstanding. The more I thought about it, the less I knew how to broach the subject, so I’d simply stay silent, deciding to wait until everyone had calmed down. There was another instance, not an argument, just a small, everyday occurrence, that left a deep impression on me. Living in Australia, I really struggle with the local accent, so I always feel anxious running errands alone. But I don’t like to keep asking him for help verbally. Instead, whenever I have to go somewhere, I’ll just quietly sit next to him until he offers to come along. This kind of restrained reliance fits perfectly with Chinese implicit communication without making him feel burdened.

Researcher: Could you describe that scenario in more detail? For instance, what kind of errands were you running? How long did you sit there? Was he able to understand what you meant each time?

Interviewee: Take things like submitting documents to the immigration office or opening a bank account. There’s a lot of technical jargon involved, and the locals speak fast with heavy accents, so I’d often catch only half of what was said. Once, I had to go downtown for some business; I started feeling anxious the night before, constantly looking up vocabulary because I was afraid I wouldn’t understand the staff’s questions or would fill out the forms incorrectly.

The next day. It was the weekend. He was reading the newspaper in the living room. I gathered all my documents, put on my coat, and sat in the armchair next to him without saying a word. I sat there for maybe ten minutes before he finally put down the paper, looked up at me, and asked, “Are you going out today? Do you want me to drive you and help with the translation?” I just nodded; I didn’t tell him I wanted him to come along because I couldn’t understand the language. I just said, “Sure, that would be a big help.” But it didn’t always go that smoothly. Sometimes he’d be so focused on a game or a match that he wouldn’t even notice me sitting there. If I sat there for a long time without any reaction from him, I’d get a bit angry, feeling like he didn’t care about me at all. Yet, I still wouldn’t say anything; I’d just get up, go back to my room, and decide not to go out at all out of spite. He’d be left baffled, wondering why I’d suddenly gotten upset.

Researcher: So why did you prefer to sit and wait for him to offer, rather than just asking directly? Is it hard for you to ask for help?

Interviewee: I can’t quite put my finger on it. It’s just that I felt awkward. I didn’t want to keep bothering him; he wanted to rest on weekends and had his own things to do. Asking him felt like assigning him a task or being a nuisance. There was also a sense of insecurity—feeling useless because I couldn’t handle even a small matter like that on my own. Maybe it’s because my English isn’t great yet, and I’m not fully familiar with the environment in Australia. Plus, I’ve always felt that attention you have to ask for is different from attention given voluntarily. If he offered to come with me, it showed he cared about me and was doing it willingly. If I asked him to go, it felt like something he was forced to do, something he’d resent doing. Isn’t that something we Chinese people value? An unspoken understanding is more precious than words; once it’s put into words, the magic is lost.

Interviewer: Right, I see. Next, I’d like to ask: in which specific situations are you most inclined to remain silent? For instance, when facing accusations from your partner, discussing sensitive topics, dealing with a clash of cultural values, feeling exhausted, or trying to express feelings of grievance or anger?

Interviewee: It happens in all those situations, I suppose. The first instance is what I mentioned earlier—when we have a disagreement or he points out a fault of mine; that’s when I’m most likely to go silent. It’s not that he’s yelling at me; even when he calmly reasons with me and explains what I did wrong, I instantly lose the desire to speak. For example, if I forget my keys when going out or burn the food while cooking, and he says, “Be more careful next time,” even though his tone is perfectly calm. I feel a sense of unease or defensiveness, so I clam up and stay quiet for a long time. Then there are sensitive topics, like whether to return to China to settle down, whether our child should receive a Chinese or Western education, or issues regarding immigration status. I tend to go silent whenever these come up. He definitely wants to stay in Australia for good, since his family, friends, and job are all here. But I haven’t fully made up my mind; my parents are getting older, and it’s impossible for me never to go back. However, discussing this easily leads to disagreement, neither of us can convince the other, so whenever he brings it up, I just give a perfunctory response or say nothing at all and change the subject. Regarding cultural values, say, when we discuss social customs or family ethics in China versus Australia. If he expresses a view I don’t agree with, I often feel it’s pointless to argue. There’s no clear right or wrong in these matters; they’re just ideas shaped by our respective upbringings. Arguing about them just leads to a fight, so I stay silent, don’t engage, and let the topic pass. Also, when I’m with his family or at a gathering and sensitive topics or local news come up, I don’t really join in. I prefer to maintain a harmonious atmosphere; it’s not as if generational differences can be resolved or fully explained in an instant. Sometimes, when they make jokes, I just sit there smiling and nodding occasionally, barely saying a word the whole time. And of course or when I’m in a bad mood or physically exhausted, I simply don’t feel like talking. For instance, sometimes I come home exhausted after a long day of work and just don’t want to talk; I just want to sit quietly for a while. It’s not directed at him. I simply lack the energy to speak. Even when he talks to me, I just respond with “mm-hmm” or “uh-huh” rather than initiating any conversation myself.

Researcher: In which of these situations do you find yourself remaining silent the most?

Interviewee: It’s probably when I feel pressured to express myself verbally. Often, it’s not that I don’t want to speak, but that I genuinely can’t. I might have a lot on my mind, but I can’t find the right words in English. The more anxious I get, the harder it is to speak, so I eventually just give up. This is especially true during arguments; when emotions run high, my mind goes blank. I struggle to articulate even simple sentences, so silence becomes my only option.

Researcher: So, would you say the primary reason you choose silence is the pressure of expressing yourself in the language?

Interviewee: There are actually many reasons; rarely is silence caused by just one thing. Usually, it’s a combination of factors that makes me lose the desire to speak. That said, the most immediate cause is definitely the pressure of expression. After all, English isn’t my native language. Casual, everyday chat is fine, but when it comes to nuanced matters, like emotions or personal viewpoints, I can’t explain myself clearly. For example, feelings like grievance, heartache, or a sense of helplessness can be described with great precision in Chinese, but in English, I simply can’t convey that complexity. If I keep it superficial, he doesn’t grasp the full picture; if I try to go deeper, I lack the words to do so. Over time, I just stopped trying. Then there’s the fact that I want to avoid arguments. I’ve always felt that fighting damages the relationship between a couple—that harmony is far more important than anything else. Many issues aren’t matters of principle; arguing over who is right or wrong is pointless. Taking a step back usually resolves things. So, whenever I sense an argument brewing, I just clam up, thinking the moment will pass. I’ve always viewed arguing as something that disrupts family harmony, something negative. There’s also the feeling that things just can’t be explained clearly. Many cultural aspects can’t be summed up in a few words, like why I dislike directly refusing people or why I care so much about “saving face.” These traits were shaped over twenty-plus years of my life; even if I explained them, he might not understand and could simply think I’m making excuses. It’s like describing snow to someone who has never seen it; no matter how much you say, they still won’t have a real concept of it. So, often I just don’t bother explaining; silence is simply less of a hassle. It’s also a matter of “face”—both mine and his. For instance, if he says something incorrect in front of others, I won’t contradict him on the spot; I’ll spare his feelings and let him save face. Conversely, if I make a mistake and he points it out, I feel embarrassed and lose face; I become too self-conscious to speak and simply fall silent. There are also times when I stay quiet because I’m afraid my English isn’t good enough. I worry that making a mistake would be embarrassing or make me look foolish, so I figure the less I say, the less likely I am to mess up.

Researcher: Among these reasons, which one do you think is the most fundamental?

Interviewee: When I first arrived in Australia, I thought language was the key factor. I used to believe that once I improved my English and could express myself fluently, I wouldn’t be silent anymore and our communication would improve. But I’ve been here nearly two years now; my English is vastly better than when I started. I can handle daily tasks and casual conversations with ease, yet I still fall silent. I eventually realized that the core issue is actually cultural mindset—deep-seated communication habits. Even if language barriers vanish completely, I’m still not used to speaking my mind directly; I’m always concerned about the other person’s feelings, maintaining harmony, and avoiding conflict. Language is merely a tool; what truly dictates how we speak is the culture and education we grew up with. Take Chinese people who have lived abroad for over a decade, for example, they might speak English just as well as the locals, yet their personalities remain reserved. They still aren’t accustomed to expressing emotions openly and often default to silence. That’s the influence of culture, something language alone cannot change.

Researcher: Have there been times when you knew you shouldn’t stay silent, that you ought to speak up, but found yourself unable to do so?

Interviewee: Oh, so many times. I almost always regret it afterward. For instance, there was a time he promised to go to IKEA with me on the weekend but then made last-minute plans to play sports with friends and apologized. I was furious inside, but the words just wouldn’t come out. In the end, I simply said, “It’s fine, go ahead,” and then went silent. He actually believed I was fine and left happily. After he left, I sat at home stewing in frustration; the more I thought about it, the more aggrieved I felt, wondering why I couldn’t just speak my mind. Yet, the next time a similar situation arises, I do the exact same thing. I can’t seem to change it. It’s as if my mouth has a mind of its own; when that moment comes, I just can’t bring myself to speak. Researcher: So, what impact does your silence have on your communication or relationship? For instance, does it help maintain harmony, defuse conflict, escalate tensions, leave your partner confused or hurt, provide time to cool off, or create lingering resentment? Could you give some examples?

Interviewee: It certainly helps defuse conflict and gives us both time to cool down. Sometimes, when emotions run high, things get heated, and it’s easy to say hurtful things in the heat of the moment. Communicating after calming down is much more level-headed; we avoid a full-blown argument. If I were to argue with him directly, things would likely escalate—nasty words would be exchanged, and we’d both end up hurt. It also helps maintain a sense of outward harmony, allowing many minor issues to just blow over. For example, if he leaves his socks lying around or doesn’t wash the dishes after a meal, I might be a bit annoyed but choose not to say anything; I’ll just stay silent and clean up myself. It’s not worth starting a fight over such trifles. This keeps the atmosphere at home peaceful. We don’t fight every day. That said, there are times when he feels confused or hurt. He often doesn’t know why I’ve suddenly gone quiet, whether I’m angry, tired, or just unhappy. He’s told me that whenever I go silent, he feels incredibly frustrated; he assumes he’s done something wrong but doesn’t know what, and the guesswork is exhausting. Many issues seem to pass because of that silence, but in reality, they aren’t resolved. they’re just temporarily suppressed. They resurface the next time a similar situation arises. Take the issue of moving back to our home country to settle down, for instance: whenever we discuss it, I tend to go silent halfway through, and the conversation just fizzles out. Yet, the issue remains, neither of us has convinced the other, and it’s something we’ll inevitably have to face eventually. It’s like a time bomb; it might not go off now, but it could explode with even greater force later on.

Researcher: Does your partner ever misunderstand your silence? How does he usually react to it? For example, does he ask what’s wrong, respond with silence of his own, show impatience, or try to draw you out? What is your take on these reactions?

Interviewee: Yes, there are misunderstandings. His most common misconception is assuming that my silence means I’m angry or giving him the “silent treatment.” In reality, sometimes I’m just tired, deep in thought, or simply don’t feel like talking. I’m not angry at all. But he interprets it as me throwing a tantrum or trying to punish him. His reactions evolved over time. When we first started dating, he was incredibly patient; whenever I fell silent, he would lean in and ask, “What’s wrong?” “Are you upset?” or “Can you tell me about it?” He would gently coax me to speak until I finally opened up. Back then, I found it quite sweet. It showed me how much he cared. But after we got married and had been together for a while, his patience wore thin. At first, he might still ask a couple of questions, but if I remained silent, he’d get a bit impatient and say, “You’re always like this. Can’t you just say what’s on your mind?” Then he’d get up and walk away to watch a game or play video games, leaving me all alone. Other times, if my silence dragged on, he would go silent too, and we’d end up in a standoff. His attitude was basically: “If you don’t want to talk, neither do I—let’s see who cracks first.” Usually, though, he was the one who couldn’t hold out and would be the first to strike up a conversation.

Researcher: So, how do you view these reactions of his? Do you feel like he doesn’t understand you, or do you think he makes a valid point?

Interviewee: I understand him, and I know he’s right. From his perspective, if your partner suddenly stops talking without explaining why, anyone would feel anxious and upset. He grew up in an environment where people express themselves directly, so he naturally finds this kind of guessing game intolerable. It really isn’t fair to make him constantly guess what I’m thinking. But I feel aggrieved, too. It’s not that I’m intentionally giving him the silent treatment or trying to give him attitude; it’s just the way I’m used to communicating. I’ve always felt that truly close people should share an unspoken understanding—that you shouldn’t have to spell everything out. Back home, that’s how things were with my best friend and my parents; we could understand each other with just a glance. But that approach doesn’t work with him. Still, I’ve gradually come to realize that I can’t hold him to my own standards. He’s a foreigner who hasn’t lived in Chinese culture, so he can’t be expected to intuitively understand my thoughts. Since we’re building a life together, we have to accommodate each other, so I need to try and change a bit, too.

Researcher: I see. So, after a period of silence, how do you usually “break the ice” and resume communicating? Does one of you take the initiative to speak, use physical contact, or does the conversation just naturally flow back through daily routines?

Interviewee: The vast majority of the time, he’s the one who breaks the ice; I rarely take the initiative to speak first. He has quite a few ways to break the ice, but the most common is striking up a conversation based on something trivial, using a small, everyday matter to get the ball rolling. Usually, when he asks something like that, I take the opening he’s offered; I’ll give him an answer, and then we gradually return to normal conversation. If it’s just a minor tiff, he’ll come sit next to me, give my arm a nudge, or gently hug me from behind. No words are needed—just a hug for a moment, and we’re back on good terms. That approach works best and is the gentlest; that’s usually how we handle small squabbles. If I’m clearly in the wrong, or if I’ve stayed silent for so long that I feel awkward, I’ll be the one to speak up first. When I initiate, I usually don’t apologize outright; instead, I show goodwill through my actions. He realizes I’m no longer angry and starts talking to me again.

Researcher: After breaking the ice, do you ever revisit the earlier conflict? Or do you just move on and never mention it again?

Interviewee: For small things, we just move on and never bring it up again. But if it’s a major conflict, something involving fundamental beliefs or principles, he’ll wait for a relaxed moment to talk it over after we’ve made up. For instance, while we’re watching TV or having a meal in the evening, he’ll gently bring it up, share his perspective, and ask for mine. He’s usually the one to initiate that kind of review; I rarely do. I tend to feel that once something is over, it’s over, bringing it up again just risks unpleasantness. But he believes that talking things through prevents lingering resentment and stops us from fighting over the same issue next time. I’ve gradually come to agree with him; talking it out actually makes things feel lighter.

Researcher: I see. Do you ever explain to your partner why you went silent in the moment? For example, telling him, “I wasn’t speaking because I needed to cool off, not because I was using the silent treatment.” Does that kind of explanation help reduce misunderstandings?

Interviewee: I didn’t used to. I never explained myself back then. I felt that once an issue had passed, there was no need to explain—those who understand you just do, and for those who don’t, explanations are useless. Plus, I felt that volunteering an explanation made it look like I was backing down, which felt a bit embarrassing. Now, though, when we have arguments or conflicts, I do start to explain.

Researcher: Do you find that kind of explanation helpful? How does he react when he hears it?

Interviewee: It is helpful. Each time I finished explaining, he would visibly relax; he said that knowing the reason for my silence, and realizing it wasn’t directed at him, put his mind at ease and stopped his imagination from running wild. Yet, while this could reduce misunderstandings, it couldn’t change his fundamental view of silence itself. After every explanation, he would ask, “Then why couldn’t you just tell me right then? Why did you have to wait until later?” He still couldn’t grasp why I couldn’t simply voice my feelings on the spot. To him, speaking one’s mind immediately is the most basic form of communication. Thus, while explanations prevented him from misinterpreting me, they failed to resolve the underlying issue.

The issue really comes down to our different communication habits.

Researcher: So, do you explain yourself after every instance of silence now? Or only when the situation is serious?

Interviewee: I only explain when it’s serious, and when it affects my emotions. There’s no need for small things; it usually blows over after a while. Besides, often by the time the silence ends and the moment has passed, I’ve forgotten to explain. There are also reasons I feel too embarrassed to share with him—things I just can’t bring myself to say. I don’t want him to think I’m fragile or useless, so I’d rather let him misunderstand and think I’m angry than reveal the real reason.

Researcher: I see. Let’s talk about this from a cultural perspective. Do you think certain concepts in Chinese culture, such as “harmony in the family leads to prosperity in all affairs,” “think thrice before acting,” “silence is golden,” “careless talk leads to trouble,” or “being prudent in speech and action,” or perhaps your upbringing, influence your choice to remain silent in an intercultural relationship?

Interviewee: A lot of that influence is subtle; I hadn’t even realized it myself before. But it definitely plays a role. Even though my parents argued a lot, whenever my grandparents intervened, they’d say that for a family living together, peace and harmony matter most—constant bickering damages the relationship. So, in my own marriage, I subconsciously strive for “harmony.” When a disagreement arises, my first instinct isn’t to argue about who’s right or wrong, but to figure out how to avoid a fight and keep things harmonious. Silence is the easiest way to handle it—no arguing, no shouting; just endure it for a bit, and it passes. After all, Confucian thought values prudence in speech. “Disaster comes from the mouth.” Even though I’m at home with my partner, that habit is ingrained in me. When I’m unsure about something or feeling emotional, my first reaction is to shut my mouth and think before I speak. I’m afraid of saying the wrong thing and hurting someone, or looking foolish. My parents were quite reserved people, too; they rarely exchanged sweet nothings. If they had a conflict, they’d argue or give each other the silent treatment for a few days, but then they’d go back to cooking and talking as usual, and things would naturally smooth over. Growing up in that environment, I came to believe that’s just how married couples are. You don’t need to spell everything out, and silence is a perfectly normal way of interacting. I’m quite introverted by nature; I don’t talk much, and I’m used to shouldering burdens on my own rather than confiding in others. I’ve never really had heart-to-heart conversations with my parents, either.

Researcher: So, do you feel that while these attitudes might be considered normal, or even virtues. In a domestic marriage, they actually become communication barriers in a cross-cultural marriage?

Interviewee: I think that even back home, if you were married to a Chinese man, he might interpret prolonged silence as a form of silent treatment. But in a cross-cultural marriage, the issue of cultural difference is a major factor; if that’s understood, I think it’s manageable.

Researcher: Right. Do you feel there’s been a change in how often or in what way you use silence within your cross-cultural marriage? How does the present compare to when you first started out together?

Interviewee: When we first started dating—during the honeymoon phase. I wanted to share everything with him. If a problem arose, I’d try hard to explain it in English; even if I was stumbling over my words, I’d still give it a go. Plus, being back home meant I was in a familiar environment. I had my own job and friends, and I felt confident. I didn’t feel inferior because of language or culture, so I didn’t resort to silence very often. The first six months after arriving in Australia were when I was silent the most. My language skills were poor back then; even going out to shop was a struggle. I had no friends and had to rely on him for everything, which made me feel incredibly insecure. I’d go silent when running errands, silent during arguments at home, and silent throughout gatherings with his friends, barely saying a few words a day. That kind of silence was mostly due to the pressure of the language barrier and a lack of confidence. Nowadays, the frequency of my silence has dropped a bit compared to when I first arrived. After all, my English has improved, I’ve adapted to life here, and I have my own part-time job and social circle. I’m much more confident and willing to speak up about things. However, the reasons for my silence have changed; now, it’s usually due to a clash of cultural values. I realize that some issues won’t be resolved by arguing—that talking about them is pointless, so I simply choose not to say anything. In the past, my silence meant suddenly clamming up and saying nothing, which would instantly make the atmosphere awkward. Now, I’ll tell him beforehand, “I’m feeling a bit worked up and need to cool off for a while; let’s talk later.” Then I’ll go to my room for a bit and only come out to talk once I’ve calmed down. Even though I still go silent, at least I give him a heads-up so he doesn’t let his imagination run wild. I suppose that counts as progress. Researcher: Do you feel this change was something you initiated yourself? Or did it come about through the process of the two of you adapting to each other?

Interviewee: It’s a bit of both. I gradually realized that staying silent wasn’t a solution. In fact, it could hurt the relationship, so I took the initiative to change. It was also partly driven by him; he’d shared his feelings with me many times, and I didn’t want him to keep feeling distressed, so I tried adjusting my communication style. Of course, I didn’t change everything, just made slight tweaks to make things easier for him to accept. You can’t change who you are at your core; I couldn’t just turn into someone who speaks their mind bluntly like he does—that wouldn’t be me anymore.

Researcher: So, do you think you’ll use silence less often in the future? Or will silence always be a part of your relationship?

Interviewee: I’ll try to handle silence more gently in the future, like giving a heads-up beforehand or explaining things afterward, so it doesn’t become a weapon that hurts the other person. As long as he understands, silence can just be a way of coexisting; intimacy doesn’t necessarily require constant talking.

Researcher: Alright, one last question. Regarding your use of silence within this cross-cultural family, is there anything we haven’t asked about that you feel is important and would like to add?

Interviewee: Often, silence isn’t a sign of indifference; it’s actually because I care so much about the relationship. I’m afraid of saying the wrong thing and hurting him, afraid that arguing will damage our bond, or that cultural differences might lead to an irreconcilable conflict. That’s why I tread carefully and choose silence to maintain harmony. It’s not that I don’t care; it’s precisely because I care so much that I’m so cautious. Also, silence isn’t just a communication issue; underlying it are questions of identity. As an outsider in an unfamiliar environment like Australia, where my language skills and cultural familiarity don’t match the locals’, silence serves as a form of self-protection. Speaking less means making fewer mistakes, which helps avoid awkwardness or being looked down upon. After living in that state for so long, I bring that habit home with me and fall into silence unconsciously. It’s not directed at my partner; it’s just that I haven’t fully integrated into this environment yet. Cross-cultural marriages require a lot of adjustment. It’s not about one person accommodating the other, but about both people moving toward each other. I’m learning to express myself directly and speak my mind, while he’s learning to understand my reticence and pick up on my emotions. Take the present, for instance: he is gradually learning to understand my silences and no longer constantly presses me for answers. A healthy intimate relationship is one that embraces both animated conversation and quiet silence. As long as two people hold each other in their hearts and are willing to understand one another, both speaking and silence serve as ways to nurture the relationship. There is no single “right” way; whatever works best for the two people involved is the best approach.

**I-03: (Female, US partner, with children, married 4–10 years)**

Researcher: Alright, let’s start with your overall impressions. Looking back at your time together, what do you think were the most significant impacts of your differing cultural backgrounds, such as values, mindsets, and communication styles, on your intimate relationship?

Interviewee: The most striking difference was definitely in communication styles. He has that typical American directness; when a problem arises, he has to address it immediately. He wears his emotions on his sleeve and voices his dissatisfaction right then and there. He simply can’t bottle it up. I, on the other hand, am more inclined to observe and weigh things up first, running the situation through my mind several times before deciding whether or not to speak, and how to phrase it. Often, my silence didn’t mean I was angry; I just needed time to organize my thoughts. But he would always interpret it as giving him the cold shoulder or acting distant, which led to a lot of misunderstandings early in our marriage. Then there were the differences in emotional expression. Americans are so open about their feelings—everything has to be verbalized. Phrases like “I love you,” “You look great today,” or “I’m so grateful for you” are constantly on their lips; they might say them several times a day. I found it really hard to adjust to that at first. It felt cringey. I’ve always believed that sincerity is shown through actions, not words. To me, a lot of caring gestures didn’t need to be spoken aloud. But he saw it differently; he’d ask, “If you don’t say it, how am I supposed to know what you’re thinking?” It took a long time for us to find a balance. Now, I’ll say those things occasionally, and he’s gradually learned to understand the meaning behind my actions. Our mindsets are truly different, too. He’s a linear thinker. He focuses on the specific issue at hand and treats each matter separately. I’m a holistic thinker; I like to consider the broader context and all the surrounding factors. For instance, when our child had just learned to walk and took a tumble, his first reaction was to rush over, pick the child up, and say things like, “Oh, poor baby, Daddy feels so bad for you”—prioritizing emotional empathy. My first reaction was to crouch down and check for scrapes or see if disinfection was needed—prioritizing practical problem-solving. He actually criticized me back then, asking why I was so calm and whether I cared about the child at all. I was speechless; does caring really have to be shouted out loud? It took time for me to explain that we simply express care in different ways.

Researcher: Did those differences in expression often lead to minor misunderstandings early on? Can you give any specific examples?

Interviewee: Oh, there were so many. For instance, about six months after we got married, he celebrated my birthday for the first time. He gave me a huge teddy bear and a bouquet of roses, and immediately stared at me, asking, “Do you like it? Are you happy?” I was actually quite happy, but I felt shy about expressing it too openly, so I just smiled, nodded, and said, “It’s pretty good—thanks.” He ended up feeling a bit down that evening. Later, he told me he thought my reaction was too lukewarm; he wondered if I didn’t like the gift or if he’d made a bad choice. I never imagined that a simple “It’s pretty good” would lead to so much overthinking on his part. In our cultural context, saying something is “pretty good” actually implies a high level of satisfaction. We’re just reluctant to sound overly effusive, but he interpreted it literally as “just average” or “unsatisfactory.” That’s when I realized that many of our default ways of expressing ourselves simply don’t work in a cross-cultural context.

Researcher: Could you describe one or two specific instances of silence that left a deep impression on you—perhaps during your daily interactions or when a conflict arose? For example, what was happening? What was the atmosphere like? How long did the silence last? And what was going through your mind when you chose to remain silent?

Interviewee: There was an incident regarding the division of household chores. He had been working overtime all week, and we had planned to take our child to the local park on Saturday. However, when he got up that morning and saw the previous night’s dishes piled in the kitchen sink and breadcrumbs scattered on the dining table, he immediately snapped. He spoke very harshly: “You’ve been home all day—couldn’t you have tidied this up? Look at the state of the house.” I had just managed to get the child to sleep. The night before, the child had an upset stomach and woke up three times, so I’d barely slept all night; I had just sat down to catch my breath and have a drink of water. Hearing him say that made me feel choked up with a sudden sense of grievance. The words were right on the tip of my tongue. I wanted to argue back: “Can’t you see I didn’t sleep a wink? Do you think looking after a child at home is easy?” But then I looked at his exhausted face and unshaven stubble, and I swallowed the words. I told myself that he was under a lot of pressure, too. We were both angry, and an argument would only escalate; plus, the child was still sleeping in the bedroom, and waking them up would just make things more complicated. I didn’t say anything then; I just stood up, walked to the kitchen, and turned on the tap to wash the dishes, deliberately moving slowly amidst the sound of rushing water. We stayed silent for about ten minutes. There weren’t actually many dishes, but I wanted to calm myself down and give him a moment to cool off, too. By the time I was washing the second dish, he walked over and leaned against the kitchen doorway. His tone had softened as he said, “I’m sorry, I was too harsh earlier. There was a problem with a project and I was frustrated; I didn’t mean to snap at you.” Tears were welling up in my eyes, but I didn’t speak right away; I dried my hands and, after a few seconds, said, “It’s okay, I know you’re under a lot of pressure.” Later, we tidied up the living room together and still took the kids to the park. That experience made me realize that silence can actually act as a buffer to cool down emotions. There’s no need to go head-to-head in the heat of the moment.

Researcher: During those ten minutes, aside from feeling aggrieved and waiting for him to calm down, were there any other thoughts going through your mind? For instance, was there a moment when you felt, “He doesn’t understand me at all”?

Interviewee: Oh, definitely. At first, I felt really resentful—thinking he didn’t see everything I did and wondering why he had the right to criticize me the moment he got home. But listening to his voice, I also realized he was exhausted; it’s not easy for either of us. Plus, I know his temper. It flares up fast but fades just as quickly. Once that initial surge passed, he’d realize he was in the wrong on his own. If I had argued back right then, he would have just gotten more agitated. It would have turned a small issue into a huge, messy conflict. It just wasn’t worth it.

Researcher: I see. Is there another scene that stands out in your memory?

Interviewee: There’s another one that took place in a restaurant on our wedding anniversary. We went to a well-known Italian place near our home and happened to run into some of his colleagues, so we ended up sharing a table. While we were chatting over the meal, someone asked if I’d had any embarrassing moments when I first arrived in the U.S. I told them about the time I went grocery shopping shortly after moving here; I couldn’t quite read the English labels and mistook fine salt for granulated sugar. I used it to bake a cake, but it turned out so salty it was inedible. I had to throw the whole thing away. I was right in the middle of a sentence when my husband suddenly interrupted me, excitedly telling his colleagues, “Oh, by the way, we went hiking in the park last week. The scenery on that trail was absolutely stunning—you guys really have to go.” He then launched into a conversation about hiking with them, leaving me completely sidelined. Since I’d been cut off mid-sentence with several people at the table looking right at me, I felt incredibly awkward; my face burned, and I simply couldn’t bring myself to finish what I was saying. I just picked up my glass for a sip of water and looked down to cut my steak, saying nothing more. There was a silence of maybe thirty or forty seconds. I’d just lost all desire to speak and felt pretty embarrassed. After chatting for a bit, he suddenly realized something was up; he leaned in and whispered, “Are you upset?” I shook my head and smiled, saying, “No, not at all—you guys keep talking,” though deep down, I was actually quite bothered. Later, on the way home, he asked me again, and I finally told him: “You interrupted me before I could finish speaking, and it was really awkward for me in front of your colleagues.” It finally dawned on him; he explained that he’d gotten so excited talking about hiking that he hadn’t noticed, and he apologized. That silence was different from the time regarding housework—that earlier instance was a deliberate, conscious choice on my part to cool things down. This time, the silence was more instinctive—a mix of sulking and a desire to avoid a public outburst. I used silence to compose myself so I wouldn’t say something harsh and make things awkward for everyone.

Researcher: It sounds like the underlying reasons for the two types of silence were indeed quite different. So, in what specific situations are you more inclined to stay silent? For instance, when facing accusations from your partner, discussing sensitive topics or cultural clashes, feeling exhausted, or dealing with emotions like a sense of grievance or anger?

Interviewee: There are quite a few scenarios. The most obvious ones involve sensitive topics or clashes in cultural values, especially religion; that is truly the biggest minefield between us. I’m an atheist, but my American husband is a devout Catholic. He goes to church every week and wants our child to be baptized. I wasn’t really on board, so he thinks I disrespect his faith. Since then, whenever the topic comes up, I just keep my mouth shut to avoid arguments. The year our child turned two, he brought the matter up formally for the first time. He had just returned from church on a Sunday and was sitting on the sofa, holding the child, when he told me he had already made arrangements with the priest to have the child baptized the following month. I was folding laundry nearby; upon hearing this, I stopped mid-motion, my heart sinking. He had mentioned it casually a few times before, and I had always laughed it off or brushed it aside, but I hadn’t expected him to go ahead and make actual arrangements. I remained silent and continued folding—albeit very slowly—while my mind raced, trying to figure out how to respond without starting an argument. Seeing my silence, he grew annoyed and asked, “I’m trying to have a serious conversation here. What kind of attitude is that?” I still didn’t speak, knowing that doing so would inevitably lead to a fight. I genuinely felt that the child should choose his own faith when he was older; baptizing him now, when he understood nothing, seemed far too hasty. To him, however, this was about passing on a faith, a pivotal life event, and my disagreement implied a lack of respect for his beliefs and a refusal to integrate into his family. Our views on this were worlds apart; there was no common ground, and neither of us could convince the other. If I had voiced my true feelings, we would have argued all night—a bad influence with the child right there. So, I stayed silent. Eventually, he sighed, took the child to the living room to play, and the topic was dropped. Since then, whenever he brings up baptism or religion, I don’t say a word; I either get up to do something else or simply listen quietly without committing to a position.

Researcher: Aside from the sensitive core issue of religion, are there other situations where you tend to choose silence?

Interviewee: Yes, for instance, when he’s highly emotional. If he’s been treated unfairly at work or had a conflict with a colleague and comes home venting rapidly, I usually just sit and listen. I don’t interrupt or rush to offer advice; I just listen silently. I know he needs to vent, not find a solution; if I were to chime in, I might disrupt his emotional release and just make him more frustrated. Once he’s said his piece, he naturally calms down. Another instance involves matters concerning his family. For example, when his mother suggested flying over to stay with us for a month during Christmas. I didn’t really want to do it; the child was too young, a long-haul flight would be too exhausting, and I felt a bit awkward spending extended periods with his family. However, I couldn’t simply refuse an elder. It would make me look inconsiderate, so I would stay silent and let him handle the conversation with his mother. After all, she is his mother; it’s more appropriate for him to speak to her than for me to do so. I also tend to fall silent in public settings or in front of others. For instance, at company gatherings or community picnics, if someone asks a personal question, like “Do your parents in China miss you?” or “When are you planning to have a second child?”, and I don’t know how to answer or simply don’t want to share too much with strangers, I’ll just smile, pause, and let my husband take over the conversation. I’ve always felt that arguing in public or oversharing personal details is rather impolite.

Researcher: So, in which of these situations do you find yourself remaining silent most often? Is it regarding religious topics?

Interviewee: Yes, definitely religious topics. I go silent the moment the subject comes up. It’s practically a reflex. There’s no middle ground there; he holds firm to his faith, and I have my own bottom line regarding my beliefs—neither of us can back down. We argued about it once and realized it was pointless; arguing only hurts the relationship, so it’s better to just stay silent and move on to another topic. Everything else is open to discussion, but that one issue inevitably leads to conflict.

Researcher: Based on what you’ve said, what do you consider the primary reason for choosing silence? Is it to avoid arguments, a feeling that it’s impossible to explain, a desire to maintain harmony, a need to save face, the influence of cultural values, the pressure of expressing yourself in the language, or simply not knowing how to respond in the moment?

Interviewee: The core reason is definitely avoiding arguments and maintaining harmony in the relationship. Home is a place for love, not for debating who is right; winning an argument at the cost of the relationship isn’t worth it. Subconsciously, I feel that arguing is deeply damaging to a relationship. I avoid it whenever possible and choose to hold back. Silence serves to suppress the conflict and prevent the issue from escalating. This is especially true now that we have a child; I really don’t want to argue in front of him for fear of scaring him. And, of course, the influence of cultural values is deeply ingrained. Back in school, teachers always told us to “listen more and speak less”—that children shouldn’t interrupt when adults were talking. I gradually developed the habit of carefully weighing my words before speaking; I felt that the more I said, the more likely I was to make a mistake, so it was better to speak less and do more. That mindset is ingrained in me; even living in the U.S. now, I can’t seem to shake it. Then there’s the pressure of expressing myself in a foreign language, something that was particularly acute during the early years of my marriage. While I could handle daily conversations fine, my English fell short when it came to arguments or expressing complex emotions. For instance, I might be thinking, “You aren’t seeing things from my perspective at all; you only see your own hard work and overlook my contributions.” I’d be filled with a sense of grievance, yet when I tried to voice it in English, it came out flat, just a simple “You don’t understand me”, failing completely to convey the depth of my feelings. The more anxious I got, the worse I spoke, until eventually, I’d just give up. Silence seemed better than trying to explain and making a mess of things, which might only lead to deeper misunderstandings. I remember one time we argued about whether to buy an expensive car seat for our child. I wanted to say, “It’s not worth the price; there’s no need to spend that much when a standard model meets safety standards.” But I got stuck on the words; after struggling to explain for a while without him understanding, and him asking, “What exactly are you trying to say?”. I felt so frustrated that I just shut my mouth and walked away. In moments like that, silence felt easier than the struggle to explain. Then there’s the issue of “face.” Chinese people care a lot about saving face, both our own and the other person’s. Take a community picnic we attended recently, for example. Many neighbors were there, and while I was helping him grill burgers, I accidentally burned two of them. He immediately shouted, “What are you doing? The heat is too high—you need to watch what you’re doing!” Several people nearby turned to look. I blushed with embarrassment; I was actually quite upset that he’d criticized me publicly and hadn’t spared my feelings. Yet, I didn’t say a word; I just nodded and set the burnt burgers aside. If I had argued back right then and there, it would have been even more humiliating, having the neighbors watch us bicker would have been a real loss of face. So, when we’re around others, even if I’m upset, I hold it in and use silence to maintain a sense of dignity. Researcher: So, among these reasons, which one do you consider the most central? Has the ranking of these reasons changed from when you first got married to now?

Interviewee: When we first got married, the language barrier was definitely one of the top two issues. Often, it wasn’t that I didn’t want to speak, but rather that I couldn’t express myself clearly, so I’d just give up and stay silent. Now, after so many years of marriage, my English has improved significantly. I have no trouble expressing my emotions during a daily argument, so the core reasons have shifted to cultural habits and a desire to avoid conflict. Deep down, I feel there’s no need to fight to win every single point; taking a step back isn’t a big deal. Plus, I know his temperament. He forgets things right after an argument and doesn’t hold grudges, so yielding a little doesn’t hurt; it’s all for the good of the family anyway.

Researcher: How does your silence affect your communication or relationship? For instance, on the positive side, it might maintain harmony or give you both time to cool off; on the negative side, it could escalate conflicts, leave your partner confused or hurt, or create lingering resentment. Could you share some specific examples?

Interviewee: There are both positive and negative sides, and they’re quite distinct. The positive aspect is that it really helps avoid emotional outbursts and gives both parties space to calm down. Take the time we argued about housework, for example: if we had fought right then and there, both of us in the heat of the moment, we might have said hurtful things and ended up both getting hurt. By staying silent for five minutes, we both cooled off; we were able to talk rationally, and the issue was quickly resolved. Another time, a project he was in charge of went wrong and his boss chewed him out. He came home incredibly agitated, everything annoyed him, his words were sharp, and he even snapped at the kids just for dropping toys on the floor. Normally, I would have said, “Why are you taking it out on the kids?” but seeing his mood, I stayed quiet. I simply poured him a drink and sat beside him. He vented to me for over half an hour, complaining about everything from his boss’s scolding to colleagues shifting the blame. Afterward, he calmed down and apologized, saying he was glad I hadn’t argued with him; otherwise, he would have completely lost it. That made me realize that silence can sometimes be a form of companionship—more effective than a lot of comforting words. However, there are definitely negative effects, too. The main one is that he might misunderstand my silence as the silent treatment or think I don’t care about him. He would often say, “When you don’t speak, I feel like I’m shut out, like I can’t get into your heart at all.” The worst instance was the first time we discussed the child’s baptism; I stayed silent all evening and barely spoke to him, going straight to be with the child after dinner, while he sat in the living room until late. Later, he told me he had lain in bed thinking for a long time, wondering if I was simply unwilling to integrate into his life, or even deigned to discuss his most important faith. He said, “When you stop talking, I feel like I’m shut out”—that sense of isolation was incredibly painful for him. That was when I realized just how vastly the meaning of silence differs across cultures. I thought I was avoiding an argument or protecting the relationship, but in his eyes, it was a refusal to communicate—a form of “silent treatment.” Furthermore, remaining silent on certain issues creates lingering emotional knots; the problem isn’t actually solved, just temporarily suppressed. Take the baptism issue, for example: we still haven’t fully talked it through. He’s likely harboring some resentment, feeling I don’t support him, while I’m constantly worried he might secretly take the child to church for the ceremony. It’s like a small splinter. It doesn’t bother you unless you touch it, but the mere thought of it makes things feel awkward.

Researcher: It seems misunderstandings are indeed a common issue in cross-cultural relationships. Did your partner misunderstand your silence right from the start? How would he usually react? Would he ask directly, go silent himself, get impatient, or try to draw you out? How did you view those reactions?

Interviewee: Early in our marriage, he didn’t understand it at all and reacted quite strongly. Whenever I went silent, he would panic and keep pressing me: “What’s wrong? Say something. Are you angry?” His persistence actually made me want to talk even less. I remember one time I was in a bad mood over something trivial and stayed silent for about twenty minutes; he paced back and forth in the living room, getting increasingly agitated, until he finally snapped, “If you have a grievance, just say it. What’s the point of bottling it up? Let’s solve the problem. What does silence actually fix?” Back then, he saw silence as avoidance—a sign of irresponsibility. Later, after we’d been married for two or three years and had reconciled following an argument, I sat down and had a serious conversation with him about it. I explained to him that in Chinese culture, silence doesn’t necessarily mean I’m angry or refusing to communicate; sometimes, I just need time to sort out my emotions. I didn’t want to say hurtful things while in the heat of the moment. It wasn’t directed at him. I also told him about how my parents interacted; I grew up watching them, and silence was simply a habit we used to handle conflict. He didn’t fully grasp it at first, but he gradually became more mindful of it. Now, he’s adapted quite a bit. When he sees me go silent, he doesn’t keep pestering me with questions anymore. Instead, he’ll say, “Okay, I won’t disturb you for now; just let me know when you want to talk,” and then go play with the kids. Occasionally, though, he still can’t help himself. After ten or fifteen minutes, he’ll peek in and ask, “Are you okay?” It’s actually quite sweet.

Researcher: Has he ever done anything particularly memorable to adapt to your silence?

Interviewee: Yes, and it was really touching. Once, he came across an article about communication differences between China and the West. It specifically mentioned that “silence” among Chinese people is understood differently than in the West. He actually saved the article and told me over a meal, “I read this piece today saying that for Chinese people, silence is about ‘thinking before speaking’. It’s not that you don’t want to talk to me, but that you’re figuring out the best way to say things. I was just too impatient before.” I was surprised and really warmed by that; I hadn’t expected him to take the initiative to learn about this and adapt to my habits. As for how I viewed his reaction, at first, I actually thought he was making a big deal out of nothing. It was just silence, right? Did he really need to be so anxious? But later, I came to understand. He grew up in an environment where people spoke their minds directly, whether with family or friends, all emotions were put out in the open. To him, silence signaled a problem in the relationship; it made him uneasy and prone to overthinking, wondering if he’d done something wrong. So, his anxiety and his constant questioning actually stemmed from how much he cared and his fear of losing the relationship. Once I realized that, I was willing to adjust myself and try not to leave him guessing.

Researcher: You’ve mentioned the influence of cultural habits several times. From a cultural perspective, which concepts within Chinese culture, or aspects of your upbringing, do you think have most profoundly influenced your tendency to use silence in your cross-cultural marriage? Take, for instance, sayings like “Harmony in the family leads to prosperity in all things,” “Think thrice before you act,” and “Silence is golden.”

Interviewee: The impact has been immense; you could say that the way I communicate now was largely shaped by the cultural environment I grew up in. First, there’s the idea that “harmony in the family leads to prosperity”—that’s truly ingrained in my very being. My grandmother always told me that when it comes to family life, getting along peacefully is more important than anything else; we shouldn’t be constantly bickering. She’d say that family members shouldn’t hold grudges overnight—a little forbearance makes the trouble pass. So, subconsciously, I came to view “maintaining family harmony” as paramount, often putting my own emotions and grievances on the back burner. As long as the family stayed together and avoided arguments, being a bit silent or swallowing a bit of hurt didn’t matter. My parents were both teachers, so discipline at home was strict; they taught me early on to “listen more and speak less.” Children weren’t supposed to interrupt adults casually, and we had to think things through carefully before speaking. Teachers at school also used to say that children who were composed and spoke sparingly were the sensible ones. Gradually, this became a habit: no matter the situation, I would listen and think first rather than rushing to state my position or jumping in to speak. I used to believe that silence was a sign of maturity and composure—a virtue. It wasn’t until I married a foreigner that I realized, in a different culture, silence could actually be seen as a problem. Then there’s the “culture of mianzi”—saving face—which I mentioned earlier. That has had a profound influence, too. Chinese people place great importance on “face”—both preserving one’s own and saving face for others. Arguing or falling out with someone in public is considered deeply embarrassing; speaking too bluntly or touching on someone’s sore spots is seen as failing to give them face. Consequently, whether I’m with outsiders or at home, I tend to hold back a bit when I speak. I avoid making absolute statements or saying anything too harsh. If I’m upset, I bottle it up and process it in silence to avoid saying something that might cause the other person to lose face or damage our relationship.

Researcher: Aside from your family upbringing, were there other experiences during your formative years that reinforced this mindset of “speaking less and exercising restraint”?

Interviewee: Yes, there were. For example, when I first started working at a company in China, the workplace culture was just like that. You can’t just interrupt a superior when they are speaking; if you have a different opinion, you should voice it in private. Raising objections in public makes the leader lose face. It’s seen as a sign of low emotional intelligence. That’s when I really came to feel that silence serves as both a form of self-protection and a matter of social etiquette. Over time, it became second nature; whether at work or in my personal life, my immediate reaction is to stay silent and observe first, before deciding whether or not to speak.

Researcher: So, do you feel that these cultural norms act as both a safeguard and a constraint in a cross-cultural marriage?

Interviewee: Exactly, that’s spot on. On the protective side, it keeps me from getting into arguments easily or saying hurtful things; I’ve defused many conflicts through silence, keeping the relationship relatively stable. But the constraints are obvious, too: sometimes I have thoughts or feelings, but because I’m used to holding back, I don’t voice them. My partner remains completely unaware, which actually leads to misunderstandings and lets tensions build up. It can also be exhausting, a feeling like I’m constantly suppressing myself, unable to express my emotions as freely as he does. So, I’m gradually adjusting now, trying to find a middle ground.

Researcher: So, from the time you first got married until now, do you feel there’s been a change in how often or in what way you use silence? What are the differences between the beginning and now?

Interviewee: There’s been a huge change. In those first few years of marriage, I would go silent very easily and very often; those silences would last a long time. Sometimes I wouldn’t say much for the better part of a day. Back then, my silence was more of a defense mechanism, a form of avoidance. On one hand, there was the language barrier; I couldn’t express a lot of things, so I just wouldn’t say anything at all. On the other hand, the culture shock was intense. I found many of his habits and views hard to accept, but I felt that even if I spoke up, he wouldn’t understand. It would be a waste of breath. So, I’d just stay silent, withdrawing into a shell to protect myself. It’s different now; I use silence much less frequently, and the duration is shorter, ten or fifteen minutes at most. Most importantly, the silence now is a choice I make, not a passive form of avoidance. Before, I would bottle things up; now, it’s more like, “I need to cool off, so let’s pause the conversation.” The nature of it is completely different. Another obvious change is that I let him know before I go silent. For instance, if I feel my emotions rising or a topic is too sensitive, I’ll just say, “I just need a few minutes to think.” Then I’ll go stand on the balcony or lie down in the bedroom for a bit to compose myself before coming back to talk to him. I don’t just suddenly stop speaking like I used to, leaving him confused and wondering what’s wrong. Also, I speak up about small things right away now instead of bottling them up. If he forgets to take out the trash or doesn’t wash the dishes properly, I’ll just say, “You didn’t take out the trash today; please remember to do it.” I don’t stew over it silently for ages, waiting for him to figure it out on his own like I used to. I’ve realized that addressing small things directly is the easiest approach; there’s no need for silence, which only lets emotions build up.

Researcher: What do you think brought about such a big change? Was it a sense of security from being together longer, or was it a conscious effort to adjust?

Interviewee: It’s a bit of both, I suppose. First, after living together for a long time, we know each other well and understand what kind of person he is. When Americans argue, they stick to the specific issue at hand; they move on once the argument is over, and they don’t hold grudges or bring up past grievances. So, I gradually let go of those concerns; I stopped worrying that speaking up would damage the relationship or that he would hold a grudge. Once I felt secure, I became more willing to express myself. I was also growing personally and came to realize that a good marriage isn’t built on endurance, but on communication. Constant silence and concession might look calm on the surface, but there are undercurrents beneath; if things explode later, the consequences are far worse. It’s better to address small issues openly rather than letting them snowball into major problems. Also, my English has improved, making it less of a struggle to express myself, so I no longer need silence as a way to avoid speaking. Of course, it’s also about him. He’s willing to understand and adapt to me, and I’m willing to change for him; it’s a mutual effort.

Researcher: We’ve discussed a lot regarding your silence in a cross-cultural family. Is there anything else, something we haven’t asked about but you feel is important—that you’d like to add?

Interviewee: I’d like to add that silence actually gave me a new perspective on communication. In a cross-cultural marriage, silence isn’t just about retreating or cowardice; it’s a conscious choice. It allows me to find a balance between two different cultural rhythms. But I’m also learning how to make that silence understood, because silence only counts as communication, rather than a source of distance, when the other person grasps the intent behind it. Another important point is that many people assume silence in cross-cultural marriages stems from poor English or a language barrier, but that’s not the case. Language is only a small part of it; the bigger issues are differences in mindsets and values. Even if you speak English as fluently as a native speaker, there are still conceptual chasms, like religion or views on family, that are hard to bridge. In those moments, silence can actually be a form of respect: respecting each other’s differences without trying to force persuasion or change the other person.

Silence has also helped me gain a clearer understanding of my own cultural identity. Back home, I never thought my communication style was anything special, but after living abroad, I realized that many of my habits and subconscious reactions were deeply ingrained by Chinese culture. My silence isn’t a sign of an introverted personality; it’s a cultural behavior. A cross-cultural marriage acts like a mirror, revealing my true self. Of course, I’m not saying silence is always the right choice. I’m still learning to strike a balance, knowing when to stay silent and when to speak up, and how to gauge that appropriately. You shouldn’t just stay silent, but neither should you force yourself to adopt a completely American-style directness—that wouldn’t be true to who you are. Finding a rhythm that feels comfortable for both of you is what matters most. Don’t bottle things up, but don’t pressure yourself to change overnight, either. If you are naturally inclined toward silence, that’s fine; it’s not a flaw, and there’s no need to doubt yourself just because your partner doesn’t understand it. However, you should at least let them know that your silence doesn’t mean you don’t love them or that you’re engaging in the “silent treatment.” It simply means you need a little time to process your emotions. Don’t make them guess; constant guesswork is damaging to a relationship.

**I-04: (Female, UK partner, with children, married < 3 years)**

Researcher: Okay, let’s start with the overall relationship. Looking back on your experience together in the past few years since you met and got married, what do you think the cross-cultural background, such as the values, ways of thinking, and daily communication habits of both parties, has had the most obvious impact on your intimate relationship?

Interviewee: The impact is actually quite obvious. Let’s talk about values first. I am a typical Chinese person. I always consider the family as a whole when doing anything, such as spending money and making decisions. I always think about “is it good for our little family?” and I have a strong sense of community. He is different. The British have a far-reaching influence of individualistic ideas. They think about everything first: “Do I like it or not? Do I feel comfortable?” Personal independence is ranked very high. Then the way of thinking is also very different. I tend to be tactful in everything I say and do, and I don’t go straight for fear of offending people or hurting their face. He is very direct. If you like someone, you just like him. If you don’t like something, he will say it on the spot. He doesn’t beat around the bush at all. When we first met, I really felt that he had no emotional intelligence. Later, I realized that it was a cultural habit. There are also quite a lot of differences in living habits and holiday concepts. Take the festival as an example. I have felt since I was a child that during the Spring Festival, the whole family must get together to have a lively New Year’s Eve dinner and visit relatives. Reunion is the first priority. But his concept of Christmas is more about partying and carnival with friends, and it does not necessarily have to be tied to family. But having said that, it’s not all bad, because we can also experience different things because of the differences. For example, I will now spend Christmas with him, baking gingerbread and decorating the Christmas tree, and he will accompany me to post Spring Festival couplets and eat dumplings, which is also quite interesting.

Researcher: Can you give me a specific little thing in life that makes you feel that this difference in thinking style or communication habits is particularly intuitive?

Interviewee: Too many, such as cooking. Just a while ago, I made braised pork. He took one bite and immediately said, “This is not delicious.” He said it in front of me. I was stunned for a moment, and I actually felt a little uncomfortable. I felt that I had worked hard for more than an hour, couldn’t you be more tactful? If it were me, even if I thought it didn’t taste good, I would still say, “It’s okay. If I add less salt next time, it might be more to my taste.” I would certainly not directly deny other people’s efforts. In terms of consumption, the difference is also particularly large. After receiving his quarterly bonus, he turned around and bought a limited-edition game console and lens for just 20,000 yuan. Without blinking an eye, he said, “It’s normal for me to buy the things I like with the money I earned.” I can’t. When I get money, my first reaction is to save it for my children to use later, or as a family reserve fund. I always feel at ease when I have savings in my hand. When we first got married, we had a lot of quarrels because of this, but now we are gradually getting along with each other. I have left a “hobby fund” for him, and he spends the fixed pocket money every month as he pleases. We can discuss the big money together.

Researcher: How do you usually coordinate the differences between holidays? Will there be conflicts due to different ways of celebrating the festival?

Interviewee: Fortunately, there are no major conflicts. During the Spring Festival, he would accommodate me and accompany me back to my parents’ house for the New Year and visit relatives with me. Although he didn’t understand many of the rules, such as why he gave red envelopes to children, and he didn’t know how to say auspicious words, he would follow me obediently and give me face. During Christmas, I would spend it with him, invite his foreign friends to have a party at home, roast a turkey, make some snacks, and make it lively. In fact, the main reason is that neither of us is a particularly serious person. We will not insist on the other party’s own culture, and we will just accommodate each other and get over it. But there were some small frictions at the beginning. For example, during the Spring Festival in the first year of marriage, I asked him to stay up until twelve o’clock with me. He thought it was incredible and said, “Why do you have to force yourself not to sleep? It makes no sense at all.” Later, he stayed with me, but he was so sleepy in the middle of the night that he fell asleep on the sofa. It’s funny to think about it.

Researcher: Okay, got it. Then I would like to ask you, can you give me some examples of daily interactions between you and your partner, or the silent scenes that impressed you the most when two people had conflicts? For example, what happened at that time, what was the atmosphere at the scene, and how long were you silent? And what was the main thought in your heart when you chose to remain silent?

Interviewee: It was when we were renovating the house last year. We bought the three-bedroom apartment we live in now, but we have never agreed on the decoration style. He wanted that kind of industrial style, with exposed red brick walls, a fully open kitchen, and a special game corner in the living room. He said it should be personalized and creative, and it would be dignified when friends come to play at home. I think that after having children, the most important thing is that the home should be warm and practical. The wood style is very good, the cabinets have more storage, and the enclosed kitchen fumes will not spread around, making it easy to clean. We talked about this matter many times, and we ended up unhappy every time. The most impressive time was when we sat in the living room after dinner on a weekend night and looked at the 3D renderings sent by the designer. He pointed to the picture of the open kitchen and said he wanted this one, saying it looked open and airy, which fit the feeling he wanted. I told him that an open kitchen is not good. We cook at home every day, and the fumes are huge. By then, the sofas and curtains in the entire living room will be stained with oil, making it difficult to clean at all. He said I was exaggerating, saying that all the British used it, and no one had oil everywhere in their house. It was because I loved cleanliness too much and was too serious about it. As he spoke, he became a little anxious, and raised his voice and said, “Can you have some aesthetics and think about cleaning every day? The house is for living in, not for cleaning.” After he said that, I didn’t want to talk anymore. I just leaned on the back of the sofa and scrolled through my circle of friends aimlessly with my phone. In fact, I didn’t look at it at all. I didn’t know what I was looking at as I scrolled around with my fingers. It was silent for about half an hour, and he didn’t say anything. He sat next to him and flipped through the design drawings on his tablet, making a crackling sound. What were you thinking at that time? First of all, I feel aggrieved. Isn’t it just for this family that I consider practicality? But more importantly, I feel that I am too emotional at the moment. If I continue to say it, I will definitely say more hurtful words, such as “You don’t care about this family at all, you only care about yourself.” Then I won’t be able to stop it. I just want to calm down first and wait until the anger subsides before talking. There is no need to fight to the death.

Researcher: During that half hour, did you expect him to come and talk to you first? Or have you ever thought about breaking the silence?

Interviewee: To be honest, I was a bit looking forward to it in the first ten minutes. I felt that if he came over to coax me with a few words, or took a step back to think about it again, I might just go down the steps. But after waiting for a long time, he didn’t make any movement at all. He was just looking at his design drawings. I became even more angry. I felt that he didn’t care about my feelings at all and was only thinking about his industrial style. Then I thought, forget it, there’s no point in taking the initiative to say it. It doesn’t make sense now anyway, so it’s better to wait. That half hour was actually quite difficult. I was thinking about a lot of things over and over in my mind. Sometimes I thought I was right, and sometimes I thought I was too stubborn. It was just a kitchen.

Researcher: Okay, what about the most impressive scene?

Interviewee: On the weekend a while ago, we went to a park on the outskirts of the city for a picnic with some of his foreign friends. They are all his foreign colleagues here, mostly British, and they all have children of about the same age. They take their children out together to play.

The weather was quite nice that day, neither too hot nor too cold in late spring. Everyone laid out a picnic mat. The men were chatting and drinking cold beer, while the women were running and playing with their children. Later, the topic of parenting came up for some reason, and several fathers complained about the parenting methods in their respective countries. I was squatting next to the mat, wiping the blueberry jam off our child’s hands, when I heard him laughing and saying to his friends that Chinese parenting is too restrictive for children. They have to take care of everything, including what time to eat, what time to go to bed, and what toys to play with. The children have no room for free exploration, and it is completely repressive in nature. He said it in a half-joking tone, but his words conveyed a firm sense of superiority. Several dads around him also echoed his words and looked at me with a smile. I paused while wiping my hands. My fingertips were a little stiff, and then I continued wiping with my head down, without looking up or answering. I just smiled at the child and took him over. For about an hour or so, I didn’t participate much in their chat. I just stayed with the child, picking up leaves and chasing butterflies. I pretended not to hear them when they talked about parenting and concentrated on taking care of the child. When he openly criticized Chinese-style parenting as being too restrictive in front of his friends, I didn’t push back. Arguing would only make him dig his heels in his prejudices, and it would end up hurting my mood. Besides, if I were to get into an argument with him in front of so many outsiders, especially his friends and colleagues, it would be awkward for everyone, and he would lose face. Furthermore, given his personality, the more you argue with him, the more convinced he becomes that he’s right; he’s determined to win the debate at all costs. In the end, not only would I fail to out-argue him, but I’d also end up furious. It simply wouldn’t be worth it.

Researcher: After he said that, did he notice your mood? Did he come over to say anything to you?

Interviewee: He probably did notice, because I didn’t really go over to chat with them or say much after that. At one point, he came over and handed me a bottle of sparkling water and asked if I wanted a sandwich; I just shook my head without even looking up at him. He likely realized I was a bit upset, but he didn’t say anything in front of our friends. He just patted my shoulder and went back to the conversation. He didn’t bring it up on the drive home either; it was quiet in the car. It wasn’t until we got home and the child was asleep that he finally asked if I was angry.

Researcher: I see. Speaking of parenting, do you two often argue about parenting issues? Do you choose to stay silent every time a parenting-related conflict arises?

Interviewee: Not exactly often, but there are definitely differences in our views; we have different ideas every now and then. I don’t stay silent every time. It depends on the situation and the setting. If we’re at home and it’s just the two of us, I usually won’t stay silent; I’ll tell him what I think, or even get into an argument. For example, he likes letting the child run barefoot on the tile floor; he claims that’s how kids do it abroad—that walking on a cool floor builds immunity and prevents illness. I, on the other hand, feel the floor is too cold, especially in early spring when the heating is turned off and the tiles are freezing. Since the child is young and has a sensitive stomach, it’s easy for them to catch a chill and get diarrhea. In those moments, I’ll tell him directly and explain my reasoning. I don’t mind a bit of an argument since there’s no one else around; we get it out in the open and move on. Then there are things like snacks. He thinks it’s fine for the child to have cookies or chocolate since their happiness is what matters most, whereas I feel snacks shouldn’t be eaten before meals because they interfere with proper eating. We bicker over little things like that, but I don’t stay silent; I speak my mind. However, whenever we’re out in public, whether it’s with his friends, my friends, or older relatives. I almost always choose to stay silent. Part of it is about saving face for him; men need to maintain some dignity in public. If I contradicted him right then and there, it would be embarrassing for him, and it would actually make it more likely for us to have a conflict later at home. On the other hand, arguing when outsiders are present not only fails to solve the problem but also makes us look ridiculous to others and ruins the mood for the outing. Besides, as I mentioned earlier, he has a strong competitive streak; if you contradict him in front of others, he’ll just dig his heels in even more. He simply won’t listen. It’s a waste of breath.

Researcher: So, when you argue about parenting issues at home, who usually ends up convincing whom? Or do you both stick to your own views and let the matter drop without a resolution?

Interviewee: Most of the time, it’s a mutual compromise; neither side “wins” completely. Take the issue of going barefoot, for example: we eventually agreed that it’s fine on the play mat and bedroom floor, but socks are mandatory on the living room tiles. Regarding snacks, we agreed on no snacking an hour before meals, though a little fruit or a rice cracker is allowed after the afternoon nap. It’s not really a case of one person convincing the other; we both just take a step back. After all, we both have the child’s best interests at heart. There are times when he listens to me, too. For instance, when the child had a fever for the first time, he was incredibly anxious and insisted on giving fever-reducing medication immediately, fearing the child might suffer brain damage. I suggested trying physical cooling methods first, using a cooling patch and wiping the body with warm water, and monitoring the situation, arguing that medication wasn’t necessary unless the temperature hit 38.5°C. He initially disagreed, citing the practice in his country of medicating fevers right away, but he came around after I showed him some educational material from a pediatrician. There are also times when I listen to him, like when it comes to letting the child eat independently. Even though the child would scatter rice grains everywhere, I initially couldn’t help but feed him because I was worried he wouldn’t get enough to eat. Later, after hearing his point about fostering the child’s autonomy, I gradually learned to let go.

Researcher: I see. Let’s go back to the topic of silence. Aside from the situational factors we just discussed, in what other specific contexts are you more inclined to remain silent? For example, when facing accusations from your partner, discussing sensitive topics, dealing with cultural clashes, feeling exhausted, or experiencing emotions like a sense of grievance or anger?

Interviewee: It happens in all those scenarios you mentioned. There are quite a few situations. The first instance is when I face his baseless accusations. If I’ve actually done something wrong, I’ll admit it; but if he’s being unreasonable, or if he’s taking out his work frustrations on me, I just don’t want to talk. For instance, there was a time when things weren’t going well at work and his boss had scolded him; he came home looking visibly upset. I knew the source of his frustration, but I also knew that anything I said at that moment would fall on deaf ears, or worse, escalate into a heated argument. It was better to let him cool off on his own; eventually, he would realize he was in the wrong. Then there are sensitive topics, like religion. He is a Christian, not particularly devout, but he attends church on Christmas and Easter and keeps a Bible at home. Sometimes he mentions wanting to take our child to church once they’re a bit older, just to expose them to the culture. I feel the child is too young for religious exposure; it’s better to wait until they are old enough to choose their own faith. However, I usually stay silent whenever this topic comes up. Religious beliefs are deeply ingrained, part of who a person is, and I worry that dismissing them as unnecessary or wrong might offend him or hurt his feelings. Besides, there’s no objective right or wrong here; arguing about it only leads to unpleasantness, so it’s better to shelve the discussion for later. Another source of conflict is differing cultural values, specifically regarding elder care and filial piety. He sees nothing wrong with parents moving into a nursing home as they age; he believes professional caregivers provide better care than children can, and that frequent visits are sufficient. I, on the other hand, can’t accept that. I believe that since our parents raised us, we should care for them in their old age—looking after them ourselves whenever possible. Sending elderly parents to a nursing home just doesn’t sit right with me; It looks bad, and people might judge the children as unfilial. Whenever we discuss this, the conversation ends on a sour note, with neither of us convincing the other. Eventually, I just stopped talking about it; since we can’t see eye to eye, it’s better to stay silent and avoid getting angry. There are also times when I’m simply too exhausted to speak. Raising a child is draining, especially when they’re little and wake up multiple times a night. You don’t sleep well, yet you still have to play with them, cook, and clean during the day. It’s a non-stop cycle. Sometimes he’d come home from work, eager to discuss weekend hiking trips or travel plans, but I’d be so tired I could barely think. I wouldn’t want to talk because I lacked the energy to discuss it or the mental bandwidth to plan anything. Finally, I also tend to go silent when I’m feeling aggrieved or helpless. For instance, if he misunderstands me—blaming me when I haven’t actually done anything wrong. I feel deeply aggrieved and don’t want to explain myself. When someone is angry, they won’t listen to explanations anyway; instead, they might think I’m just making excuses or being defensive, which only makes things worse. So, I simply stay silent and wait for him to figure it out on his own.

Researcher: You mentioned choosing silence when the topic of religion comes up. Aside from the fear of offending the other person, are there other reasons? For example, do you feel you can’t articulate the subject clearly in English?

Interviewee: That plays a part, too. It’s a practical issue. Religion is a profound topic; there are many concepts I struggle to explain even in Chinese, let alone English. If I don’t express myself precisely, it could easily lead to misunderstandings, making him think I disrespect his faith. Plus, I don’t know that much about Christianity. I’m unclear on many of the doctrines, so I’m afraid of saying something wrong or looking foolish. It’s better not to say anything; silence is the safest bet. Actually, it’s not just religion; I hesitate to discuss many topics that touch on deep cultural issues for fear of failing to convey my meaning and making things more confusing.

Researcher: So, how does the silence born of exhaustion differ from the silence caused by anger or a sense of grievance?

Interviewee: They are completely different, two totally distinct states. Silence due to exhaustion is simply a lack of energy to speak; there’s no negative emotion involved, no anger or unhappiness, just fatigue. If he speaks to me then, it’s not that I don’t want to engage; I genuinely lack the energy to respond. My mind feels foggy and sluggish. Silence caused by anger or grievance, however, is emotionally charged. It’s a deliberate choice not to speak because I’m angry or harboring resentment, a conscious decision to avoid communication. Also, exhaustion passes once I recover, a good night’s sleep fixes it. But with angry silence, if the issue isn’t resolved, the discomfort lingers, and I feel a heavy, stifling weight in my chest.

Researcher: I see. So, what do you consider the primary reason for choosing silence? Is it to avoid arguments, a feeling that things can’t be explained clearly, a desire to maintain harmony, or to save face for both parties? Or is it influenced by cultural norms, the pressure of expressing yourself in a foreign language, or simply not knowing how to respond in the moment?

Interviewee: There are many reasons, a whole mix of them, rather than just one single factor. Cross-cultural marriages involve significant differences to begin with; if you argue over every little trivial matter, life becomes impossible. There are many Chinese concepts and social nuances that I could explain to him for ages without him grasping them. It’s like playing the lute to a cow. Then there’s the cultural conditioning: growing up, I constantly heard sayings like “silence is golden,” “the more you say, the more likely you are to slip up,” and “harmony in the family leads to prosperity.” After hearing these for over twenty years, it’s ingrained in me that speaking less does no harm, especially during a conflict, where saying less prevents things from escalating. Plus, we Chinese tend to be reserved; we don’t like to wear our hearts on our sleeves. Whether it’s emotions or thoughts, we’re used to keeping them inside, believing that if the other person truly understands you, they’ll get it without needing a lot of words. The pressure of expressing myself in a foreign language is a very real factor, too. Although my English is decent—fine for daily conversation and work—when I’m genuinely angry or emotional, my brain defaults to Chinese. Formulating thoughts into English takes time, and the delivery lacks impact; sometimes I fail to convey the exact meaning, which gives him openings to pick apart my argument. Rather than stumbling through a clumsy explanation, I’d rather stay silent, calm down, and organize my thoughts before speaking. Otherwise, I can’t win the argument, which is even more infuriating. Sometimes, I simply don’t know how to respond in the moment, so I fall silent. For instance, when he suddenly told me his mother was coming to stay for two months, I was completely blindsided. I didn’t know whether to agree or object. Agreeing felt inconvenient—our lifestyles, daily routines, and views on child-rearing are all different. But disagreeing made me worry he’d think I was being unfilial or unwelcoming toward his mother. In that instant, my mind went blank; I couldn’t think of what to say, so I just stayed silent to catch my breath and process things before responding.

Researcher: You mentioned the pressure of expressing yourself in a foreign language—could you elaborate on that feeling? Was there a specific instance where it was particularly noticeable, where you felt you couldn’t explain yourself clearly in English, so you just gave up on saying anything?

Interviewee: Oh, absolutely. There have been so many times. For example, we once had a disagreement about “boundaries.” I wanted to explain that in Chinese intimate relationships, boundaries aren’t drawn so rigidly; couples are expected to be involved in each other’s lives. To us, that involvement signifies care and closeness. It’s not about trying to control the other person. But I kept struggling to find the right English words to express that feeling of “no boundaries between us”—that sense of shared life. No matter how much I explained, he didn’t get it; he just thought I was trying to control his social life and his time. Eventually, I just stopped talking about it. The more I said, the more confused things got, so silence seemed like the better option. Honestly, arguing in a second language puts you at such a disadvantage—you can’t articulate your point even when you’re right, so it’s often easier to just say nothing.

Researcher: So, what kind of impact does your silence have on your communication or your relationship? Does it temporarily maintain harmony and defuse conflict? Does it escalate tensions and leave your partner confused or hurt? Or does it give you both time to cool off, even if it risks leaving unresolved issues? Could you share some specific examples?

Interviewee: The upside is that it really does temporarily defuse conflict and give us both time to calm down. When people are angry, they can say some pretty hurtful things; silence helps prevent the argument from spiraling out of control. Take the time we were looking to get a new car, for instance. He wanted a two-door sports car. He thought it looked cool and would make him look good when driving it. I wanted an SUV. I thought it would be more practical for taking the kids out, with plenty of space and better safety. We had a pretty heated argument at the time. He said I didn’t understand cars and only cared about practicality—that I was boring. I accused him of not caring about the family and only thinking about his own fun. We were getting more and more worked up, and it looked like we were about to start digging up old grievances. Realizing things were going south, I stopped talking, turned around, went into the bedroom, and closed the door. After cooling off for the afternoon, I thought about it in the bedroom and realized it was normal for him to like sports cars—most men do. He’d been thinking about it in the living room, too. At dinner that evening, he brought it up himself; he said he’d thought it over and agreed that an SUV was more practical. It would be easier for taking the kids out or driving the elderly parents around. He said we could wait until the kids were older to get a sports car. You see? That moment of silence let us both calm down and think rationally. If we’d kept arguing right then, who knows how bad it could have gotten? But there’s a clear downside, too: it can easily leave unresolved issues, especially if the other person doesn’t know why you went silent. For example, there was an incident involving a female colleague of his. She was on the same project team and would often message him in the evenings. At first, it was all work-related, but later she started sharing bits of her daily life, like what coffee she’d had or a cute cat she’d seen on the street. It made me feel uncomfortable when I saw it, but I didn’t say anything; I assumed they were just ordinary colleagues. Once, while he was showering and his phone was on the living room coffee table, that woman sent another message asking to treat him to a meal sometime. I happened to walk by and saw it; I felt terrible in that moment. When he came out of the shower and noticed I looked upset, he asked what was wrong, but I just shook my head without saying a word. I barely spoke the rest of the evening; I gave only perfunctory replies when he tried to talk to me, mostly remaining silent. He found my behavior baffling and got a bit angry, thinking I was giving him the cold shoulder for no reason and didn’t trust him. We ended up in a standoff for two days—hardly speaking, with a really heavy atmosphere at home. Even our child sensed it and became unusually well-behaved. Although we eventually cleared the air, and he promised to be mindful of boundaries, keeping work discussions at the office and avoiding personal chats outside of work—that awkwardness left a lingering sense of unease. I felt that if I had just spoken up right away, we could have avoided that two-day silence and the mutual distress. But at the time, I kept it bottled up; I felt that saying something would make me look petty or like I didn’t trust him.

Researcher: How did you eventually resolve the issue? Who initiated the conversation?

Interviewee: I did. Bottling it up for two days was unbearable, and I knew that kind of situation wasn’t sustainable. It wasn’t good for the child, either. The next evening, after the child had gone to sleep, I sat on the living room sofa waiting for him to finish work. When he came out, I told him I wanted to talk about his colleague. I voiced my concerns—explaining that seeing them discuss personal matters made me uncomfortable; it wasn’t that I didn’t trust him, but rather that it bothered me a bit. He was taken aback at first, then apologized, admitting he hadn’t considered my feelings. He insisted they were strictly colleagues—that the woman was just naturally lively and acted that way with everyone on the team. He promised to be more mindful of boundaries, stop discussing non-work matters, and try to avoid one-on-one interactions. We both felt relieved after that conversation. The issue itself wasn’t actually a big deal; the silence had just blown it out of proportion. Him guessing what was wrong while I kept it all inside only made things worse. Researcher: Aside from leaving lingering hurt feelings, have there been instances where silence caused a conflict to escalate?

Interviewee: There was one time. Shortly after we got married, we argued over where to spend the Lunar New Year. I wanted to go to my parents’ place, but he wanted to go back to the UK to spend the holiday with his parents; after all, it was our first year of marriage, and he wanted to visit his family. As the conversation went on, I felt like I couldn’t get through to him, so I just stopped talking, went to our room, and closed the door. He thought I was giving him the “silent treatment,” deliberately ignoring him, so he got angry too. He sat in the living room all night without speaking to me or coming back to the bedroom. What started as a minor issue, something that could have been resolved with a proper discussion, turned into a standoff lasting nearly a week because of that silence. Eventually, a close friend of mine advised me that in cross-cultural communication, you can’t rely on guesswork; you have to speak up, because if you don’t, he’ll never know why you’re upset. So, I took the initiative to talk to him. That’s when I found out he had no idea why I was angry; he thought I simply didn’t want him to visit his parents and considered me selfish. Since then, I’ve realized that silence shouldn’t last too long, or else a small matter can blow up into a major issue.

Researcher: I see. How does your partner react to your silence now?

Interviewee: We’ve been together for nearly three years now, so he’s gradually figured out my temperament; he doesn’t keep pestering me anymore. He gives me space and doesn’t force me to speak right away. He’ll wait an hour or two until he sees I’ve calmed down a bit, then come over and ask softly, “Can we talk now?” If I still don’t want to talk, he won’t ask again; he’ll just wait a while longer. He’s incredibly patient. Of course, there are times when he gets impatient, too. For instance, if I stay silent for too long, or if I clam up repeatedly over similar issues, he’ll get a bit emotional and sigh, saying, “Can you not always be like this? If there’s a problem, just say it. When you stay silent, it’s exhausting for me. I’m left guessing, but I just can’t figure it out.” Whenever he says that, I feel a twinge of guilt; I know he genuinely doesn’t know what to do and isn’t trying to pressure me on purpose.

Researcher: Has he ever explicitly mentioned that he views your silence as a form of “cold violence” [the silent treatment]?

Interviewee: Yes, he brought it up once right after we got married. It really stuck with me. That time, we were giving each other the silent treatment over where to spend the Lunar New Year. After we made up, he told me that he felt my silence was a form of cold violence—that it was even more painful than arguing. He said that with an argument, at least you know what the other person is thinking and where the conflict lies, whereas silence leaves him feeling helpless, with no idea what to do. For him, it’s actually a different kind of hurt.

Researcher: From the time you first married until now, has there been any change in the frequency or way you use silence in this cross-cultural marriage? How does the situation now compare to the beginning?

Interviewee: There have definitely been changes—significant ones. After nearly three years of adjusting to each other, it’s impossible for things to have stayed exactly the same.

Back when we first got married and started living together, all sorts of cultural differences and lifestyle habits clashed. We’d have minor squabbles every few days and major arguments every week. I felt like I couldn’t communicate about many things, so I’d just resort to silence. For example, he wouldn’t put things back where they belonged after using them, he’d leave his socks lying around, and he’d smack his lips while eating. These little things really bothered me, but I didn’t want to nag him every day. I didn’t want to be annoying like a nagging mother, so I’d bottle it up and use silence to express my dissatisfaction. Back then, the periods of silence were long, too; I could hold it in for half a day or even a whole day, refusing to speak to him and just stewing in my own frustration. We’ve been married for nearly two and a half years now, and after gradually adjusting to each other, the frequency of those silences has dropped significantly, maybe once every month or two. Even when silence does occur, it lasts a much shorter time; usually an hour or two, or half a day at most, before I take the initiative to communicate. It’s not like before, where I’d bottle things up and wait for him to guess what was wrong, getting even angrier if he couldn’t figure it out. The nature of the silence has changed, too. Early on, the silence was mostly emotional. I was angry or felt wronged, so I’d give him the cold shoulder out of spite. I wouldn’t think about a resolution; I’d just stew in it, waiting to see who would back down first. Now, the silence feels more like a conscious “cooling-off” period. If I feel my emotions aren’t right in the moment, or if a conversation hits a dead end, I’ll go quiet for a bit to calm down and figure out exactly what I want to say before actively addressing the issue. When I go quiet now, I have a plan. I know what I’m going to say to him later, rather than just acting out of spite.

Researcher: What do you think drove this change? Was it a natural result of getting used to each other over time, or were there specific incidents that made you want to change?

Interviewee: It was a mix of both. On one hand, after living together for a while, we’ve come to understand each other’s temperaments. Knowing that he doesn’t have bad intentions—that we just have different perspectives—makes me less prone to getting angry. Plus, I’ve gotten used to a lot of the little things. For instance, if he leaves his socks lying around, I’ll just pick them up and toss them in the laundry basket now; I don’t get worked up for ages like I used to, viewing it as some terrible habit. On the other hand, there were definitely a few incidents that made me realize staying silent wasn’t working. There was that time I mentioned earlier—the week-long “cold war,” and the time he accused me of using the silent treatment as a form of emotional abuse. That’s when I realized that, from his perspective, my silence wasn’t a way to avoid conflict. It was hurtful. Also, bottling things up doesn’t solve anything; issues just pile up until there’s a massive blow-up that’s much harder to deal with.

Researcher: If you compare yourself to your Chinese female friends, those in non-intercultural marriages, do you think there’s a difference between how you use silence in your intercultural marriage and how they use it in their relationships? If so, what are the main differences?

Interviewee: It’s probably a bit different. I often vent about this to my close girlfriends. I have a very close friend whose husband is also Chinese; they’ve been married about as long as we have and have kids, too. I know their dynamic inside out. When they argue, my best friend never holds back; she voices her grievances immediately. Even if the fight gets heated, sometimes involving thrown objects, they make up right after her husband offers a few soothing words, acting as if nothing happened. Even when there is occasional silence, it’s usually just a huff, waiting for the other person to coax them out of it, and it never lasts long; a little reassurance fixes things. For instance, they once argued over who would take the child for vaccinations; her husband claimed he was too busy with work, and she just exploded: “You’re busy, but am I not? Is the child mine alone?” She let it all out in a torrent of words. He quickly admitted he was wrong and took the day off the next day to go with them. If it were me, I’d probably just stay silent and take the child myself; I wouldn’t want to argue, feeling that speaking up wouldn’t change anything. I think the main difference is that their silence is less burdened by overthinking. It’s more spontaneous. That said, personality plays a role too, so you can’t simply say it’s a black-and-white difference. I’m actually quite introverted; when I’m with my best friend, I don’t talk much. I tend to be the type who just swallows things and puts up with them. I’m generally quite cautious about how I handle things.

Researcher: Alright, that covers most of our questions. Is there anything else regarding your silence within an intercultural family, something we haven’t asked about but you feel is important or would like to add?

Interviewee: In an intercultural marriage, silence can sometimes serve as a buffer or a form of self-protection. After all, when two people from completely different cultural backgrounds come together, there are bound to be clashes. If you tried to hash out every single issue, it would be exhausting—mentally and emotionally draining. A bit of silence, giving each other some space, can actually help stabilize the relationship. But the caveat is that silence shouldn’t be a form of avoidance; you can’t just bottle up problems and pretend they didn’t happen. After the silence, you still need to communicate properly and clear the air. Otherwise, the issues pile up, and eventually, there will be an explosion that’s much harder to deal with. Also, it can’t just be one person staying silent; the other needs to try to understand, too. I’ve started telling him, “I’m staying silent not because I’m angry, but because I need time to think,” and he’s gradually come to accept that. Sometimes, when he encounters Chinese customs he doesn’t understand, he goes silent too, and I don’t pressure him to accept them immediately. In an intercultural marriage, you really cannot rely on just one partner to compromise. I am learning to express myself more directly and speak up more often, while he is learning to understand my reticence and the meaning behind my silence. Only when both partners meet halfway can the relationship go the distance. People often say intercultural marriages are difficult; the challenge lies in the fact that you must accept not just the individual, but the entire cultural background and mindset they bring with them. Silence is just a small part of this, but beneath it lies the collision and adjustment of two distinct cultures. Furthermore, I don’t think silence is exclusive to the Chinese partner; the non-Chinese partner goes silent too. For instance, when I talk to my husband about Chinese social customs, like the intricate web of extended family relationships or the tradition of fenziqian (monetary gifts), He sometimes doesn’t grasp the nuances; he falls silent, unsure of what to say. So, silence is actually a two-way street; it’s just that our culture values reserve, so we might employ it more frequently. There is no need to view the Chinese partner’s silence as something extraordinary; it is simply a form of communication. When used well, it reflects wisdom in how we get along; when used poorly, it becomes an obstacle to the relationship.

**I-05: (Female, UK partner, with children, married > 10 years)**

Researcher: I see; that’s very clear. Looking back at the more than ten years you’ve spent together, from your initial cross-cultural romance to your long-term marriage, what do you think were the most significant impacts of this cross-cultural background (such as differences in values, mindsets, and communication habits) on your intimate relationship?

Interviewee: When we first started dating, the cultural differences felt like a novelty—things like his quintessential British humor, his politeness, and his respect for personal space were all things I really appreciated. But once we actually got married, and especially after having children and living under the same roof—those differences turned into real clashes. British communication habits are actually quite interesting; on the surface, they are incredibly polite and courteous, yet when it comes to resolving conflicts in an intimate relationship, he places huge importance on verbalizing everything. He believes that as long as a problem is laid out openly and the logic is sorted through, it can be resolved. I, on the other hand, grew up with a traditional Chinese upbringing; my mindset leans more towards “understanding without explicitly stating things.” I feel that sometimes it’s better not to be too blunt or absolute—that if everyone understands the situation, there’s no need to spell out every single word. These differing communication styles led to a lot of misunderstandings early on. He felt I wasn’t transparent enough and tended to bottle things up, while I felt he was too aggressive, insisting on dissecting every emotion and detail. Then there were the issues of family boundaries and values. In the Chinese concept of family, the individual and the family are deeply intertwined and mutually permeable; for instance, when it comes to educating children or caring for aging parents, we are accustomed to pouring all our energy into it, sometimes even sacrificing personal comfort for the sake of the family. He, however, was raised with an individual-first mindset; even after starting a family, he continued to place great value on personal space and individual boundaries. In those first few years of marriage, I often thought he was a bit cold-blooded or overly calculating, while he felt I sometimes overstepped or placed too much weight on the opinions of elders.

Researcher: So, in your daily interactions or when conflicts arise, do you ever fall silent? If so, could you describe one or two specific instances where this silence was particularly memorable? For example, what was happening at the time, what was the atmosphere like, how long did the silence last, and what was the primary thought going through your mind when you chose to remain silent? Interviewee: Yes, absolutely. And to be honest, silence is something I resort to quite often in my marriage. When my child had just started first grade, my mother was staying with us to help with the school run. You know how older Chinese people are. She was deeply concerned about the child’s homework and would hover nearby, constantly urging the child along. One evening, the child was slow with the homework, and my mother made a few nagging comments; the child burst into tears. When my husband came home from work and saw the scene, his expression changed instantly. He didn’t lash out at my mother directly, but he walked over, picked up the child, and said to me, with a very serious, almost icy British politeness, “I feel your mother’s way of teaching is undermining the child’s self-confidence. This is our home, and I don’t want the child growing up in a state of anxiety.” The atmosphere in the living room froze right then and there. My mother stood there, incredibly awkward; she didn’t grasp the full picture, but she knew her son-in-law was angry, and her eyes held a mix of hurt and a desperate desire to please. My husband looked at me, waiting for me to speak up, to either take a stand or join him in setting some ground rules and clarifying things with her. But in that moment, I couldn’t utter a single word. I fell completely silent.

Researcher: How long did that silence last? What was going through your mind during that time?

Interviewee: From the moment he finished that sentence, I just lowered my head and walked into the kitchen to clear the table without saying a word. For the rest of the evening, aside from whispering “It’s okay, you go ahead and sleep” to my mother in Chinese, I didn’t say a single thing to my husband. That silence lasted nearly a full day and night; we didn’t speak to each other again until the following evening. My inner thoughts were incredibly complex. It was like a fierce internal struggle following a moment where my mind had gone completely blank. I felt deeply aggrieved. My mother worked so hard helping us with the child, yet he didn’t offer a word of thanks; instead, he outright dismissed her because of a difference in parenting philosophies and showed her no consideration. He simply didn’t understand the sacrifices made within a Chinese family. But another voice immediately silenced me, telling me: “Once you speak up, this stops being just a quarrel between husband and wife; it turns into a clash between Eastern and Western cultures—a son-in-law challenging his mother-in-law. If I start lashing out, my mother—watching from the sidelines—will be even more mortified, and the whole household will erupt in chaos tonight.” So, I chose silence. That silence wasn’t a case of “having nothing to say,” but rather the realization that “if I speak now, every word I utter will act like dynamite.”

Researcher: Based on what you’ve just mentioned, let’s break this down: in which specific situations are you most inclined to remain silent? For instance, when facing accusations from your partner, discussing sensitive topics, dealing with cultural clashes, feeling exhausted, or experiencing emotions like grievance, helplessness, sadness, or anger?

Interviewee: If I had to categorize them, there are a few typical scenarios. The first is when I feel that the cost of explaining myself verbally is too high and bound to be futile, especially regarding deep-seated cultural clashes. In those moments, if he criticizes me, I usually choose silence. I know it’s not a matter of English proficiency; it’s a collision of two sets of values shaped over decades. It’s hard to convince him during an argument lasting only minutes or hours; explanations just turn into excuses, and excuses only trigger a bigger debate. Then there are times when I feel extremely aggrieved, disappointed, or angry—when emotions run high. I’m the type of person whose throat tightens up when I’m emotional; if I forced myself to speak, I might cry or lose control. The way we were raised, losing control is seen as undignified and irrational. To preserve my last shred of dignity and composure, I choose silence first. Another crucial point is when facing extremely sensitive topics, such as politics, ideology, or differing historical perspectives. You know, Chinese and Western media often report on political events from vastly different angles. Whenever my husband starts talking about Chinese politics, I simply don’t bite. By staying silent and shifting the focus away from sensitive topics, I avoid offending him without letting things get awkward. And finally, there are times when I am simply physically and mentally exhausted. After a full day of work, I still have to look after the kids and handle household chores when I get home; if he tries to engage me in conversation or nitpick over trivial matters at that point, I don’t even have the energy to get angry, so I simply respond with silence.

Researcher: Looking at the underlying reasons, what was your primary motivation for choosing silence? Was it to avoid arguing, maintain harmony, save face (for yourself or your partner), cultural influences, the pressure of expressing yourself in a foreign language, or simply not knowing how to respond at the moment?

Interviewee: If I were to rank them by importance, I’d say my motivations operate on different levels. The core motivation was maintaining harmony and saving face. Marriage is a long-term partnership, not a debate competition; there’s no need to determine who is right or wrong every single time. Winning an argument at the cost of the relationship, or tearing a rift in it that can’t be mended, simply isn’t worth it. My English is actually quite fluent, and I use it daily for work, but when you’re in the heat of an argument with him, you certainly can’t out-argue him in English. When I’m really angry, I have to mentally translate my thoughts into English first. It’s exhausting, and sometimes I feel like the words that actually come out don’t quite capture what I truly wanted to say. If I’m flustered and can’t find the precise English word, I feel a deep sense of powerlessness. Another reason is that I want to give us both a cooling-off period. I’m someone who needs solitude and time to process negative emotions. When I feel offended or blamed, I can’t just sit down immediately like he does, and look the other person in the eye to sort through issues point by point. I need to calm down, quell my anger, and figure out exactly what’s upsetting me before deciding whether to discuss it. If I responded right then and there, I might say things that are incredibly hurtful.

Researcher: So, in terms of actual outcomes, what impact does this silence have on your communication or relationship? Does it maintain harmony and defuse conflict, or does it escalate tensions and leave your partner feeling confused or hurt? Could you share some specific examples?

Interviewee: There are certainly positive effects. The most obvious one is avoiding a lot of hurtful words. Take the earlier example of the conflict between my mother and him regarding child-rearing: if I had gotten into a shouting match with him right in front of my mother, I might have said things that were impossible to take back. Even though my silence made the atmosphere icy at the time, it did prevent the conflict from escalating further. By the next day, once everyone had calmed down, there was at least room for a rational conversation. However, there can be negative consequences, too. As a native Brit, he doesn’t have this concept in his cultural background. In his view, communication is the only way to resolve conflict, whereas silence is usually interpreted as a refusal to communicate, or even a sign of arrogance. Once, we had an argument over something trivial; I habitually shut down, stopped speaking, and retreated to my study, closing the door behind me. I thought I was simply cooling off, processing my emotions, and preventing the conflict from escalating. But half an hour later, he stormed in, clearly agitated. He told me, “That attitude of yours really hurts. You can get angry at me, argue with me, or even blame me, but you can’t treat me like I don’t exist. I feel shut out and punished.” That conversation really struck a chord with me. I realized then that my silence came across as aggressive to him.

Researcher: So, when faced with your silence, does he take the initiative to ask what’s wrong? Does he try to draw you out? And how do you view these reactions of his?

Interviewee: Yes, he does; he’ll ask why, what happened, or what’s the matter. If he asks a few times, I’ll definitely answer him eventually. I feel like he’s taking my feelings into account, too.

Researcher: After a period of silence, how do you usually “break the ice” and resume communication? Does one of you speak up first? Is it through physical contact, like a hug? Or is it a natural transition back through everyday activities?

Interviewee: Usually, he’s the one who initiates it. For instance, he might ask what I’d like to drink or eat, using a tone that’s as neutral as possible—completely devoid of emotion. If I’m no longer in the heat of the moment, I’ll respond positively.

Researcher: Do you explain the reasons for your silence to your partner afterward? Does that kind of retrospective explanation help reduce misunderstandings?

Interviewee: Yes, I definitely explain it. Once I realized he didn’t understand my silence or my tendency toward reticence at all, I knew I had to explain things. After we’d hit a few communication roadblocks, we had a serious talk about it one calm weekend evening while having drinks and chatting in the garden. I explained to him that when I chose silence in the heat of anger, it was because I needed time to process my emotions and calm down, and because I cared deeply about the relationship. It was a way for me to self-regulate, so I needed a little time. I was very sincere when I said this, and he took it to heart. Since then, whenever a conflict arises and I go silent, I make sure to say, “I need a moment to cool off. It’s not that I’m ignoring you; I just need some space.”

Researcher: From a cultural perspective, do you think certain concepts in Chinese culture, such as “harmony brings prosperity to the family,” “think thrice before acting,” “silence is golden,” “loose lips lead to trouble,” or “be cautious in speech and action,” or perhaps your upbringing, have deeply influenced your choice to use silence in your cross-cultural marriage?

Interviewee: To begin with, I grew up in a very typical traditional Chinese family. As far as I can recall, my parents rarely had loud arguments, threw things, or screamed in front of me. When they had a disagreement, the most common scenario was the silent treatment or simply finding something else to do while quietly letting their anger subside. My mother often taught me: “A girl should be composed; think before you speak, because saying too much leads to mistakes.” She’d say, “When living together as a family, taking a step back opens up a world of possibilities; a little forbearance gets you through the hard times.”

Subtly, I absorbed the message that expressing anger was undignified and openly escalating a conflict was a sign of incompetence, whereas tolerance and restraint were signs of maturity, good breeding, and a commitment to the bigger picture. So, when I encounter conflict in my marriage, my first instinct isn’t to attack or argue, but to exercise restraint. Silence is the outward manifestation of that restraint. Also, as I mentioned earlier, it’s easy to say hurtful things when you’re angry, so it’s crucial to think carefully before speaking.

Researcher: Do you feel that the frequency or manner in which you use silence in your cross-cultural marriage, or your understanding of silence itself, has changed? How does the present compare to those first few years of marriage?

Interviewee: In the early years of our marriage, when I was angry and went silent, I would wait for him to guess what was on my mind and wait for him to try to appease me. If he didn’t guess or guessed wrong, I’d get even angrier, and the silence would drag on, from one day to two or even three. Honestly, that really took a toll on our relationship and left behind a lot of unresolved emotional knots. But now, I know when to stay silent and when to speak up. The duration of the silence has shortened; whereas I used to clam up for two or three days, now it’s usually just a few hours or half a day, once I’ve processed my emotions, I take the initiative to revisit the issue. The silence has become more transparent, too. As I mentioned earlier, I’ll tell him upfront that I need some quiet time to cool off, clarifying that it’s not directed at him. I’m also more selective about when I use silence. For trivial matters, pure emotional venting, or irreconcilable, broad issues, I use silence to let things slide without wasting energy. However, for matters involving principles, our child’s upbringing, or core family interests, I express myself with absolute clarity and precision once I’ve calmed down; I never use silence as a substitute for communication anymore.

Researcher: Finally, regarding your use of silence within your cross-cultural family, is there anything we haven’t asked about that you feel is important or would like to add?

Interviewee: I think silence is actually a very complex behavior. It’s hard to fully articulate. In a cross-cultural marriage, when two people from different worlds live under the same roof, language can sometimes feel inadequate or even destructive. At certain moments, a measured silence isn’t about giving up on communication; it’s about giving each other space to redefine the boundaries of the relationship. Of course, this kind of silence relies on mutual understanding, trust, and effective follow-up communication. If it’s just a matter of bottling things up and endless silence, the marriage is bound to run into serious trouble. So, active communication is definitely crucial.

**I-06: (Female, UK partner, with children, married 4–10 years)**

Researcher: Do you ever go silent during your daily interactions with your partner or when conflicts arise? If so, could you describe one or two specific instances that left a strong impression on you, such as what happened, the atmosphere at the time, and how long the silence lasted? What was the primary thought going through your mind when you chose to remain silent?

Interviewee: Because of our different upbringings and educational backgrounds, we often have differing subjective views on minor matters. Initially, I felt that once we had a child, the child should become the top priority; however, he believed the couple’s relationship should always come first. I was eventually influenced by his perspective, and now we both prioritize each other, which we feel benefits our relationship.

Researcher: In what specific situations are you more inclined to remain silent? For example, when facing accusations from your partner, discussing sensitive topics, dealing with a clash of cultural values, feeling exhausted, or experiencing emotions like grievance, helplessness, sadness, or anger?

Interviewee: Yes, it happens, especially when we were in the UK. For instance, if we argued about the same issue repeatedly and it came up again, I would ask why it was happening once more; if his attitude remained unchanged and he failed to acknowledge his mistake, I would choose silence. I felt there was no point in saying anything more. It was a waste of breath since we’d already discussed it so many times. Plus, arguing in English meant I couldn’t really win the argument anyway, so I figured I might as well save my energy.

Researcher: What are the main reasons you choose silence? For example, wanting to avoid an argument, feeling it’s impossible to explain yourself clearly, wanting to maintain harmony, protecting your own or your partner’s face, the influence of cultural values, the pressure of expressing yourself in a foreign language, or simply not knowing how to respond at the moment?

Interviewee: When discussing a topic I’d rather avoid, I might respond at first, but if the other person keeps pressing after I’ve replied or indicated I don’t want to talk about it, I’ll choose silence. I also choose silence when I know I’m in the right but can’t articulate my thoughts due to language barriers, or when I feel an argument is meaningless and won’t lead to a resolution. I tend to go silent quite often; I don’t think it’s entirely because we come from different countries. It’s just my personal way of dealing with problems. I prefer to keep things to myself and process them internally. Researcher: What impact does your silence have on your communication or relationship (e.g., maintaining harmony, easing conflict, escalating tension, confusing or hurting your partner, allowing time to cool off, leaving unresolved issues/resentment, etc.)? Could you give an example?

Interviewee: I just can’t be bothered to argue. I feel like I wouldn’t win the argument anyway due to language barriers, so I simply choose not to speak.

Researcher: Does your partner misunderstand your silence? How do they usually react to it. For instance, by asking questions, choosing silence themselves, showing impatience, or trying to draw you out? What is your take on these reactions?

Interviewee: Because I often go silent, it eventually became a standard pattern for how we handle arguments: the silent treatment comes first. We ignore each other for a while, then talk things over once we’ve calmed down. Actually, this works better for us; the issues get resolved more effectively. If he wants to keep discussing the matter while I’m staying silent, he’ll try to prompt me with questions, but I usually just keep quiet. I don’t have any particular opinion on it other than knowing that if I stay silent, he gets anxious. Since I can’t out-argue him, this is the best strategy I’ve found.

Researcher: From a cultural perspective, do you think any concepts from Chinese culture (such as “harmony brings prosperity,” “think twice before acting,” “silence is golden,” “loose lips sink ships,” or “being cautious in speech and action”), or perhaps your upbringing. Influence your choice to use silence in an intercultural intimate relationship?

Interviewee: I don’t think those traditional cultural ideas are the reason I choose silence; it’s more likely due to my personality. I’m not really a traditional person.

Researcher: Do you think the frequency or manner in which you use silence in your intercultural marriage has changed? How does the current situation compare to the beginning?

Interviewee: It happens much less often now. Especially since we moved back to China, I feel much more at ease, and we’ve grown closer. We rarely argue these days; we talk things through openly, and he’s become more patient, too.

Researcher: Comparing your communication style with that of your Chinese friends of the same gender (who are not in intercultural relationships), do you think there is a difference between your use of silence in an intercultural family and how they use silence in their intimate relationships? What is the main difference?

Interviewee: I don’t think there is a difference. In both cases, it’s a choice made to cool off after an argument reaches a point where it’s going nowhere and both sides start attacking each other personally rather than addressing the issue.

Researcher: Regarding your tendency to go silent in a cross-cultural family setting, is there anything else, something we haven’t asked about but you consider important—that you’d like to add?

Interviewee: I have to admit that language is a barrier that leads to more frequent silence, but I feel it’s largely a matter of individual personality.

**I-07: (Male, Australian partner, without children, married < 3 years)**

Researcher: Looking back at your time together, what do you think were the most significant impacts of your differing cultural backgrounds, such as values, mindsets, and communication habits, on your intimate relationship?

Interviewee: I think the impact was actually quite profound, though I didn’t realize just how significant it would be at the start. Before we got together, we both felt that if you like someone and your personalities click, cultural differences shouldn’t be a major issue. Plus, having studied abroad myself, I thought I was reasonably open to Western culture. But after we married, and especially once we started living together. I discovered that cultural differences actually permeate so many little things. For instance, we had different understandings of what “showing care” looks like. If I came home from work and saw she was tired, I wouldn’t necessarily ask a lot of questions; instead, I might just cook dinner or take care of household chores. To me, that was showing care. But sometimes she would ask, “Why didn’t you ask how my day went?” I found that a bit odd at the time. After all, hadn’t I already cooked the meal? I eventually realized she needed verbal confirmation. She wanted me to actually say things like, “Are you really tired today?” or “Did something happen?” I tend to express myself through actions, whereas she places more value on verbal expression. Yes, I think that difference is particularly striking. Another major difference was our approach to addressing problems: should we talk about them immediately? She had a habit of wanting to clear the air right away if something made her uncomfortable; she’d say, “Let’s talk about it.” But I was actually quite intimidated by that kind of sudden, face-to-face confrontation. Growing up in China, conflicts in my family often weren’t voiced immediately. If my parents were angry, they wouldn’t explicitly say, “I’m angry because of X”; instead, they might suddenly go silent, or the atmosphere at the dinner table would just feel off. After a while, things would return to normal. At least in my experience, that was the norm. So, at first, I couldn’t understand why she insisted on hashing things out right away—couldn’t we just let some things slide?

Researcher: Do you think this difference affected how you both interpreted “silence”? Could you give some examples of times when silence occurred after a conflict or argument? For instance, what happened? What was the atmosphere like? How long did the silence last? And what was the main thought going through your mind when you chose to remain silent?

Interviewee: We certainly have different understandings of silence. To me, silence sometimes acts as a buffer—a way to process things and avoid saying something too harsh. But for her, my silence might be interpreted as punishment or a lack of respect; she might even think I’ve given up on communicating with her altogether. So, often, my original intent differs from the message she actually receives. There was one time we were discussing where to spend Christmas. She wanted to go to Australia because her family is there, and I was actually open to the idea. However, I was incredibly busy with work that year. I had a project on the go, and my parents also wanted us to visit them for the Spring Festival. She asked, “What do you think about going to Australia for Christmas?” I was already feeling a bit annoyed because I knew that a trip to Australia—booking flights, requesting time off, and planning the itinerary—would be a hassle. But instead of saying “I don’t want to go” right away, I just said, “Let’s see when the time comes.” She immediately asked, “What does that mean?” I replied, “It means we’ll decide later.” She felt I was being evasive. I didn’t tell her directly that I wasn’t keen on going because I was afraid she’d think I didn’t want to meet her family. That wasn’t my intention at all; I just felt overwhelmed with things to do and didn’t want to make a decision right then. I also knew that if I simply said “no,” she might perceive it as a rejection of her family. So, I chose not to say anything for the moment. Looking back, that choice to hold off was essentially a form of silence—not a total lack of speech, but rather withholding a clear answer or delaying a definitive response. There was also something that happened recently. I was playing a computer game. It was my day off, and I really wanted to play, but she suddenly complained. She asked why I was spending the whole day at the computer instead of hanging out with her, especially when I finally had a day off. She also pointed out that I wasn’t doing any housework—not even picking up trash on the floor that was right in plain sight. That irritated me a bit, so I snapped back, “I’m only playing all day today, aren’t I?” My tone wasn’t exactly pleasant. We didn’t really speak to each other for the rest of the evening.

Researcher: So, who was the first to break the silence? Interviewee: Me. Since I feel that the issue is largely my fault, I tend to take the initiative to apologize, seek reconciliation, and defuse the tension.

Researcher: In what specific situations are you more inclined to remain silent? For instance, when facing accusations from your partner, discussing sensitive topics, dealing with conflicting cultural views, feeling exhausted, showing defiance, protecting your psychological boundaries, or expressing feelings like grievance, helplessness, sadness, or anger?

Interviewee: What I just mentioned—silence in the face of my partner’s accusations. Is exactly the kind of situation where I go quiet. I feel like I’m already very angry, and if I keep talking, I might lose control, so I stay silent. I’m not the type of person who likes loud arguments. Especially at home, I really dislike it when voices are raised on both sides. So, if she gets emotionally heated, I sometimes suddenly go quiet. This silence is a conscious choice, sometimes even deliberate. But by “deliberate,” I don’t mean I’m intentionally giving her the silent treatment; rather, I realize that whatever I say might be inappropriate at that moment. For example, we once argued about the division of household chores. She felt I wasn’t doing enough, while I thought her standards were too high. She said, “You don’t notice what needs to be done.” I really wanted to push back because I felt I was doing a lot, but I didn’t say anything at the time. I knew that if I said, “I’ve already done so much,” she might think I was keeping score or being defensive. So, I stayed quiet. Later, I washed the dishes, took out the trash, and went back to my room. We do argue quite a bit about the division of chores. How should I put it? I consider myself quite open-minded, but in her eyes, I still have a bit of a “male chauvinist” streak. For instance, when we have guests over, I habitually expect her to prepare tea and snacks while I take the lead in the conversation. She’ll ask, “Why do you always assume I’ll do those things?” I’m often left speechless; I realize that, subconsciously, I’m still influenced by the traditional Chinese idea that “men handle the outside world while women handle the home.” Western culture tends to favor an equal division of chores, whereas Eastern culture often retains traditional gender roles. These cross-cultural differences can easily spark conflict over housework, so I often use silence to avoid direct confrontation.

Researcher: Do you feel that your silence is sometimes a form of compromise? Interviewee: Sometimes it’s a compromise, and sometimes it isn’t. Sometimes it’s simply to avoid conflict, while at other times, it’s actually about setting a boundary. For instance, if she demands an immediate answer to a question while she’s highly emotional, I might say, “I need some time,” and then stop talking. To me, that signals that I’m not in a state to continue the discussion right then. I also tend to go silent when the topic involves parents or family values, as these subjects are quite sensitive. Once, for example, she remarked that Chinese parents sometimes overstep by getting too involved in their adult children’s lives. I had mixed feelings about that. I knew her point wasn’t entirely unfounded, but if she stated it bluntly, my initial reaction was still to feel that she didn’t understand Chinese families. Yet, I didn’t want to immediately push back, because arguing with a partner about whose culture is “right” rarely leads anywhere productive. So, more often than not, I choose to listen.

Researcher: What is the primary reason you choose silence? Is it to avoid an argument, a feeling that things can’t be clearly explained, a desire to maintain harmony, a need to save face (for yourself or the other person), the influence of cultural norms, the pressure of expressing yourself in the language, or simply not knowing how to respond in the moment?

Interviewee: If I had to rank the reasons, the first would be not wanting to escalate the conflict; the second, feeling that some things are difficult to explain in the moment; and the third, the pressure of language. Take the difference in cultural values, for example. In China, we naturally value family harmony and try to accommodate every family member’s feelings. But people from Western countries prioritize individual choice and personal freedom. Because of this clash in mindsets, I usually choose to stay silent to cool down conflicts, rather than destroying our relationship through constant arguing. Of course, I’m speaking from my own perspective; this may not necessarily apply to all Chinese or Western people. However, my upbringing made me prioritize relationship stability over voicing my own opinions in the moment. My wife is more likely to say, “This is what I think, and you can disagree.” She views differing opinions as normal. In contrast, I used to interpret a difference of opinion as a sign of trouble in the relationship. These are two very different mindsets. Also, when we argue, I realize that while my English is sufficient for daily conversation, it isn’t always enough to accurately express complex emotions. Casual chatting is fine, but it’s difficult when I need to explain why I feel offended or why a certain comment made me lose face. I worry about not expressing myself precisely. Sometimes, by the time I finish explaining something in English, the main point has shifted. So, I sometimes just give up. Plus, communicating about daily chores and explaining cultural differences in English day in and day out is mentally and physically exhausting. When a disagreement arises, I often lack the energy to even formulate an argument; I can’t be bothered to explain too much, so I resort to silence—language fatigue drains the patience needed for cross-cultural communication. That’s one reason for my silence. Another reason is the influence of my upbringing. You know the saying, “Silence is golden” and “Trouble comes from the mouth”? While I know these are traditional views, they really do shape one’s behavior. Especially after getting married, I subconsciously feel that a couple shouldn’t fight to determine a winner or loser. It’s better to process my emotions first before trying to solve the problem, to separate problem-solving from the need to prove I’m right. If I feel that continuing an argument would only prove my point without actually improving the relationship, I might just stop arguing. That said, if one person always stays silent while the other speaks, problems can accumulate over time.

Researcher: What impact does your silence have on your communication or relationship? For instance, does it maintain harmony, defuse conflict, escalate tension, leave your partner confused or hurt, provide a cooling-off period, or create lingering resentment? Could you give an example?

Interviewee: If I kept talking while angry, I’d likely say things I’d regret later. So, silence allows both sides to cool down. However, relying on silence to resolve every issue definitely doesn’t work. It can easily lead to the silent treatment, making the atmosphere at home incredibly awkward. My wife dislikes awkwardness, and so do I. It’s okay to stay silent for a bit to let tempers cool, but you shouldn’t let the silence drag on too long; that really can create lingering resentment. Besides, it’s best to be open and honest about your thoughts rather than expecting the other person to guess—guessing is hard, and nobody can read minds, right? I remember once we were discussing buying a house. She felt we should live in Australia eventually because her parents are there and she thought the pace of life was less stressful. But I actually wanted to stay in China for the next few years. We discussed this issue several times. During the first discussion, I hardly said anything. She asked me, “Where do you see us living?” and I replied, “I don’t know.” It wasn’t that I didn’t know; I knew I didn’t want to leave China just yet, but I didn’t know how to articulate it. It was such a huge issue. If I said I wanted to stay in China, she might immediately worry that we’d have to live apart long-term, or think I wasn’t taking her family into account at all. So, I gave a vague answer. Later, she suddenly said, “You never tell me what you really want.” That really struck a chord with me. It made me realize that silence can sometimes make the other person feel insecure; silence can actually damage the relationship. The trickiest part is that you might feel like the issue has passed, but the other person hasn’t moved on. For instance, we once had an argument; by the next day, I was back to normal and thought everything was fine. But she told me, “You seem fine, but I’m still hurt.” That’s when I realized that just because I didn’t mind it anymore didn’t mean she felt the same way.

Researcher: Do you take the initiative to resolve conflicts caused by silence? Do you proactively offer explanations? How do you usually “break the ice” and resume communication after a period of silence?

Interviewee: Yes, we do. And things are much better now than when we first got married. We’ve gradually developed a fairly consistent routine. Usually, if I’m the one who goes silent, I’ll reach out to her after a few hours. Sometimes I don’t dive straight into the problem. I might ask about something trivial first, but it’s actually a signal that I’m ready to reconnect. I’m not really the type to sit down and say, “Right, let’s resolve this conflict.” I prefer to ease the tension first, maybe by having a meal or going for a walk together, and then talk things through gradually. She eventually realized this too, so now she sometimes says, “Okay, let’s take a walk.” We actually find it easier to talk when we’re out walking. Sitting face-to-face feels too formal and a bit awkward.

Researcher: So, do you take the initiative to explain why you’re being silent?

Interviewee: I do now. I didn’t used to. Now I’ll say straight out, “I was quiet because I didn’t want to hurt you,” or “I needed time to think.” That way, at least she knows what my silence means. We even have a simple agreement now: if I need space, I can just say, “I need some time, but I’m not leaving the conversation.” It means I don’t want to continue right this moment, but I’m not avoiding the issue.

Researcher: Do you think the frequency or way you use silence in your cross-cultural marriage has changed? How does it compare to the beginning?

Interviewee: The change is quite noticeable. In the beginning, I was silent a lot more because I wasn’t sure what was okay to say and what wasn’t. For instance, early on, she’d ask, “Do you like this?” If I didn’t like it, I wouldn’t say “I don’t like it” directly; instead, I’d say, “It’s okay.” She’d get really confused and ask, “What does ‘okay’ mean?” I’d say, “It’s... alright.” She’d press, “Do you like it or not?” and I’d admit, “Not really.” She’d laugh and ask, “Why didn’t you just say that?” At the time, I thought it was a simple question, but I realized later it wasn’t. In a Chinese context, phrases like “it’s okay” or “it’s alright” don’t always mean what they literally say. They might imply, “I don’t really like it, but I don’t want to embarrass you.” I used vague expressions instead of direct ones. It’s a form of partial silence. I wasn’t staying completely quiet, but I wasn’t voicing my true feelings. This was especially common right after we got married. Now, I’m more direct. If I don’t like something, I’ll say, “I don’t really like it, but if you like it, that’s okay.” That way, at least I’ve expressed my thoughts. I do this because I believe sincere expression of one’s true feelings is vital in a marriage, and also because we’ve shared so many experiences by now. Early on, I was afraid of saying the wrong thing. Now, I know she won’t stop loving me just because I disagree with her. That sense of security is crucial. In the past, I stayed silent because I was afraid of conflict. Now, if I fall silent, it’s usually to sort out my emotions. My emotions have stabilized; I don’t get overly emotional anymore, and I can articulate things clearly and quickly. I now consciously choose when to speak and when not to.

For instance, if she’s just come home from work exhausted and I notice she’s already in a bad mood, I probably won’t bring up a particularly sensitive topic right away. I’ll wait until the next day. But I keep the matter in mind. I don’t pretend it never happened.

Researcher: How do you view the changes in power dynamics and the process of cultural integration between a couple as their marriage progresses?

Interviewee: I think they do change. At least for us, the biggest change after getting married was gradually evolving from two individuals with different cultural backgrounds into a single family unit. Early on, I used to think in terms of “this is a Chinese habit” or “this is an Australian habit.” Now, I don’t categorize things so simply. For example, our household has a blend of customs. We make dumplings for Chinese New Year, but she also prepares a dessert common in Australian households. We communicate in a mix of Chinese and English. Even the way we argue varies; sometimes we fight in English, while other times I might suddenly blurt something out in Chinese. Although she doesn’t understand every word of Chinese, she can tell from my tone whether I’m genuinely angry or just joking.

Researcher: So, who do you think holds more say in the family?

Interviewee: I think it’s quite balanced now. But that wasn’t the case at the beginning. Since English is her native language, she naturally had the upper hand in situations requiring quick expression. I sometimes needed a moment to formulate my thoughts.

Especially during arguments, she could say a lot all at once, whereas I had plenty of ideas but couldn’t articulate them quickly enough in English. So, sometimes I’d just stop talking. If one person is constantly silent, the other might get into the habit of making decisions for them. That’s something I became wary of later on. For example, when deciding where to go on weekends, she would usually make a suggestion, and I’d say, “Whatever works for you.” Over time, she genuinely came to believe that I was truly okay with anything.

But that wasn’t the case. Once, she planned an activity I absolutely disliked, and I suddenly told her, “Actually, I don’t want to go.” She was completely surprised. She said, “But you always say it’s fine.” I replied, “I know. That’s my problem.” This made me realize that I couldn’t equate staying silent with keeping the peace; in the long run, the other person wouldn’t know who I really am or what I’m actually thinking.

Researcher: So, does your silence sometimes carry an undertone of resistance or refusal?

Interviewee: Yes, though not in a particularly overt way. For instance, there was a time she kept wanting me to attend an event organized by a friend of hers. I didn’t really want to go because I was exhausted from work. She kept asking, and I kept putting her off. Eventually, she asked, “So you’re not coming?” and I said, “I don’t think so.” In reality, my earlier silence was already expressing my reluctance. She just didn’t pick up on it. That kind of silence is essentially a way of stating one’s position. I’ve come to realize this is a fairly common communication style in Chinese culture; silence can sometimes serve as a wordless refusal or rejection. However, this approach isn’t necessarily effective in a cross-cultural marriage, because the other person might genuinely not understand. She might wonder, “Why didn’t you just say no?” So, I try to avoid using that method now.

Researcher: Do you feel you’ve developed a communication style that sits somewhere between the Chinese and Australian ways? Is there a difference in the frequency or nature of your silence now compared to when you first got married?

Interviewee: Yes. I feel like I don’t operate entirely according to Chinese norms, nor entirely according to hers. For example, I used to think conflict should be avoided at all costs. Now, I don’t find conflict itself all that frightening; what’s truly scary is failing to address it. That said, I still dislike heated confrontations. My approach now is to pause, letting emotions settle on both sides before bringing up the issue and discussing a solution. We used to discuss important matters when we were exhausted from work, which easily led to arguments or silence. Now, we save those conversations for the weekend and make a point of listening fully before responding; communication is more efficient, and there’s less avoidance through silence. This is a style we’ve developed together. We’re actually changing each other. Another shift is that I’ve come to acknowledge silence as a form of communication. I used to think that if I didn’t speak, I wasn’t communicating anything. Now I realize that silence conveys a message, too. The problem is, the other person might not interpret that message correctly. So, if I do use silence, I need to explain it clearly. That’s how I see it now. For instance, if I’m in a bad mood today and don’t say a word, she might wonder if she did something wrong. So now, I’ll say, “I’m just tired today. It’s not about you.” It’s a simple phrase, but it’s something I wouldn’t have said in the past.

Researcher: What positive impacts do you think this change has had on your marriage?

Interviewee: The most immediate change is that the atmosphere at home is much more relaxed. Before, whenever we had a conflict, we’d give each other the silent treatment; the air in the living room felt stifling, even breathing felt heavy. Eating or watching TV together felt awkward, and I’d end the day feeling pent-up and frustrated. Now, if a conflict arises, we call a “time-out” first. Once we’ve calmed down, we talk it through properly. We can usually sort things out the same day without having to go to bed angry, which makes daily life much more comfortable. There are fewer misunderstandings, too, and a greater sense of security. In the past, whenever I went silent, she’d let her imagination run wild, worrying that I’d fallen out of love or had other plans. When she got agitated, I’d think she was being unreasonable, and the more I dwelled on it, the more aggrieved I felt. Now, we lay everything out on the table; we know exactly what the other person is thinking. We don’t have to live by guesswork or dredge up old grievances over trivial matters, and our relationship has actually become more stable. Before, we’d gloss over a lot of issues—things looked fine on the surface, but the resentment was buried deep. Once it piled up, even a tiny incident could trigger an explosion. Now, we bring problems out into the open. Even if we argue a bit, once it’s aired out, it’s truly resolved—no lingering hard feelings. Over time, we’ve come to understand each other better—knowing each other’s boundaries and what matters most. It’s not just about changing how we communicate; it’s about genuinely being able to see things from the other person’s perspective. Looking ahead, this dynamic we’ve forged together gives us more confidence to face the future. Whether it’s changing jobs, having children, or having our aging parents come to live with us, new conflicts are bound to arise. But we know we can solve problems together. Instead of hiding or resorting to the silent treatment, it gives us a real sense of peace of mind.

Researcher: Regarding your tendency toward silence in this cross-cultural marriage, is there anything else, something I haven’t asked about but you feel is important and you’d like to add?

Interviewee: There is one thing I think is quite important: often, silence shouldn’t simply be interpreted as a matter of whether or not someone is willing to communicate. I used to wonder if I was simply too introverted or too avoidant. But later, I realized it wasn’t entirely a matter of personality; it had to do with how I learned to handle relationships growing up. For instance, I was taught not to interrupt others while they were speaking, to listen when elders talked, to avoid arguing with a spouse in front of others, and to refrain from saying things that might cause someone to lose face. All those ingrained habits eventually carry over into your own marriage. However, my wife’s upbringing was likely different. She believes that if you disagree with something, you should speak up. Consequently, we sometimes viewed each other’s behavior as abnormal. She wondered why I wouldn’t say anything, while I wondered why she felt the need to voice everything. We eventually realized that neither approach was the definitive “right answer.” Another point is that silence is sometimes linked to the perceived likelihood of being understood; if I feel I won’t be understood even if I speak, I’m more inclined to stay silent. For instance, regarding issues involving Chinese families, I used to worry she would simply view Chinese parents as controlling. So, whenever she criticized the Chinese family dynamic, I would clam up. Now, however, I tell her, “I understand your perspective, but my own experience was different.” That approach works much better. Silence doesn’t necessarily mean a refusal to communicate; sometimes, it’s just a way of seeking a safer or more appropriate method of communication. Of course, if you remain silent indefinitely without ever returning to the conversation, then it does effectively become a refusal to communicate.

Interviewer: If you could offer one piece of advice to Chinese people entering into cross-cultural marriages, what would it be?

Interviewee: I’d say don’t oversimplify cultural differences, and don’t attribute every problem to culture. Sometimes what seems like a cultural difference is actually just a difference in personality. That said, culture does influence how a person understands love, responsibility, family, and conflict. A Chinese person accustomed to silence might feel they are preserving the relationship, whereas a Western partner might interpret that silence as rejection. The most important thing is to tell your partner, “Just because I’m not speaking right now doesn’t mean I don’t care about you.” At the same time, set a timeframe for yourself, for example, “I need an hour of quiet, but we will talk tonight.” Reflecting on my own experience, the most interesting realization for me was that I used to consider myself a good communicator. I never had issues at work or with friends. But after getting married, I discovered that communication within an intimate relationship is completely different from ordinary social interaction, especially in a cross-cultural marriage. You aren’t just dealing with a specific issue; you are dealing with the families, upbringings, and values that shape each person. So, the key is learning how to communicate, and learning to understand, tolerate, and accept one another.

**I-08: (Female, UK partner, with children, married 4–10 years)**

Researcher: Alright, I have a general idea of the situation now. Looking back at the nearly ten years you’ve been together, from dating to marriage, what do you think were the most obvious impacts of your cross-cultural backgrounds (such as differences in values, mindsets, and communication styles) on your relationship?

Interviewee: Oh, there are so many; things were different in every aspect. Right after we got married, there were new surprises every day. The most obvious difference was definitely communication style. He’s a typical Westerner, straightforward and direct; he says exactly what’s on his mind without ever beating around the bush. If he wanted a specific birthday gift, he’d hand me a list, complete with links, so there was no need for guessing games. I’m the opposite; I grew up used to keeping things to myself. If I was upset, I wouldn’t say so directly; I’d just hope the other person would notice on their own. When I was angry, I wouldn’t say, “I’m mad, come comfort me.” Instead, I’d talk less, use a flat tone, or sit by myself scrolling through my phone, waiting for him to ask what was wrong. But he’d never pick up on it. I’d end up bottling it up until I couldn’t hold it in anymore and finally blurted it out. Only then would he have a “lightbulb moment” and realize, “Oh, so you were upset.” Then there were the differences in values—money being the classic example. He spends whatever he earns; by the end of the month, there’s nothing left. He doesn’t panic even if he maxes out his credit card because he believes money is meant to be spent and living in the moment is what matters most. I’m not like that. My mom taught me from a young age to save money. Having savings gives me peace of mind. I make sure to put a fixed amount aside every month, I compare prices before buying anything, and I absolutely refuse to buy useless things. We clashed quite a bit over this early on; he’d say I was too stingy and made life exhausting, while I’d say he lacked a plan and ask what would happen if an emergency came up. And the differences in parenting philosophies were even greater. His approach is very hands-off. If the kids fall or bump into something, as long as there’s no blood, he lets them pick themselves up, saying it fosters independence. It breaks my heart, though; whenever they fall, I rush over to help them up, but then he accuses me of coddling them. Then there was the issue of schooling: he felt that primary school was just for play—that grades didn’t matter as long as the child was happy, whereas I believed a solid foundation was essential and that learning had to happen. We argued quite a bit over that.

Researcher: When you first discovered these differences, did you find them hard to accept? Or did things improve as you gradually adjusted to each other?

Interviewee: It was definitely hard at first; sometimes it even escalated into questions about whether he loved me. For instance, if he couldn’t guess why I was angry, I’d think, “If he really cared about me, how could he not understand what I’m thinking?” But after living together longer, I realized it wasn’t a lack of love. He simply didn’t have that habit. Growing up, no one ever asked him to guess what others were thinking, so he never learned how. We’ve adjusted much better now, though, and have found compromises for a lot of things.

Researcher: Right. Let’s talk about silence next. Do you ever experience moments of silence in your daily life with your partner or during arguments? If so, could you describe one or two particularly memorable instances? For example, what happened, what the atmosphere was like, how long the silence lasted, and what was going through your mind?

Interviewee: Oh, absolutely. What couple doesn’t have their tiffs? One instance that really sticks in my mind is the time I was cooking several dishes—the kind that generate a lot of smoke and grease. Our kitchen range hood wasn’t very effective to begin with—extractor fans in British homes generally have weak suction, so as I cooked, the smoke built up until the whole kitchen was hazy. He had been in the living room playing with the kids but came over when he smelled the smoke. He stood in the kitchen doorway, frowning, and asked why there was so much smoke, saying the kitchen walls would turn yellow from it over time. He always frowns at the smoke and grease from Chinese cooking and complains that it is unhygienic. I didn’t stop the work at hand and didn’t respond to him. I just turned on the range hood silently. I used silence to resolve his negative opinions on my culinary culture. At the time, I had my back to him and kept turning the ribs in the pan without saying a word. He stood in the doorway for a moment, and seeing that I was ignoring him, he sighed and went back to the living room. I just didn’t want to pay him any mind at that moment.

Researcher: What exactly were you thinking at that moment? Did you feel aggrieved, or did you feel like it was impossible to get through to him?

Interviewee: I guess I felt a bit aggrieved. I’d gone to the trouble of cooking a meal to give the kids something better to eat; not only did he not help, but he just stood there picking faults. Besides, that’s just how Chinese cooking is—you need high heat and quick stir-frying to get that wok hei (breath of the wok) flavor; how could there not be cooking fumes? He eats Western food every day without thinking it’s greasy, yet suddenly my stir-fried dishes are considered unhygienic? I was actually pretty angry inside. I didn’t want to talk to him; I was furious and kind of wanted to vent my frustration on him.

Researcher: I see. So, in what specific situations are you more likely to choose silence? For instance, when facing his accusations, discussing sensitive topics, dealing with cultural clashes, or when you’re tired, aggrieved, or angry?

Interviewee: I go silent in quite a few situations. It’s hard to say which happens most often; they all do. The first is when there’s a clash of cultural values, like when we talk about child-rearing, meals, or family relationships. Whenever our cultural differences come up and we can’t see eye to eye, I tend to clam up. There’s no right or wrong answer, and talking about it is pointless, so I’d rather not say anything. The second situation is the cooking incident I mentioned earlier—when he criticizes me or nitpicks. I also go silent when he complains that the house isn’t clean, accuses me of wasting money on shopping, or says I spend too long on the phone with my family. It’s not that I think I’m in the wrong; I just don’t want to argue with him. The more I argue, the more he goes on, so it’s better to just shut up; once he realizes I’m not engaging, he usually stops. I also go silent when I’m exhausted. Sometimes, after a long day at work, I still have to pick up the kids, cook, and tidy the house. I’m so tired I don’t want to speak, let alone start a conversation. It’s not directed at him; I just don’t have the energy to talk.

Researcher: Among these situations, which one makes you go silent the most often? Is there any situation where you absolutely wouldn’t stay silent?

Interviewee: Cultural clashes probably trigger it the most. After all, those issues are the hardest to resolve. I used to go silent because of language barriers too, but I’ve been here for several years now and can handle daily communication just fine, so that happens much less often than when I first arrived. There really aren’t any situations where I absolutely wouldn’t stay silent. Even when I’m clearly in the right, I don’t immediately get into an argument with him; I’ll pause for a moment to think about what to say. If it involves the children’s safety or if he speaks ill of my parents, I’ll certainly push back, but I won’t shout or scream. I’ll still stay quiet for a few seconds to gather my thoughts before speaking. It’s become a conditioned reflex: when something comes up, I shut my mouth first and think about how to phrase things.

Researcher: So, what is the main reason you choose silence? Is it to avoid an argument, a feeling that it’s impossible to explain, a desire to maintain harmony, a wish to save face for both parties, cultural influence, the pressure of expressing yourself in a foreign language, or simply not knowing how to respond in the moment?

Interviewee: It’s actually a mix of all those reasons. Rarely is silence caused by just one thing; usually, several factors combine, and I just lose the desire to speak. The biggest reason is probably wanting to avoid conflict and keep the peace at home. I’ve always felt that harmony is the most important thing in a marriage. What’s the point of arguing every day? Many issues aren’t matters of principle; if both sides take a step back, things blow over. Especially after having kids, I really don’t want to argue in front of them for fear of leaving them with emotional scars. So, whenever I feel my temper rising, I just clam up and wait until everyone has cooled down. There are many cultural nuances that can’t be explained in a few words. A lot of my views were shaped over twenty-plus years of life; no matter how much I explain, if he hasn’t lived through it, he can’t truly understand. He might even think I’m just making excuses. So, often I just don’t bother explaining; silence is easier.

Language plays a role, too. While I can handle daily communication fine, it’s hard to articulate complex emotions or subtle feelings in English. For instance, if I’m feeling aggrieved, heartbroken, or resigned—emotions I could express clearly in Chinese in an instant. I struggle to convey that complexity in English. If I keep it superficial, he doesn’t get it; if I try to go deeper, I lack the words, so I just give up. I know many Chinese people who have lived here for twenty or thirty years and speak English as fluently as the locals, yet they remain reserved by nature. They aren’t used to speaking directly about their emotions and tend to fall silent first when faced with an issue. That’s something deeply ingrained in our culture. It’s not something that changes just because you learn a new language. Researcher: So, what impact does your silence have on your communication or relationship? Does it, for instance, maintain harmony or defuse conflict? Or does it escalate tensions, leave him confused and hurt, or perhaps provide a cooling-off period while also creating lingering resentment? Could you give an example?

Interviewee: On the positive side, it certainly defuses conflict and gives everyone time to cool down. Sometimes, when emotions run high, a heated exchange can easily lead to hurtful words; staying silent until things calm down makes the issue seem trivial, and the problem just blows over. It also maintains a semblance of peace, allowing many minor issues to be put aside. For example, if he leaves his socks lying around or doesn’t put things back where they belong, I might be annoyed but choose not to say anything; I’ll just stay quiet and tidy up myself. It’s not worth a fight over such small things. It keeps the household atmosphere pleasant rather than chaotic. I don’t think it creates lingering resentment, though, because I usually explain why I was silent. We clear the air pretty quickly. Otherwise, we’d just end up arguing again the next time something similar happens.

Researcher: So, overall, do you feel that your silence does more good than harm to the relationship?

Interviewee: In the short term, it’s definitely beneficial. It avoids arguments and keeps the peace in the moment. But in the long run, if you don’t actively communicate, the problems don’t disappear; they just pile up. Plus, a lack of communication over time creates a rift between the two of you.

Researcher: Does your partner ever misunderstand your silence? How does he usually react to it? Does he ask you what’s wrong, go silent himself, get impatient, or try to draw you out? What do you think of these reactions?

Interviewee: He most often assumes that my silence means I’m angry. To be fair, there are times when I am angry and don’t want to talk to him, sometimes even using the silent treatment to punish him or show my displeasure. But other times, I might just be tired, deep in thought, or simply not in the mood to speak; in those cases, I’ll actually tell him why I’m quiet. There are also times when I’m silent because I’m waiting for him to comfort me, but he thinks I’m ignoring him and just walks away, which makes me even angrier. He simply doesn’t grasp the meaning behind my silence. Back when we were first dating or newly married, he was incredibly patient, but as time went on, that patience wore thin. Usually, he’d ask a couple of questions; if I still didn’t speak, he’d get a bit impatient and say, “You’re always like this. Can’t you just say what’s on your mind?” and then he’d get up and walk away. Other times, if I stayed silent for too long, he’d go silent too, and we’d end up in a standoff. He’d think, “If you don’t want to talk, I won’t either. Let’s see who cracks first.” But usually, he’s the one who can’t hold out; after all, there are kids in the house, so someone has to speak eventually.

Researcher: Has he ever had a serious conversation with you about how he actually feels when you go silent?

Interviewee: Yes, he has. After a three-day standoff we had a while back, he opened up quite a bit once we made up. He said that whenever I clam up, he’d actually prefer I have a huge argument with him—yelling out all my grievances—rather than staying silent and bottling it all up. He just feels helpless and anxious. He also mentioned that sometimes he doubts himself, wondering if he’s done something wrong or isn’t good enough, which makes me unwilling to share anything with him. During the first six months of our marriage, I was silent a lot; he even asked if I regretted coming to the UK or if I didn’t want to be with him anymore.

Researcher: I see. After a period of silence like that, how do you usually break the ice and start talking again? Does one of you speak first, is there physical contact, or do you transition back naturally through everyday matters?

Interviewee: If he initiates the conversation, I’ll definitely respond. He often uses the kids as a way to break the silence. For example, he might suddenly call out to a child, “Come here, let Daddy give you a hug,” or ask, “Who’s taking the kid to school tomorrow?” He acts as if nothing happened, using the children to open the conversation. When he does that, I usually take the opportunity to step down and answer him, and things gradually return to normal. After all, with the kids around, we can’t stay in a standoff forever; we have to talk. So, since having children, the periods of silence have become shorter, a day or two at most; they don’t last long. There’s physical contact, too, usually when the tension is minor, like if I’m just in a bit of a mood. He might come over and touch my arm or give me a gentle hug from behind; we don’t need to say a word—just a hug, and we’re okay again. But if it’s a major conflict, we wouldn’t handle it that way. I mean, if I’m that angry, would he even dare to touch me?

Researcher: Are there times when the silence lasts a particularly long time? Like, for several days?

Interviewee: The longest instance was right after we got married; we gave each other the silent treatment for three days. We didn’t have kids back then, so we just dug in our heels—doing our own things, cooking our own meals, and sleeping back-to-back in the same bed without speaking a word. I actually regretted it by the second day, but I was too proud to be the first to speak, so I just held out. Eventually, he bought a cake I liked and said, “Don’t be mad anymore,” so I took the opportunity to let it go. Looking back, it seems pretty childish. We were both miserable, all just to prove a point. Now that we have a child, a long silent treatment is impossible; the kid acts as a go-between, calling out for Mom and then Dad, so we naturally end up talking to each other.

Researcher: After the ice is broken, do you go back and discuss the original conflict? Or do you just move on and never mention it again?

Interviewee: We do bring it up. We wait until we’re both in a good mood and have calmed down, then sit down and talk about it. For instance, in the evening after the child is asleep and we’re sitting on the sofa watching TV, he’ll gently bring it up, share his perspective, and ask for mine. He’s usually the one to initiate that kind of review; I rarely do it myself. I tend to feel that once something is over, it’s over, and bringing it up again might just lead to more unpleasantness. But he believes that talking things through prevents lingering resentment and stops us from fighting over the same issue next time. After all these years, I agree with him—talking it out actually makes me feel more at ease.

Researcher: Do you explain the reasons for your silence to him afterwards?

Interviewee: Yes, I do. If he asks about it, I’ll certainly explain.

Researcher: Do you think those explanations help? How does he react when he hears them?

Interviewee: They definitely help. He always looks visibly relieved after I explain. Still, he wishes I would just express my feelings in the moment. That would be better.

Researcher: I see. Let’s talk about this from a cultural perspective now. Do you think certain concepts in Chinese culture, such as “harmony in the family leads to prosperity in all undertakings,” “think thrice before acting,” “silence is golden,” or “loose lips lead to mistakes,” or perhaps your upbringing, influence your choice to remain silent in your cross-cultural marriage?

Interviewee: I think they do have some influence, more or less. Westerners don’t seem to hold these views; they really don’t have these concepts. They place more emphasis on verbal communication and expression, believing it’s better to speak your mind. However, I grew up receiving a Chinese education, so I was taught to be mindful of my words and actions. That said, I think I’ve changed a bit since coming to the UK a few years ago; I’m not as inhibited as I used to be, and I’ve started speaking my mind more openly.

Researcher: So, do you think there has been a change in the frequency or manner in which you use silence in your cross-cultural marriage? How does the situation now compare to when you first started living together?

Interviewee: During my first two years in the UK, I wasn’t very familiar with the environment or many other things, so that was when I was silent the most. Now, I definitely use silence much less often; as I mentioned earlier, I feel I’m gradually starting to express my own thoughts. Also, when living in a foreign country, you have to learn the local culture and try to integrate. You also have to learn about your husband’s, and even his entire family’s—values and perspectives. It felt a bit jarring at first, but over time, I’ve slowly integrated.

Researcher: Do you think this integration came about through your own conscious adjustments, or did it develop as the two of you gradually adapted to each other?

Interviewee: It was definitely a process of mutual adaptation; it certainly couldn’t have happened through my efforts alone. When two people live together for a long time, they develop a certain rapport. Whether it’s communication, daily habits, or mindsets, it’s really about learning from each other’s strengths and weaknesses to minimize conflict and friction.

**I-09: (Female, UK partner, without children, married < 3 years)**

Researcher: Alright, looking back at your time together, what do you think were the most significant impacts of your differing cultural backgrounds, such as values, mindsets, and communication styles, on your intimate relationship?

Interviewee: The impact was quite comprehensive; you could say it permeated every aspect of our lives. The most obvious difference was between direct and indirect communication. He was raised to speak up immediately if there was a problem, focusing on the issue rather than the person; he felt comfortable openly disagreeing, even in front of friends. Growing up, my family rarely spoke harshly to one another face-to-face, and even when my parents argued, it never involved loud, explosive shouting. So, when I encountered conflict, my instinct was to stay silent and suppress my emotions first. At first, he perceived this silence as coldness or a refusal to communicate, while I felt he was being too aggressive, sometimes saying things that made me lose face. He also felt I cared too much about my parents’ opinions; for instance, when we were making decisions, I’d say, “My parents might want us to do X or Y.” He would counter with, “This is our life, not theirs.” That clash of perspectives was very stark. For Chinese people, marriage is often seen as the union of two families, whereas Westerners tend to view it as something more independent and free, a life that belongs to the couple themselves.

Researcher: You mentioned the difference between direct and indirect communication earlier. Could you share a recent, small incident that illustrates this?

Interviewee: A few days ago, I got a new haircut and a new hair color. He bluntly said the color didn’t really suit me. I knew he didn’t mean any harm. He was stating a fact, but I still felt hurt and rejected in that moment. I just murmured “Mmhmm” and didn’t say anything else; the rest of the meal was completely silent. Later, he asked if I was angry, and I said no. In reality, I just didn’t know how to respond in the moment.

Researcher: Okay, the third question gets to the heart of the matter. Do you ever fall silent during your daily interactions or when conflicts arise? If so, could you describe one or two specific instances of silence that left a deep impression on you? For example, what happened, what was the atmosphere like, and how long did the silence last? What was going through your mind when you chose to remain silent?

Interviewee: Yes, it happens more often than I realized. A while back, a British friend of his came to Asia from London and wanted to spend some time in Shanghai. He was really happy at the time; while we were eating, he just told me his friend could stay at our place for two weeks. I wasn’t keen on the idea. I was swamped with work back then. My project was at a critical stage, and I’d get home around eight or nine every night, too exhausted to even speak. Plus, we live in a small two-bedroom apartment; having someone stay for two weeks meant I’d have to take care of his friend’s meals, and the thought of that stressed me out. But I didn’t say no right then; I just gave a noncommittal “Oh” and kept my head down, focusing on my meal. He probably took that as consent. I was dead silent while doing the dishes that night. Usually, I might hum or chat with him, but that evening, I didn’t make a sound. He came over and asked, “Are you okay?” I said, “I’m fine,” though I was anything but fine. My silence lasted the whole evening, from after dinner until bedtime, a good three hours or more. Aside from the bare minimum, I didn’t say a word. He asked me a couple of times, but I just kept saying it was nothing. Even in bed, I lay with my back to him, scrolling through my phone, my body tense. He tried to hug me a few times, but I didn’t respond. I was really conflicted. On one hand, I didn’t want to come across as stingy or unwelcoming. Chinese culture places a lot of importance on hospitality, especially for a close friend of his, and refusing might make me look like I was disrespecting him. On the other hand, I was genuinely exhausted and didn’t want to shoulder that extra burden. I hoped he’d pick up on my reluctance from my silence, but he completely missed the cues. I was even a bit angry with him at the time, wondering why he couldn’t see I was upset or why he didn’t just suggest, “Never mind, let him stay at a hotel.” But then again, I felt like expecting that was childish.

Researcher: What happened next? How was it resolved?

Interviewee: A week later, as his friend’s arrival drew near, he started discussing room arrangements and what bedding to buy. I suddenly snapped and told him I’d actually been against the idea all along. He was shocked. “Why didn’t you say anything back then?” he asked. “I thought you agreed.” I cried and said, “I was afraid to speak up; I didn’t want you to think I was selfish.” He felt hurt, too, saying that if I’d told him sooner, he could have easily arranged for his friend to stay at a hotel. It wasn’t a big deal. We had a huge argument over it. In the end, his friend stayed at a hotel, but the whole thing left a lingering tension between us. He felt I was deceiving him or being dishonest; I felt he didn’t understand my predicament. It took a long time for that knot to slowly unravel.

Researcher: When you were silent back then, did you hope he would keep pressing you?

Interviewee: Yes. I actually hoped he wouldn’t just let my “I’m fine” brush him off; I wanted him to persist a little, give me a hug, or ask, “Are you really okay? You look unhappy.” But he probably thought that since I said I was fine, he should respect that. It’s a cultural difference, too: he trusts words, whereas I believe the other person should be able to read non-verbal cues.

Researcher: What about the second memorable instance of silence?

Interviewee: The first Spring Festival after we got married. My parents wanted us to go back to my hometown for the holiday, but he wanted to use the break to travel to Southeast Asia. He argued that the Spring Festival was my family’s tradition, not his, and asked why we couldn’t just go back for two or three days and then go on a trip. Hearing that made me feel deeply aggrieved; I felt he didn’t respect my family or understand what the Spring Festival meant to me. But I didn’t know how to explain the feelings of family reunion and filial piety in English. So, I just sat on the sofa clutching a cushion, looking down at my phone, though I wasn’t actually taking anything in. My eyes were getting red, but I held back the tears. He didn’t notice at first, but later he walked over and asked, “What’s wrong? Are you crying?” I shook my head and stayed silent. That silence lasted about half an hour. What I was thinking was: even if I told him, he wouldn’t understand; he’d just think I was being unreasonable, so it was better not to say anything. I felt angry and hurt, but mostly, I felt a sense of powerlessness. I felt caught in the middle—my husband on one side, my parents on the other, and it was agonizing. Plus, I knew that if I tried to explain it in English, my words would be broken and disjointed, which might just annoy him more.

Researcher: How did he react specifically while you were silent?

Interviewee: He sat down next to me, looked at me, and asked a few questions. When he saw I wasn’t speaking, he got a bit anxious; he started looking at his phone, then stood up to get a glass of water from the kitchen and handed it to me. I took it but didn’t say anything. Eventually, he sighed and said, “Okay, I’ll give you some space,” and went to the study. Actually, I felt even worse after he walked away; I felt like he should have kept trying to comfort me. But I also know he’s giving me space, not because he doesn’t care. His way of doing things is just different from what I expect.

Researcher: What physical sensations do you experience when you go silent? How do you behave?

Interviewee: I feel a tightness in my chest, like something is pressing down on it, and my throat constricts. Sometimes my hands get cold, or I keep picking at my fingers or looking at my phone. If I’m really emotional, I feel like crying, but I hold it back because I don’t want to appear overly emotional. Sometimes I’ll hug a cushion; having something to hold makes me feel a bit safer. When I don’t want to speak, I might just “battle” it out with him over WeChat instead.

Researcher: In what specific situations are you more inclined to stay silent? For instance, when facing accusations from your partner, discussing sensitive topics, dealing with cultural clashes, feeling exhausted, or expressing feelings of grievance, helplessness, sadness, or anger?

Interviewee: I think it’s a mix of all those things. It happens when his tone is sharp, like when he’s accusing me of something. For example, if he asks, “Why are you always like this?”—the moment words like “always” or “never” come up, I instinctively shut down. I feel like he’s already labeling me, so explaining myself is useless; it just makes things look worse. It also happens when we discuss sensitive topics, like politics, history, family values, or his views on his parents. Especially when it involves things related to China; my English isn’t good enough, and I’m afraid of saying the wrong thing, so I just stay quiet. I also go silent when I’m exhausted; after a long day at work, if I have to argue in English, I’d rather just be quiet. If I feel aggrieved or sad, and that emotion surges up, I can’t speak either. I might go silent when I’m angry, too, but that kind of silence is colder. I’ll walk away, avoid looking at him, and lock myself in my room. Also, sometimes he makes sweeping generalizations like “You Chinese people always...” I really resent that; explaining the nuance feels too complicated, and I’m afraid it’ll just start an argument.

Researcher: You mentioned him saying things like “You Chinese people...”. What exactly has he said? How did you react at the time?

Interviewee: There was one time he saw a news story and asked, “Why do Chinese people care so much about ‘face’?” That made me uncomfortable; I felt like he was generalizing over a billion people. But I didn’t argue back; I just gave a non-committal “Mmhmm.” He noticed my expression change and asked if he’d said something wrong. I said “Nothing,” but deep down, I was actually quite upset. I tend to stay silent on topics like that because opening them up leads to a major debate about values, and since my English isn’t fluent enough to keep up, I’d be at a disadvantage. I knew he didn’t mean any harm, but that kind of generalization made me feel offended. For me, silence is a way to prevent the conflict from escalating.

Researcher: When he speaks to you in a heated tone, is your silence temporary, or can it last for a long time?

Interviewee: If it involves accusations, I might stay silent for anywhere from a few minutes to half an hour right then and there. Then, I’ll retreat to another room to calm down or wait for him to come to me. If he doesn’t initiate contact, it could drag on until the next day. I feel I need time to process the hurt rather than lashing out immediately.

Researcher: What is the primary reason you choose silence? For instance, avoiding arguments, feeling like things can’t be explained clearly, maintaining harmony in the relationship, saving face (yours or his), cultural influences, the pressure of expressing yourself in a foreign language, or simply not knowing how to respond in the moment?

Interviewee: I relate to almost all of those reasons. The most important ones are avoiding arguments and maintaining harmony. I’ve always felt that arguing damages the relationship, so I prefer to just endure it if I can. A second reason is the feeling that things can’t be explained clearly, especially in a cross-cultural context; there are feelings he might not grasp, and trying to explain them could just trigger more problems.

Researcher: Does the pressure of language expression play a role in your silence?

Interviewee: It does, yes. For example, when we argue, he speaks very fast and uses words I’m not familiar with; my brain gets stuck trying to process the emotions and the translation at the same time. Sometimes I know what I want to say, but the meaning gets lost or distorted when I say it in English, which might lead to misunderstandings. So, I simply choose not to speak at first. However, I use body language to show him I’m not angry. Since my English sometimes fails to accurately convey my inner feelings, I rely on gestures, eye contact, and facial expressions to signal that I’m not upset. Over time, he’s gotten used to this and waits patiently for me to articulate my thoughts. It took a long time to reach this point; at first, he didn’t understand, and the more I used gestures, the more impatient he would get. Sometimes my English fails to capture exactly how I feel, so I’ll use gestures, eye contact, and facial expressions to show him that I’m not angry. Gradually, he gets used to this and patiently waits for me to voice my thoughts. This process took a long time; at first, he didn’t understand, and the more I used gestures, the more impatient he became.

Researcher: Could you give an example of how you use non-verbal cues to convey “I’m not angry”?

Interviewee: Well, there was a time we were upset because he had to cancel a date to work overtime unexpectedly. I wasn’t actually angry, just a bit disappointed, but I didn’t know how to say “disappointed but I don’t blame you” in English. So, I waved my hand a bit, patted his shoulder, and pointed to my head to signal that I was gathering my thoughts. I managed a faint smile, and he looked into my eyes and seemed to understand. Later, he told me he’d learned to read my expressions and gestures; he knew that if I was willing to get close to him or touch him, I wasn’t angry with him. That communicates things faster than words.

Researcher: So, do you feel that silence is an active choice for you, or an automatic reaction?

Interviewee: At first, it was an automatic reaction, like a reflex; the moment emotions flared up, I’d clam up. Later, as I became aware of my silence, I started trying to control it consciously, though it’s still automatic a lot of the time, especially when I’m tired. I stop speaking before I even have time to think about it.

Researcher: What impact does your silence have on your communication or relationship? For instance, does it maintain harmony, defuse conflict, escalate tension, leave your partner confused or hurt, give you both time to cool off, or create lingering resentment? Could you give an example?

Interviewee: Sometimes, silence really does prevent me from saying hurtful things in the heat of the moment and gives us both time to calm down. For example, if he loses his temper and I were to argue back, I might say something really harsh. If I stay silent, he eventually calms down, and we can talk later, which actually works out better. But more often than not, my silence leaves him confused and hurt. He doesn’t understand why I’m not speaking; he feels like I’m refusing to communicate or even punishing him. He once said directly, “When you go quiet, I feel like you’re punishing me.” That really upset me to hear, because I never intended to punish him. Then there was the incident with his friend coming to stay. I stayed silent for about a week, and when I finally exploded, the conflict actually escalated. He felt deceived, and I was left with lingering resentment. The same thing happened during that Spring Festival. I went silent for half an hour; he thought I was so angry I didn’t want to speak to him, but in reality, I was just sad and felt helpless. So, prolonged silence just lets problems pile up; they don’t go away on their own. Researcher: Looking back now, which instance of silence caused the most damage to the relationship?

Interviewee: The time his friend came to stay with us. I bottled everything up for a whole week, so he had no idea what I was really thinking. In the end, he felt I was being “dishonest,” and that perception was more damaging than the actual issue. It took a long time to rebuild trust. That experience made me realize that silence doesn’t always protect a relationship; sometimes, it’s like planting a landmine. I also started reflecting on why I was afraid to speak up. It was likely because I was terrified of conflict.

Researcher: Was there ever a time when you felt silence was positive—that it actually protected the relationship?

Interviewee: There was one time he’d had a really rough day at work; he came home in a terrible mood and snapped at me. I wanted to snap back, but I could see he wasn’t in a good state, so I chose silence instead. I just poured him a glass of water and sat nearby. A little while later, he apologized on his own, admitting he shouldn’t have brought his work frustrations home. I think that silence was a good thing because it prevented work stress from turning into a marital argument. If I had talked back right then, we both might have exploded.

Researcher: Your partner must misunderstand your silence at times, right? How does he usually react? Does he ask what’s wrong, go silent himself, get impatient, or try to draw you out? How do you view these reactions?

Interviewee: Yes, he misunderstood it a lot at first. He’d assume my silence meant I was angry, ignoring him, or even giving him the “silent treatment.” Usually, he’d start by asking, “Are you okay?” or “Did I do something wrong?” If I said, “I’m fine,” he’d wait a bit and then say, “Okay, take your time.” But if it dragged on, he’d get restless, pacing the room or sighing, and say, “I can’t read your mind. You have to talk to me.” Sometimes he’d go silent too; we’d just do our own things, but the atmosphere would be icy. I remember a few times when he deliberately ignored me, and that cold standoff was really painful. I understand his reactions, though; silence wasn’t a common form of communication in the environment where he grew up, so he tends to interpret it as rejection. But I wish he understood that my silence isn’t meant to punish him. It’s mostly just me needing some time for myself. Researcher: Has he ever done anything that made you feel he was gradually coming to understand you?

Interviewee: Yes. He eventually realized that if I was willing to get close to him while remaining silent, like tugging at his shirt, pouring him a glass of water, or gently touching his hand, it meant I wasn’t angry. He would say, “Okay, I know you’re not angry. But we still need to talk later.” That sentence was important to me because it gave me space while also setting an expectation for communication. I think he’s more patient than I am. Plus, he learned to tell whether I was truly angry or just needed time. Once, when I was silent, he sat next to me, held my hand, and said, “I’m here. Take your time.” I relaxed immediately.

Researcher: Does your husband ever get particularly frustrated by your silence?

Interviewee: He does. Especially when he’s in a rush or when the matter at hand is time-sensitive. For instance, if we needed to book flights for a vacation and I stayed silent, he’d get agitated and say, “We have to decide today. Can you give me an answer?” In those moments, I’d feel even more pressured and unable to speak. Later, he learned to bring up important decisions in advance, giving me time to think rather than forcing me to commit on the spot. That was part of us finding a way to work together.

Researcher: You mentioned earlier that you sometimes use non-verbal cues to let him know you aren’t angry; is that related to the pressure of using a foreign language?

Interviewee: Yes, exactly. As I mentioned before, sometimes my English isn’t enough to accurately express my inner feelings, so I use gestures, eye contact, and facial expressions to convey that I’m not angry. Gradually, he got used to this and would patiently wait for me to put my thoughts into words. Once that habit was established, he became less anxious. But I also know that relying on that alone isn’t enough; we still need to articulate the real issues clearly. My English is improving, too.

Researcher: After a period of silence, how do you usually “break the ice” and resume communicating? Does one of you speak up first, use physical contact, or does the conversation naturally transition back through everyday activities?

Interviewee: Most of the time, he’s the one who speaks first, because he finds the silence harder to bear than I do. He might ask, “Do you want to talk now?” or “Can we talk?” Sometimes, if I’m not ready yet, he’ll say, “Okay, maybe after dinner,” giving me a bit of breathing room. Other times, he’ll just come over and hug me; my body softens, and my emotional defenses start to lower. Then there are times he asks something mundane like, “What should we have for dinner?” I know he’s offering me a way to break the tension, so I’ll respond, and we’ll gradually ease back into the real conversation. Sometimes I’m the one to break the ice—usually after I’ve calmed down—by going to the living room to sit next to him or giving him a hug from behind. Occasionally, I’ll send a message in English via WeChat, since writing is easier than speaking, to explain why I had gone silent. He’ll read it, reply, and then we’ll talk face-to-face. Physical contact is crucial for us; words sometimes fall short, but a hug can convey so much.

Researcher: Is there a particular instance of breaking the ice that stands out in your memory?

Interviewee: There was one time we spent the whole afternoon in silence over the division of household chores. That evening, I was lying in the bedroom when he knocked and came in carrying a glass of hot milk; he set it on the nightstand without saying a word. I looked at him, and he sat on the edge of the bed and said, “I hate fighting with you.” I burst into tears, and we hugged each other. I apologized later, and he admitted he needed to change, too. I still remember that glass of milk. Sometimes, breaking the ice doesn’t require grand reasoning; a small gesture is enough.

Researcher: Have you ever tried using pen and paper or WeChat messages to break the ice?

Interviewee: Yes, especially when the issue is complex. Typing allows me to be more organized. I can think things through before hitting send. He replies thoughtfully, too. We’ve actually had some important conversations over WeChat that went more smoothly than face-to-face talks. When we’re face-to-face, getting emotional makes it easy to get tongue-tied or start crying.

Researcher: Do you explain the reason for your silence to your partner afterward, saying something like, “I wasn’t speaking because I needed to calm down, not to give you the silent treatment”? Does that kind of explanation help reduce misunderstandings?

Interviewee: I do, and I’ve found it’s essential to explain. Usually, a few hours later or the next day. Once my emotions have settled, I’ll tell him, “I wasn’t speaking because I needed to calm down; it wasn’t the silent treatment, nor was I trying to ignore you.” That kind of explanation really helps him understand. He would breathe a sigh of relief after hearing that and say, “Next time, just tell me straight away that you need time, and then I won’t panic.” We even agreed on a signal: if I say “I need a moment,” he knows I’m not refusing to communicate. But sometimes I still forget to say it in the heat of the moment, because silence is an automatic reaction for me, not a conscious choice. Explaining things doesn’t eliminate misunderstandings entirely, but it certainly reduces them. At least he knows I’m not trying to punish him on purpose. However, he has said he wishes I would shorten the duration of my silence; even just saying “give me ten minutes” is better than total silence.

Researcher: Do you find the act of explaining difficult?

Interviewee: It is a bit difficult, because explaining requires me to formulate my thoughts in English. If something has just happened and I’m still emotional, explaining becomes an added source of stress. So, I’ll often type out a message on WeChat, edit it, and then send it to him. That way, I can express myself more clearly. Sometimes I write it in Chinese first and then translate it into English; it’s slower, but more accurate.

Researcher: Have there been times when you explained yourself, but he still didn’t understand?

Interviewee: Yes. For instance, during the Spring Festival. I explained at length later that it wasn’t that I didn’t want to travel, but rather that I felt I should be with my parents during the holiday. He still couldn’t fully grasp why going home was a must or why we couldn’t just go at a different time. He said he respected my choice, but he didn’t understand the logic behind it. In situations like that, explaining can only mitigate the misunderstanding; it can’t completely bridge the cultural gap. We can only reach a compromise rather than achieve full mutual understanding.

Researcher: From a cultural perspective, do you think certain concepts in Chinese culture, such as “harmony in the family leads to prosperity,” “think thrice before acting,” “silence is golden,” “loose lips cause trouble,” or “be cautious in speech and action,” or perhaps your upbringing, influence your choice to remain silent in an intercultural romantic relationship?

Interviewee: The influence is huge. I was raised on the idea that “harmony in the family leads to prosperity”—that there shouldn’t be arguments at home, and that arguing is a bad thing. My mother would often say, “Just endure it and it will pass,” or “Don’t say everything that’s on your mind.” I heard phrases like “think thrice before acting,” “silence is golden,” “loose lips cause trouble,” and “be cautious in speech and action” constantly while growing up. My grandmother, in particular, used to say, “Girls shouldn’t talk too much.” These ideas conditioned me to stay quiet during conflicts; I felt that saying too much might hurt our relationship or make me appear immature or unvirtuous. There was also the cultural taboo against airing “dirty laundry” in public, so I was reluctant to discuss our issues with friends, which meant I internalized all the stress. Growing up, my parents rarely had loud arguments, but they engaged in the “silent treatment.” I remember my mother would stop speaking when angry, and my father would wear a stern, unsmiling face; the silence in the house was oppressive. I likely picked up this pattern unconsciously, viewing silence as the default way to handle conflict. However, in my husband’s family, they argue openly but will have coffee together afterward. They don’t do the silent treatment. So, we have completely different understandings of silence.

Researcher: Do you think the idea that “silence is golden” helps or hinders in a cross-cultural marriage?

Interviewee: It’s a bit of both. Sometimes silence helps by preventing angry words. But if used too much, it becomes a hindrance. He doesn’t subscribe to the “silence is golden” philosophy; to him, silence signals a problem. If I stay silent, he assumes something is wrong with our marriage. So now, I use it less often, or at least I try to give him some kind of signal. I think “thinking before you act” is good, but you have to actually act after thinking; you can’t just keep thinking forever without ever following through.

Researcher: You mentioned your parents’ silent treatment; is there a specific memory that stands out to you?

Interviewee: Yes. When I was a child, my parents had a falling out over something. I don’t remember the specific issue, just that they didn’t speak to each other for days. Mealtimes were dead silent; I didn’t even dare make a sound while picking up food with my chopsticks. My mother would slam doors, but she simply wouldn’t speak. I was terrified back then, wondering if I had done something wrong. I’ve forgotten how they eventually made up, but that icy atmosphere has stayed with me. So now, when I go silent, I sometimes catch myself wondering if I’m repeating my mother’s behavior. That’s one reason I want to change. I don’t want my husband to experience the kind of fear I felt as a child.

Researcher: Do you think the frequency or way you use silence in your cross-cultural marriage has changed? How does it compare to the beginning?

Interviewee: Yes, quite a bit. In the beginning, I used silence more frequently and for longer periods. I often wanted him to guess what was wrong, believing that if he loved me, he would understand. I realize now that it’s unrealistic and unfair to him. So, I try to shorten the periods of silence; I used to clam up for an entire evening, but now I try to keep it under half an hour. I also make a point of saying “I need a little time” before or during the silence, or I use non-verbal signals to let him know I’m not angry. He’s adjusting too. He doesn’t pressure me to speak constantly anymore and gives me space, though he does insist that we talk about things eventually. The frequency has dropped because we’ve made some agreements. For instance, if there’s something important to discuss, he’ll say beforehand, “We need to talk tonight,” which gives me time to prepare what to say and get into the right mindset. That has reduced the amount of time I spend in silence.

Researcher: What brought about this change?

Interviewee: A few major conflicts made me realize that my silence was hurting him and our relationship. Especially after the incident where his friends came to stay with us, he told me very seriously that he didn’t know how the marriage could continue if I didn’t communicate. That scared me because I really care about this relationship. So, I started making changes. Also, my English has improved, so I feel more confident expressing myself. I’ve also read books on communication and realized that silence doesn’t always mean staying calm; sometimes, it’s a form of avoidance.

Researcher: Has your husband changed as well?

Interviewee: He has. He used to hound me with questions, but now he gives me some space while letting me know he’s nearby and ready to talk whenever I am. He’s also more mindful of his tone and avoids using absolute language. We’re really adjusting together. When I go silent, he observes my facial expressions and body language instead of jumping to the conclusion that I’m angry. That makes me feel respected.

Researcher: If you feel the urge to go silent now, what do you do?

Interviewee: I try to tell him, “I need a little time,” and then go to another room or out onto the balcony for a while. If I really can’t bring myself to speak, I’ll send him a text or give his arm a tap. I’m also practicing direct communication; it’s still hard, but I’m doing better than before.

Researcher: Alright, one last question. Is there anything else regarding your silence within a cross-cultural marriage, something we haven’t asked about but you feel is important and you’d like to add?

Interviewee: I’d like to add that in a marriage between a Westerner and someone from a different culture, silence isn’t necessarily a sign of weakness or “cold violence.” It could be a form of reflection, a way of protecting oneself, or perhaps just a matter of habit. We shouldn’t view silence merely as a problem; we need to consider the function behind it. The key, however, is for both partners to understand each other’s silence. For me, non-verbal cues and subsequent explanations are crucial. If there is only silence, with no other action, the other person suffers greatly. Furthermore, I don’t see silence as a one-sided issue; both partners need to make adjustments. He needs to learn not to pressure me when I am silent, yet still show he cares and let me know he is there. I hope your research focuses on the function of silence rather than simply equating it with a communication breakdown. In cross-cultural marriages, silence can sometimes serve as an emotional buffer rather than an obstacle.

**I-10: (Female, UK partner, with children, married > 10 years)**

Researcher: What do you think are the most noticeable impacts that the cross-cultural background between China and the UK, such as differences in values, mindsets, and daily communication habits, has had on your intimate relationship?

Interviewee: There’s a lot to talk about there. At first, the differences were mostly about daily life, so they didn’t feel like a huge shock. Take meals, for instance: he’d always have cold milk and toast for breakfast, whereas I needed hot porridge or soy milk; otherwise, my stomach would feel uncomfortable all day. Then there were our daily routines. He liked staying up late, watching sports or playing video games until 11 or 12 at night, while I wanted to be in bed by ten. These were minor things we could easily accommodate; now he’ll have millet porridge with me, and I’ll occasionally stay up past midnight to watch a Premier League match with him. The things that really had a deep impact and were hard to reconcile were the underlying conceptual differences. Things like how we define “family,” how we understand “responsibility,” and our sense of personal boundaries. These are values ingrained in us from childhood and are very hard to shift. For example, he believes that even after marriage, two people remain independent individuals with their own social circles and hobbies, so they don’t need to be joined at the hip for everything. I, on the other hand, feel that marriage makes us a single family unit; we should approach things as a family, discuss decisions together, and not just look out for ourselves. Our attitudes toward spending differed greatly, too. He’s the type who lives in the moment, spending whatever he earns, buying things he likes without hesitation, and never making a conscious effort to save. I’m always thinking about saving money, setting aside funds for the children’s education and medical reserves for our aging parents, plus keeping money for emergencies and the mortgage. We clashed quite a bit over saving money early on, but we eventually worked out a system: we manage our own salaries separately and split fixed household expenses down the middle. He also voluntarily sets aside a portion for the kids’ tuition, so things have finally stabilized.

Then there are social customs and the nuances of interpersonal relationships; he just doesn’t get the Chinese way of handling social obligations. For instance, when a relative’s child gets married and we have to give a cash gift, he finds it really strange. He asks why we should give money to someone for no apparent reason. It’s the same with dining out with friends: he thinks splitting the bill is the only logical way, whereas I feel that taking turns to treat each other—reciprocity—is the normal way to socialize. These things might seem trivial, but when they crop up every now and then, and happen frequently enough. They become exhausting. It starts to feel like the two of you are never on the same wavelength.

Researcher: You mentioned the difference between prioritizing the individual and prioritizing the family—a fundamental divergence between Chinese and Western cultures. Could you elaborate on how this manifests in your daily life together?

Interviewee: Oh, there are so many examples; I run into this constantly. Take weekends, for instance: he often wants to go hiking alone or hit a bar with his expat colleagues to watch a game. He views that as his personal time, something he’s entitled to. I, on the other hand, feel weekends should be for family time, taking the kids to the park or the library, or cooking together at home. At first, I’d get upset, feeling he was neglecting the family, while he thought I was being unreasonable, arguing that even married people need personal space. It’s even more apparent with work. If he’s unhappy or bored with his job, he’ll just quit, without giving a second thought to the mortgage or our daily expenses. He always says you can find another job, but you shouldn’t spend your whole life compromising your own happiness. I could never do that. When I choose a job, stability and the ability to balance work with family needs, like school drop-offs and pickups, come first; personal preferences usually take a back seat. For me, family always comes first, and personal interests have to wait. Oh, and then there’s the children’s education—that’s where we disagree the most. He believes a child’s happiness is paramount; grades don’t matter as much as being able to pursue what they love later in life. I argue that the educational environment back home is different. Everyone is racing forward, and if you don’t run, you fall behind. If they don’t build a solid foundation early on, they won’t get into good middle or high schools, and their future options will narrow significantly. Neither of us has been able to convince the other on this point.

Researcher: I see; I have a general idea now. I’d like to ask about your interactions or times when conflict arises: do you ever go silent? If so, could you describe one or two specific instances where the silence was particularly memorable? For example, what happened, where were you, what was the atmosphere like, how long did the silence last, and what was going through your mind when you chose to stay silent?

Interviewee: Oh, definitely, and it happens quite often. It was probably the summer break between my child’s second and third grades, two years ago. One night, past ten o’clock, after the child had gone to sleep, the two of us were sitting on the living room sofa discussing plans for the summer. I wanted to sign him up for “bridge” classes in math and English, plus a calligraphy course; almost all his classmates were doing the same, and I was worried he wouldn’t be able to keep up once the third-grade curriculum got harder. He strongly objected the moment he heard the idea, arguing that summer vacation was meant for play—that it was pitiful for a child to have to attend classes every day after finally getting a break. He said that when he was young, he spent his summers playing soccer outside and never took extra tutoring classes, yet he had turned out just fine. I tried to explain that the educational environments in China and the UK are different: the pressure to advance academically is immense, and since everyone else is studying, you get left behind if you don’t. If we didn’t stay on top of things then, falling behind at one stage would mean falling behind at every subsequent stage. We grew increasingly heated as the conversation went on, our voices rising. He accused me of being overly anxious and projecting my own life expectations onto the child; I accused him of being out of touch with reality and failing to grasp the fierce competition in China. Midway through, I suddenly felt utterly exhausted, like a balloon losing its air, and simply didn’t want to talk anymore. I leaned back against the sofa and started scrolling aimlessly through my phone; I wasn’t actually absorbing any of the content. I just wanted to stop arguing with him. The only thing on in the living room was the TV, playing a replay of a soccer match he hadn’t finished watching earlier. The volume was low, yet the room felt incredibly quiet. At first, he kept talking at length, but when he saw I had gone silent, he stopped and asked, “Why aren’t you saying anything?” I ignored him. He made a few more points, but seeing that I still wasn’t reacting, he finally fell silent. I remained quiet for over an hour, just sitting there scrolling—my fingers moving mechanically back and forth while my mind was in turmoil. I couldn’t tell if I felt more anger or a sense of grievance; mostly, I just felt a profound sense of powerlessness. I knew he had the child’s best interests at heart, but having never grown up or navigated the exam system here, he couldn’t truly understand that kind of pressure. Rather than arguing until things turned ugly and we fell out completely, it was better to just stop talking. At least that way, the conflict wouldn’t escalate any further. Eventually, he was the one to stand up first; he went to the fridge, grabbed two bottles of ice water, and handed one to me. I took it, but I still didn’t say a word. After a while, his tone softened, and he said, “Let’s discuss this slowly; there’s no rush.” Only then did I give a soft “mm-hmm,” effectively ending the standoff.

Researcher: That silence lasted over an hour. It must have been agonizing in such a tense atmosphere, right? Did the thought “If only he understood me” cross your mind at any point?

Interviewee: Oh, absolutely. In that moment, I felt a real sense of grievance. I felt like all the hard work I’d put into planning was for the sake of our family and our child, yet not only did he fail to understand, he actually turned around and accused me of being anxious. But then I thought about it from another angle: he grew up in the British education system and never experienced the cutthroat, “thousands-crossing-a-single-plank-bridge” pressure of the Chinese system, so it was only natural that he wouldn’t get it. Arguing further wouldn’t settle who was right or wrong. It would only hurt our relationship, so I just swallowed my words.

Researcher: I understand. Do you have any other specific examples?

Interviewee: There was another time when he suddenly told me he was bored with the job he’d held for five years. He wanted to quit, take a gap year, and drive the whole family across Europe, starting from the UK, driving all the way to Spain and Italy, and spending six months or more traveling before looking for work again. I was chopping vegetables when he said it, and my knife slowed down immediately. My first reaction was that it was completely unrealistic. The child had to go to school; we couldn’t just have them take a six-month hiatus, could we? We had a monthly mortgage to pay, and our aging parents on both sides needed us. What if they got sick or had a health scare? Who would be there to help? I told him it was too risky and that the child’s schooling couldn’t be disrupted. He argued that we could arrange a leave of absence for the child, claiming that travel itself is the best form of education, offering far more than rote learning in a classroom. He also said we could cover the mortgage with our savings for the time being and that he could find a job anytime upon our return; money could always be earned, but our youth was fleeting, and we shouldn’t let work and family tie us down forever. That was when he said it: “You should live for yourself first; you can’t spend your whole life revolving around other people.” He always says that people should live for themselves first, which completely goes against my family-first belief. But I’ve never argued with him about it. It’s hard to bridge the ideological gap between Chinese and British cultures anyway, so choosing not to poke at it through silence maintains our domestic harmony far better than trying to prove who’s right. After hearing this sentence, I was completely speechless. I put the chopped vegetables on the plate and stood by the stove, watching the oil in the pot slowly heat up without saying a word. He was still planning the route with great interest, saying which church he wanted to see and which old restaurant he wanted to eat paella in. Later, when he saw that I didn’t respond, he came over and touched my arm and asked, “What do you think? Let’s talk.” I still didn’t say anything, so I picked up the spatula and started cooking. The oil splashed and crackled, which just covered his voice. That meal was very quiet, and I hardly spoke actively. When he asked me anything, I just hummed or shook my head. From cooking to cleaning up the kitchen after dinner, for more than an hour, I didn’t communicate much with him. In fact, it’s not that I didn’t talk at all. I just shut up and avoided talking about the topic of resigning during the trip. That night, I lay in bed tossing and turning and couldn’t sleep. I’m not angry that he has this idea; I just feel that after twelve years together, how come we still can’t think of getting together when it comes to priorities in life? I also want to go out and play, and I also want to be relaxed and not care about anything, but there are seniors and juniors above me, and responsibilities on my shoulders. How can I just leave? But I knew it was useless to tell him this. In his view, parents have their own lives, and children have their future. People must first be responsible for themselves. If I argue with him, it will only appear that I am pedantic and constrain him, and in the end we will end up unhappy. So I just stopped talking. If I did, it would just be a quarrel, which would be boring.

Researcher: So the silence at that time was more about the powerlessness and helplessness of knowing that you couldn’t convince the other party, right?

Interviewee: Yes, it’s just powerless. You clearly know that neither person’s intentions are malicious, but they just can’t get along. And this difference is not anyone’s fault; it’s just different cultures and different growth environments. You can’t decide who is right and who is wrong, and you can’t force the other person to change, so you can only hold it in and suppress the matter temporarily with silence.

Researcher: Next, I would like to ask you, in which specific situations do you usually prefer to remain silent? For example, when you face accusations from your partner, discuss sensitive topics, conflict with cultural concepts, feel tired, or have feelings of grievance or anger?

Interviewee: Hmm... Let me think about it. There are probably several situations. The first, and most common, is when it involves a fundamental conflict of cultural concepts. For example, the priorities of individuals and families mentioned just now, as well as fundamental issues such as supporting the elderly, dealing with relationships, and educating children, are difficult to reach an agreement on, and it is useless to talk about them, so I choose to remain silent most of the time. For example, whose home to go to during the Chinese New Year, and how much living expenses to give to both parents, I seldom argue with him about these things now. I either make arrangements myself and then tell him, or I just go along with what he wants. I don’t want to waste my time, and there is no point in arguing. There are also times when two people quarrel and get extremely emotional. I’m not very good at quarreling, and I can’t say unpleasant things, but when I get emotional, I’m afraid that I’ll say a lot of unpleasant things, and I won’t be able to take back the hurtful words I say. So at this time, I will shut up, be silent for a while, and wait until I calm down before speaking. For example, sometimes if he says something that makes me particularly angry, I will not retort on the spot. I will go to the balcony and stand for a while, or go to the bedroom to lie down for a while, and then come out when the anger is almost gone. And when it comes to family members on both sides. For example, when my mother-in-law comes to live with her, I don’t like some of her living habits. I won’t point them out to her face, nor will I complain too much to my husband, for fear that he will be caught in the middle. Also, if there is anything going on with my parents’ family, such as my brother’s need for working capital, I won’t discuss it too much with him, for fear that he will think our family has too many problems and is troublesome. This kind of matter involves family members on both sides. If you talk too much, it will easily lead to resentment. It is better to keep silent and handle it properly by yourself. I definitely don’t want to talk when I’m extremely tired. At this time, if he came over to talk to me about trivial matters at work, or complained about something, I would just respond with a yes. In fact, it was a kind of passive silence. I had no energy to speak or use my brain to respond. It wasn’t directed at him; it was simply that I was physically and mentally exhausted and didn’t want to speak. In another situation, if he says something that I don’t agree with outside or in front of friends, I will remain silent and will not dismantle him on the spot. Chinese people pay attention to face. A man should always be treated with dignity when he is outside. If anything happens, we can talk about it at home, or even not talk about it at home. There is no need to embarrass him over a trivial matter.

Researcher: Comparing these situations, in which situation do you remain silent most frequently? Is it a time of culture clash, or a time of heightened emotion?

Interviewee: The conflicts between cultural concepts are the most common. Small conflicts and emotions in daily life actually pass quickly and can be solved with just a few words. But cultural differences are unavoidable, and we encounter them every once in a while. And no matter how much you talk about this kind of thing, it’s useless. It can’t change the other person’s perception for decades, so you can only deal with it with silence. Silence when you are emotionally agitated is to keep yourself calm; silence when there is a cultural conflict is to know that what you say is in vain, and you simply don’t say anything.

Researcher: I understand. The causes of triggering are different, and the nature of silence is also different. Then I want to ask you, what is the main reason why you choose to remain silent? For example, do you not want to quarrel, feel that explanations are unclear, maintain relationship harmony, protect each other’s face, or are you influenced by cultural concepts, or are you under pressure to express yourself in language?

Interviewee: There are many reasons, but the core one is to maintain family harmony. I have heard the saying “family and everything will be prosperous” since I was a child. I think it is more important than anything else. When two people live together, especially in a cross-cultural marriage, the differences are huge. If they argue and quarrel every day, the family will break up. Silence can at least make the home quiet and prevent chaos. In my heart, family harmony is much more important than fighting over right and wrong and gaining the upper hand. There are indeed many things that cannot be explained clearly. Culture is something that is difficult to truly empathize with without personal experience. For example, when I told him about “human relations,” “subordinates,” and “school district housing,” he could understand it literally, but he couldn’t understand the pressure and logic behind it at all. I explained it to him once or twice, but I felt tired even if I explained too much. Just like I can’t understand why he didn’t ask for money from his family much after he turned 18, and his parents didn’t care about him buying a house and getting married. Some things can’t be explained through words, so just don’t explain them and just stay silent. At the same time, you have to take care of your face, his face, and my face. For example, if he does something wrong, I won’t hold on to it and talk about it endlessly, for fear of hurting his self-esteem. If I have done something wrong, I will not keep defending it. The more I defend myself, the more embarrassed I will be. It is better to remain silent and let the matter pass. And when you are outside, you must save enough face for him, and don’t let him get off the stage in front of his friends. In the first few years of marriage, there was still a bit of pressure to express myself verbally. At that time, his Chinese was not as good as it is now, and many complex emotions and feelings could not be expressed clearly. My English was also average, and I didn’t know how to speak accurately when it came to words involving delicate emotions. Sometimes when I get angry and want to criticize him, I don’t know how to express it properly in English, so I just don’t say it at all. Now that the language is completely fine, this reason no longer exists. Or maybe it just becomes a habit. This has always been the case in my family since I was little. When my parents quarrel, my mother is always the silent one. When my father gets angry, things change. I’ve seen it since I was a child, and I feel that this is how couples get along. They don’t have to argue about everything. They just take a step back, be silent for a while, and it’s over. Of course, it also has something to do with my own personality, which is relatively introverted and reserved.

Researcher: You have repeatedly mentioned that “everything thrives when everything is at home.” This concept is really important to you. Do you feel that in order to maintain this “harmony,” you have actually suffered a lot of injustice?

Interviewee: There must be some grievances. Sometimes, after the silence is over, I will secretly shed tears while hiding in my room, wondering why I am always accommodating and why I always shut up first. But then I thought about it; he was also accommodating me. He is a foreigner who left his hometown and came to China. He adapted to my pace of life, ate Chinese food, learned Chinese, and accompanied me back to my hometown during holidays. He also made a lot of sacrifices. In marriage, especially cross-cultural marriage, someone has to give in. We can’t both be at each other’s throats, as that would make life impossible. I should keep quiet for a while and find peace at home, which is actually quite worth it. And there are many things that I felt were a huge grievance at the time, but when I look at them two days later, they are actually no big deal.

Researcher: Okay. Then I want to ask, what impact has your silence had on your communication or relationship? For example, does it maintain harmony, or does it escalate conflicts or confuse your partner? Can you also give an example to explain?

Interviewee: It can definitely avoid the escalation of conflicts and maintain superficial peace. For example, when two people are angry, if you choke on each other’s words, the quarrel will definitely become more fierce, and you can say any unpleasant things. At this time, if one person shuts up and becomes silent, there will be no more quarrels. When the person calms down, everything will be easier to discuss.

Also, when children are around, silence can prevent them from being frightened. We never quarrel in front of our children. Sometimes when we have conflicts, we just don’t talk to each other, and our children won’t notice or be afraid. I think this is very important. We should not let adults’ conflicts affect children’s emotions. But if I remain silent for a long time, he will misunderstand me and think that I am being cold and violent, that I don’t care about him, or that I am refusing to communicate. In the first few years of marriage, there were many misunderstandings because of this. I remember that it was just our third year of marriage, and it was also because we had to go to someone’s house during the Chinese New Year. I wanted to go back to my hometown to be with my parents, but he wanted to go back to England for Christmas. We couldn’t reach an agreement, so I fell silent and didn’t talk to him for several days. As a result, he thought that I regretted marrying him and wanted to divorce him. During that time, he was particularly depressed and couldn’t do well in class. He even secretly asked his Chinese colleagues if Chinese people’s long silence meant that they wanted to break up. Later, his colleague told him that Chinese people’s silence may be just because they are angry, not because they want a divorce, so he asked him to take the initiative to comfort her. Only then did he dare to come and talk to me, and only then did I realize that he had thought so much. At that time, I realized that although silence prevented an on-the-spot quarrel, it would cause a great psychological burden on the other party, making him have random thoughts and feel insecure. Another thing is that after you remain silent, the problem is not really solved, but is temporarily suppressed, like a knot in your heart, and it will explode again the next time you encounter a similar thing. For example, we didn’t talk about my child’s enrollment in class after the last silence, but I still secretly enrolled my child during the summer vacation. When he found out, he was very angry and said that I didn’t respect him, and we had a big fight again. Just because we didn’t talk about it last time and just covered up the problem with silence, it eventually broke out. The more silence you have, the more emotions you will accumulate in your heart. After a long time, you will feel tired, feel that you have been compromising in this marriage, and occasionally feel that it is not worth it. But fortunately, I can adjust myself, chat with friends, or go shopping to buy some clothes, and it gets over.

Researcher: Is there any time when silence actually made the conflict more serious, even worse than a direct quarrel?

Interviewee: Yes, it was the time my mother-in-law came here. Not long after the baby was born, my mother-in-law came from the UK to take care of me during my confinement period. In fact, she is a very nice person and has a kind heart, but her way of raising children is really very different. For example, she wants to give her children room-temperature water, saying that British children always drink cold water since they were young, and she also wants them to sleep on their stomachs, saying that this way their hair will look better. I definitely didn’t agree with her, but I was too embarrassed to say it directly. After all, she was an elder and flew all the way to take care of me. I was afraid that she would be unhappy if I told her, and that I would be treated like a donkey for my good intentions. So I kept silent. I didn’t comment on what she did, but I definitely didn’t look good, and I didn’t take the initiative to chat with her. Every day when she talks to me, I just respond with a few words and hide in the bedroom most of the time.

After staying for about a week, my mother-in-law felt that I didn’t welcome her and that she was causing trouble here, so she bought her own plane ticket to leave. My husband was very angry when he found out. He had a big fight with me, saying that I was ignorant and disrespectful to his mother. He also asked why he didn’t speak up if he had any opinions and insisted on showing off to the old man. That time we had a cold war for almost a week, which was even more uncomfortable than a direct quarrel. Later, I took the initiative to apologize to him, saying that it wasn’t that I didn’t welcome my mother-in-law, but that I didn’t know how to express my different opinions to the old man. I was afraid that I would hurt the old man’s heart, so I kept silent. He understood after listening, but also told me that I must speak out if I have anything in the future, and don’t hold it back. Silence is more hurtful than an argument. Since then, I have learned that some things can be kept silent, and some things cannot be kept silent, especially when it involves family members. Communication must be communicated, otherwise the misunderstanding will only become deeper and deeper.

Researcher: Has he ever had a serious conversation with you, saying that he doesn’t like your silence and wants you to speak out?

Interviewee: Yes, many times. He told me that every time I didn’t speak, he felt very panicked and insecure. He didn’t want to see me being cold and silent. It felt like being abandoned. He said that he hoped that if I had any dissatisfaction or thoughts in the future, I would tell him directly, even if I scold him, just don’t ignore him. I was actually quite touched when I heard it. I didn’t expect that my silence would cause him so much harm. I always thought that silence was to avoid quarrels and for the good of the family, but I didn’t expect that it would make him feel even more uncomfortable. After that, I also tried to change and speak out as much as possible when encountering problems, but it was really difficult. When encountering serious differences, I would still subconsciously shut up.

Researcher: Well, decades of thinking and behavioral habits cannot be changed simply by changing them. Next, I want to ask you, how do you usually “break the ice” and resume communication after a period of silence? Did one party take the initiative to speak, or was it through physical contact, or was it a natural transition through daily affairs?

Interviewee: We almost never specifically say, “Let’s talk.” That would be too formal and awkward. Most of the time, I speak naturally through small everyday things. For example, after a night of silence, he would come over to me the next morning and ask me, “What do you want to eat in the morning? I’ll buy it.” Or I would ask him, “Have you packed the child’s schoolbag?” With such trivial things, the atmosphere would relax as soon as we talked, and then things would slowly return to normal. It was as if the silence before had never happened. Everyone was tacit and did not mention the quarrel just now. Other times, he will take the initiative to do small things to show his kindness. For example, bring me something delicious when you come back from work, or take the initiative to wash dishes, mop the floor, or do some housework that you don’t usually do. I knew at a glance that he was handing me the steps, so I just followed them and didn’t hold them all the time. There was very little physical contact. Neither of us has a particularly outgoing personality, especially when there are children around, and we don’t go out of our way to hug or hold hands to make peace. At most, I just touch my hand when handing something over, or he pats me on the shoulder, which is considered a sign of goodwill. When no one is around, I will hug occasionally, but rarely. Most of the time, I rely on daily routines to make the transition. Another situation is when children act as peacemakers. For example, if the two of us don’t talk, the child will run over and hold my hand, and then hold his hand and say, “Mom, dad, why don’t you talk?” The two of us can’t help laughing, and then we’ll make up. Children are actually the most useful step between us.

Researcher: Generally speaking, who takes the initiative to break the ice more often? Is it your husband or you?

Interviewee: He takes the initiative more often because he is the kind of person who can’t stand silence. He doesn’t feel comfortable when the house is quiet. He also knows I care a lot about saving face and won’t easily be the first to back down, so he usually initiates the conversation to break the ice. However, if the issue stems from me, say, I’m having a rough time at work, come home and lash out, and then go silent. I’ll take the initiative to talk to him or offer him some fruit once I’ve calmed down; that counts as making up. Overall, he initiates reconciliation about 70% of the time, and I do it 30% of the time.

Researcher: I see. Next, I’d like to ask: after the silence ends, do you explain the reason for it to your partner? For instance, telling him, “I wasn’t giving you the silent treatment; I just needed to cool off”? Does that kind of explanation help reduce misunderstandings?

Interviewee: Early in our marriage, I basically wouldn’t explain. Back then, I felt like, “You’re my husband; you should understand why I’m silent. Do I really have to spell it out?” I also thought that once something was over, it was over. There’s no need to bring it up again, as doing so might just trigger another argument. It wasn’t until a major misunderstanding involving my mother-in-law, and another one involving a visit to my parents’ home, that I realized he truly didn’t understand. If you don’t say it, he really doesn’t know what you’re thinking. So, since then, once the atmosphere has eased after a period of silence, I’ll occasionally explain things to him. For example, last time he mentioned quitting his job to travel, I stayed silent all evening. The next morning at breakfast, I told him, “I didn’t stay quiet on purpose yesterday; it’s just that the news was so sudden I couldn’t process it right away. Plus, our perspectives are so different that I was afraid we’d argue if I said too much, so I just kept quiet.” Explaining definitely helps. At the very least, once he hears it, he stops letting his imagination run wild; he knows I don’t love him any less and I’m not targeting him. I just need time to digest things. But the help is limited; he can understand that I need to cool off, but he still doesn’t approve of that approach. He always says, “If there’s an issue, we can talk it out together. Why resort to silence?” Just as I don’t understand why everything has to be clarified on the spot. Why not wait a bit until everyone has calmed down? It’s a cultural difference; there’s no way around it. Explaining lets him know why I was silent, but it doesn’t make him agree with the method. Still, it’s better than not explaining at all; without an explanation, his thoughts would just go further and further off track. Researcher: Have you ever tried giving a heads-up before the silence sets in? For instance, saying something like, “I’m feeling a bit worked up right now; I’d like to calm down for ten minutes before we talk”?

Interviewee: I’ve tried it a few times, but it’s hard to pull off. When emotions really flare up, or when I feel utterly helpless, the thought of saying that doesn’t even cross my mind. I just clam up instinctively. Also, sometimes the silence isn’t because I’m angry; I might just find the conversation pointless or lose interest in the topic. I don’t know how to articulate that feeling, so I just go silent. Only on rare occasions, when I’m being rational, do I say something beforehand like, “Let’s drop this topic for now and talk about it later”, which I suppose counts as a form of “announced” silence. But that doesn’t happen often; most of the time, I just suddenly stop talking.

Researcher: I understand; it’s often an instinctive reaction, so it’s hard to plan ahead. Let’s shift to a cultural perspective. Do you think certain concepts in Chinese culture, such as “harmony brings prosperity to the family,” “silence is golden,” or “think thrice before acting,” or perhaps your upbringing, have influenced your tendency to go silent in your cross-cultural marriage?

Interviewee: My parents always told me that when living as a family, harmony is paramount—don’t nitpick, don’t be overly competitive, and know when to take a step back. So, whenever I do something, my first thought is whether it might disrupt family harmony or hurt feelings. I feel it’s fine to say less or stay silent for a moment. This is especially true with those close to me; you have to be careful with your words because you know exactly where their vulnerabilities lie, and the wrong words can be deeply hurtful. So, when a conflict arises, my first reaction isn’t to blurt something out; I hold back and consider whether it’s appropriate to say it. After thinking it over, I often decide against saying anything at all and just stay silent. Chinese culture values composure and avoiding impulsiveness, and that applies to speech, too. Westerners might say whatever comes to mind, being very direct, but we don’t do that; we filter our words through our minds, considering the other person’s feelings and the potential consequences. Often, the words are right on the tip of my tongue but get swallowed back down, turning into silence. Then there’s the concept of “face”—that plays a significant role, too. Chinese people value the principle of “leaving a way out so we can meet again on good terms later”; even between spouses, it is important to save face for the other person and leave some room for maneuver for oneself. One shouldn’t speak in absolute terms or act in a way that burns bridges. So, even if I don’t agree with his ideas, I wouldn’t refute him on the spot until he’s speechless; instead, I’d give him a graceful way out, using silence to signal my disagreement without actively arguing. I grew up in this kind of environment, and through constant exposure, I came to believe that this is simply how couples interact. Silence isn’t the silent treatment or a cold war; it’s a way of getting along, and a way of giving each other space. Naturally, I adopted this approach after getting married, though I hadn’t anticipated that it would so easily lead to misunderstandings in a cross-cultural marriage.

Researcher: So, do you feel that this habit of silence—cultivated since childhood—has actually become a communication barrier in your cross-cultural marriage?

Interviewee: It’s hard to say; there are pros and cons. If I had married a Chinese husband, we’d likely share the same logic of interaction: a moment of silence would be enough for him to understand what I meant—no need for words, the mutual understanding would just be there. But in a cross-cultural marriage, that doesn’t work. If I stay silent, he genuinely doesn’t understand and starts overthinking things, which actually creates more conflict. On the other hand, silence has certainly helped us avoid many clashes. If we were both straightforward like Westerners, saying exactly what’s on our minds, we’d probably be arguing every day and wouldn’t have lasted this long. So, I wouldn’t call it just a barrier; it’s a double-edged sword. It can prevent conflict, but it can also cause misunderstandings. The key is finding the right balance; knowing when to stay silent and when to speak up is crucial. After years of adjusting to each other, I’ve learned to speak when appropriate and remain silent when it’s not. While misunderstandings still happen, things are much better now than they were when we first got married.

Researcher: So, looking back over the years, do you feel there has been a significant change in how often or in what way you use silence in your cross-cultural marriage? What are the differences between the early days of your marriage and now?

Interviewee: In the beginning, the language barrier was a major factor; I didn’t know how to accurately express deep thoughts or emotions in his language. Since I couldn’t articulate them clearly, I’d simply choose not to say anything at all. There was also a lack of understanding regarding each other’s cultures and temperaments; even over minor issues, I’d feel there was a disconnect or an inability to communicate, so I’d retreat into silence. Back then, the silence was mostly involuntary. It happened when I encountered a situation and didn’t know how to respond or resolve it, so I simply kept my mouth shut. It was a silence born of feeling at a loss. It also lasted a long time; a single bout of anger could lead to silence for a day or two, sometimes even three or four. It was essentially the “silent treatment.” Now that we’ve been married for so many years, the frequency of such silence has dropped significantly; it might happen only once every month or two. For one thing, we know each other so well and we know where the other person’s boundaries lie and what words might trigger anger, so we are careful in our daily interactions and avoid stepping on those landmines. We tend to turn a blind eye to minor issues; we let them slide without taking them to heart, so they don’t trigger a silent standoff. Plus, I’ve grown personally, and my communication skills have improved. Things that used to make me go silent are now things I try to express in a way he can accept, rather than just bottling it all up. Also, the silence now is more of a conscious choice. I realize that speaking up about a particular issue won’t help. It would only lead to an argument, so I choose not to say anything; I opt for silence. It’s not that I don’t know how to speak; it’s that I simply don’t want to. The duration is shorter, too, an hour or two at most, or maybe half a day, and we’ve usually made up by bedtime. I don’t get into that stubborn mindset I had when I was younger, where I’d refuse to speak for days on end. Simply put, as I’ve aged, my mindset has become calmer, and I’ve gained a broader perspective. I no longer feel the need to argue over who is right or wrong, nor do I view silence as a form of self-sacrifice or grievance. I know what matters and what doesn’t; for trivial things, a moment of silence lets the moment pass, and there’s no need to waste time and energy on them.

Researcher: So, do you think this change stems more from the two of you developing a rapport through the process of adjusting to one another, or from a shift in your own mindset and perspective?

Interviewee: It’s a bit of both; neither could happen without the other. First, there’s the process of adjustment. We’ve been together for twelve years, living side by side every day; no matter how vast the cultural or personality differences were initially, we’ve developed a mutual understanding over time. He knows my silence isn’t a form of emotional abuse, and I know his bluntness isn’t malicious. We both know each other’s boundaries and are willing to accommodate one another—that’s the foundation.

Then there’s the change in my own mindset. When I was younger, I felt that marriage was about logic and determining right from wrong. I believed my views were correct and his were mistaken, and I constantly wanted to change him. I don’t see it that way anymore. In a marriage, especially a cross-cultural one, there isn’t really an absolute right or wrong; people are simply different, not necessarily right or wrong.

Once I realized this, I stopped obsessing over getting my partner to agree with me, and I stopped feeling aggrieved when I wasn’t understood. Silence ceased to be a reluctant compromise and became an act of active acceptance. I acknowledge our differences and am willing to embrace them; that’s why I choose silence over arguing. I think this shift is positive. It shows our marriage is maturing.

Researcher: I can sense that you have a very calm mindset now. For the final question: regarding your tendency toward silence in this cross-cultural family, is there anything important you’d like to add that we haven’t covered yet?

Interviewee: My silence often stems from caring and a desire for acceptance. Because I value this family and our relationship, I avoid saying hurtful things or getting into heated arguments; instead, I choose to take a step back and remain silent. This is especially true in cross-cultural marriages, where the differences between partners are vast. You can’t force the other person to change into the version of them you want, nor can you impose your own culture on them. In those moments, silence acts as a buffer, giving each other space and allowing the relationship some breathing room. Of course, staying silent indefinitely isn’t the answer. You still need to communicate and clarify things when necessary. Silence is a means, not an end; the ultimate goal is a harmonious home. If silence leads to more misunderstandings or conflict, you have to adjust. You can’t just keep quiet forever. One more thing: silence isn’t exclusive to the Chinese partner; my husband goes silent sometimes, too. For instance, if I bring up social obligations or etiquette that he finds baffling, he might just clam up, shrug, and go about his business. The truth is, regardless of cultural background, everyone has moments when they don’t want to talk or need silence. It’s just that the triggers, the duration, and the interpretation of that silence vary from person to person. My perspective on silence also changed after we had children. I used to think silence was just an adult matter, no big deal. Later, I realized that children are actually very sensitive; they can sense when their parents aren’t speaking to each other. So now, I try to avoid silence in front of the kids. If a conflict arises, I either address it openly right then and there or wait until the children are asleep. I don’t want my child to grow up in a stifling atmosphere, nor do I want him to think that silence is the only way to solve problems.

**I-11: (Female, US partner, with children, married 4–10 years)**

Researcher: Right, let’s continue with that topic. Looking back at your time together, what do you think are the most noticeable impacts that your cross-cultural backgrounds, such as differences in values, mindsets, and communication styles, have had on your relationship?

Interviewee: There have definitely been both pros and cons, and they’ve been quite apparent. On the positive side, life is genuinely more interesting than it is for couples from the same culture; we get exposed to so many different things. It’s like opening a whole new window on the world. For instance, we celebrate both sets of holidays earnestly: we observe the Spring Festival and the Mid-Autumn Festival, but we don’t skip Christmas or Thanksgiving either. Every Spring Festival, I’ll get him to help make dumplings and put up spring couplets; I tell him the legend of the Nian beast and teach him how to offer New Year’s greetings to elders. For Thanksgiving, he’ll spend the week leading up to it researching recipes to roast a turkey and bake pumpkin pie, and he shares stories about how his family celebrated on the farm when he was growing up. It’s the same with raising our child.We tell her fairy tales from both cultures and teach her both Chinese and English. We’ve used a bilingual approach from the start so she understands that there are different cultures and lifestyles in the world; I think that really broadens her horizons. Then there’s the difference in mindset: he’s incredibly direct and approaches problems simply. Sometimes I’ll agonize over the nuances of social etiquette or interpersonal dynamics, but he’ll cut right to the heart of the matter with a single sentence, helping me break out of my habitual way of thinking. But the core issue really lies in communication styles and mindsets. There are distinct differences between people from our respective countries. I have a typically Chinese, indirect communication style; I like to beat around the bush rather than flatly refusing something or making a direct demand. I always feel that being too blunt might hurt feelings, and that leaving a little wiggle room makes things more comfortable for everyone. He, on the other hand, is a straight shooter. He says exactly what’s on his mind. He never tries to guess what I’m thinking, nor does he ever drop hints. Sometimes, when I say “whatever” or “anything works,” he takes it literally and assumes I truly don’t care. For example, when we’re deciding where to eat on the weekend, I might actually be craving hotpot but worry he’ll think it’s too greasy, so I’ll say, “Whatever you like. You pick.” He’ll end up picking a random place, and I’ll have no appetite the whole time we’re there. Then he’ll ask with a look of genuine innocence, “Didn’t you say it didn’t matter? Why are you upset?” It leaves me feeling a mix of annoyance and amusement. There are so many little things like that. In the first six months of marriage, it happened almost every week; things only improved after we’d spent time adjusting to each other and learning from our mistakes. There are also differences in values. For instance, our attitudes toward spending. He holds a typical American mindset: living in the moment, buying what he likes, and rarely saving money. He believes money is meant to be spent and thinks lowering one’s quality of life just to save is foolish. I, on the other hand, come from a traditional Chinese background; I value saving and feel secure only when I have a nest egg. I like to shop around and compare prices, and I absolutely refuse to spend money unnecessarily. We still have occasional disagreements on this front, though we are gradually adapting to each other.

Interviewer: Next, I’d like to ask about your daily interactions or times when conflicts arise: do you ever go silent? Could you describe one or two specific instances of silence that left a deep impression on you? For example, what was happening, what was the atmosphere like, how long did the silence last, and what were your main thoughts while you were silent?

Interviewee: Oh, definitely. I’m not the type of person who likes to argue; my first reaction when something happens is to clam up and take a moment to compose myself. After I finished my postpartum recovery period, I was dealing with a bit of postpartum depression. My mood and overall state of mind were really poor. On top of that, my body had changed significantly after giving birth, and looking at myself in the mirror made me anxious. One day, we went for a walk in the park, and I asked him to take a photo of me. When I saw the picture, I looked so fat and felt so ugly. I muttered a few words of complaint, but he just said, “But that’s what you actually look like.” I completely broke down right then and there. I didn’t want to say a single word to him for the rest of the day. I kept thinking, “I’m already in this state. Why do you have to say something hurtful to provoke me?” Another instance was last month when we went to a friend’s birthday party. Before leaving, I was busy getting everything ready: the gift for our friend, snacks for the baby, wet wipes, and a spare change of clothes—just in case she got dirty or threw a tantrum while we were out. I’m not exactly fast to begin with, and there were so many odds and ends to pack, so it took a few extra minutes. He kept leaning against the doorway, urging me on: “Why are you taking so long? Everyone else is already there waiting for us. Can you hurry up?” When he said that, I paused mid-fold with the clothes and just stopped talking. I stayed silent for several minutes, quietly packed everything into the bag, grabbed it, and walked out the door. I barely spoke to him on the way there, either. I just felt a deep sense of grievance. It wasn’t like I was dragging my feet; I was packing things we genuinely needed. If the child got hungry or their clothes got dirty, I’d be the one dealing with it, wouldn’t I? He was only focused on not being late and hadn’t considered those details at all. But I didn’t want to argue with him right then and there. After all, we were heading to someone’s birthday party. Fighting before leaving would have been a bad omen and made things awkward for everyone once we arrived. Besides, I didn’t think he’d really understand even if I explained it; he’d likely just think I was making a fuss over nothing. So, I simply chose to stay silent and let it go.

Researcher: Did you talk it through after it was over?

Interviewee: We talked that night after the child had gone to sleep. He’d realized I was upset on the way home, but with the car full of people, he didn’t say anything while driving. Once we got home, he apologized on his own initiative, admitting he’d been in too much of a rush and hadn’t realized how much I had to pack. I told him how I felt—hurt and frustrated—and we cleared the air pretty quickly. It wasn’t a huge deal, really; in that moment, I just didn’t feel like talking.

Researcher: When you were silent, did you anticipate his reaction? For instance, did you know he would come over and ask you what was wrong?

Interviewee: I had a pretty good idea. I know his personality. He can’t stand an awkward silence. If I didn’t speak, he was bound to come over and ask. So, I wasn’t worried; I just felt that staying quiet for the moment was better than getting into an argument.

Researcher: I see that covers those two specific scenarios. I’d like to ask: in what specific situations are you more inclined to remain silent? For example, when facing accusations from your partner, discussing sensitive topics, dealing with clashes in cultural values, feeling exhausted, or experiencing emotions like hurt or sadness?

Interviewee: Actually, there are quite a few situations where I instinctively go silent. All the ones you mentioned apply. Whenever a conversation touches on sensitive topics involving deep-seated cultural values, like going home for the Lunar New Year, attitudes toward spending, our child’s future education, whether to emigrate, or how to handle eldercare for both sets of parents. I tend to clam up. Underlying these issues are decades of deeply ingrained cultural differences; you can’t just resolve them with a single argument. I often feel that the more I say, the more likely I am to say the wrong thing, so it’s better to pause and think about the best way to express myself. Then there are times when he criticizes or dismisses me, like I mentioned earlier, complaining that I’m too slow or saying I did something wrong, and my first reaction is to clam up. It’s not that I agree with his criticism; it’s just that when I’m being called out, the words I’d use to argue back get stuck in my throat. Especially when I’m already feeling aggrieved, I’m even less inclined to speak; I’m afraid that if I do, my voice will crack with tears, which would be even more embarrassing. When I’m utterly exhausted, I don’t have the energy to respond. I just want to zone out. And when I’m furious, I don’t want to talk either; who’s in the mood to chat when they’re angry?

Researcher: So, how does the silence born of exhaustion differ from the silence that occurs during a conflict?

Interviewee: It’s completely different. Silence born of exhaustion is about zoning out. It’s devoid of emotion; I simply lack the energy. I hear what he’s saying, but it doesn’t really register. Silence during a conflict, however, is charged with emotion—whether it’s a sense of grievance or anger. My mind is racing, but I just don’t want to open my mouth.

Researcher: I see. I’d like to dig a little deeper: what is the primary reason you choose silence? Is it to avoid arguments, a feeling that things can’t be explained clearly, a desire to keep the peace, or save face? Or is it influenced by cultural norms, the pressure of expressing yourself in a foreign language, or simply not knowing how to respond in the moment?

Interviewee: The main reason is that I just can’t be bothered to argue. I don’t want to fight, and I’m afraid of damaging our rapport or ruining our moods. Sometimes, if I just hold back and stay quiet, the moment passes. For instance, he sometimes leaves things lying around in a mess, which bothers me. If I were to say something, he might think I’m being fussy, and we’d end up arguing. So, I just don’t say anything; I tidy up myself to save the hassle. Then there’s the genuine feeling that things can’t be explained properly, especially when cultural differences are involved. Some things are ingrained in the Chinese psyche; I could talk at length about them in Chinese, but in English, I can’t find the right words. Even if I managed to translate them, he wouldn’t grasp the underlying cultural logic. There’s also the pressure of verbal expression. While daily communication is fine, when emotions run high, like when I’m extremely angry or hurt, my brain defaults to Chinese words. Trying to switch to English makes me freeze up instantly. Sometimes, it really just comes down to not knowing how to respond in the moment. For instance, if he suddenly tells me about a major decision, something I’m completely unprepared for and haven’t had time to process. I might fall silent for a moment to gather my thoughts before deciding whether to agree or disagree. Take the time he suddenly mentioned wanting to quit his job to start his own business; I was totally stunned and stayed silent for quite a while. It wasn’t that I opposed or supported the idea; I just hadn’t processed it yet and didn’t know what to say.

Researcher: You mentioned the pressure of expressing yourself verbally earlier. Does that affect you significantly? Do you ever avoid speaking English altogether because you’re afraid of making mistakes?

Researcher: Does the need to “save face” for the other person influence your decision to stay silent? For example, do you choose silence in public settings to avoid embarrassing him?

Interviewee: Yes, that’s definitely a factor. When we’re out with friends or his colleagues, even if he does something wrong, I certainly wouldn’t call him out on it right then and there; I’d stay silent and bring it up later at home. I feel like men need to save face in public, criticizing him on the spot would put him in an awkward position and look bad to others. But he doesn’t seem to grasp that concept at all. Once, when we were out for a meal, I ordered too much food. He immediately said, “Can we actually finish all this? It’s such a waste.” I was so embarrassed I blushed and quickly tried to smooth things over. Later, when I talked to him about it, he acted completely innocent, saying, “I was just stating a fact. Why couldn’t I say that?” I suppose that’s a cultural difference.

Researcher: I see. How do you think your silence affects your communication or relationship? Does it help maintain harmony and defuse conflict, or does it escalate tensions, leave the other person confused or hurt, or create lingering resentment? Could you give an example?

Interviewee: The most immediate benefit is that it gives us both time to cool off, preventing us from saying hurtful things in the heat of the moment and acting as a buffer against conflict. I find that really useful. For instance, there was a time he accidentally broke a ceramic ornament my grandmother had left me. It was incredibly important to me, something I’d kept with me since childhood. When I saw the shards on the floor, I felt like my head was going to explode; I was absolutely furious. If I had spoken right then, I’m sure I would have said some really nasty things. But I forced myself to hold back and stay silent; instead, I turned around and went out to the balcony for half an hour. During that half-hour, I gradually calmed down; I knew he hadn’t done it on purpose and that he must have felt terrible about it. If I had scolded him right then, the item wouldn’t have been restored, and it would only have made him feel worse. When I returned, he had already cleaned up the shards and was standing there looking at me, at a loss for what to do. I told him, “It’s okay; you didn’t mean to do it. Just be more careful next time.” He was really surprised. He thought I was going to fly into a rage. You see, a bit of silence can really prevent a lot of unnecessary arguments.

However, there are times when it confuses him or even makes him feel hurt. In his culture, you’re supposed to speak your mind; silence is seen as a form of “cold violence”—a sign that you no longer love or care about the other person. So, whenever I went silent, his mind would run wild, imagining all sorts of things. Also, sometimes I’d take out frustrations from arguments with friends or being scolded by my boss on him, which really wasn’t right. And if silence becomes a habit—bottling things up instead of talking—it eventually turns into deep-seated resentment. Short, intentional silences used to cool off are good, but silence born of avoidance—prolonged and unexplained—damages the relationship and easily breeds misunderstandings and grudges.

Researcher: So, looking back, do you think silence has had more of a positive or negative impact on your relationship?

Interviewee: In the first few years of marriage, it was definitely more negative. I didn’t know any better back then; whenever an issue arose, I’d retreat into silence, which caused a lot of unnecessary misunderstandings. Now, because I manage the duration of the silence and make an effort to explain things afterward, it has a more positive effect, mainly acting as a buffer for emotions and easing the tension. But overall, I still think the downsides outweigh the benefits. After all, silence isn’t communication; it just delays the conflict. Truly resolving issues requires speaking up.

Researcher: How does he usually react when he misunderstands your silence? Does he ask what’s wrong, go silent himself, get impatient, or try to draw you out? What do you think of those reactions?

Interviewee: Whenever I go silent, he assumes I’m angry or upset with him, so he’ll keep pressing me to explain why. But sometimes, when I’m angry, I just don’t want to deal with him; other times, I genuinely need time to process things, or I’m simply tired. So, I’ll ask him not to keep pressing me and to give me some space first. Of course, there have been times when we’ve given each other the silent treatment, going a long time without speaking or acknowledging one another. But that rarely happens; it lasts half a day at most, and he’s always the one who can’t hold out and speaks up first. He says he can’t stand the silence at home. It feels too stifling. We’ve developed a rapport now, though; he knows when to ask and when to give me space.

Researcher: Did the change in his reaction come about because you specifically discussed it, or did he figure it out on his own over time?

Interviewee: A bit of both, I suppose. After one particularly big argument, we sat down and had a serious talk. I explained that when I’m angry, I don’t want to talk to anyone. It’s just how I cope. I need time to sort out my emotions, and I’ll speak up when I’m ready; I asked him not to keep pestering me and to give me some space. He told me that seeing me go silent made him anxious and led his mind to wander; he hoped I could at least say something like, “I’m fine, I just want some quiet time.” Since then, we’ve reached an understanding: he doesn’t pester me relentlessly, and I try my best to give him a signal first.

Researcher: I see. Let’s talk about this from a cultural perspective. What concepts within Chinese culture, or aspects of your upbringing, do you think influence your choice to remain silent in an intercultural relationship?

Interviewee: For starters, the elders in my family always taught me that living together requires harmony—that arguing damages relationships and that taking a step back opens up a world of possibilities. So, subconsciously, I feel that maintaining family harmony is the top priority. Unless it’s a matter of principle, I tend to endure things, stay quiet, and avoid stirring up conflict. In my marriage, I habitually use silence to avoid arguments; I feel that if I don’t speak, a fight won’t break out, and the home remains harmonious. Even if I’m feeling upset inside, I just bottle it up and let it pass. I believe in thinking carefully before speaking rather than blurting out whatever comes to mind. After all, the more you say, the more likely you are to make a mistake; trouble often stems from loose talk. When I was little, if I talked too much or talked back to adults, my mother would say I was ill-mannered and that outsiders would think I lacked proper upbringing. It’s the same in marriage; I often turn thoughts over and over in my mind but choose silence because I feel speaking up might be inappropriate or lead to misunderstandings. Plus, my parents were prone to the “silent treatment.” After an argument, they could go days without speaking to each other, so my family of origin has definitely influenced me in that regard.

Researcher: Do you feel that these deeply ingrained beliefs and habits are particularly hard to change?

Interviewee: It is indeed difficult. Often, it’s an instinctive reaction, swallowing words just as I’m about to say them, without even realizing I’m doing it. For instance, there are times when I’m genuinely angry and want to speak my mind, but just as the words reach my lips, I think, “Forget it; why say it and hurt the relationship?” and fall silent instead. It has become second nature; it’s not something I can simply flip a switch and change. That said, I am consciously trying to adjust; after every instance of silence, I remind myself that next time I need to speak up—that I can’t keep going on like this. After all, my husband isn’t just anyone; he’s the person I’ll be spending decades of my life with, so active communication is certainly better than prolonged silence.

Researcher: Have you ever wondered if you would use silence more frequently if your partner were also Chinese?

Interviewee: If we were both Chinese, he would likely understand what I meant when I stayed silent, without me having to explain it explicitly. For instance, if I fell silent because I was angry, he would know I was upset and come to soothe me or reflect on his own behavior. The “cost” of silence would be much lower in that case.

Researcher: Do you feel there has been any change in the frequency or way you use silence in your cross-cultural marriage? How do things differ now compared to when you first got married?

Interviewee: During the first few years of our marriage, I used to shut down and stay silent a lot. But now, I’ve become much more proactive in explaining the reasons and meanings behind my silence so he isn’t left guessing. When we were newlyweds, whenever we argued, I would often just turn around, walk into the bedroom, close the door, and stay by myself. I wouldn’t open the door when he knocked or answer his calls; I’d completely shut myself off and had absolutely no desire to communicate. Silence was a frequent occurrence back then. Almost every time we had a tiff, I’d go silent and ignore him, sometimes for the better part of a day. Now, I realize I can’t use my personality as an excuse to make my partner bear the anxiety caused by my silence. A close friend of mine who studied journalism and communication once explained the concepts of high-context and low-context cultures to me. He said China is a high-context culture where communication is indirect and relies on implicit understanding, whereas the US is a low-context culture where communication is direct and relies on explicit words. That really resonated with me, and we talked about it at length. He also told me that while he really wanted to understand me, he couldn’t possibly guess what I was thinking if I didn’t say it out loud. Since then, I’ve made a conscious effort to adjust. For one thing, the frequency of my silence has dropped significantly; I now speak up about many of the minor issues I used to handle by staying silent. It turns out that speaking up usually leads to no problems at all. He accepts it readily and doesn’t get angry. The way I go silent has changed, too. Before, I’d clam up without warning, saying nothing and leaving him to guess. Now, if I really need to cool off or have some quiet time, I’ll tell him beforehand: “I’m feeling a bit emotional right now and need ten minutes to calm down. Let’s talk later, okay?” Or, after the silence ends, I’ll explain right away why I went quiet, whether I was angry, felt wronged, or was simply deep in thought. Of course, I haven’t changed completely; sometimes, when emotions run high, I still instinctively go silent. But it’s a vast improvement over the early days of our marriage. At the very least, my silence doesn’t turn into a misunderstanding or a “cold war” anymore.

Researcher: Did you find this process of change difficult? Did you encounter any obstacles?

Interviewee: It wasn’t too bad, actually not particularly difficult, because, after all, we’re building a life together. Marriage is about mutual compromise; he’s making changes for me, too. For instance, he’s learned to be a bit more tactful. He doesn’t blurt things out bluntly anymore and is more considerate of my feelings. Plus, we both make an active effort to learn about each other’s cultures, habits, mindsets, and even our native languages. We are both still willing to listen to each other’s views and thoughts. Since we both want to build a good life together, we have to put in the effort and pull in the same direction. He often encourages me, saying it’s great that I’m willing to speak up now. He’s making changes too; for instance, when something comes up, he’ll wait for me a moment instead of pressuring me for an immediate answer. I feel like it’s a case of “one plus one is greater than two”—that’s the kind of outcome you want in a marriage; otherwise, it loses its meaning and just becomes draining.

Researcher: I see. If you compare yourself to your Chinese female friends—those not in cross-cultural relationships, do you think your silence in a cross-cultural family differs from the silence they experience in their intimate relationships? If so, what are the main differences?

Interviewee: For them, cultural factors play a very small role in their silence; it’s mostly about personal emotions. My silence often isn’t just an emotional outlet; it’s mixed with considerations regarding cultural differences. For example, I might stay silent because I don’t know how to explain a Chinese cultural concept in English, or because I feel he wouldn’t grasp the cultural logic behind a situation, or because I’m concerned about “saving face” or maintaining harmony. You could say my silence is half emotion and half communication barriers caused by cultural differences. The consequences of that silence vary in severity, but silence can easily blow small issues out of proportion or even shake the foundation of the relationship. If the other person can’t read your emotions, you’re left to your own overthinking.

Researcher: Do you feel that your tendency toward silence has actually been forced to change because of the cross-cultural environment? If you were in a same-culture marriage, perhaps you wouldn’t have thought about changing that habit?

Interviewee: Yes, that’s exactly right. If I had married a Chinese person, I might have considered silence quite normal, like my parents, who would go silent when there was a conflict and eventually things would just blow over. But because it’s a cross-cultural marriage and silence leads to so many misunderstandings and problems, I’ve been forced to reflect and change. In that sense, while a cross-cultural marriage is complicated, it has pushed me to grow and become someone who is more willing to communicate.

Researcher: Alright, that covers most of our main questions. Is there anything else regarding your silence in a cross-cultural family, something we haven’t asked about but you feel is important and would like to add? Interviewee: I feel that silence isn’t always a bad thing; it can actually be part of cross-cultural learning. Honestly, much of the deep understanding my husband and I have of each other’s cultures came from conversations that took place after a period of silence. For instance, whenever I fell silent and later explained the reasons behind it, he gained a better understanding of Chinese culture; conversely, when he shared how my silence made him feel, I gained insight into the American logic of communication. Through these repeated exchanges, we’ve come to understand not only each other’s cultures but also our respective personalities and boundaries. In fact, our relationship is closer and more transparent than that of many same-culture couples who never argue or communicate deeply. However, balance is key; you can’t let silence turn into a habit of avoidance. If you go silent every time a problem arises without ever explaining yourself, the relationship will inevitably drift apart, and a rift will form. Silence must be followed by communication and reflection. You have to talk things through openly for the silence to have any value; otherwise, it’s just a form of silent treatment or emotional withdrawal. Also, silence isn’t always caused by cultural differences. I’ve seen many cross-cultural couples who, the moment a conflict arises, simply say, “Our cultures are different,” and stop communicating, using silence to dodge the issue. While cultural differences are a reality, they aren’t an excuse for a lack of communication. If you genuinely want to build a life together, you should take the initiative to bridge that gap rather than hiding behind those differences in silence. My husband is learning Chinese and trying to understand Chinese culture, while I’m learning to express myself more directly and adapting to his way of thinking; when both partners take a step forward, the differences don’t seem so vast.

**I-12: (Male, US partner, without children, married < 3 years)**

Researcher: Alright. For the first question, I’d like you to look back at the time from when you started dating to when you got married. What were the most noticeable impacts that your differing cross-cultural backgrounds, such as differences in values, mindsets, and communication habits, had on your intimate relationship?

Interviewee: There’s actually quite a lot to say about that. I met my wife during my second semester of graduate school in the U.S.; a friend organized a barbecue, and that’s how we connected. She’s an art teacher and a California native who had never been to China before; her knowledge of the country was basically limited to Chinese food and kung fu movies. Things were actually fine when we first started dating—the novelty of the relationship overshadowed the differences. Everything felt interesting back then; for instance, she was curious about why I drank hot water every day, and I found the lively family gatherings at Thanksgiving fascinating. But once we officially committed to each other and moved in together, the differences gradually became apparent. There were definitely differences in values; the most obvious one was our perspective on stability. I was raised to plan ahead, saving money, buying a house, mapping out the future, and taking life step-by-step. She was the opposite; she had a typical American mindset—life is short, so enjoy the moment. Money is meant to be spent, and tomorrow’s problems can wait until tomorrow. For example, when we first moved in together, we earned similar salaries, but I would consistently save half of mine every month, whereas she would spend everything she made and often max out her credit cards, needing me to help pay them off. We clashed quite a bit over that in the beginning. Then there were the differences in mindset: she tends to focus on the specific issue at hand, treating each matter separately without dragging in other things. I, on the other hand, tend to look at the big picture, considering cause and effect as well as the feelings of those around me. The most obvious difference, however, was in communication habits—which is also most relevant to today’s topic. When faced with an issue, I tend to go silent and reflect deeply before responding, whereas she demands an immediate reaction, constantly urging me to “just say what you’re thinking.” The more she pressured me, the less I could say, so I would just stay silent; the mismatch in our cross-cultural communication rhythms made talking to each other increasingly difficult. She speaks her mind openly. If she’s happy, she says so; if she’s upset, she states directly, “I’m angry.” She never holds things back or makes you guess what she’s feeling. I’m just not like that; I tend to keep things to myself, especially negative emotions. I don’t like voicing them because I feel it might hurt the relationship or simply isn’t necessary. We had a lot of misunderstandings about this when we first started living together: she’d think I was hiding something from her, while I felt she was making a big deal out of nothing—bringing up every little issue.

Researcher: You mentioned earlier that differences in communication habits became more apparent after you started living together. Could you give a specific example of a minor incident from that time to help us better visualize these differences?

Interviewee: During our first month of living together, I got stuck in traffic on my way home from work and was about fifteen minutes late. We had agreed to cook dinner together at six, but because of the traffic and my phone battery running low, I didn’t manage to let her know. When I arrived, she was standing in the entryway looking really upset. She said straight out, “You’re fifteen minutes late and didn’t even send a message. I’ve been standing here waiting for ages; I’m unhappy and was worried about you.” My initial reaction wasn’t to explain the traffic; I just thought, “It’s not a big deal. Why be so serious?” With the Chinese girlfriends I’d had in the past, they might have gotten a little huffy or acted coy, saying something like, “What took you so long?” but they wouldn’t have accused me so directly. I felt a bit awkward, so I just said, “Sorry, I got stuck in traffic,” changed my shoes, and went into the kitchen without saying another word. That just made her angrier. She followed me and asked, “Is ‘sorry’ all you have to say? Do you know how worried I was? You just don’t care about my feelings.” I was completely baffled. I thought, “I’ve already apologized; what more do you want?” I was annoyed too, but I didn’t say anything; I just kept my head down, washing the vegetables in silence. We barely spoke while cooking or eating that night; the atmosphere was incredibly tense. I only found out later that she felt my apology was perfunctory—that by not even offering an explanation, I was dismissing her feelings. But at the time, my mindset was: late is late. Any explanation would just sound like an excuse, so it was better to just admit the mistake and move on. You see? Even with a small thing like that, our perspectives were completely different. Researcher: So, the difference in how you view silence actually existed right from the time you started living together. Aside from communication habits, were there any other differences in values that led to major disagreements?

Interviewee: Oh, absolutely. There were so many. For instance, our attitudes toward spending. She also felt that once we were married, we were a family of two; she viewed both sets of parents as outsiders who shouldn’t interfere in our lives. I, on the other hand, felt that they are our parents, after all; we should show them filial respect. Sometimes, when they offer advice, it’s for our own good, so there’s no need to be so defensive. Then there were holiday plans: she insisted on spending Christmas with her family, while I felt we had to go back to China for the Spring Festival to see my parents. We almost had a massive falling-out over that issue during our first year of marriage.

Researcher: Could you describe one or two specific instances, either in your daily life or during an argument, where a moment of silence left a particularly deep impression on you? For example, what exactly was happening, what the atmosphere was like, how long the silence lasted, and what was going through your mind at the moment you chose to remain silent?

Interviewee: It happened shortly after we got married, regarding how to use our joint savings. We had received some cash gifts at the wedding, plus what we’d saved from our jobs. It came to a little over $40,000. I was thinking we could pool it together for a down payment on a small apartment here and finally settle down; after all, renting indefinitely wasn’t a long-term solution, and having a home would provide stability for when we eventually had children.

One evening, right after dinner, we were sitting on the living room sofa, and I brought up the idea. I expected her to agree, but she absolutely blew up. She called me crazy and said that money was earmarked for travel. She’d already planned it all out: two weeks in Europe, then South America. She wanted to go while we were still child-free, arguing that we wouldn’t get the chance once we had kids. I tried to explain that buying a home was an investment and a step toward stability, and that we could save up for trips later, settling down was the priority. She wouldn’t listen at all. She got more and more agitated, calling me too conservative and boring, saying I cared only about money and didn’t know how to enjoy life. She even accused me of marrying her just to get residency status and claimed I didn’t love her at all. When she brought up the issue of getting residency status, I instantly lost my temper; I felt she was being completely unreasonable and just spouting off whatever came to mind. I wanted to snap back, the words were right on the tip of my tongue, but I swallowed them. I felt there was no point in arging further; she was in the heat of the moment and wouldn’t listen to a thing. If the argument escalated, it would only be harder to resolve later. So, I just leaned back against the sofa and stopped talking. The atmosphere in the room suddenly went cold. One moment we were arguing heatedly, and the next, there was total silence—save for the sound of the AC and the occasional car passing on the street outside. That silence lasted for about twenty minutes. For the first ten minutes or so, she kept talking, berating me and claiming I didn’t respect her ideas or take her life plans seriously. Later, seeing that I remained silent, her voice grew quieter; she asked why I wasn’t saying anything and urged me to speak, but I still ignored her. She came over and tried to pull my arm, but I pulled away. That made her panic a bit; her voice started to sound like she was on the verge of tears. Eventually, seeing that I still wouldn’t engage, she went back to the bedroom on her own, gently closing the door with a slight slam. I sat alone in the living room for a long time—until well past midnight—before finally going to sleep in the guest room.

Researcher: During those twenty minutes of silence, aside from anger, were there any other thoughts going through your mind? For instance, did you struggle with whether or not to speak up?

Interviewee: I definitely struggled with it. Several times I wanted to open my mouth and argue, to pour out everything I was feeling, but I held back each time. I felt that since she was so emotionally worked up, nothing I said would get through to her. It would just be a waste of breath. I was also afraid that if I got too emotional, I might say something even more hurtful, like calling her selfish, and once said, those words couldn’t be taken back. Another thing was that I felt a sense of grievance. I had worked so hard to save money for our future together, only to have her accuse me of doing it just to get residency status. It hurt deeply, but I didn’t want to play the victim or use my hurt feelings to garner sympathy, so I just bottled it all up.

Researcher: Are there any other instances of silence that left a deep impression on you?

Interviewee: There was also the time my mother came to visit last month. My mother came to the U.S. for the first time and stayed for a month. She’s a classic Chinese mom, hardworking and a chronic worrier. Once she arrived, she cooked and tidied up for us every day, though she did tend to be a bit overbearing; for instance, she’d complain if we slept in and skipped breakfast, or criticize us for ordering unhealthy takeout. One morning, she got up just after six to make porridge, mix up some pickled vegetables, and steam buns, then called us to come eat. My wife was used to having cold cereal with milk or just a piece of bread for breakfast; she wasn’t accustomed to hot porridge, especially the savory kind. She sat down, took one look at the porridge, and said directly to my mom, “Auntie, I’m not used to eating this in the morning; I’ll just get some cereal for myself.” I was sitting right there at the table with my chopsticks in hand. I’d just picked up a bun, and I froze instantly. I looked up at my mom; the smile vanished from her face, and her hand, still holding the ladle, hung suspended in mid-air. It was incredibly awkward. My wife didn’t seem to think anything was wrong; she just got up and went to the cupboard to grab her cereal. A heavy silence fell over the table. It was dead quiet. I just kept my head down, staring at the porridge in my bowl, without saying a word.

Researcher: What was going through your mind at that moment? Why did you choose to stay silent instead of trying to smooth things over?

Interviewee: Anything I said would have been the wrong thing. I just thought, “I’d better keep my mouth shut and let this blow over.” My wife was going to leave for work right after eating anyway; I figured I could comfort my mom later and put the whole thing behind us. If I’d chimed in, it might have just escalated the conflict; arguing in front of an elder would have been terrible.

Researcher: So, how did the situation resolve itself? Did you talk to either of them about it afterward?

Interviewee: My mom was the first to snap out of it; she smiled and said, “If it’s not to your taste, then it’s not to your taste—no big deal. Young people have different habits.” Then she took her bowl into the kitchen. My wife finished her cereal quickly and left for work; she even thanked my mom on her way out. She really didn’t seem to think anything of it. Later, I had a quick chat with my mom. I told her that dietary habits are different here and asked her not to take it to heart—that my wife hadn’t meant anything personal by it. But I didn’t mention this to my wife; I didn’t think it was necessary. She hadn’t done anything wrong. She’s just a straightforward person. If I’d brought it up, she would have thought I was making a fuss about nothing, and it would have just led to an argument.

Researcher: So, in the end, you explained the situation to your mother yourself without telling your wife, effectively letting silence smooth it over.

Interviewee: Right, pretty much. I feel that talking too much about things like that just causes trouble; it’s better to let bygones be bygones.

Researcher: I see. Those two scenarios are very specific. Now, I’d like to ask: in what particular situations are you more inclined to remain silent? For instance, when facing accusations, discussing sensitive topics, dealing with cultural clashes, feeling exhausted, expressing defiance, maintaining boundaries, or when you’re emotional?

Interviewee: If the other person is extremely agitated or in the heat of the moment, I’ll definitely stay silent. When she’s angry, she speaks very fast and loudly. She gets really worked up. At times like that, she won’t listen to a word you say; the more you try to explain yourself, the angrier she gets. It just adds fuel to the fire. So, I usually don’t say anything; I wait until she’s vented and calmed down before speaking. I’m also very likely to stay silent when the issue involves both our families, especially family conflicts stemming from cultural differences. Take that incident with my mom I mentioned earlier: both sides are family, so criticizing either one would be wrong; silence was the only option. Another example is when her parents ask when we’re having kids. She considers that a private matter and snaps back, “That’s our business; you don’t need to interfere.” I just stand there, smiling and trying to laugh it off without saying a word. I can’t exactly join her in snapping at her parents, but I can’t say she’s wrong either, so I just stay silent. I’ll also definitely stay silent when I’m exhausted or feeling really down. Then there are times when what she says offends me or crosses a line; I use silence to show I’m unhappy. For instance, once at a dinner with friends, she joked in front of everyone, “Chinese people only know how to save money; they don’t know how to enjoy life.” I was right there and felt really embarrassed. I couldn’t call her out in front of outsiders and make her lose face, so I didn’t say anything. I just let my expression drop and used silence to let her know I was displeased. Finally, if there’s an issue I haven’t fully thought through yet and don’t know how to answer, I’ll stay silent for the time being. For instance, sometimes she suddenly asks me questions about the long term—things I hadn’t even considered. I won’t just give a casual answer; instead, I’ll stay silent for a moment to think it through clearly before speaking. I believe one has to be responsible with their words; you can’t just spout nonsense.

Researcher: In which of these situations do you most often choose silence?

Interviewee: Probably when the other person is emotionally agitated, or when family conflicts are involved. Other times, like when I’m tired or haven’t fully thought things through. I might stay silent too, but those instances are less frequent.

Researcher: Conversely, are there situations where you absolutely wouldn’t stay silent, where you’d insist on making things clear?

Interviewee: Yes, when matters of principle are involved. For example, if she truly misunderstood Chinese culture or said something really nasty about my parents, I certainly couldn’t tolerate that; I’d have to set the record straight. But those situations are rare. She’s just blunt, not malicious; she doesn’t say things that are truly out of line. Generally, I don’t like arguing over trivial matters; I feel it’s unnecessary and damages the relationship.

Researcher: So, silence for small things, but no backing down on matters of principle. Next, I’d like to ask: what is the primary reason you choose silence? Is it to avoid arguments, a feeling that things can’t be explained clearly, a desire to maintain harmony, saving face, cultural influences, language barriers, or simply not knowing how to respond?

Interviewee: It’s a mix of all those reasons. The main one is definitely wanting to avoid arguments, as I feel fighting really damages the relationship. I prefer to wait until tempers have cooled before communicating, so the conflict doesn’t escalate. I really dread arguments, especially ones involving throwing things or saying cruel words. I feel they are deeply damaging; every fight makes the bond a little weaker. And there are indeed things that are hard to explain, especially cultural matters or your innermost thoughts. It’s about protecting both myself and the other person. Sometimes, laying all your true thoughts out on the table makes you vulnerable to getting hurt, or hurting the other person. Ultimately, it really comes down to the influence of cultural values and communication habits—things that are somewhat difficult to change. Westerners are used to laying everything out on the table and speaking explicitly, whereas we Chinese rely heavily on the immediate context and what goes unsaid. So, as the Chinese spouse, I sometimes prefer to express my stance through silence. There is actually a lot of unspoken meaning embedded within that silence. For instance, my silence doesn’t mean I’m admitting defeat or that I don’t care about you. It might be because I realize you’re angry and I’m choosing to yield to you. Or, I might feel something isn’t right but don’t want to make you lose face in public, so I use silence to signal my disagreement. It could also be that I’m hurting inside but don’t want to burden you with my negativity, so I bottle it up. Chinese people might grasp these nuances immediately, whereas foreigners might find them harder to understand. The language barrier isn’t really an issue, though, since my English is quite good; I speak it every day and use it at work, so communication is basically seamless.

Researcher: Do you think the Chinese cultural concept of “face” plays a significant role in your marriage?

Interviewee: It certainly does. For example, when we’re around others, I’d never speak ill of her. Even if she’s in the wrong, I’ll preserve her dignity and save the discussion for when we get home.

Researcher: I’d like to ask next about the impact your silence has on your communication or relationship. On the positive side, it might maintain harmony or allow you to cool off; on the negative side, it could escalate conflicts, leave your partner confused or hurt, or create lingering resentment. Could you give some examples?

Interviewee: It’s definitely a double-edged sword. There are both pros and cons. Let’s start with the positives. For one, it can quickly defuse a conflict and give both parties time to calm down. There was a time we had a really heated argument; she was furious and accused me of not caring about her. I had been swamped with work that day and had neglected her, and she just got more and more worked up as she spoke. I was getting angry too, feeling like she didn’t appreciate how hard I worked, but I knew that if we kept arguing, we’d end up saying things that were truly hurtful. So, I just shut my mouth and stopped defending myself. I sat there and listened to her vent; seeing that I wasn’t saying anything, she eventually quieted down. Then I got up, poured her a glass of water, and set it in front of her—still without saying a word. About half an hour later, she had calmed down and came over to apologize, admitting she’d been too emotional and shouldn’t have said such harsh things. That was when I explained my side, and she said she understood. You see, if I had argued back right then, we might have actually ended up breaking up. But by staying silent and letting everyone cool off, the issue was much easier to resolve. Then there’s the ability to maintain a veneer of harmony, especially when outsiders or elders are around, so that conflicts don’t go public and everyone saves face. For instance, the last time my mom was visiting, I stayed silent; at least we didn’t get into an argument right then and there. Otherwise, my mom would have been upset, she would have felt awkward, and the whole family would have been miserable. Chinese culture values the idea that “family scandals shouldn’t be aired in public”—you can do whatever you want behind closed doors, but you have to keep things cordial in front of others. But there are plenty of negative consequences, too, arguably even more. She would get really confused and hurt, even feeling like I was subjecting her to the “silent treatment.” She’s American; In her upbringing, the concept of “using silence to express emotion” likely didn’t exist. If you don’t speak, she assumes you’re punishing her, that you don’t love her anymore, or that you want to break up. Take the first time we argued about buying a house: I stayed silent all night and slept in a separate room. Later, she told me she’d cried for ages that night, tossing and turning, convinced I was going to divorce her; she was terrified. Hearing that made me feel incredibly guilty. I had just wanted to cool off. I hadn’t meant anything more by it, but I ended up causing her such severe emotional distress.

It’s also likely to leave lingering emotional baggage, since the underlying issue isn’t actually resolved. It’s just been temporarily suppressed. Sometimes, silence actually escalates the conflict, making things blow up even more. If it starts as a minor issue, my silence makes her feel like I’m giving her the cold shoulder. Then the argument shifts from the original topic to “Why won’t you talk to me?” or “Why do you always bottle things up?” The conversation drifts further and further away from the original point, and the conflict actually gets worse.

Researcher: Did you realize at the time that your silence would lead her to imagine all those scenarios? Did you ever feel like she was overreacting?

Interviewee: At first, I really did think she was overreacting. I mean, I just wasn’t talking; what was the big deal? Right after we got married, I even thought she was making a mountain out of a molehill and being too sensitive. Later, we had a serious talk about it. She explained that when her parents fought, they would give each other the silent treatment, ignoring each other for days—before eventually divorcing. That’s why she’s so afraid of silence; whenever it happens, she feels like something terrible is about to happen and loses her sense of security. Once I heard that, I really understood and felt for her, so I gradually started to change and tried my best not to bottle things up. Researcher: Do you take the initiative to resolve conflicts caused by silence? Do you proactively offer explanations? After a period of silence, how do you usually “break the ice” and resume communication?

Interviewee: At first, I generally wouldn’t take the initiative to smooth things over or explain myself. I felt that once something was over, it was over. There was no need to bring it up again; doing so might just stir up bad memories and lead to another argument. Usually, she would be the one to break the ice—maybe by giving me a hug, bringing me something to eat, or asking if I was hungry, and I’d take that opportunity to step down from my position. Back then, I think I had a bit of a “male chauvinist” mindset; I felt it was undignified for a man to back down first. Even if I was in the wrong, I’d wait for her to speak first. Looking back, that seems pretty immature. After we’d been married a while, I gradually realized how much this mattered to her and how hurtful the silence was for her, so I started taking more initiative. For instance, after a silent spell, once I’d calmed down, I might go to the kitchen and make something she loved, like pasta or some cookies, and bring it to her, saying, “Don’t be mad anymore; have a bite to eat.” She’d usually take that opening, and as we ate, the conversation would naturally flow again. Sometimes I’d explain myself directly: “I didn’t mean to stay silent earlier; I was just so angry that I was afraid I’d say something hurtful, so I wanted to cool off.” It felt really awkward and embarrassing at first, but I got used to it with practice. Now, we have an unspoken agreement: during an argument, if either of us feels emotions running too high, we can call a “time-out,” saying something like, “I need to cool off,” and then we each go to our own rooms for a bit. We only come back out to talk once we’ve calmed down. You can’t just disappear without a word or stay silent and ignore the other person. This agreement has been really helpful; it’s cut down on a lot of misunderstandings. Sometimes, if neither of us feels comfortable speaking first, we use sticky notes—leaving a little message on the fridge like “Pizza tonight?” or “Want to walk the dog?” If the other person replies “Sure,” that counts as making up; we don’t need a formal apology. It works well for people like us who find it hard to be open. Or, we might just stay silent in person and text each other instead. Researcher: So, who tends to break the ice more often—you or her?

Interviewee: It’s usually her. I still feel a bit awkward initiating the conversation; it just feels strange to me. But I’m much better than I used to be. I used to hold back for days, but now I can only last half a day at most before I can’t help but speak up—staying at a standoff like that is uncomfortable.

Researcher: When you do take the initiative to explain or apologize, do you feel any psychological resistance, like, “I didn’t do anything wrong, so why should I be the one to back down?”

Interviewee: I definitely felt that way at first. I thought speaking first meant losing—that I’d lose face. But I gradually came to realize that between a husband and wife, you shouldn’t worry so much about that. Besides, often it’s not a matter of right or wrong; it’s just a difference in culture and mindset. There’s no standard answer. Who speaks first doesn’t matter; solving the problem is what counts. Furthermore, my silence often makes her feel insecure. Even if I wasn’t the one who caused the initial issue, my silence hurts her, so there’s no harm in apologizing.

Researcher: Let’s talk about the cultural aspect now. Which concepts from Chinese culture, or perhaps your own upbringing, do you think have the biggest influence on your tendency to choose silence in an intercultural marriage?

Interviewee: I was introverted as a child and didn’t talk much. In school, teachers preferred quiet, obedient students; as long as you didn’t cause trouble, it didn’t matter if you never spoke up in class. I remember once in elementary school, I raised my hand to answer a question, got it wrong, and my classmates laughed at me. After that, I never volunteered to speak again; I’d just sit and listen. Even if I knew the answer when the teacher asked something, I wouldn’t say it. I was afraid of being wrong. Also, whenever I did poorly on a test and tried to explain to my dad that the questions were too hard and everyone else struggled too, he’d glare at me and say, “If you didn’t do well, it means you didn’t study hard enough. Don’t make excuses.” After a few times, I stopped explaining. If I did badly, I’d just keep my head down and grunt “mm-hmm” in response to anything asked, just staying silent. Explaining was useless anyway, and I’d just get scolded. The influence of my upbringing was really profound. My parents were a classic traditional Chinese couple—the husband handled the outside world while the wife managed the home. My dad was a man of very few words, extremely reserved; he never said sweet things or expressed his emotions. When the two of them argued, there was never any shouting or screaming; instead, they would give each other the silent treatment. If my dad was angry, he’d retreat to the balcony or the stairwell to smoke in silence. My mom would just go about her routine—cooking, doing laundry, though when she was really mad, she might refuse to wash his clothes; still, the main thing was that she wouldn’t speak to him. After two or three days, things would smooth over—maybe my dad would pitch in with some chores, or my mom would call him for a meal. Having grown up watching this, I came to believe that this was simply how married couples argued: you didn’t need to hash everything out; a few days of cooling off would do the trick. I suspect my habit of staying silent is something I inherited from my dad, or perhaps picked up simply by being around him. Then there’s the concept of “face”—Chinese people place great importance on it, both one’s own and others’. We tend to hold back a bit when speaking to ensure things remain amicable for the future. We avoid absolute statements or calling people out directly; if we disagree, a smile and a moment of silence usually get the message across, allowing everyone to save face. This approach works wonders back home, where there’s a tacit mutual understanding, but it falls flat abroad; if you stay silent there, people simply won’t know what you mean. Later, I interned at a state-owned enterprise in China for six months, and the workplace culture was exactly the same: listen more, speak less; don’t interrupt your superiors; don’t ask questions or make comments that are out of line. I gradually developed this habit of silence. Even after moving abroad and changing environments, it’s not easy to shake something that’s so deeply ingrained in your nature.

Researcher: So, in the way your parents interacted, were there almost no moments where they sat down and openly discussed their conflicts?

Interviewee: There might have been a few, but they were extremely rare. I don’t think they ever really talked through their issues or had heart-to-heart conversations. I rarely have heart-to-hearts with my parents either; I generally don’t share my true inner thoughts with them. When my dad was younger, he loved playing cards and mahjong and would often come home very late. My mom would get angry and wouldn’t save any dinner for him or speak to him. A couple of days later, he’d come home early, bring some fruit, or help out with housework, and they’d make up, without ever mentioning the card games. I used to think this was perfectly normal; isn’t that just how married couples are? It wasn’t until I was with my wife that I realized that when an argument breaks out, you can actually sit down, talk things through openly, and avoid the silent treatment.

Researcher: Do you feel that this habit of silence—cultivated since childhood—works well in a domestic context but becomes an obstacle in a cross-cultural marriage?

Interviewee: Actually, I think that if silence persists for too long, it becomes an obstacle regardless of whether it’s a cross-cultural marriage or not.

Researcher: I’d like to ask next: has the frequency or manner in which you use silence in your cross-cultural marriage changed? How does the situation now compare to when you first got together?

Interviewee: It has changed significantly—both the frequency and the way I handle it are different. When we first started dating, I resorted to silence very often; almost any conflict would trigger it. Back then, I struggled to articulate myself clearly—the more I spoke, the more likely I was to say the wrong thing, so I’d simply stay quiet. I also didn’t understand her personality well; I was afraid of saying something that might upset her or making a faux pas due to cultural differences, so I often just kept my mouth shut. I remember about six months into our relationship, we attended her cousin’s wedding. Her relatives asked, “When do you plan to get married?” and I was completely caught off guard. By Chinese standards, talking about marriage after only six months of dating is too soon. If I said I wanted to get married, I’d seem too eager; if I said I didn’t, I’d appear unserious. I just smiled and couldn’t find the words. I stayed silent for a long time. Eventually, she smoothed things over by saying, “We’re still getting to know each other; we’ll let things take their natural course.” Now that we’ve been married for two years, the frequency of my silence has dropped. I’d say it’s about half of what it used to be. Plus, the silence now is usually a conscious choice; I want to calm down and organize my thoughts, rather than simply not knowing what to say. The duration has shortened, too. In the past, I might stay silent for half a day or even a full day and night; now, it’s usually just an hour or two. Once I’ve cooled off, I’ll take the initiative to talk to her. Another change is that, back then, silence meant I was stewing in anger—my mind would race with wild thoughts, and the more I thought, the angrier I got. Now, when I’m silent, I organize my thoughts internally. I consider how to express myself, how to convey my ideas without hurting her, and only speak once I’ve sorted it out. For instance, last month we had a disagreement about whether I should change jobs. I received a job offer from another company with a significant salary increase, but it required frequent business travel, spending about half of every month away. I really wanted to take it, as it was a rare opportunity, but she was opposed to it. She felt the travel was excessive and didn’t want to be left alone at home; plus, we were planning to have a child, and she worried that the separation would be detrimental and drastically reduce the time we could spend together. Initially, I tried to explain my side, but she disagreed and became quite emotional, so I stopped talking and sat in silence for about twenty minutes. During that time, I reflected on her concerns and how to alleviate them, why I wanted the job, and how to communicate this so she would understand. Once I had sorted out my thoughts, I sat down next to her and explained my reasons for wanting to switch jobs. It wasn’t just about the money, but also about my long-term career development. I promised to video call her every day, try my best to return on weekends, and bring her a gift from every trip. I also proposed doing it for just two years to save up some money, after which I would switch to a more stable job when we were ready to have a child. I then asked about her other concerns beyond just being alone. She told me she wasn’t trying to stop me from going; she was simply worried that the travel would exhaust me and that our relationship might drift apart due to the time spent apart. We eventually agreed to give it a try for three months; if it didn’t work out, I would resign and look for something else. In the past, I might have just stayed silent for a day, let the matter drop, and skipped the job—all while harboring resentment and feeling unsupported. Things are different now; silence is just a pause, but ultimately, you have to talk things through openly.

Researcher: What do you think caused such a significant change in you?

Interviewee: Spending a long time together deepened our bond and improved our mutual understanding. I realized she wasn’t an unreasonable person, and I understood that my silence upset her and made her feel insecure. When you’re building a life together, you can’t always expect the other person to accommodate you; you have to be willing to make changes for them, too. You gradually realize that if you don’t voice your thoughts, the other person truly won’t know what’s on your mind. This is especially true in cross-cultural marriages. You can’t expect your partner to read your mind. They aren’t inside your head; constant guessing just leads to misunderstandings, whereas talking things out openly actually resolves the issue. Researcher: Did your wife notice these changes in you? How did she react?

Interviewee: She mentioned that I’m more willing to talk now than I used to be; I take the initiative to explain things to her instead of bottling everything up. She’s quite happy about it. She also said she’s making changes herself. In the past, she’d get anxious and fly off the handle the moment I went silent, but now she’ll ask, “Do you need a moment to cool off?” instead of getting straight into an argument. I think it’s a two-way street: I change a little, she changes a little, and gradually we find a rhythm that works.

Researcher: Here’s another question: how do you think the process of cultural integration between a couple evolves as the marriage goes on?

Interviewee: I think it’s inevitable; the longer you’re married, the deeper that cultural integration becomes. It’s a process of mutual permeation and gradually drawing closer. At the beginning, it’s mostly about accommodating each other, like me joining her for Christmas and Thanksgiving to eat turkey, and her joining me for Spring Festival to eat dumplings, mooncakes, and zongzi. We’d push ourselves to adapt for the sake of the other person. But over time, it stops being just an accommodation; it truly integrates into daily life and becomes a habit of one’s own. For instance, she knows how to use chopsticks now, and also loves hot pot and mala xiangguo (spicy dry pot); she insists on having Chinese food at least once a week. She’s actually a bigger foodie than I am. During the Spring Festival, she takes the initiative to buy and put up chunlian (Spring Festival couplets) and even wishes my parents a “Happy New Year” in Chinese. As for me, I’ve gotten used to eating cold meals, and on Thanksgiving, I join her in roasting a turkey and watching football games. I used to think Thanksgiving was just about the turkey, but now I find the family gathering really heartwarming. Since my wife comes from a large family, our get-togethers are always packed with people. I’m close with her relatives and really like them, too; her family is so warm, and I’ve been able to fit right in. Communication styles are also part of cultural blending. I’m learning to speak my mind directly instead of bottling things up, while she’s learning to understand my silence and give me some space. Of course, when I’m really angry, I still go silent and don’t want to talk—that’s just instinct, and I think it’s perfectly normal. But most of the time, I’m more willing to communicate openly. Plus, the longer we’re together, the better we understand each other; sometimes words aren’t even necessary. A single look or expression says it all. That kind of silence isn’t about conflict; it’s about mutual understanding—a case of “silence speaking volumes.” It’s not about me becoming her or her becoming me; it’s about us evolving into a unique “us.”

Researcher: One last question: regarding your silence within this cross-cultural family, is there anything we haven’t asked about that you feel is important or would like to add?

Interviewee: In a cross-cultural marriage, the hardest part isn’t the language barrier; it’s the difference in mindsets and habits of expression. You can’t judge your partner’s behavior based on your own cultural standards. You have to try to see things from their cultural background and way of thinking, asking yourself why she said that or why he did that. Also, don’t expect your partner to instinctively understand your silence, and don’t force yourself to instantly become someone else. Adjusting to each other takes time. It doesn’t happen overnight, so give each other some space. Another point I think is crucial: don’t completely abandon your own culture and habits just to accommodate the other person. Silence has its merits, too, like showing composure, considering the bigger picture, and avoiding impulsive reactions. As long as you find a balance that feels comfortable for both of you, that’s what matters; there’s no need to completely negate who you are. We don’t have children right now, so perhaps there are fewer points of conflict. Once we do have kids, more issues and disagreements will arise, and the silent spells might become more frequent. But I believe that as long as we are willing to communicate and understand each other, there is no problem that cannot be solved.

Interviewer: If you could offer one piece of advice to Chinese people entering into cross-cultural marriages in the future, what would it be?

Interviewee: Don’t be afraid of differences, and don’t bottle things up. Try to voice your thoughts; even if you don’t express them perfectly, or even if it leads to an argument. It’s still better than leaving your partner to guess what’s on your mind. Also, share more about your culture with your partner so she understands why you go silent; it’s not a lack of love, but simply a matter of habit.

**I-13: (Male, US partner, with children, married 4–10 years)**

Researcher: To start off, I’d like you to give us an overview. Looking back at your time together, what are the most noticeable ways that your different cultural backgrounds, such as values, mindsets, and communication styles, have impacted your intimate relationship?

Interviewee: It affects everything. Communication style is definitely the most obvious one. She’s the type of person who is incredibly direct; she speaks her mind. If she’s happy, she says so, and if she’s upset, she tells me exactly what I did to annoy her without ever beating around the bush. I’m different; I tend to bottle things up, especially when I’m unhappy. I don’t like voicing those feelings because I worry it might hurt the harmony of the relationship. That one point alone caused friction almost every day during our first two years of marriage. Then there are the differences in values. Take the concept of family boundaries, for instance: she believes that once we’re married, our own little family comes first. She views both sets of parents as “outsiders” who need to respect boundaries and shouldn’t interfere in our lives. I, on the other hand, feel like, well, we’re family; why do we need so many boundaries? If my parents come to stay and help with the kids, do we really need to draw such clear lines? There’s also a difference in mindset: when we run into a conflict, she insists on resolving it immediately, even if it means arguing until 3 a.m. She has to get everything out in the open; she can’t go to sleep while still angry. I, however, tend to get more heated the longer we argue. I’d rather put a pause on it, get some sleep, and talk when we’ve both calmed down the next day. So, whenever we fought, I’d want to stop, but she’d insist on continuing. It would end up with me going silent, which just made her even angrier.

Researcher: You mentioned the differences in communication styles. Could you give us a concrete example from your daily life to help us visualize this? For instance, is there a specific moment in your day-to-day routine where you thought, “Oh, we really do come from different cultures”?

Interviewee: There are so many examples—let’s just look at our morning routine. Right after we got married, she insisted that I tell her “I love you” and give her a hug and a kiss every morning before I left for work. I really wasn’t used to that; I felt like, “We’re a married couple. Why do we need all this performative stuff?” Plus, I was usually in a rush in the mornings, thinking about the meetings I had that day; I didn’t have the headspace for that kind of thing. I’d usually just go through the motions—a quick, perfunctory hug before heading out, and she’d get upset, claiming I didn’t love her. I found that so frustrating. Does whether or not I love her really hinge on a single phrase like that? Then there’s mealtime. She likes to chat about all sorts of things while eating—work, friends, news stories, talking as she eats. Growing up, my family followed the rule of “no talking while eating or sleeping”; my parents rarely spoke at the table, so I got used to eating in silence. Early in our marriage, when she’d talk to me at the table, I’d often just give noncommittal grunts in response. She’d think I was ignoring her or acting cold, but really, I was just used to not talking while eating.

Researcher: I see—these are small, everyday differences, but they come up frequently. Let’s move on. Do you ever go silent during your daily interactions or when you’re having a conflict with your partner? If so, could you describe one or two specific instances that stand out in your memory? For example, what was happening, what was the atmosphere like, how long did the silence last, and what was going through your mind when you chose to stay silent?

Interviewee: Oh, definitely, and it happens quite a bit. Back in March, my parents came to stay with us for a month to help with the kids. One Friday, I came home from work and sensed something was off the moment I walked in. My mom was in the kitchen washing dishes with a long face, and my wife was in the living room playing with our child, looking equally unhappy. Later that night, after the kids were asleep, she sat me down on the sofa. The floor lamp was on, casting a warm yellow glow, but the atmosphere felt incredibly heavy. She said to me, “Did you see your mom chasing our daughter around trying to feed her cantaloupe today? I’ve told her so many times not to spoon-feed her. She needs to eat by herself. She’s five years old! If she’s still being fed now, what’s going to happen when she starts elementary school? I even told your mom not to do it right in front of the child, but she seemed to get upset. Couldn’t you just talk to her about it privately?” My first reaction was a headache; I didn’t say a word. I just picked up a cigarette from the table and walked out onto the balcony. I leaned against the railing and smoked, one cigarette after another. I think I smoked two... maybe ten or fifteen minutes passed? She followed me to the balcony doorway and stood there waiting for me to speak, but I just kept staring at the trees downstairs without saying a thing.

Researcher: What exactly was going through your mind during those ten or fifteen minutes? Were you thinking about how to respond to her, or did you simply not want to talk?

Interviewee: It was just chaotic, a real mess, and I was caught in the middle. I agreed with her point—children really should feed themselves, and we had actually reached a consensus on that before. But on the other hand, I felt she shouldn’t have said it right in front of my mom; it made my mom lose face. My mom raised children that way her whole life; she traveled a long way to help look after the grandkids, only to get lectured by her daughter-in-law. As her son, I couldn’t exactly turn around and tell my mom she was wrong, could I? At that moment, I felt like no matter who I sided with, I’d be in the wrong. If I spoke up for my mom, my wife would surely think I was playing favorites—that I wasn’t on her side or respecting her parenting philosophy. But if I sided with my wife, I’d have to criticize my mom, and how heartbroken would she be then? Being caught in the middle like that, anything I said would be wrong, so I just stayed silent. I thought I’d wait until everyone had cooled down, then mention it to my mom privately and smooth things over—just let it blow over. But because I kept quiet, she started crying. She said, “You’re always like this, hiding whenever there’s a problem. Do you even consider me part of the family?” Then she went into the bedroom and gently closed the door. I stood out on the balcony for another five or six minutes before going back in; once inside, neither of us said a word, and we went to sleep with our backs to each other.

Researcher: I understand; being caught in the crossfire between a wife and mother is a really tough spot to be in. So, what about the second memorable scene?

Interviewee: It was right after our first child was born. I had just switched to a new company and was under immense pressure, working overtime until 11 or 12 every night. On our third wedding anniversary, I remembered the occasion when I left in the morning and planned to get off work early to have dinner with her. But that afternoon, a major client showed up unexpectedly; the meeting dragged on past nine, and then I had to take them out for a business dinner. I didn’t get home until nearly midnight. When I opened the door, I saw candles lit in the dining room and a spread of steak, salad, and a small cake on the table, but everything had gone completely cold. She was sitting at the table with red, teary eyes; it was obvious she’d been waiting for a long time. My first reaction was guilt, but I also felt a strange sense of irritation. I felt like... it wasn’t as if I’d been out having fun; I was working hard for the sake of the family. Did it really have to be such a big deal? I just casually remarked, “Why aren’t you asleep yet? I was out with a client today and forgot to let you know.” She exploded instantly, stood up, and shouted at me, “Forgot? Today is our third wedding anniversary! I started getting ready at three in the afternoon and waited for you all night, and you think ‘I forgot’ covers it? Do you even care about this family? Do you care about me?” She got more and more worked up; through her tears, she talked about how hard it is raising the child alone, how I never show I care, and how I can’t even say a kind word. I just sat on the shoe-changing bench in the entryway—head down, shoes still on—listening to her berate me without saying a single word. It went on for maybe half an hour; eventually, she was too exhausted from crying and talking to go on, so she went back to the bedroom, slamming the door loudly behind her. I sat in the entryway for nearly an hour before finally going to sleep on the sofa.

Researcher: You didn’t say a single word during that half-hour? Did you ever think about saying “I’m sorry” or explaining the work situation?

Interviewee: I thought about it, but I couldn’t bring myself to say it. The words “I’m sorry” were right on the tip of my tongue, but I just couldn’t get them out; it felt really awkward, like, for a grown man, saying that feels a bit too mushy. Plus, I felt a bit aggrieved at the time; I was working overtime like crazy to make money—wasn’t it all for the sake of the family, so she and the child could have a better life? Why couldn’t she understand that? But I didn’t say any of that, because I knew it would just make her angrier. She’d think I was making excuses. I figured it was better to just keep my mouth shut. She was in the heat of the moment, and anything I said would just add fuel to the fire; I thought maybe things would calm down once she’d finished yelling and venting. That was my mindset at the time.

Researcher: I see; both scenarios are very specific. Next, I’d like to ask: in what specific situations are you more inclined to remain silent? For instance, when facing accusations from your partner, discussing sensitive topics, dealing with a clash of cultural values, feeling exhausted, or experiencing emotions like grievance or anger?

Interviewee: Let me think... it depends on the situation. The first type, and the situation where I’m most likely to go silent, involves my parents and in-laws. Especially when my parents were living with us, whenever the conversation turned to the mother-in-law/daughter-in-law dynamic, I’d basically clam up. Honestly, being caught in the middle is unbearable; no matter who you side with, you’re wrong. Say too much and you make mistakes, so it’s better to say nothing at all. The second type is when I’m exhausted or under immense pressure at work. For instance, after five or six meetings in a day or dealing with a project crisis, my brain is completely fried by the time I get home. I just want some peace and quiet. I don’t want to talk. If she starts going on about domestic trivialities or small matters then, I simply don’t have the energy to respond. I’ll either give perfunctory “uh-huhs” or just stay silent. Then there are times when she’s highly emotional—pointing fingers, accusing me, or losing her temper. The more agitated she gets, the less I want to speak. I feel that when people are angry, they say hurtful things they don’t mean. If I engaged, the argument would just escalate until it got out of hand. It’s better to keep my mouth shut and let her vent; once she’s let it all out, things usually blow over.

Then there are issues stemming from vast cultural differences—things that are simply impossible to explain. For example, if she complains that I’m not romantic or don’t know how to express love, or says my family’s social obligations are too much of a hassle, I usually stay silent. There’s no point in arguing; she grew up in a completely different environment and simply can’t grasp where I’m coming from. If I tried to explain, it would just look like I was making excuses, so I just didn’t say anything.

Researcher: Looking at it from the other side, are there situations where you absolutely wouldn’t stay silent—where you’d definitely speak up? For instance, if you were furious, would you explode?

Interviewee: I rarely get truly furious; I have a pretty mild temperament. If she really crossed a line, like saying something deeply disrespectful to my parents, I might say a few words. But I wouldn’t shout or scream; I’d just make my stance clear in a serious tone. Most of the time, I just bottle it up or stay silent. I’ve never liked arguing; I find it embarrassing. It doesn’t solve problems, and it just damages the relationship.

Researcher: I see. Let’s continue. What are the main reasons you choose silence? Is it to avoid conflict, a feeling that things can’t be explained, a desire to keep the peace or save face, or is it influenced by cultural values or your own communication style? Interviewee: The main reason is definitely that I just don’t want to argue. I’ve always felt that home is a place for affection, not for debating who’s right or wrong. What do you gain by winning an argument? If you make the other person cry or prove you’re right, you end up hurting the relationship. It’s not worth it. So, I tend to hold back or stay quiet whenever I can; taking a step back opens up a world of possibilities, and keeping the peace at home is more important than anything else. The thing is, some matters are truly hard to explain, especially when it comes to cultural differences. For instance, I might tell her that when elders come to help, we need to save them some face. We shouldn’t point out their mistakes to their faces. She’ll ask, “Why do we need to save face? If they’re wrong, they’re wrong; anyone should be able to point that out. It has nothing to do with respect.” I can’t explain to her that it’s not about right or wrong; it’s about social nuances and the kind of etiquette that’s been ingrained in me since childhood. She wouldn’t understand even if I explained it at length. She’d just think I was making excuses, so I simply stopped trying. Personality plays a role, too. I’m naturally introverted and not very articulate; I’m not good at saying sweet things or expressing my emotions. My dad taught me from a young age that a man should be steady—speak less and do more, and avoid being glib. He believed reliable men let their actions speak, not their words. I’ve always believed that, too; I feel that fancy talk is no substitute for solid, practical action. Sometimes, my mind just goes blank, and I don’t know how to respond. If she suddenly launches into an emotionally charged tirade, I get flustered. I don’t know what to say or how to phrase things to satisfy her, so I just fall silent until I can gather my thoughts.

Researcher: Do you face any pressure regarding language? For example, do you usually communicate in Chinese or English? Are there times when emotions run high and you can’t find the right words, so you just stay silent?

Interviewee: We mostly speak Chinese. She’s lived in China for nearly a decade and works as a teacher, so her Chinese is incredibly fluent. She has no trouble arguing in it at all. My English is decent, but when it comes to a real argument or speaking from the heart, my native language feels more natural. So, language isn’t the main issue; it really comes down to differing mindsets and personalities. Even if we spoke the exact same language, there are still things that just wouldn’t get said.

Researcher: I see. So, what impact do you think your silence has on your communication or relationship? Does it, for instance, maintain harmony or give you both time to cool off? Or does it escalate conflicts, hurt the other person, and create lingering resentment? Could you give some examples?

Interviewee: There are pros and cons, but the negative effects definitely outweigh the positive ones. On the positive side, it certainly prevents conflicts from escalating. For example, when she’s really worked up. If I were to argue back while we’re both angry, we might say hurtful things that can’t be taken back later. But if I stay silent, she eventually runs out of steam and calms down on her own, so things don’t get too ugly. Silence also gives me time to think about how to handle the situation properly, so I don’t say the wrong thing or make a bad decision impulsively. But the downsides are significant. First, she gets deeply hurt; she feels like I’m giving her the silent treatment or that I don’t care about her. Take our anniversary, for instance: after we made up, she told me that when I sat in the entryway without saying a word that night, she felt utterly hopeless. She thought I didn’t love her at all and that our home didn’t matter to me. Hearing that made me feel terrible; I hadn’t realized my silence would make her think all that. Then there’s the fact that many issues simply aren’t resolved. They’re just temporarily suppressed, treating the symptoms rather than the root cause. Like the issue of feeding the child: after I stayed silent last time, I mentioned it to my mom privately. She agreed to stop, but turned right around and kept doing it—just not when my wife was looking. When my wife found out later, we had another fight. So, my silence hadn’t solved the problem at all; instead, it made her feel like I wasn’t a man of my word and just built up more resentment. Also, over time, a rift develops between the two of you. Even though you sleep in the same bed and see each other every day, you feel emotionally distant; you bottle things up instead of talking, and you gradually grow apart. I get that feeling sometimes at night when the kids are asleep and we’re sitting on the sofa, each on our own phones, not saying a word to each other for ages.

Researcher: Regarding this “rift” you mentioned—was there any specific moment during your years of marriage when you felt it particularly strongly?

Interviewee: Yes—during the times we were giving each other the silent treatment. The longest silent treatment lasted a whole week; the house was eerily quiet. Aside from the sound of the children, neither of us said a word. We ate separately, and she slept in the bedroom while I slept on the sofa. At the time, it didn’t feel like a home at all. It was like living with a stranger I was sharing an apartment with. It was a terrible feeling, but I didn’t know how to break the ice, so we just let the standoff drag on.

Researcher: I see. Could your partner be misinterpreting your silence? How does she usually react to it? Does she press you for answers, go silent herself, get impatient, or try to draw you out? What is your take on these reactions?

Interviewee: There are so many misunderstandings. It happens all the time. She often assumes my silence is a form of “cold violence”—that I’m intentionally punishing her or that I’ve stopped loving her. She also tends to interpret my silence as tacit admission, as if I’m in the wrong or admitting fault. In reality, that’s rarely what I mean; I just don’t know how to put my feelings into words.

Researcher: Has she ever complained about specific issues? For instance, regarding the expression of affection—did she ever say anything that really stuck with you?

Interviewee: Oh, she’s said plenty. My wife always complains that I’m terrible at expressing my love. But I just can’t wear my heart on my sleeve or be as outwardly passionate as Westerners are. I’m much more reserved. Even though I don’t say the words, my love for her is woven into everything I do for her. Honestly, I feel like I do far more than I say. She loves cherries; no matter how expensive they are, if I pass a fruit shop and see fresh ones, I’ll buy them for her. When she’s up late—until 11 or 12 at night—preparing lessons, I’ll quietly warm up a glass of milk and place it on her desk without disturbing her. Last year, when her parents came to visit, I planned the itinerary, booked the hotels and flights, and drove them around the whole time. I was worried they wouldn’t be used to things in China. When the child cried in the middle of the night, I was usually the one to get up and soothe them so she could get a full night’s sleep, since she’s a light sleeper. To me, these things are all acts of love—proof that she and this family are always on my mind. But she doesn’t see it that way; she thinks these are just a husband’s duties, not expressions of affection. What she wants is for me to say “I love you” every day, to hug and kiss her when leaving the house, to give flowers and say romantic things on holidays, and to put my feelings into words clearly. I really can’t bring myself to do that; it feels incredibly awkward, like acting. It doesn’t feel like me.

Researcher: Have you ever tried to make a conscious effort to change for her sake? Like trying to say a few sweet things or expressing your feelings more openly?

Interviewee: I have, and I feel like I’m gradually getting the hang of it. Last year, for her birthday, I made a point of ordering flowers and buying a necklace in advance. When she was blowing out the candles, I forced myself to say, “Happy birthday, honey; I love you.” Just saying it gave me goosebumps; I felt completely uncomfortable and thought it seemed so forced. But she burst into tears right then and there. She hugged me and cried for a long time, saying it was the happiest birthday she’d had in all the years we’d been married. I was really moved, too; it turned out what she wanted was so simple. But that was the only time; after that, I couldn’t bring myself to say it again. I always feel that saying those things on purpose is fake—not the real me. I guess some things are just ingrained in my nature and can’t be changed.

Researcher: I see. Let’s continue. After a period of silence or the “silent treatment,” how do you usually “break the ice” and start communicating again? Who tends to make the first move?

Interviewee: Most of the time, I’m the one who makes the first move, but I rarely apologize directly; I usually break the ice through my actions. For instance, the day after an argument, I might get up early and make the breakfast she loves, like a soft-boiled egg and her favorite oatmeal, and serve it to her. She’d get the message, accept the olive branch, and start talking to me again. Or, on my way home from getting off work, I’d pick up her favorite milk tea and leave it on the dining table; I wouldn’t even have to say anything. Seeing it, she’d know I was trying to make amends, and half her anger would vanish. During the silent treatment, I’d have our daughter go over and snuggle up to her, saying, “Mommy, let’s play with blocks,” or “Daddy says you shouldn’t be mad anymore.” As soon as she saw the child, her heart would soften and her anger would mostly dissipate. Then the whole family would play together, and through that interaction, we’d start talking and naturally make up. Now that we’ve been married a long time and have found our rhythm, she sometimes takes the initiative to break the ice herself. She might tap me and ask, “Hey, still sulking?” or hand me a piece of fruit. I’d quickly seize the opening to say a few words, and we’d move past it.

Researcher: So, how would you compare the difficulty of breaking the ice through actions versus directly apologizing or communicating with words?

Interviewee: There’s a huge difference. Cooking, buying things, or doing chores feels completely natural to me. There’s no difficulty at all. But actually saying “I’m sorry,” “I was wrong,” or “Don’t be mad” feels incredibly hard, like trying to reach the heavens. I always feel that for a grown man to say those things, acting so submissive, is embarrassing and feels really cheesy. It’s probably a common trait among Chinese men: we express love through actions, but we’re clumsy with words.

Researcher: I’d like to ask about the aftermath: after the silence, do you explain to your partner why you were silent? Does that kind of explanation help reduce misunderstandings?

Interviewee: I will explain it later. Usually, it happens after we’ve made up and the atmosphere is relaxed, like when we’re chatting in bed at night or cooking together in the kitchen, and I’ll just casually mention it. I might say something like, “I didn’t mean to ignore you that day; my mind was just a mess, and I didn’t know what to say. I was afraid that if I said too much, we’d just end up arguing again,” or “Something happened at work that day and I was in a bad mood; it wasn’t directed at you.” Researcher: So, how effective is that kind of explanation? Does it really reduce misunderstandings?

Interviewee: It helps a bit, but the effect is limited. It depends on the situation. For instance, regarding things like not wanting to talk because of high work stress, after explaining it a few times, she understands now. If I come home looking grim and don’t say much, she won’t pester me; she gives me space and waits for me to bounce back before talking. Explanations work for issues like that—things that aren’t rooted in fundamental cultural differences. But when it comes to personality or cultural values, like mother-in-law/daughter-in-law dynamics or how to express affection—explaining doesn’t help. Rationally, she knows I’m introverted and that I’m in a tough spot, caught in the middle, but emotionally, she still can’t accept it. Next time I go silent, she still gets angry and feels like I’m avoiding the issue. After all, cultural differences that are ingrained in your bones can’t be smoothed over with just one or two explanations. It’s like how I know she needs direct expressions of affection, but I just can’t bring myself to do it; and she knows I’m reserved, but she just can’t accept the silence. We understand the logic, but when the moment actually comes, we still instinctively get angry or upset.

Researcher: Right. Let’s talk about the cultural factors. What concepts in Chinese culture, or aspects of your upbringing, do you think influenced your tendency to go silent in intimate relationships?

Interviewee: My dad always said a man should be steady and composed, speaking less and doing more; he thought guys who chattered away or had a glib tongue were unreliable. He believed that talking too much leads to mistakes, so it’s better to listen more and get things done. That’s exactly the kind of person my dad was. Growing up, I hardly ever heard him say sweet things. He didn’t say them to my mom, either. But he shouldered the burden of everything in the family, big or small. When my mom was sick, he was the one who took care of her; when money was tight, he worked two jobs without ever complaining. My mom would sometimes grumble about him not being good with words, but she knew he cared deeply about the family. Watching that while growing up, I felt that’s how a man should be: contributing silently without needing to say much. Then there’s the concept of “valuing harmony above all else.” In our family, we always emphasized that harmony brings prosperity; arguing was seen as deeply shameful, something that would make us a laughingstock among the neighbors. So, when conflicts arise, I tend to just endure them or make concessions rather than causing a scene or burning bridges. I’ve been conditioned since childhood to bottle up my unhappiness, to avoid speaking out and disrupting the harmony. Then there’s the issue of “face”; Chinese people place such a huge emphasis on it, not just one’s own face, but others’ as well. For instance, if an elder makes a mistake, you can’t point it out to their face; you have to give them a way to save face. Even if you disagree with what someone says, you shouldn’t contradict them directly; you have to be considerate of their dignity. I’ve heard these things my whole life, and they’ve become second nature to me. It’s the same in my marriage; I often feel that if you spell things out too bluntly, it hurts feelings and causes a loss of face, so it’s better left unsaid. Also, Chinese culture doesn’t seem to encourage men to express their emotions, especially negative ones like vulnerability or a sense of grievance. There’s that saying, “A real man doesn’t shed tears easily”; men are expected to shoulder their burdens alone. Voicing them is seen as being overly sentimental or incompetent. So, growing up, whenever I felt aggrieved or stressed, I’d process it internally. I rarely shared these feelings with anyone—not even my parents, let alone my wife. I felt it was embarrassing to talk about it, and that it wouldn’t solve the problem anyway.

Researcher: So, what was the atmosphere of communication like in your family when you were growing up? Did you and your parents often open up to each other or express your feelings?

Interviewee: Not a chance. Ours was a very traditional Chinese family; we never talked about things like that. I grew up without ever saying “I love you” to my parents, and they never said it to me, either. We kept our feelings to ourselves and showed them through actions. For example, when I went to college in another city, my mom would start packing my bags two weeks in advance, stuffing them full of food and clothes. But when she saw me off, she’d just say, “Take care of yourself”. She’d never say, “Mom misses you.” My dad was even more reticent; he’d quietly send me living expenses, and when he called, he’d only ask if I had enough money. He never asked if I was happy or if I’d been treated unfairly. So, I’ve adopted that same approach; I believe feelings are demonstrated through actions, not words. Saying nice things doesn’t compare to doing something concrete. I used to think this was the right way—until I got married and met my wife. That’s when I realized there was another way to live, another way to express affection. Researcher: I see; one’s upbringing certainly has a significant impact. Moving on, I’d like to ask: after so many years of marriage, do you think the frequency or manner in which you use silence in the relationship has changed? How does it compare to when you were first married?

Interviewee: In the first two years of marriage, I used silence a lot—probably once or twice a week. Back then, the culture shock was intense; there were differences everywhere. Every time we spoke, we’d disagree; every disagreement made me want to argue, and the thought of arguing made me go silent. I felt exhausted, like communication was impossible, as if we were from two different worlds. For instance, if she asked me to say “I love you,” I’d feel awkward and go silent; if she suggested we visit our respective families separately for the Lunar New Year, I’d think it was unreasonable and go silent; if she said my parents shouldn’t interfere in our lives, I didn’t want to hear it, so I’d go silent again. Silence was my shield back then. Whenever I faced an unsolvable problem, I’d retreat into it. Now, after seven years of marriage, I use silence much less, maybe once every two weeks or even once a month. On one hand, I’ve gradually changed; I don’t clam up as an immediate reaction anymore. I try to voice my thoughts. I might not express myself eloquently or say exactly the right things, but at least I speak up instead of just hiding. She’s adapted, too; she understands my personality and knows it’s not personal. It’s not directed at her, so she doesn’t hound me to speak like she used to. When I go silent, she just goes about her own business and waits for me to come around before we talk. We’ve really settled into a rhythm and understand each other’s boundaries and habits. Having a child brought big changes, too. A lot of our attention shifted to the child, so there were fewer conflicts between us. Plus, to create a healthy family atmosphere, we make an effort to communicate; we don’t want to give the silent treatment in front of the child or scare them. Before, silence was pure avoidance. I wouldn’t think about anything; I’d just wait for the issue to blow over. Now, when I go silent, I’m actually thinking about how to talk to her later and how to solve the problem. It’s not just about hiding anymore.

Researcher: Do you think this change was mostly driven by your own desire to improve, or was it the result of adapting to each other and environmental influences? Was there a specific incident that made you determined to change this habit of going silent?

Interviewee: It was a bit of both, I suppose. There was certainly a period of passive adjustment; constant arguing and the silent treatment are unbearable for anyone, yet life goes on, so mutual compromise becomes inevitable. The main reason I actively decided to change was my child. When my daughter was around three years old, we had an argument. I went silent while my wife cried, and our daughter was terrified; she huddled in the corner clutching her toy and sobbing, “Mommy, Daddy, please don’t fight.” Seeing her like that made me feel terrible, deeply guilty. I realized I couldn’t let my child grow up in such a cold, distant atmosphere. I didn’t want her to think that family conflict meant bottling everything up, or that men were supposed to clam up and say nothing. From then on, I made an effort to speak up more. Even if I didn’t express myself perfectly, or even if it led to an argument, it was still better than the silence that frightened my child. Also, I didn’t want to live my whole life like that—feeling a barrier between us, living together in name only but emotionally estranged; there’s no joy in that. Life is neither incredibly long nor short, but keeping everything bottled up is painful, so I tried to change my approach.

Researcher: Regarding your tendency toward silence in a cross-cultural marriage, is there anything else you’d like to add, something we haven’t asked about but you feel is important?

Interviewee: It’s just that people often seem to assume that silence in cross-cultural marriages is always the Chinese partner’s fault—that it stems from Chinese people being introverted or disliking communication. But that’s not the case; Western partners go silent too. It’s just that the reasons and the underlying meanings differ. Take my wife, for example: when she’s truly angry, she also goes silent and stops talking. But her silence is an active choice; it’s “I don’t want to talk to you; I’m protesting”—that is an expression of emotion. My silence, however, is more passive; it stems from not knowing what to say or from a desire to avoid conflict. Although the outward behavior—not speaking, is the same, the underlying meaning is completely different, and this is precisely where misunderstandings easily arise. I don’t think we should simply use Western communication standards to judge silence in a Chinese context. There is a tendency to view direct expression as “right” and silence as “wrong,” or even as a form of emotional abuse. In reality, that isn’t the case; silence can sometimes be an act of tolerance or respect. For instance, when dealing with cultural differences where there is no clear right or wrong, insisting on winning an argument can actually damage the relationship. In such moments, remaining silent to give each other some space is a form of consideration. Not every issue needs to be clarified immediately, nor does every emotion need to be vented right away. I know that prolonged silence can certainly hurt a relationship, and I am working on changing that myself. I just hope people won’t take a one-size-fits-all approach or assume silence is inherently wrong; the underlying cultural logic is different. Ultimately, whether a marriage is cross-cultural or not, it requires mutual accommodation and understanding. Whether it involves silence or direct expression, as long as both partners feel comfortable and can sense each other’s intentions, that is what matters most. We’ve spent a few years adjusting to one another and have gradually found a balance; while conflicts and silences still occur, things are much better than when we first got married. We’ll take it one step at a time. We have a long life ahead of us.

**I-14: (Female, UK partner, without children, married < 3 years)**

Researcher: Alright, let’s move on to the first question. Looking back at the time you’ve spent together, from dating to marriage, what do you think were the most significant impacts of your cross-cultural backgrounds (such as differences in values, mindsets, and communication habits) on your intimate relationship?

Interviewee: The differences that stood out most were in our mindsets and communication styles. Since English isn’t my native language, my phrasing could sometimes come across as a bit stiff or blunt, making my partner perceive an aggressiveness I didn’t intend. I also inevitably carried over certain Chinese linguistic habits; sometimes, my partner wouldn’t quite pick up on my indirectness or grasp my true intentions. My husband, on the other hand, has a classic British mindset. He’s very straightforward. If he likes something, he says so; if he’s unhappy, he speaks up immediately. He never beats around the bush. I, however, have that typically Chinese, indirect personality. I tend to consider whether the other person might get upset before doing anything. Sometimes, fearing I might hurt feelings by speaking out, I’d simply stay silent and let the moment pass.

Researcher: Could you describe one or two specific instances, whether during daily life or a conflict, where a moment of silence left a particularly deep impression on you? For example, what exactly happened? What was the atmosphere like? How long did the silence last? And what was going through your mind at the moment you chose to stay silent?

Interviewee: I remember once discussing the topic of faith with my partner. Many families abroad hold religious beliefs, like Christianity or Catholicism. The first time we touched on the subject, it was just a casual chat comparing religious practices in China and abroad. In China, there is freedom of belief; personally, my family leans towards Buddhism and visits temples to make offerings during holidays. To my partner, this looked like worshipping multiple gods, whereas he believes there is only one God. That’s where we disagreed. While we respected each other’s beliefs, a later discussion on the topic ended unpleasantly due to our differing views, and I ultimately chose silence. My main thought at the time was that our differing perspectives and cultural backgrounds made it impossible for us to approach the issue from the same angle; I felt that mutual respect was all that was needed. Oh, and does leaving a WeChat message on “read” without replying count? If he upsets me, I often just don’t want to reply to his messages. We had originally planned to visit a home furnishings store together over the weekend to buy a bookshelf for our new place. We’d even picked out the style. But then, on Thursday night, my mom suddenly called to say my dad had been hospitalized for observation due to high blood pressure and asked me to come home to check on him. I immediately told my husband we couldn’t go to the store anymore because I had to visit my parents. He got a bit annoyed right away and replied via WeChat: “We made these plans last week. You’re always like this, putting your family’s affairs first and changing our agreed-upon plans at the drop of a hat.” I saw that message just past 2:00 PM while I was killing time on my phone at work, and a wave of hurt and frustration washed over me. My dad was sick and in the hospital—was that something that could just be rescheduled? What did he mean by “always putting them first”? My fingers hovered over the input box; I typed out various things—“Of course I have to go back if my dad is sick,” “We can buy the bookshelf anytime,” “Why are you so inconsiderate?”, but I kept typing and deleting, typing and deleting, and in the end, I didn’t send a single word. My thought process was: typing wouldn’t get the point across clearly, and the more I said, the more agitated I’d get; if an argument broke out, I wouldn’t be able to focus on work for the rest of the afternoon. Plus, when emotions run high, you end up saying hurtful things that damage the relationship. It was better to say nothing for now and talk when we saw each other in person. So, I simply set his chat to “mute notifications,” turned my phone face down on the desk, and forced myself to concentrate on work. I couldn’t help checking my phone twice in the interim; I saw he’d sent two more messages—“Why aren’t you saying anything?” and “What do you mean by this?”, but I held back and didn’t reply. From just after 2:00 PM until he got home from work past 6:00 PM—over four hours. I didn’t send a single reply; I was essentially leaving him on “read” while staying silent and invisible. When I got home, I looked at him, but neither of us spoke first. I changed my shoes and went straight into the kitchen to cook, while he sprawled on the living room sofa without coming over to talk.

Researcher: During those four-plus hours, aside from fearing an argument might hurt the relationship, were there other thoughts going through your mind? For instance, were you perhaps acting out of spite, feeling he should apologize first? Or were you worried he might be jumping to conclusions?

Interviewee: There was definitely an element of acting out of spite. At the time, I felt it was completely unreasonable of him. My dad was seriously ill—not only did he show no concern, but he also blamed me for changing our plans. Why should I be the one to back down first? I just bottled it up, waiting for him to admit he was wrong. But I was also worried he might overthink things, wondering if I was giving him the silent treatment on purpose or if I didn’t care about him. Yet, the sense of grievance was so strong that I just couldn’t bring myself to speak first. Besides, I really didn’t know how to respond; if I said something harsh, we’d fight, but if I downplayed it, I’d feel resentful. So, I chose to avoid the situation—out of sight, out of mind—hoping it would just blow over. That was my habitual way of handling conflict: go silent to create a buffer and just ride it out.

Researcher: I see. That kind of behavior online—leaving messages unread or turning on “Do Not Disturb”—is essentially a way of hiding to give yourself some emotional breathing room. Is there another instance of silence that stands out in your memory?

Interviewee: There was one that happened in person. We were hosting a gathering for friends; he’d brought a few foreign colleagues over for dinner, along with two of their partners. During the meal, the conversation turned to going home for the Lunar New Year. He laughed and joked to the group, “Oh, her? She does whatever her parents say. Her word is her parents’ word. She’s still just like a little girl who hasn’t grown up.”

I was in the middle of picking up some food with my chopsticks when he said that; my hand froze, and I felt my face flush instantly. Everyone at the table was watching, including his boss. If I’d contradicted him right then and there, he would have been humiliated. You know how it is with Chinese culture. We value saving face for our partners in front of others, especially men; you’re expected to give them plenty of face in public. So, I just smiled, stayed quiet, and looked down to keep eating. For the rest of the meal, maybe another half-hour. I barely spoke. If someone asked me something, I’d just smile and nod or give a noncommittal “mm-hmm,” keeping up a polite smile but saying nothing of substance. After the guests left and he walked them downstairs, he came back in high spirits, saying everyone had enjoyed the meal. I just gave a curt “mm-hmm,” turned away to clear the dishes, and didn’t say another word to him. He didn’t notice at first and came over wanting to help with the dishes, but I stepped aside and said, “No need, I’ll do it myself.” He finally realized something was wrong, but by then I didn’t want to talk about it anymore. I just stayed silent; after tidying up, I curled up on the sofa and scrolled through my phone. I gave him half-hearted responses whenever he spoke to me, and I didn’t have a proper conversation with him for the rest of the night.

Researcher: What was your immediate reaction when you heard that comment at the dinner table? Aside from wanting to save face for him, were there any other emotions?

Interviewee: My first reaction was embarrassment, followed immediately by anger and a sense of grievance. I felt he didn’t respect me at all—speaking to me like that in front of others, making it seem like I had no mind of my own. But then I thought, maybe he was just making a casual joke without any malice; foreigners often tease people about things like that. Plus, making a scene in front of everyone would just be awkward and ruin the meal. You know, “don’t air dirty laundry in public.” If there’s a grievance, save it for home; you want to keep up an appearance of harmony in front of outsiders. But once we got home, that anger bubbled up again. Yet, I didn’t want to bring it up myself because I felt it would make me look petty or unable to take a joke. So I bottled it up, using silence to express my displeasure, waiting for him to realize something was wrong and apologize. But it turned out he didn’t even consider that possibility; he just thought I was tired.

Researcher: Those two scenarios are very specific and representative. Moving on to the third question: in what specific situations are you more inclined to remain silent? For instance, when facing accusations from your partner, discussing sensitive topics, dealing with cultural clashes, feeling exhausted, expressing defiance, protecting your psychological boundaries, or experiencing feelings of hurt or grievance?

Interviewee: There are quite a few situations. ;et me sort them out. First, I definitely go silent when there’s conflict in our communication. For example, when we’re chatting online and he upsets me, I don’t want to reply. Text messages lack tone and facial expressions. They feel cold and are so prone to misunderstanding. Plus, it’s easy to get carried away and say things without thinking when arguing via text. So, generally, if an unpleasant topic comes up or he says something that really stings, I’ll leave the message unread or just turn on “Do Not Disturb” and wait until we meet in person to talk about it. I’ve always felt that clearing things up face-to-face is infinitely better than typing; text-based arguments tend to escalate, so it’s often better to stay silent for a moment to let things cool down. Also, in front of others or in public, no matter what upsetting things he says, I never snap back right away. I choose silence. As I mentioned, it’s about saving face for him, but also for myself; if a couple argues in public, both parties lose face, and others just watch the spectacle. When he’s extremely agitated and pointing fingers at me in accusation, I also choose silence. For instance, if he gets angry about something and starts yelling at me, I absolutely won’t argue back; I just keep my mouth shut and listen, waiting until he’s finished venting before saying anything. I feel that when someone is in the heat of the moment, they won’t listen to a word you say. Arguing just adds fuel to the fire, so it’s better to wait until he’s calm to communicate. When the conversation turns to sensitive topics involving cultural differences, like the bride price, whose family to visit for the Lunar New Year, or how to raise future children, and we can’t see eye-to-eye at first, I’ll stay silent. For example, when we first got married and discussed the bride price, he viewed it as akin to a commercial transaction and couldn’t understand it at all; I tried explaining the social nuances and traditional customs for ages. But he wouldn’t listen, so I just stopped talking. I fell silent because I felt it was futile. Impossible to explain clearly, and a waste of breath. There are also times when I’m simply too exhausted to speak, like after a long day of overtime at work, when I come home too tired to utter a word. If he keeps chattering on about work or going on about his video games, I’ll just give perfunctory “mm-hmm” responses, essentially using silence to get by because I lack the energy to engage. Then there are times I use silence as a form of protest; if he does something that really angers me, I’ll deliberately stop talking to him and give him the cold shoulder to let him know I’m upset. Once, he went out drinking with friends and didn’t get back until 2 a.m. without telling me beforehand, and he didn’t reply to my messages. I was genuinely furious; for the next two days, I didn’t initiate any conversation. I was dismissive when he tried to talk to me and replied to his messages with just a word or two. I was intentionally using silence to punish him and make him realize he was in the wrong. Controversial content online counts as a form of this, too, I suppose. Sometimes he comes across YouTube videos or posts on overseas social media platforms discussing the differences between China and the West. Some of these are quite one-sided, bordering on smearing China, and he’ll forward them to me to ask what I think. In those moments, I usually don’t comment; I might just give a quick “like” or scroll past without arguing. I feel there’s no point in debating it. It leads nowhere and easily sparks a fight, so I simply stay silent and don’t take a stand to avoid trouble.

Researcher: I’ve heard you mention several different types of silence, some acting as a buffer, some involving restraint, and others serving as a form of resistance. How often does this situation—avoiding controversial online content—actually happen? Do you frequently disagree over things you see online?

Interviewee: Not often, though it does happen occasionally. He’s actually quite friendly toward China; it’s just that sometimes he stumbles upon odd videos and sends them to me out of curiosity. I generally prefer not to discuss these things with him because so much of it is deeply rooted in culture. I could explain for ages and he still might not get it, and it could easily turn into an argument. For instance, he once sent a video claiming it was strange that Chinese people don’t split the bill (go Dutch) but take turns treating each other. If I tried to explain the culture of social reciprocity and the exchange of favors, I could talk for half an hour and he still might not understand. So, I’ll just reply with “interesting” or send an emoji to move the conversation along, using silence to sidestep the issue rather than arguing over who’s right or wrong. It’s the same with social media updates; sometimes he posts things, complaining about traffic in China or the inconvenience of getting things done, and I’ll usually just “like” the post instead of arguing in the comments. After all, we have mutual friends, so saying too much isn’t ideal. A silent “like” suffices; even if I disagree internally, there’s no need to voice it.

Researcher: So, what is the primary reason you choose silence? Is it to avoid arguments, a feeling that it’s too hard to explain, a desire to maintain harmony, a need to save face for both parties, the influence of cultural values, the pressure of expressing yourself in a foreign language, or simply not knowing how to respond at the moment?

Interviewee: In some foreign cultures, direct communication and open confrontation are seen as healthy ways to resolve issues. In Chinese culture, however, harmony, the avoidance of direct conflict, and the principle of “seeing the truth without pointing it out” are considered virtues. The latter might perceive the former’s directness as “aggression,” while the former might view the latter’s silence as “avoidance” or “dishonesty.” My parents taught me to be gentle and sensible, to avoid arguments, and to prioritize harmony. They taught me not to go head-to-head when conflicts arose at home. So, my first reaction to conflict isn’t to rush in and argue; instead, I withdraw or go silent to maintain a semblance of harmony. Plus, I don’t want an argument to ruin my mood. It’s about diffusing the tension and calming myself down first. Sometimes, when I don’t know how to respond, I just need a moment to collect my thoughts. There are other factors, too, like a lack of linguistic nuance. English doesn’t express emotions with the same subtlety or intensity as Chinese; it relies more on underlying culture, humor, and subtext, which can dampen the desire to communicate. Many delicate feelings can only be accurately expressed in Chinese; they lose their meaning or nuance when translated into English. Even though I’m fine with everyday English conversation, when I’m actually arguing and emotions run high, words get stuck on the tip of my tongue. I struggle to phrase things correctly and worry that I won’t convey my meaning accurately, potentially leading to even deeper misunderstandings. So, I’d rather say nothing than risk making things more chaotic with explanations. Saving face is definitely a factor, too, something I mentioned earlier.

Researcher: You mentioned the pressure of expressing yourself in a foreign language; that’s a common challenge in cross-cultural marriages. Could you give a specific example? Was there a time when you wanted to explain something clearly but chose silence instead because of language barriers?

Interviewee: Yes, there was one instance that really stuck with me. We were arguing about the division of household chores. I was working overtime every day back then, getting home after nine, and still having to wash dishes and mop the floors, while he would just lie on the sofa playing video games. One day, I was absolutely exhausted and asked if he could share the housework. He replied, “Housework is a shared responsibility, and I do contribute. I took out the trash last week.” That made me a bit angry. I wanted to tell him, “Taking out the trash is just a tiny part of it; I do the vast majority of the chores. It’s not that I don’t want to do them. It’s just that work has been incredibly draining lately. I just want things to be fair and for us to share the load.” But when the words reached my lips, I got stuck. I didn’t know how to express those words, or that feeling of aggrieved helplessness, accurately in English. I was afraid that if I spoke up, it would sound like complaining and make him think I was making a big deal out of nothing. I stood at the kitchen doorway, dazed, trying to figure out how to phrase things, but in the end, I just said, “Never mind, it’s nothing,” and turned away to wash the dishes. It was that feeling of powerlessness—having so much to say but being unable to get it out, so I just bottled it up. At that moment, I really felt that if we were speaking Chinese, I could have hashed it all out clearly with him; but in English, I couldn’t make myself understood, so I just gave up and stayed silent.

Researcher: So, what impact does your silence have on your communication or your relationship? Does it help maintain harmony, defuse conflict, escalate tensions, leave your partner confused or hurt, give you both time to cool off, or create lingering resentment? Could you give an example?

Interviewee: Prolonged silence between my partner and me tends to create lingering resentment and escalate conflicts; neither of us feels happy, yet the silence prevents the root cause from being addressed. So, we made a pact: whenever a conflict arises, we must review the day’s events and resolve the issue before going to sleep. If problems are left to fester, they only escalate to a more serious level. For instance, there was a time he went out with friends, and a female colleague took a photo with him and posted it on social media. Seeing that made me feel really uncomfortable. However, I didn’t say anything to him because I felt that speaking up would make me seem petty or untrustworthy. So, I stayed silent for days and acted cold and distant. At first, he didn’t know what was wrong; he only realized it later after checking his social media feed. He came over to explain that she was just a regular colleague and it was just a group photo. We talked it through and cleared the air right then. That way, I didn’t have to keep up the silence—prolonged silence doesn’t solve the underlying issue; instead, it erodes the trust between us.

Researcher: So, you actively try to resolve conflicts caused by silence? Do you take the initiative to explain things? How do you usually “break the ice” and resume communication after a period of silence?

Interviewee: In the beginning, I certainly wouldn’t take the initiative to resolve things; I’d wait for him to come to me, waiting for him to strike up a conversation and give me a way to back down gracefully. Back then, I felt, “Why should I be the one to lower my head first?” Especially when I felt I wasn’t in the wrong, there was no way I’d speak up first. Breaking the ice was usually up to him; he might come over and ask, “Are you hungry? Shall I order takeout?” or “Want to watch a movie?” or hand me some fruit. I’d take the opening he offered, exchange a few words, and we’d make up. But that kind of reconciliation was only superficial. The problem wasn’t actually solved. The next time the same thing happened, we’d argue and go silent all over again. The real change happened after a fight during the Lunar New Year. It was a terrible argument. We were both quite hurt, so we sat down and had a serious talk that lasted all night. He said he couldn’t stand it when I kept everything inside and made him guess, because he couldn’t figure it out. I explained that I was used to bottling things up because I was afraid that speaking out would lead to a fight. After that, we agreed on some ground rules, and gradually, I learned to take the initiative in resolving conflicts. Nowadays, if I’m in the wrong or if I’ve gone silent for no apparent reason, I’ll take the initiative to break the silence and smooth things over. If the issue lies with him, I’ll wait until I’ve calmed down and then clearly explain my feelings, saying something like, “What you just said made me uncomfortable, which is why I stopped talking.” I don’t wait for him to guess what’s wrong like I used to. If we argue and one of us needs to cool off, we just say, “I need a moment to calm down.” However, the cooling-off period can’t exceed 24 hours; once that time is up, we have to sit down and talk it through. We also can’t just disappear or leave messages on “read” without replying; we have to let the other person know: “I’m a bit worked up right now, so I’m going to take some time to cool off and talk later.” For instance, if a WeChat conversation turns unpleasant, I won’t just go silent or turn on “Do Not Disturb.” Instead, I’ll send a message saying, “I’m feeling a bit emotional right now; let’s talk about this in person when I get home tonight,” and then stop the conversation there. That way, he knows I’m not intentionally ignoring him. I just need a buffer period so he doesn’t let his imagination run wild.

Researcher: Have you encountered any difficulties in sticking to these rules? For example, has there been a time when someone couldn’t help but break them or failed to follow through?

Interviewee: It was definitely hard to take the initiative and speak up at first, swallowing one’s pride isn’t easy. But I got used to it over time; I realized that bottling things up doesn’t help, and talking openly is actually better. Now, we rarely give each other the silent treatment for more than half a day; we resolve issues the same day they arise. We don’t let things drag on overnight.

Researcher: That’s great—the two of you have gradually found a dynamic that works for both of you. From a cultural perspective, what concepts within Chinese culture, or aspects of your upbringing, do you think most influence your choice to use silence in an intercultural relationship?

Interviewee: Chinese people place a huge emphasis on harmony—concepts like “harmony brings prosperity to the family” and “amiability brings wealth.” We strive for harmony and wholeness in everything. So, in an intimate relationship, I tend to view arguing as something negative that disrupts harmony; I try to avoid it if possible and just endure things. Silence is the most direct way to maintain harmony, even if it’s just a superficial kind of harmony. There’s also the culture of “tacit understanding”—that unspoken connection. Chinese people believe that someone who truly understands you knows what you’re thinking without you having to say a word, especially in intimate relationships. I used to think that since you’re my husband, you should understand why I’m angry and grasp my emotions; if I had to spell it out, it would lose its meaning. So, I would resort to silence, waiting for him to figure it out and come soothe me. But Westerners don’t buy into that; they think, “How am I supposed to know if you don’t say anything?” That’s where conflicts easily arise.

Then there’s the influence of my upbringing; that was exactly how my parents interacted. My dad wasn’t much of a talker, and neither was my mom. When they argued, they’d give each other the silent treatment, ignoring one another completely. After two or three days, my dad would finally break the ice and say something, and the issue would just blow over. They never sat down to talk things through openly; they’d bottle it up and then reconcile naturally. Growing up watching that, I assumed that’s just how married couples fight—that silence and the cold shoulder were normal, and there was no need to articulate everything clearly. So, when I first started living with my husband, I defaulted to that same pattern, never imagining he’d find it completely unacceptable. My early education played a role, too; teachers taught us to be humble and reserved, not to be too sharp or argumentative. Girls, in particular, were expected to be gentle and sensible—never to pick a fight, or else be labeled a “shrew.” Consequently, I never learned how to argue; whenever I faced conflict, my only response was to withdraw and stay silent.

Researcher: You mentioned that your parents’ dynamic had a profound influence on you. Did you realize that you were actually replicating your mother’s communication style?

Interviewee: I didn’t realize it at first; my husband pointed it out to me later. He said, “When you’re angry and go silent, you’re exactly like your mom.” I was quite surprised when he said that, but upon reflection, it was true. When my mom was angry, she’d stop talking; she wouldn’t even call my dad for meals. She’d just give him the cold shoulder until he initiated contact. I did the same thing: I’d go silent, freeze him out, and wait for him to come and soothe me. It was like I was cut from the same cloth. Eventually, I realized this approach wouldn’t work—that the dynamic from my parents’ generation wasn’t necessarily suited to our cross-cultural marriage. My mom’s method worked on my dad because he was Chinese and understood those unspoken rules, but it didn’t work on my husband; he simply didn’t get it. That’s when I started to change.

Researcher: The imprint of one’s upbringing runs deep, yet you were willing to actively adjust for the sake of the relationship. So, you feel that the frequency or manner in which you use silence in your cross-cultural marriage has changed, right? What are the specific differences between the beginning and now?

Interviewee: As I mentioned earlier, I now communicate proactively, so the time spent in silence has certainly decreased. In the beginning, when conflicts arose, I would choose silence to sidestep the issue, feeling there was no need for endless arguments to prove who was right or wrong, or I would bottle up my grievances, using silence as a form of protest while waiting for my partner to realize their mistake on their own. However, with his encouragement, I resort to silence much less often. I find myself going silent much less often now. Based on mutual understanding and respect, my partner and I are actively trying to integrate two cultures and embrace differences. We learn and compromise on dietary habits, celebrate festivals from both countries, and share personal demands and feelings candidly. Take dining, for example: in the past, I’d have my Chinese food and he’d have his Western food. We ate separately. Now, I’m learning to cook Western dishes for him, and he’s learning to use chopsticks and joining me for hotpot or luosifen (river snail rice noodles); even though he ends up sweating profusely, he’s still willing to try them with me. As for holidays, during Chinese New Year, we put up couplets and make dumplings together, video-call his parents to offer greetings, and I explain Chinese traditions to him; at Christmas, we decorate the tree, have turkey, and send gifts to my parents. Most importantly, we now try to be open and honest about our needs and feelings rather than bottling things up. I no longer wait for him to guess what I’m thinking, and he’s learning to pay attention to my moods. Sometimes he even picks up on things before I say a word. It’s really about making mutual concessions and meeting each other halfway.

Researcher: That’s a very noticeable change; it sounds like your dynamic has become much smoother. When did this shift begin? Was there a specific turning point?

Interviewee: It happened after a major argument. We sat down, calmed down, and really communicated; things felt better after that. We laid everything out on the table—our thoughts, cultural differences, and personal habits. After that conversation, we both made a conscious effort to change. I learned to speak my mind directly instead of holding things in, and he learned to be more observant of my emotions rather than being so straightforward or oblivious. Things gradually improved; it took about six months to settle into the way we interact now.

Researcher: Comparing your communication style with that of your Chinese friends (who are not in cross-cultural relationships), do you think your silence within your cross-cultural family differs from the silence they experience in their intimate relationships? What are the main differences?

Interviewee: There is a fundamental difference regarding the reasons and emotions behind the silence. With my Chinese friends, the silence feels more like a choice—perhaps stemming from anger, disappointment, or a need to cool off. It’s an emotional decision made after an argument, intended to signal the seriousness of the situation; it’s rooted in a conflict-resolution strategy or a sort of interpersonal “game” common to a shared cultural background. The silence between my partner and me stems from a fundamental communication barrier. When my partner makes a joke or references an allusion rooted in his cultural background, I may grasp the literal meaning, but I fail to connect with the underlying emotions he intends to convey. In the absence of a shared cultural context, this silence can be misinterpreted as a form of “cold violence,” a refusal to communicate, or a personality flaw—sentiments that can undermine basic trust.

Researcher: Regarding your silence within this cross-cultural family, is there anything we haven’t asked about that you feel is important or would like to add? Or is there anything you’d like to...

Interviewee: Regarding silence in a cross-cultural family, I think learning how to coexist with silence is crucial. The key isn’t to eliminate silence entirely, but to learn to understand and utilize it. Brief moments of silence can be beneficial. I also believe there are different types of silence. Sometimes, I find myself falling silent unconsciously. I don’t even realize what’s happening in the moment. I think it might also be linked to my personality; I’m an introvert, not particularly outgoing, and I often need solitude to recharge. So, at times, silence actually brings me a sense of calm. Other times, it might simply be that our communication styles haven’t quite meshed yet. That’s where active communication and mutual understanding come in. To maintain a harmonious relationship, I believe tolerance is the most important thing. Differences are an objective reality, but as long as both partners are willing to communicate and find common ground, they aren’t a problem. You have to respect those differences and each other’s unique traits, seeking common ground while accepting diversity. Respect is what matters. You’re never going to find two people who are 100% perfectly in sync; what truly counts is your willingness to proactively nurture and repair the relationship. You have to learn to express your emotions and feelings. Often, silence arises simply because we can’t articulate what we mean, so we bottle it up. If we could express our thoughts openly, many conflicts simply wouldn’t happen. Cross-cultural marriages certainly come with differences and conflicts, but they also offer unique joys. As long as both partners are willing to adjust and be tolerant for the sake of the other, things will work out well. Silence itself isn’t the problem; the real issue is an unwillingness to communicate or to work through differences together.

**I-15: (Female, US partner, with children, married < 3 years)**

Researcher: Alright, let’s officially begin. First question: Looking back at the time you spent together from when you met until you got married, what do you think were the most noticeable impacts of your cross-cultural backgrounds, such as differences in values, mindsets, and communication styles, on your relationship?

Interviewee: How should I put it? Honestly, when we were dating, the differences didn’t seem that significant; in fact, they felt quite novel. He’s American—very outgoing and outspoken—which was quite different from the guys I’d known before; I found that really attractive at the time. We met at an industry event; he hadn’t been in China long and his Mandarin wasn’t very fluent, but he was bold enough to speak up anyway, using a mix of gestures and guesswork. It was actually quite charming. Back then, our dates were all about dining out, movies, and having fun—nothing but happy times. We didn’t run into any real-world conflicts, so the cultural differences actually felt like a plus. It wasn’t until after we got married, and especially after having children, that the impact became truly apparent. Those differences shifted from a source of romantic novelty to practical, everyday issues we had to face. The most obvious difference was in communication styles. He is incredibly direct; whether he’s happy or unhappy, he voices it immediately. If there’s a disagreement, he wants to hash it out right then and there. For him, open communication is the top priority. I’m the opposite; I tend to put my emotions aside for a moment because I don’t want to cause a scene or damage the relationship by arguing too fiercely. I don’t think a marriage needs a winner and a loser. Then there were issues involving our extended families. He believes our nuclear family comes first and that neither set of parents should interfere too much. My parents, however, always wanted to lend a hand—coming over to stay or helping with the kids—which made him uncomfortable. He felt his boundaries were being crossed, leading to quite a few conflicts. There were also differences in mindset; he’s very logical and focuses on reasoning and determining right from wrong, whereas I sometimes prioritize attitude and feelings. For instance, during an argument, I care about his attitude and whether he’s considering my feelings. But he’s busy debating who was right or wrong and whether the logic holds up. Often, he’d feel like he’d won the debate, but I’d end up feeling even more upset.

Researcher: I see. It sounds like you were essentially on different wavelengths regarding what mattered most. So, when faced with a disagreement, his first instinct is to solve the problem, whereas yours is to avoid conflict. Is that right?

Interviewee: Yes, that’s pretty much it. I’ve always felt that when a couple lives together, it’s not really about who’s right or wrong; insisting on a “win-lose” outcome—where you might win the argument but lose the emotional connection—just doesn’t make sense to me. He sees it differently, though; he believes that keeping issues bottled up inside, rather than speaking up, is what truly damages the relationship.

Researcher: I see. Do you ever go silent during your daily interactions or when conflicts arise with your partner? If so, could you describe one or two specific instances that stand out in your memory? For example, what was happening, what was the atmosphere like, and how long did the silence last? Also, what was the main thought going through your mind when you chose to remain silent?

Interviewee: Oh, definitely. It happened quite a lot, especially during that initial adjustment period in our first year of marriage. There were times when I’d simply stop talking in the middle of a conversation and go silent. One instance that comes to mind was shortly after we got married, before we had children, and it was sparked by differences in our daily habits. We experienced a lot of friction in our day-to-day lives when we first started living together. That particular blow-up wasn’t caused by a single event; it was the result of many small issues piling up. There’s still a massive gap between our daily habits in a Sino-American household. I’m used to hot meals and warm water, but he can’t survive without salads and iced drinks for every single meal. I keep an early schedule, but he often stays up late hanging out. After countless fruitless attempts to compromise, I just stopped nagging him and accepted these cultural gaps quietly, sticking to our respective routines. It was a Friday, I think. We had agreed beforehand to cook at home that weekend and try out a new dish I’d learned. But that afternoon, he texted to say colleagues had invited him to a bar and he’d be back late. I was a bit annoyed, though I didn’t stop him; I just told him to try to get back early. In the end, he didn’t return until past 1:00 AM, reeking of alcohol; he walked in carrying a bottle of iced Coke and started drinking it right away. I hadn’t gone to sleep yet. I’d been waiting up, worried he might have had too much to drink and something bad might happen. But when he walked in and saw my stern face, he actually got annoyed first. He complained that I was giving him attitude again, asking if it was really worth getting so angry just because he’d gone out for a drink with friends. He even said that back when he was in the States, staying out until three or four in the morning on weekends was normal, and that I was being too controlling. Hearing that left me speechless. All the worry and the sense of grievance I’d been holding onto suddenly felt pointless, like nothing I said would matter. I just stood in the living room looking at him without saying a word. Maybe it was two or three minutes? I don’t recall exactly how long, but those minutes felt incredibly long. Then I turned, went into the bedroom, closed the door, and lay down on the bed, without saying a single word the whole time.

Researcher: What exactly was going through your mind during those two or three minutes? Were you angry, hurt, or thinking something else?

Interviewee: A mix of everything, really, but the main feeling was a sense of powerlessness. I suddenly realized I couldn’t get through to him. If I told him, “I was worried about you,” he’d surely say, “I’m an adult; I know what I’m doing—no need to worry.” If I said, “We agreed to have dinner together this weekend,” he’d say, “It was a last-minute invite from friends. What could I do? Can’t we just eat together next time?” It was like punching a pile of cotton wool; he simply didn’t grasp what mattered to me and instead saw me as being unreasonable. So, what was the point of saying anything? The more we talked, the more we’d argue until everyone was angry. It was better to say nothing at all. That’s when I had just one thought: forget it. There’s nothing left to say.

Researcher: I can understand that sense of powerlessness, feeling like the other person just can’t empathize with how you feel. Anything else? Interviewee: When our child was six months old, I had just quit my job to stay home and look after the baby; I was exhausted. It was the weekend, and I had hoped he could watch the baby for a while so I could catch up on sleep or tidy up the house. Instead, he’d made plans to go cycling with friends; he left early in the morning and didn’t get back until the afternoon. When he returned, I was holding the baby, trying to get him to sleep. The baby had been suffering from diarrhea and was incredibly fussy, and I’d been running on fumes for days. I said to him, “Could you please stop running off every weekend? It’s really exhausting looking after the baby all by myself.” And do you know what he said? He said, “Raising the child is something we both do, but I’m tired from work all week too. I need to relax on weekends. Cycling is my hobby—you can’t deprive me of my personal time.” He even claimed that in the U.S., it’s common for mothers to raise several children on their own without being as exhausted as I was, implying I was just being too delicate. I was on the verge of tears when I heard that, but I gritted my teeth and didn’t cry or say a word. I just sat on the sofa holding the baby while he stood there lecturing me. I didn’t look up or respond the whole time. I just stayed silent for about ten minutes. Finally, once the baby fell asleep, I put him in the crib and went to the kitchen to cook, still without saying a word to him.

Researcher: During those ten minutes of silence, how did he react? Did he keep talking?

Interviewee: He kept talking at first, explaining his point of view, saying I should have a life of my own and not focus all my attention on the baby and him. But when he saw I wasn’t saying anything, he probably lost interest and stopped, then went to take a shower. Those ten minutes felt agonizing; I felt both aggrieved and angry, but I just didn’t want to get into an argument with him. For one thing, the baby was sleeping, and I didn’t want to wake him up. For another, I felt that telling him “I’m tired” wouldn’t make him understand; he’d just think I was inefficient or bad at managing my time.

Researcher: Did the thought “He doesn’t understand or care about what I’m going through” cross your mind back then?

Interviewee: Definitely. I felt truly disheartened at the time. But on second thought, that was the environment he grew up in; his mother was a stay-at-home mom raising three kids, and his father rarely got involved in household matters, so he viewed that as normal. He has no concept of how exhausting it is for Chinese mothers to raise children or how much help they usually get from older family members. So, there’s no point in explaining it; silence is the better option.

Researcher: Right, I have a pretty clear picture of the situation now. Next, I’d like to ask: in what specific situations are you more inclined to remain silent? For instance, when facing accusations from your partner, discussing sensitive topics, dealing with a clash of cultural values, feeling exhausted, or trying to express feelings of grievance or anger—which of these scenarios makes you more likely to clam up?

Interviewee: As I mentioned earlier, things like daily habits and child-rearing philosophies—whenever it touches on those deep-seated cultural differences, I tend to go silent. I know these things can’t be changed just by arguing; his mindset, formed over twenty or thirty years, isn’t going to shift, and neither is mine. Arguing back and forth yields no results, so it’s better not to say anything. I also stay silent when he criticizes me, especially if his words are particularly hurtful or if I feel he simply doesn’t understand me. For example, if he accuses me of being too controlling or acting “delicate,” I don’t even want to defend myself. When someone is angry, the more you explain, the more fuel you give them to keep arguing, and things just escalate. So, I just shut my mouth and let him finish; once his anger subsides, things get better. I definitely clam up when I’m physically exhausted, too. If I’ve been up late for days looking after the kids or if I’m not feeling well, I simply don’t have the energy to argue or explain. When there’s a conflict, I just want to be left in peace; I don’t want to talk, even opening my mouth feels like too much effort. Then there are sensitive topics, like issues involving our respective families of origin or money, where I also tend to stay silent. For instance, his mother once came to stay with us for a month; we had very different lifestyle habits, and while I had issues with things, I didn’t want to tell him. If I did, he’d think I was looking down on his mom, so I just had to bottle it up. The same goes for the question of whose family we visit for the Lunar New Year. We argued about it once right after we got married, but later on, whenever the topic came up, I didn’t want to discuss it. I felt that arguing back and forth just hurt our relationship, and in the end, one side would have to compromise anyway. Finally, there are times when I need to express negative emotions, like when I feel deeply aggrieved or heartbroken, but I actually find myself unable to speak. Take the time the child was sick, for instance: I stayed up for two days and nights straight while he was away on a business trip. When he got back, he actually complained that I hadn’t taken good care of the child. I felt like crying right then, but I couldn’t say a word; I just looked at him in silence. I’ve always felt that when you’re truly devastated, you can’t speak. Anything you say just seems superfluous.

Researcher: You mentioned earlier that you tend to go silent when the conversation involves either side’s family of origin. Could you elaborate on that? For example, is it because you’re afraid of damaging the relationship with your mother-in-law, or because you feel he wouldn’t take your side anyway?

Interviewee: It’s a bit of both, I suppose. First of all, she is his mother, after all; if I said too much, he’d definitely be unhappy. If I put myself in his shoes and if he kept criticizing my parents, I wouldn’t be happy either. So, for a lot of minor issues, I just keep it to myself. I don’t want to put him in a difficult position or create awkwardness for everyone. Then there’s the fact that I know he’ll inevitably side with his mother. For instance, she often feeds the child using her own chopsticks. I find that unhygienic and mentioned it to him once, but he said, “That’s how I was raised, and nothing bad happened to me,” and accused me of being too fussy. After that, I stopped bringing up similar issues. Speaking up just leads to complaints, so it’s better to just keep a close eye on things myself. Besides, the older generation loves the child too. They just have a different way of showing it, so there’s no need to cause conflict over it. Also, many intergenerational conflicts can’t be resolved overnight; sometimes you just have to turn a blind eye and put up with it.

Researcher: I see. So, in many cases, your silence is actually a way of being considerate. You don’t want to put the other person in the awkward position of being caught in the middle.

Interviewee: Right, that’s part of it. I feel that when you’re a family, it’s best to just let things slide a bit; if you get too hung up on every little detail, life becomes impossible.

Researcher: What would you say is the main reason you choose silence? Is it to avoid arguments, a feeling that it’s impossible to explain things clearly, a desire to maintain harmony, a wish to save face (for yourself or the other person), the influence of cultural norms, pressure regarding verbal expression, or simply not knowing how to respond at the moment? Could you tell me which reasons you consider the most important?

Interviewee: Let me think... the most important reason? Well, the first one would definitely be wanting to avoid arguments. Couples shouldn’t fight all the time; too much arguing wears down the relationship. I’ve always felt that arguing really damages a relationship; I prefer to avoid it if possible—just hold back and let things slide, because taking a step back often opens up a wider perspective. For me, silence is a way to prevent arguments; if I don’t speak, a fight can’t start, and the conflict won’t escalate. Also, I often feel like things just can’t be explained clearly. It’s not really a language issue—his Chinese is actually quite good; he handles daily communication perfectly well and even uses idioms. But the underlying cultural logic is different. You tell him something, and what he understands is completely different from what you meant to convey. For instance, if I say, “A family should act like a family,” he’ll ask, “What does a family act like? Why do we have to be the same?” Take meals, for example: I think it’s a wonderful thing for a family to sit together in the evening and share a hot, home-cooked meal. But he thinks everyone should just eat whatever they want—no need to force it. You eat your Chinese food, I eat my salad—that’s happiness too. If I insisted on arguing about why we should eat the same meal together, he’d just think I was being unreasonable. So, often I’m on the verge of saying something, but when I imagine his reaction, I swallow the words back down; I feel like even if I explained it, he wouldn’t get it. It would just be a waste of breath. Plus, getting angry is a way of venting my emotions; sometimes when he upsets me, my attitude is: “You’ve upset me, so I simply don’t want to talk to you.”

Researcher: You mentioned earlier that the difficulty in explaining things stems more from cultural logic than language. I really agree with that. Was there ever a time when you tried to explain your perspective properly, only to find that he still couldn’t grasp it?

Interviewee: Oh, plenty of times. For example, I once had a serious talk with him about keeping each other updated on our schedules. I told him, “I’m not trying to control you or check up on you; I just want to know if you’re coming home late or going out for a work engagement so I’m aware and don’t worry about you. It’s just a basic sign that we care about each other. It’s not about interfering with your freedom.”

And what did he say? He said, “I’m an independent adult; I have the right to manage my own time. If you’re worried about me, you can send me a message. I’ll reply when I see it, but I’m under no obligation to report every single thing I do to you.”

You see? We just couldn’t get on the same wavelength. I was talking about caring and respect, while he was talking about independence and rights. We were coming from completely different perspectives. After that conversation, I never brought the matter up again. If he wants to talk, he talks; if he doesn’t, I don’t ask. Asking too much just leads to arguments.

Researcher: So, in many cases, silence is actually a coping mechanism that develops gradually after attempts at communication prove ineffective, right?

Interviewee: Exactly. It’s not like I wanted to be silent from the start. When we were first married, I did try. I’d share my thoughts, try to communicate when there was a conflict, and work on finding common ground. But after trying so many times, things always ended badly. Either he’d think I was being unreasonable, or I’d feel he was selfish and cold. After a while, I just got tired; I didn’t want to put in the effort anymore. Silence was simply less of a hassle.

Researcher: I see. Let’s talk about the impact of that silence. How does your silence affect your communication or your relationship? Does it maintain harmony and defuse conflict? Does it escalate tensions and leave your partner confused or hurt? Or does it give you both time to cool off—while perhaps leaving unresolved issues behind? Feel free to share some examples.

Interviewee: How should I put it? There are both pros and cons, though I feel the downsides might outweigh the upsides.

Let’s start with the benefits: the most immediate one is avoiding an escalation of conflict. For instance, if he’s furious and highly emotional, and I argue back, things just get worse. We end up saying hurtful things that really damage the relationship. If I stay silent and don’t engage, he eventually loses steam and calms down on his own, so the argument never really takes off. In that sense, silence does maintain a surface-level harmony and gives us both time to cool down; once the emotions pass, the issue might not seem like such a big deal anymore. But there are plenty of downsides, too. For one thing, he gets confused and hurt. He has no idea why I’m silent or what’s going on in my head. To him, my silence is more painful than an actual argument. Also, the conflict isn’t really resolved. It’s just suppressed, and over time, that can lead to lingering resentment. There are many things where I might stay silent and let the moment pass, but that doesn’t mean I’ve truly stopped caring about the issue deep down. Take the issue of the baby’s one-month celebration, for instance. I wanted to host a lively party and invite relatives and friends—that’s the custom where I’m from. But he didn’t see the point; he thought the baby was too young and that the fuss would be too hard on the child. Plus, he felt that accepting gifts created social obligations—a real hassle. We argued a bit at the time, but then I just went silent, and in the end, we didn’t hold the celebration. It’s been nearly a year, but thinking about it now still leaves a bit of a bad taste in my mouth. Yet, I’ve never brought it up again; there’s no use. It would just lead to another fight. It’s like a small knot. It doesn’t bother me day-to-day, but every now and then, when I think of it, it still pricks at me. Another downside is that I feel the intimacy between us fading over time. Because we bottle up so much instead of speaking our minds, it eventually feels like we have nothing left to say to each other. We only chat about the baby or what to eat—trivial daily matters—and rarely share deeper emotions or thoughts. It feels more like we’re just “sharing a life” rather than being close the way we were when we were dating.

Researcher: You mentioned that this creates an emotional knot. Do you ever worry that if too many of these little issues pile up, they might eventually blow up into a major conflict?

Interviewee: I do worry about that, actually. But then I think, isn’t that just what life is like? What couple doesn’t have conflicts? Whether it’s an intercultural marriage or not, making a life together requires compromise. You can’t expect to settle every single issue clearly in your favor or always get your way. Besides, I don’t know what would happen if things did blow up; it might turn out worse than the quiet, uneventful life we have now.

Researcher: Has there ever been a time when silence actually made things worse? Like a minor issue escalating into a big problem simply because of the silence?

Interviewee: Yes, there was. Shortly after we got married, it was his birthday. I had prepared a gift and cooked a whole spread of food. But he ended up working late without telling me; he didn’t get home until very late and had already eaten with his colleagues. I was incredibly disappointed. When he came in and tried to talk to me, I ignored him and just sat silently on the sofa.

At first, he didn’t know what was wrong, and I wouldn’t tell him. Eventually, he got angry too; he thought I was being unreasonable, giving him the cold shoulder over a small thing. We ended up in a silent standoff for three days, and we only made up because he finally broke the silence and spoke to me first. It was only later that I told him it was his birthday—an occasion I’d spent a long time preparing for. He even criticized me for not telling him directly, asking how he was supposed to know otherwise. You see, the situation was originally his fault, but because I stayed silent, it ended up looking as though I was the one being unreasonable. Looking back, if I had just said, “I prepared a birthday surprise for you today, and I’m disappointed you forgot,” we probably wouldn’t have had such a long silent standoff. But at the time, I just couldn’t bring myself to say it; it felt like I was fishing for him to feel guilty, which seemed rather pointless.

Researcher: I understand that feeling. I’d like to ask next: does your partner misunderstand your silence? How does he usually react to it? For instance, does he ask what’s wrong, respond with silence of his own, show impatience, or try to draw you out? And how do you view those reactions of his?

Interviewee: There are definitely misunderstandings, and significant ones at that. To this day, he thinks that when I’m silent, I’m angry or giving him the “silent treatment.” He simply doesn’t grasp that sometimes, my silence is just because I don’t want to fight. Whether we’re arguing, or I’m just too tired to talk, or even when my silence is actually a form of compromise or concession. He just doesn’t get it at all. Early in our marriage, whenever I went silent, he’d get really anxious; he’d immediately lean in and keep asking, “What’s wrong?” “Did I do something?” “Say something,”—he’d keep at it until I finally spoke. Back then, he was quite patient and would try to coax me out of it. But as it happened more often, he started getting a bit impatient. Now, if I go silent, he might ask a couple of times at first, but if I still don’t speak, he gets annoyed. He’ll say, “You’re doing this again. Can’t you just say what’s on your mind?” Then he’ll go off by himself and stop talking too, effectively turning it into a mutual silent treatment.

Sometimes, he tries to draw me out, like deliberately bringing up other topics or using the kids as a conversation starter to get me to respond. For instance, if I’m angry and staying quiet, he might say to the child, “Look, sweetie, why isn’t Mommy talking to us?” hoping I’ll jump in. I know he cares—otherwise, he wouldn’t ask. But I also feel like he doesn’t truly understand me. He thinks “just saying it straight” is the answer, but he doesn’t realize that sometimes you don’t speak up not because you don’t want to, but because it’s useless, or might even make things worse. He’s always saying, “How am I supposed to know what you’re thinking if you don’t say it?” But I feel that someone who truly cares about you should be able to read your emotions without everything having to be spelled out. Of course, I know it’s a cultural difference; he doesn’t have that ability to “read the room” or pick up on unspoken cues, so I can’t entirely blame him. Still, I feel a bit let down, like he doesn’t quite get me.

Researcher: Let’s talk about what happens after the silence. How do you usually “break the ice” and get back to normal communication after a period of silence or the silent treatment?

Interviewee: Most of the time, he’s the one who makes the first move. Americans tend to be more outgoing, after all. They can’t hold it in for too long. After half a day or a full day of silence, he’ll usually come over and start talking. He rarely apologizes directly or brings up the argument we just had; instead, he’ll find some other excuse, like asking, “What’s for dinner?” or “Where are the baby’s diapers?” or handing me something—just to strike up a conversation. As for me, I usually just take the opportunity to smooth things over; if he strikes up a conversation, I respond, and we naturally reconcile, as if the previous conflict never happened. Sometimes I’ll take the initiative to break the ice myself, like calling him over when dinner is ready, or handing him a piece of fruit or something, and that counts as making up. After all, a prolonged cold war between a couple isn’t right; someone has to be the first to yield. I’m not the type to be overly stubborn or proud; once things are mostly resolved, that’s enough for me.

Researcher: So, after breaking the ice, do you usually go back and discuss the topic that caused the silence in the first place? Or do you just move on and never mention it again?

Interviewee: The vast majority of the time, we just move on and don’t bring it up. I, for one, really don’t want to revisit it. I feel that since we’ve already made up, what’s the point of digging up old grievances? It would just lead to another argument and sour the atmosphere that we’d finally managed to smooth over. Sometimes he brings it up, like after we’ve reconciled, he might say, “About that thing earlier, I don’t think I was wrong,” or “Why were you angry earlier? Can you tell me now?” Usually, in those moments, I’ll just say, “Oh, that’s all in the past; let’s not talk about it,” and steer the conversation elsewhere. I really don’t want to rehash it; it’s exhausting, and it doesn’t solve anything. It just ends on a bad note.

Researcher: Do you ever feel that, if you don’t talk it through openly, the problem remains there, and you’ll end up arguing again the next time a similar situation arises?

Interviewee: I do realize that the problem hasn’t actually been solved. It’s just been suppressed for the time being. But I’m starting to shift my approach a bit now; things are going to change.

Researcher: Have you ever tried talking to him about this Chinese style of communication—the “silence” and “indirectness”? Like explaining the underlying cultural reasons?

Interviewee: I have. I told him that Chinese people value harmony above all else and dislike direct confrontation; often, silence is a way to preserve the bigger picture and save face for everyone involved. After hearing that, he said he could understand the cultural differences, but he couldn’t accept that kind of behavior in an intimate relationship. He believes that a couple should be completely open and honest with each other—that everything should be voiced, because keeping things bottled up only creates distance. You see, that’s just how it is: he can grasp the concept of cultural differences intellectually, but when it comes to actual daily life, he still can’t quite accept them. He still feels uncomfortable. So, all the explaining in the world doesn’t really help much; at best, it just keeps him from thinking I’m deliberately targeting him.

Researcher: I see. Let’s talk about this from a cultural perspective. What concepts within Chinese culture, or perhaps aspects of your upbringing, do you think influence your tendency to remain silent in an intercultural intimate relationship?

Interviewee: One factor is the concept of “valuing harmony above all else” that I mentioned earlier. Growing up, I constantly heard my family say things like “harmony brings prosperity to the household,” “taking a step back opens up a vast sky,” and “a moment of forbearance brings calm waters.” The idea was to avoid conflict with others—whether at home or out in the world, because arguing was seen as something terrible and shameful. This was especially true for girls; we were expected to be gentle, sensible, and reserved. We weren’t supposed to make a scene or shout, or else we’d be seen as ill-mannered or a “shrew.” If I got into an argument with other kids when I was little, my mother would scold me first, regardless of who was right or wrong, accusing me of being “immature” or “looking for trouble.” So, I developed a habit early on of backing down and holding my tongue whenever a conflict arose; I’d avoid speaking up if I could, just to steer clear of confrontation. Then there’s the culture of “saving face,” which plays a huge role, too. It’s not just about saving face for others; it’s about saving face for yourself. At home, for instance, if a disagreement got too loud, I’d worry the neighbors might hear and laugh at us. And in front of outsiders, it was even more important to preserve my husband’s dignity. I couldn’t let him lose face or be put in an embarrassing position. So, often, even if I had strong objections, I’d bottle them up and stay silent to avoid damaging our mutual dignity. There’s also the cultural emphasis on “tacit understanding”—the idea of a “meeting of minds” where you just know what the other person is thinking. Especially in intimate relationships, there’s this expectation that a true partner should understand you without everything needing to be spelled out. If you have to say it explicitly, it somehow loses its value. So, I often hope he’ll intuitively grasp my emotions and thoughts rather than having to hear me say them out loud. When he doesn’t pick up on it, I get disappointed, which makes me even less inclined to speak up, and we end up in a vicious cycle.

Researcher: What about your friends, the ones who also grew up in Chinese culture? Do they tend to go silent in their marriages as often as you do? Interviewee: Pretty much. It seems like all my married friends are the same way. Especially the women—rarely do you see them getting into screaming matches with their husbands every day. Usually, when a conflict arises, they just bottle it up or give each other the silent treatment for a few days, and then things naturally smooth over. Everyone feels that there’s no point in trying to reason everything out between a husband and wife; it’s better to just turn a blind eye and let it slide. In fact, people tend to think that couples who speak their minds openly and argue constantly are unhappy or taking things too seriously. So, it’s not just me; most people in our social circle handle conflicts this way. It’s just that in a cross-cultural marriage, this kind of conflict stands out more because the other person doesn’t play by those same rules.

Researcher: Right. Let’s talk about changes. Do you think the frequency or the way you use silence in your cross-cultural marriage has changed? How does it compare to when you were first together versus now?

Interviewee: There have definitely been changes—significant ones. Let’s start with the frequency. When we were first dating, there was almost no silence. Every day was happy, and we didn’t really run into conflicts; even if there was a minor tiff, we’d make up quickly. There was no need for the silent treatment. The first six months of marriage—the adjustment period—saw the most silence. New conflicts popped up every day, and we were constantly arguing; when we got too tired to argue, we’d go silent. Now, we’ve been married nearly three years and have a child, and the frequency of silence has actually dropped a bit. We’ve talked through many of our conflicts, and a lot of things just don’t trigger an emotional reaction in me anymore. For instance, him coming home late, or eating salads and drinking iced beverages—things that used to make me angry. I’m indifferent to them now. I don’t take them to heart, so naturally, I don’t go silent over them. Then there’s the change in how I use silence. In the beginning, silence was mostly out of spite or a way of acting out. It was like saying, “I’m mad, I’m ignoring you, come coax me.” It was emotionally charged and passive; the silence was meant to make him notice my feelings and try to appease me. Now, when I go silent, it’s because I clearly realize that talking about the issue won’t help and arguing won’t change anything. So, I consciously choose not to speak. It’s an active choice. It’s not out of spite or anger; I’ve simply accepted our differences with a calm mindset and don’t want to waste my breath. For instance, in the past, if he said he was going out to socialize, I’d get angry and go silent, waiting for him to coax me. Now, if he says he’s going out, I just give a simple “mm-hmm,” acknowledge it, tell him not to drink too much, and then go about my business. It doesn’t bother me at all. It’s not that I’m sulking; I just feel there’s no need to get angry or argue over it. Simply put, my silence used to be driven by emotion, whereas now it’s rational. Before, I was forced into silence; now, I choose it.

Researcher: So, do you feel that this silence—this conscious choice—is actually a form of self-protection?

Interviewee: Exactly. It is self-protection. I used to pour my heart out trying to communicate and find common ground with him, but I’d get rebuffed every time and end up feeling miserable. After that happened enough times, I learned to protect myself and stopped laying all my emotions and thoughts bare. If I don’t care what you say and don’t react, you can’t hurt me. It might seem a bit passive, but it really works. It makes life a lot easier for me.

**I-16: (Female, Australian partner, with children, married 4–10 years)**

Researcher: To start off, I’d like to ask for a general overview. Looking back at your time together over the past few years, what do you think has been the most significant impact of your differing cross-cultural backgrounds, such as differences in values, mindsets, and communication habits, on your intimate relationship?

Interviewee: The biggest impact is definitely our modes of expression. Specifically, our communication rhythms are completely different. He has a classic Australian personality: very direct. Whether the issue is big or small, he wants to hash it out and resolve it immediately; he absolutely refuses to let things drag on. I, on the other hand, prefer to calm down first, sort out my emotions and thoughts, and then communicate. For instance, if we’re both angry, I’d rather take a breather and wait for the tension to subside before talking; otherwise, the words we say end up being hurtful. But he doesn’t see it that way at all; he believes that “addressing issues openly in the moment shows respect for the other person, whereas dragging things out amounts to the silent treatment.”

Researcher: Could you give a specific example from early in your marriage where this difference in rhythm caused a noticeable conflict?

Interviewee: There were so many instances. We clashed over this almost daily during the first two years. One incident that really stands out happened in our second year of marriage, when we were living in Perth. We had invited some of his colleagues over for dinner. We’d finalized the menu three days in advance, and I’d already bought all the ingredients. Then, the night before the dinner, while we were prepping food in the kitchen, he suddenly said he wanted to swap the main course—fish—for steak, because one of his colleagues didn’t eat fish with bones. I was taken aback; I pointed out that we’d already bought the fish and changing it would be a waste, so maybe we could come up with another solution? But he wouldn’t hear of it. He insisted on drawing up a new menu right then and there, saying we had to decide immediately or we wouldn’t have enough time the next day.

Researcher: How did you feel in that moment?

Interviewee: I felt suffocated, like I was being forced to make a decision on the spot. It wasn’t that I disagreed with the change; I just didn’t see the need to decide that very second. We’d both been busy all day; I felt we could just rest for a bit and figure it out the next morning. There was plenty of time. But he couldn’t do that; he felt he “couldn’t sleep until the issue was resolved” and insisted on hashing it out right then. That was when I really clearly realized that our communication styles were just different. Sometimes I feel he’s too impatient and doesn’t leave any room for a buffer; he, in turn, feels I avoid issues, lack candor, and keep things bottled up. At first, this difference in pace caused a lot of friction. We’d constantly get upset over it, but after spending so much time together and adjusting to one another, we understand each other much better now.

Researcher: Aside from the pace of communication, were there other significant impacts?

Interviewee: Another factor was our differing understanding of “family boundaries.” For instance, I tend to view a couple as a single unit where decisions are made jointly, whereas he places greater emphasis on “individual independence”, believing that even spouses should have their own private space and decision-making power. However, this didn’t have as immediate an impact as our communication styles; it was something we worked through gradually. The core issues really came down to how we spoke, when we spoke, and how much we shared—those differences were the most pronounced.

Researcher: Could you describe one or two specific instances of silence that left a deep impression on you—perhaps during your daily interactions or when a conflict arose? I’d like to know what was happening, what the atmosphere was like, how long the silence lasted, and what was going through your mind when you chose to remain silent.

Interviewee: When an argument breaks out, my first instinct is often to just clam up. There was one time the kids’ school organized a field trip to the botanical garden. It was originally scheduled for Friday, but when I picked the kids up on Wednesday, the teacher mentioned in the group chat that rain was forecast for Friday, so the trip had been moved to the following Monday. That day, I got off work really late; after picking up the kids, I had to go grocery shopping, then cook and tidy up toys once we got home. I was busy until past ten o’clock before I could finally rest, and in the process, I completely forgot to tell him about the schedule change. Then, on Thursday evening, while he was filling out his work schedule, he suddenly looked up and mentioned that he’d already asked his boss for time off on Friday afternoon to volunteer for the field trip. That’s when it hit me. I’d forgotten to mention the change. I just casually said, “Oh, the trip got moved to Monday; I forgot to tell you.” He immediately shut his laptop and said, in a rather harsh tone, “How could you not tell me something so important beforehand? I’ve already made arrangements with my boss; changing the schedule now is a huge hassle.” I was standing by the dining table, still holding the dishcloth I’d used to dry the dishes, and I was suddenly left speechless. I just fell silent for about five minutes, I think. He walked over from the dining table, crouched down in front of me, and kept asking, “Why aren’t you saying anything? Do you think I shouldn’t have called you out on it? If you have something to say, just say it.” I still didn’t respond.

Researcher: What was going through your mind during those five minutes? Were you angry?

Interviewee: It wasn’t entirely anger; my thoughts were just a bit jumbled. On one hand, I felt aggrieved. I’d been busy all day picking up the kids, grocery shopping, cooking, and tidying the house, while he’d come home from work and just slump on the sofa watching TV, ignoring everything. And now, he was turning around and blaming me for forgetting something? But on the other hand, I knew I had forgotten to mention it; I was in the wrong, so there wasn’t much to argue back with. Most importantly, I knew that if I spoke up then, my tone wouldn’t be pleasant. Speaking while emotional would only make things messier and lead to an argument, and the kids were in the living room watching cartoons; it wouldn’t be good for them to hear that. So, I just wanted to stay quiet for a moment and let the feeling pass before saying anything.

Researcher: How was the silence broken?

Interviewee: Eventually, he stood up, sighed, and said, “Alright, I was a bit harsh. You should calm down for a bit,” and then he went to bathe the kids. By the time he finished settling them in and came back out, I had pretty much calmed down too. I sat down and explained the situation clearly to him; he understood, and we agreed to remind each other about things like that in the future. In the end, we both messaged his boss to reschedule, and the matter was put to rest.

Researcher: Are there any other instances of silence that left a deep impression on you?

Interviewee: There was another time involving money—probably the year before last. His younger sister wanted to get a new car but was short by 20,000 Australian dollars, so she asked to borrow it from him. He told me about it while we were lying in bed that night and asked what I thought. My immediate reaction was that I didn’t really want to lend it. It wasn’t that I was stingy; it was just that his sister had borrowed money once before. She’d promised to pay it back in six months but dragged it out for nearly two years without ever mentioning it. In the end, we only got it back because we needed the funds for a down payment on a house and he finally asked for it. But at the time, I didn’t know how to express that thought. Just think about it: on one hand, it’s his own sister. If I flat-out refused to lend the money, I’d look like a stingy sister-in-law who’s targeting his family. It could hurt their sibling bond. But if I agreed, I’d feel genuinely uncomfortable; surely, lending money requires some sort of concrete plan for repayment, right? When he asked, “Do you think it’s okay?” I just stared at the bedside lamp without saying a word for about seven or eight minutes. He found it odd and asked, “If you don’t want to lend it, just say so. Why aren’t you speaking?” I hadn’t organized my thoughts yet, so I just said, “Let me think about it.” The next day, I actually looked up a few words and carefully considered how to phrase things so I could express my concerns without sounding like I was criticizing his sister; only then did I have a proper conversation with him. In the end, we agreed on a clear repayment schedule with his sister and drew up a simple IOU, and the matter was resolved. That silence really wasn’t because I was angry; it was purely because my words couldn’t keep up with my thoughts, and I was afraid of saying the wrong thing.

Researcher: It sounds like the triggers for those two scenarios were quite different. So, in what specific situations do you tend to stay silent? For instance, when facing accusations, discussing sensitive topics, dealing with a clash of cultural values, feeling exhausted, or experiencing emotions like grievance or anger?

Interviewee: Mostly when I’m highly emotional, like during an argument, or when he misunderstands me or says something harsh. I’ll definitely go silent first. I know that when my emotions flare up, I tend to speak without a filter and say hurtful things I’ll regret later. So, I keep my mouth shut until I calm down to avoid saying something damaging. Another situation is when our cultural values clearly differ and there’s no objective right or wrong; I’d rather not speak then. For example, he always says, “Kids need to be independent and bear the consequences of their actions,” whereas I feel, “Kids are still young; parents should help out where they can.” That kind of difference isn’t about who’s right or wrong. It’s just down to our different upbringings. We could argue until dawn without reaching a conclusion, so I just stay silent; there’s no need to fight over who wins or loses.

Researcher: Could you give a specific example? Something other than the kids’ schooling—maybe a small, everyday matter? Interviewee: For instance, last year when my child started elementary school, he wanted the child to take the bus alone. He argued that he had taken the bus to school by himself at age eight and that it would foster independence. I felt it wasn’t feasible—not because I didn’t want the child to be independent, but because we live far from the school, the route requires a transfer, and it’s crowded during the morning rush hour. The child is only six; what if they missed the stop or encountered a bad person? We discussed this several times, but we just talked past each other—neither could convince the other. Eventually, I stopped arguing; when he brought it up, I’d just listen without saying anything. After all, getting up ten minutes early to drive the child to school didn’t really inconvenience me. Differences in mindset like that can’t be resolved by a single argument; talking too much about it just hurts the relationship, so silence is better. Then there are times when my language skills fall short, especially when dealing with nuanced or complex emotions, like feeling aggrieved, awkward, or slightly angry without actually blaming him. Articulating such ambiguous feelings takes a long time even in Chinese, let alone English. Sometimes, if I feel slighted, say, after a minor misunderstanding with a neighbor, I’d want to vent to him. But when the words reached my lips, I couldn’t find the right terms. It would come out as just “I’m unhappy,” prompting him to ask, “Why? Who upset you?” I couldn’t explain it clearly; the more I tried, the more muddled it became. In the end, I’d just give up and process it silently on my own.

Researcher: Aside from those three scenarios, are there others? For example, when you’re exhausted, do you also not want to talk?

Interviewee: Oh, definitely. If I’ve had a busy day at work and come home to look after the child and do housework, to the point where I’m too tired to even speak, and he tries to discuss something complex, I’ll only give brief responses. That’s a form of silence, too, I suppose. But that kind of silence stems from physical exhaustion rather than a deliberate refusal to communicate; it’s different from the silence during an argument.

Researcher: So, all things considered, what is the primary reason you choose silence? Is it to avoid arguments, a feeling that things can’t be explained clearly, a desire to maintain harmony and save face for both parties, or is it influenced by cultural norms or language barriers?

Interviewee: Most of the time, it’s to avoid arguments, though a desire to maintain harmony in the relationship plays a part as well. I’ve always felt that when two people live together, there’s no need to constantly vie for the upper hand; sometimes, a moment of silence to let things cool down allows many issues to simply blow over. It’s really a form of respect—respect for your partner and for the relationship itself—rather than just giving in to your temper and lashing out.

Researcher: You mentioned the influence of culture earlier. Is this perspective on silence something you’ve held since childhood?

Interviewee: Yes, it’s something I grew up with. Whenever the adults in my family argued, like my grandmother or my mother, they would often say, “Harmony brings prosperity to the family.” They believed that family members shouldn’t harbor deep-seated grudges, so it’s best for everyone to take a step back. I have a vivid memory of my parents arguing: my mother would never shout back at my father. Instead, she would quietly go about her housework without saying a word. She’d wait for his anger to subside before the two of them sat down to discuss the matter. Even back then, I felt that silence could defuse anger; the fiercer the argument, the more it damages the relationship, whereas staying quiet often prevents things from escalating. Of course, there’s also the factor of language barriers. I mentioned this earlier. For instance, if I want to explain something complex or express a subtle emotion, I worry that my English might fail me or I might not convey my meaning clearly, leading to misunderstandings. In those cases, I’d rather stay silent until I’ve figured out exactly how to phrase things. I’m especially cautious when the topic involves his family or friends; I’m afraid that saying the wrong thing might make me appear rude or seem like I’m targeting them, so silence feels like the safer option.

Researcher: Does “saving face” play a role? For example, do you choose silence to avoid conflict when you’re out in public?

Interviewee: Yes, that’s definitely a factor. If we disagree while shopping at the supermarket or dining with friends, I certainly wouldn’t voice it right then and there; I’d stay silent and wait until we got home to talk about it. I believe that even as a married couple, you should show each other respect in public. Arguing in front of others looks bad. He doesn’t seem to share that mindset, though; he’ll voice his disagreement immediately, regardless of who’s around. Sometimes I find it embarrassing. That’s likely a cultural trait, too.Chinese people value the idea that “family scandals shouldn’t be aired in public,” meaning conflicts should be resolved behind closed doors.

Researcher: So, how do you think this silence has affected your communication or your relationship? Does it help maintain harmony and defuse conflict, or does it escalate tensions and leave him feeling confused or hurt? Perhaps you could share some specific examples.

Interviewee: In the short term, silence definitely helps cool things down. Now that we’ve been together a while, he knows that my silence doesn’t mean I’m ignoring him. It just means I want to calm down a bit, so he doesn’t keep pestering me like he used to. For instance, if we have a minor spat, I might stay silent for a few minutes until I’ve composed myself; then, when I speak to him calmly, the issue gets resolved quickly without turning into a huge argument. But there are downsides, too. Sometimes, if the silence drags on, he feels like I’m “emotionally withdrawing” or “refusing to engage,” and he interprets it as the silent treatment. I remember one instance vividly: we couldn’t agree on whether to get a pet for our child. He wanted to get a cat to help foster a sense of responsibility, but I thought it was too much of a hassle—scooping litter, feeding, vaccinations—and I worried that our child’s interest would be short-lived, leaving us to do all the work. After talking for a long time with no resolution, I just didn’t want to discuss it anymore. For the next two days, I didn’t bring it up, and I was rather distant in our everyday conversations. The following evening, he sat me down for a serious talk. He said, “If you don’t want to get a pet, just say so; there’s no need to give me the cold shoulder.” He thought I was using silence to protest or give him the silent treatment. That’s when I realized the disconnect: I thought I was simply “putting the issue aside for now” to avoid an argument, but to him, it looked like a refusal to communicate, like I was sulking. Later, I explained my concerns to him. We agreed to try getting a hamster first; if our child could take good care of it, we’d consider a cat later. That settled the matter. Silence is a double-edged sword: it can protect the relationship by avoiding head-on clashes, but it can also create distance. If you don’t speak up, the other person starts guessing, and all that speculation can easily lead to a rift. This is especially true in cross-cultural marriages, where people interpret silence differently, making misunderstandings more likely.

Researcher: Was there ever a time when silence actually had a positive effect, or even brought you closer together?

Interviewee: Yes, there was. It was probably last year. He ran into some trouble at work—a project he was in charge of went wrong, and his boss chewed him out. He came home looking terrible; he walked in, sat on the sofa, and didn’t say a word. In the past, I might have pressed him with questions like, “What’s wrong? What happened?” But that day, I didn’t ask. I simply went to the kitchen, poured him a glass of ice-cold beer, grabbed some of his favorite nuts, and set them down in front of him. Then, I sat beside him and watched the game without saying a word. We sat there for maybe half an hour or so before he voluntarily opened up about work—mentioning the immense pressure and his fear that the project might fall through, which could jeopardize his promotion. I didn’t offer any grand advice that day; I just listened and nodded occasionally. Later, he told me how grateful he was that I hadn’t pestered him with questions, allowing him some quiet time to himself. That’s when I realized that silence can sometimes be more effective than words; you don’t always need to get to the bottom of everything—quiet companionship is a form of empathy, too.

Researcher: So, how does your husband usually react when he misunderstands your silence? Does he ask you directly, go silent himself, get impatient, or try to draw you out? What do you make of these reactions?

Interviewee: He asks me. And then we start talking. We work things out through conversation. After we make up following a tiff caused by my silence, I always explain to him that my silence doesn’t mean I don’t care or that I’m giving him the cold shoulder; it’s just that when emotions run high, I need a little time to sort out my thoughts before talking. After explaining this a few times, he’s gradually taken it to heart. Now, if I suddenly don’t talk, he wouldn’t press me for answers right away; he’d give me some space.

Researcher: Next, I’d like to discuss this from a cultural perspective. What concepts in Chinese culture, or perhaps aspects of your upbringing, influence your choice to remain silent in your cross-cultural marriage? Things like “harmony brings prosperity,” “silence is golden,” or “loose lips lead to trouble”?

Interviewee: They have a huge influence. Take “loose lips sink ships” or “silence is golden.” I’ve heard these sayings my whole life. My grandmother was a very traditional Southern woman; she was gentle her entire life and rarely lost her temper. If there was a conflict at home, she’d be the first to yield. She didn’t say much, but she was incredibly thoughtful in her actions. She often told me that a girl should be composed and steady, not boisterous or loud. She’d say, “Better to speak less than more, and better to speak well than to speak a lot,” because talking too much makes it easy to make mistakes or offend people. My parents were the same way; their generation viewed silence as a sign of good character. Women, in particular, were expected to be patient and accommodating rather than assertive. We never had screaming matches at home; we’d process things in silence and make up after a couple of days. So now, when I face family conflict, my instinctive reaction is to stay silent first, then communicate once tempers have cooled.

Researcher: Aside from family values, were there other experiences while growing up that reinforced this mindset?

Interviewee: School played a part, too. I remember back in elementary and junior high, the girls who talked a lot or talked back to teachers were often labeled “frivolous” or “flighty,” while the quiet girls, the ones who spoke little but got things done. Were praised for being “sensible” and “well-behaved.” I subconsciously internalized the idea that speaking less was the right, proper thing to do, and that talking too much was actually a bad thing. Then there’s the concept of “saving face,” which also has a deep influence. Chinese people value leaving room for maneuver and preserving dignity, whether dealing with family or outsiders. Even between husband and wife, people avoid saying things that are too harsh or final, and certainly avoid arguing in public. Like I mentioned earlier, if a disagreement arises when we’re out, I’ll stay silent and save the discussion for when we get home. I simply don’t want my partner to lose face or give outsiders a spectacle to laugh at. These might seem like small, everyday concepts, but they really do shape the choices you make every time. Researcher: So, do you think these ideas clash a bit with the reality of a cross-cultural marriage?

Interviewee: They definitely do. Because he just doesn’t operate that way. If I try to save face by staying silent, he completely misses the point; instead, he thinks I’m holding back or being insincere. So, often you can’t just act on instinct—you have to make a conscious effort to adjust. You have to explicitly spell out things that are taken for granted in our culture; otherwise, it’s just a waste of breath.

Researcher: Looking back at all these years together, do you think the frequency or way you use silence in the marriage has changed? How does it compare to when you were first married?

Interviewee: Yes, the change is quite noticeable. When we were first married, I would go silent very easily. Part of it was the language barrier. I lacked confidence and didn’t know how to express myself. Another part was the vast cultural gap; I felt like I couldn’t get through to him on many issues, so I’d just stop talking altogether. I used silence a lot back then, sometimes over the smallest things, and I’d bottle it up for ages. The longer we’ve been married, the more I’ve consciously tried to control that tendency; I’ve realized I can’t just clam up the moment I feel uncomfortable. I know I have a tendency to lapse into habitual silence, but now I control my silence length and avoid prolonged reticence. For example, when we are discussing something important, I’ll tell him, ‘Give me five minutes to think,’ so he doesn’t feel like I’m just avoiding the issue.

Researcher: How well does this method work?

Interviewee: It works really well. At first, it felt quite unnatural; whenever emotions ran high, my instinct was just to clam up. I’d hurriedly blurt out, “Give me five minutes,” and it sounded really abrupt. But over time, it’s become a habit. I can even joke about it now; if we disagree on something, I’ll raise my hand and say, “Time out. I’m switching to ‘cool-down mode’ and will be back in five minutes,” and he’ll smile and say, “Sure, I’ll time you.” We rarely get into arguments over silence anymore because he knows I’m not refusing to speak. I just need a moment to sort out my thoughts. Plus, the periods of silence are much shorter now. Before, I might not want to talk for half an hour or even the better part of a day; now, it’s usually just ten or fifteen minutes before I speak up on my own once I’ve gathered my thoughts. I don’t bottle things up over little issues anymore, either, like if he forgets to take out the trash or doesn’t wash the dishes properly. I just say it directly. I don’t do what I used to do, worrying that speaking up would upset him, so I’d just do the chore myself and stew in silent resentment. Overall, the silence is more manageable now; it’s not an instinctive act of avoidance, but a deliberate choice to take a breather.

Researcher: How does your silence in a cross-cultural relationship compare to that of your Chinese friends who are in same-culture relationships? What are the main differences?

Interviewee: I’ve noticed that for my Chinese friends with Chinese husbands, their silence is more like a shared understanding—both parties know exactly what the silence means without needing an explanation. For instance, I have a close friend who once had a fight with her husband and gave him the silent treatment for three days—neither of them spoke to the other. On the third day, he cooked a spread of her favorite dishes and brought them to her, and just like that, they made up. They didn’t have to bring up the argument at all; they both knew the issue was water under the bridge. It’s a sort of unspoken rapport, and mutual understanding without words. Their silence might signal “let’s drop this,” “I’m angry, come coax me,” or “I agree but don’t want to say it outright,” and the partner just “gets it” without needing an explanation. But that simply doesn’t work in a cross-cultural relationship. Nine times out of ten, my silence gets interpreted by him as “refusing to communicate,” “you don’t love me anymore,” or “you want to break up.” We’re just not on the same wavelength. So, I have to explain myself—either before or after a period of silence—by saying something like, “I’m feeling a bit emotional right now and need to calm down before we talk.” Otherwise, he’s bound to let his imagination run wild. In other words, within a cross-cultural context, silence in itself is meaningless; you have to articulate its purpose, to “translate” it into a form of communication the other person understands, or else it just leads to misunderstandings.

Researcher: Do you think their silence might sometimes be a tactic used within the relationship? Like using silence to get the other person to back down or compromise?

Interviewee: That happens too. I have friends who stop speaking when they’re angry, giving their partner the silent treatment, and waiting for them to offer comfort or an apology. It’s actually a way of acting coy or registering a protest, and the partner usually understands the game. But if I tried using silence to protest, my husband wouldn’t pick up on it at all. He’d just think, “Oh, she needs to cool off,” and leave me alone. He wouldn’t come over to comfort me. So that tactic doesn’t work on him; it’s far more effective to just say, “I’m angry, and I need you to comfort me.”

Researcher: One last question: regarding your silence within a cross-cultural family, is there anything we haven’t asked about that you feel is important and would like to add?

Interviewee: I’d really like to emphasize that not all silence is hostile. Especially in cross-cultural marriages, where there are so many linguistic and cultural differences, speaking more can sometimes lead to more mistakes and misunderstandings. Sometimes, simply being quiet together, cooking a meal, or watching a movie without talking, allows you to feel each other’s intentions; silence becomes a form of empathy. My husband and I have that kind of rapport now. Sometimes, after a tiring day at work, we’ll just curl up on the sofa and look at our phones. We don’t feel the need to make conversation, yet it doesn’t feel awkward. It’s actually very comfortable. But the prerequisite is that both parties understand the meaning of that silence. If one person sees silence as companionship while the other sees it as distancing, then silence creates a gap rather than understanding. So, the key isn’t whether or not you speak; it’s whether the two of you can reach a consensus on what the silence means. Don’t let yourself be bound by your own cultural habits. It’s not that our habit of silence is bad, but in a cross-cultural relationship, you can’t expect the other person to instinctively understand what your silence signifies. If something needs to be said, say it, even if you don’t express it perfectly or come across as a bit blunt; it’s still better than leaving the other person to guess. For instance, if you need to cool off, just say, “I need five minutes.” It’s a simple thing, yet it can prevent a lot of misunderstandings. Also, there’s no need to force yourself to change overnight. Silence isn’t a flaw; it’s a self-protection mechanism we’ve developed over a lifetime, so don’t invalidate yourself just because the other person doesn’t understand it. It’s about gradually finding a rhythm that works comfortably for both of you. After all, whether a marriage is cross-cultural or not, at its core, it’s about two people accommodating and understanding each other, right?

**I-17: (Female, UK partner, with children, married 4–10 years)**

Researcher: Looking back at your time together, what do you think were the most significant impacts of your differing cross-cultural backgrounds, such as values, mindsets, and communication habits, on your intimate relationship?

Interviewee: I think the impact was quite substantial; our values were truly worlds apart. The main thing was the huge difference between our family backgrounds. You could say I came from a lower-class family back home—that was just how I was born. Of course, my family has been in business for the last decade or so, and our material circumstances have improved a lot, but their mindset remains stuck in that lower-class background; they’ve never really moved past it. As for him—well, his family isn’t exactly wealthy either, but I feel like he actually “regressed” a bit after living in the UK. When I look at the photos his parents sent him from his childhood, I’m amazed at how many countries he visited back then. He even went on cruises. It seems like they had a very comfortable life growing up. Now that they’re adults, most people in his social class are just ordinary folks—his cousins and older brother, for instance, work as teachers; they’re just regular people. Still, his childhood and mine were like two different worlds; different upbringings lead to different values. Those values were actually what attracted me to him in the first place. When I first arrived in Thailand, I felt that foreigners really knew how to live like human beings. They didn’t seem anxious, and they knew how to appreciate what they had instead of constantly obsessing over what they lacked. Of course, this varies from person to person; not every foreigner is like that. Another thing is that they don’t engage in excessive social comparison, like worrying about which handbag I’m carrying or what clothes I’m wearing. He really knows how to enjoy the present moment, and that was incredibly appealing to me. That’s what brought us together.

Our mindsets were very different, too. But now, I think I’ve been influenced a lot, almost subconsciously, because I feel that many foreigners have such a healthy, positive mindset. Of course, that has a lot to do with cultural background and the sense of security instilled in childhood. We tend to lack that sense of security, right? Our social safety nets aren’t as good as theirs, so we don’t have that same feeling of being secure. From a psychological perspective, they say a person’s life path is largely set by the age of seven—that the mindset, habits, and personality formed before then are pretty much fixed. Actually, there isn’t a huge amount of change after the age of seven. I used to think it was impossible. After all, my own life changed drastically after I turned thirty. But looking back now, especially after having children. I realize that while I kept telling myself, “Oh, I don’t want to be like my parents; I’ll never yell at my kids,” and tried to parent differently than I was raised, those deep-seated traits from my upbringing would sometimes inadvertently surface. We are truly heavily influenced by our families of origin. I’ve come to feel that perhaps 60% to 70% of one’s life is determined before age seven, with later influences playing a smaller role. He’s lived in the UK since he was forty, and things have gone well for him there in every respect; I think that’s given him a strong sense of security.

Looking at the bright side, I think it’s because he hasn’t endured much hardship; he grew up in a relatively stable environment. His life has been quite steady. He’s not like me; I’ve changed jobs frequently and run various small businesses. Those ups and downs shape a person differently.

Communication habits are so important. I used to complain about him constantly. After we had kids, as the time commitment for parenting grew, leaving us with no time for ourselves, and expenses rose, all I could see were his flaws. But now that the kids are in school and I’ve slowed down, I realize we actually complement each other in some ways. In a sense, he acts as a mirror for me. There’s a book called The 5 Love Languages. Communication really matters. Answering this question made me realize something: our dating days were wonderful, but after marriage and children—when time became scarce and the cost of living went up. I focused entirely on his shortcomings. Yet, reflecting on it now, I see he has strengths too. For one thing, he is very willing to communicate. If something comes up, he keeps asking questions. He really engages. Sometimes, when I’m angry, I don’t want to answer, but looking back, I see that communication is absolutely vital. The Bible says, “Do not let the sun go down on your anger.” My point is that I don’t think there should be a rift between a husband and wife; any issues or knots in the relationship need to be resolved promptly. If you resort to the silent treatment, you gradually lose the desire to communicate, and the distance between you just keeps growing. So, whether it’s an intimate relationship, a bond with parents, or a partnership, communication is crucial. He is the type who speaks up and asks questions whenever something comes up, whereas I’m not really one for communicating. I used to think, “Don’t you get it? Do I really have to spell it out for you?” But now, approaching forty, I’ve realized that men and women really are different. If you don’t say it, he genuinely won’t understand. That’s why I believe expressing yourself is vital. Don’t feel awkward about it. Whether it’s about money, sex, or any other emotional need, you have to tell him directly; your needs matter, so don’t make him guess. Making others guess just drains your own energy. I find that most Westerners prefer communication and tend to avoid the silent treatment, though, of course, it varies from person to person.

Researcher: Do you ever go silent during your daily interactions with your partner or when a conflict arises? If so, could you describe one or two instances that left the deepest impression on you, such as what happened, the atmosphere at the time, and how long the silence lasted? What was going through your mind when you chose to remain silent?

Interviewee: Oh, this is a bit similar to the previous question. Regarding the thoughts behind the silence, sometimes I’d think, “Why did I marry you and live with you 24/7 if you don’t understand me?” I think a common mistake people make is desperately wanting those closest to us—parents, children, partners—to understand us. But I’ve come to realize that, aside from yourself, no one can truly understand you—not even your parents. So, I feel that for the sake of harmony in a relationship, we sometimes need to let go of the expectation that others will fully understand us—that they will truly empathize or grasp our perspective. That doesn’t mean the love has vanished; it’s just that, at times, it is genuinely difficult for others to truly empathize. For instance, he and I grew up in completely different environments; there are things we Chinese people think about that he simply can’t relate to or “get.” That said, it doesn’t matter if you don’t understand; you haven’t walked a mile in my shoes, so it’s not crucial. Later, I realized that even my parents couldn’t understand, though, of course, that wasn’t the main point. What matters most, and what I believe to this day, is that the most intimate and important relationship one has is the one with oneself. External things aren’t that significant. As for moments where I stayed silent? There were so many. I used to be someone who disliked communicating; I’d always want others to guess what I was thinking, leading to plenty of silent moments. He would constantly follow me around asking, “What’s wrong? Why?” Sometimes I’d think, “You’re just pretending not to know,” but later I realized he genuinely didn’t have a clue. It probably transcends nationality; some men back home are like that, too. Now, I feel it’s essential to learn to love yourself. What does that mean? It means expressing your needs, that is loving yourself. Whether the other person truly understands, pretends not to, or is just feigning ignorance, it doesn’t matter; you just state it directly. If something makes you uncomfortable, you have to speak up.

Researcher: In what specific situations are you more inclined to remain silent? For instance, when facing accusations from your partner, discussing sensitive topics, dealing with cultural clashes, feeling exhausted, or experiencing emotions like grievance, helplessness, sadness, or anger?

Interviewee: In the past, I stayed silent because I still held onto some expectations. Now, if I stay silent, it’s because I think, “Forget it. There’s no point in saying anything because you wouldn’t understand anyway.” Even if you were Chinese, or someone born and raised in the exact same place as me, you still couldn’t truly understand me. Let alone a foreigner; communication and mutual understanding are simply impossible unless you’ve actually walked the path I’ve walked. Of course, it’s possible I don’t understand him, either. Nowadays, I feel whether others understand me isn’t that important. Knowing what I want and how I should live my life. I think that matters more than expecting others to understand me, whether it’s a partner or anyone else. You can remain friendly, but don’t place overly high expectations on others. I think a lack of understanding is perfectly normal, even with a partner.

Researcher: What are the main reasons you choose silence? For example, avoiding arguments, feeling it’s impossible to explain clearly, maintaining harmony in the relationship, protecting your own or the other person’s face, cultural influences, the pressure of verbal expression, or simply not knowing how to respond at the moment?

Interviewee: I think it’s a mix of all those things, a little bit of everything. If there are issues I don’t know how to respond to right away, or if I’m feeling emotional, I choose not to communicate at that moment. I wait until I’ve calmed down so I can speak in a composed tone rather than one laced with anger. That’s why I opt for a temporary pause. Sometimes, later on, I realize the matter isn’t important anymore. It’s in the past, so I don’t bring it up. There are also limitations regarding language. Now, I stick to communicating about practical matters, like what I’m doing tomorrow or what tasks I have, and just state those things directly. As for emotional needs, those deeper things. I feel it’s okay if they aren’t met [by my partner]. I’ve shifted that deep need for understanding and expression into other areas, like exercise, fitness, or the things I’m learning and the books I’m reading.

Researcher: What impact does your silence have on your communication or relationship? Does it help maintain harmony, defuse conflict, escalate tension, leave your partner confused or hurt, provide time to cool off, or create lingering resentment? Could you give an example?

Interviewee: As I mentioned, I don’t really need that deep, emotional level of communication. However, surface-level communication—the practicalities of daily life—is essential. It’s not just about communicating; it’s about ensuring neither person feels shortchanged. Marriage isn’t as grand or lofty as one might imagine; it’s really just a partnership. That’s why communication is so important. You have to communicate clearly and transparently. Things like who helps with the kids, who does the dishes, and how much is spent this month versus next month—these need to be laid out clearly. If neither person feels like they’re getting the raw end of the deal, life goes much more smoothly. You shouldn’t be shy about discussing these things, including money. We’re a family, but my husband and I are also long-term partners. I once saw an interview with a musical duo who worked together very happily. When asked why they hadn’t disbanded after so many years, they said, “We always split the earnings fifty-fifty; the division is crystal clear.” I think marriage is just like that. When we interact—since I’m not one to open up. If he asks me something when I’m angry, I often just can’t be bothered to explain it to him. He harbored some resentment; at the time, he felt I had a terrible temper and wondered why I had changed so much after marriage. Yet, I felt incredibly aggrieved and exhausted, and I was the one raising the child. Now, I try to love myself more. That’s why I believe communication is key—you have to let him know exactly what it is that makes you angry. Open communication is absolutely vital—whether it’s verbal or physical. You should just say what’s on your mind. It’s even easier to be direct with a foreigner, since they tend to be more straightforward in their communication. You can be direct with Chinese men, too, but I feel we Chinese people tend to have a stronger sense of pride. You have to be mindful of that and perhaps adjust your approach, whereas with a foreigner, you can be more blunt.

Researcher: Do your partners ever misunderstand your silence? How do they usually react to it. Do they ask you about it, choose to stay silent themselves, show impatience, or try to draw you out? What is your take on these reactions?

Interviewee: Of course, there are misunderstandings. He’s a very straightforward guy, a “straight man” in the stereotypical sense, so he doesn’t get it at all. Once, while we were dining with friends, he accused me of bullying him—of using “cold violence” [the silent treatment]. He found my silence—my refusal to communicate—scary. Admittedly, at first, I was reluctant to communicate, though now it’s more that I just can’t be bothered. That said, I’ve been making adjustments; I started going to church a few months ago, and that’s helped me change my ways. I realized my behavior wasn’t right. We need to show respect to everyone. A teacher at the church said something that really made me rethink things. He asked, “Where is the child?” and I replied, “The foreigner took him.” He asked, “Doesn’t the foreigner have a name? Why do you keep calling him ‘the foreigner’? You should use his name; even if you dislike someone, you still owe them respect.” That really resonated with me. I realized I needed to change this aspect of my life. No matter who the person is—parents, children, or a partner—respect is the bare minimum. Also, we shouldn’t let too much emotion cloud our communication; the goal should be to resolve the issue at hand. I truly feel that attending church these past few months has been a huge help to me. Now, whenever I approach a situation, I try to resolve the issue first before expressing my emotions. This is crucial, because otherwise, I’m the one who ends up at a disadvantage. Foreigners tend to communicate quite calmly. We Chinese people often lead with our emotions, which can make actual communication impossible. That used to be me. I’d think, “I’m so angry right now; do I really have to talk to you?” But I’ve adjusted my approach. I prioritize solving the problem first, then dealing with the emotions.

Researcher: From a cultural perspective, do you think certain concepts in Chinese culture, such as “harmony in the family leads to prosperity,” “think thrice before acting,” “silence is golden,” “loose lips cause trouble,” or “be cautious in speech and action,” or perhaps your upbringing, influence your choice to remain silent in cross-cultural intimate relationships?

Interviewee: These concepts really run counter to the Western mindset. Foreigners don’t have that many preconceived notions; they don’t even really have the concept of “respecting the elderly and cherishing the young” in the traditional sense. They always view everyone as equals. I think that’s actually quite right. They don’t assume a superior tone just because they’re an elder. They’ll say, “Could you please do this for me?” If you pour them a cup of tea, they’ll say “Thank you” and even add your name. It feels genuinely comfortable—the way they speak is very pleasant. Also, they tend to focus on the issue rather than the person. In other words, even if we’re family—parents or siblings—the problem still needs to be addressed. It’s not a case of letting things slide just because you’re my parent or older brother; a mistake is a mistake. They value family too, but they believe issues need to be resolved. They don’t subscribe to ideas like “harmony brings prosperity” or “don’t air dirty laundry in public.” I don’t think they really follow the “think thrice before acting” rule either; most foreigners seem to be people of action who enjoy life in the moment. From what I’ve seen, many foreigners express their emotions immediately rather than bottling them up. Right? They live much more relaxed lives. Back home, we have so many rules and social conventions, but they don’t have those constraints.

Researcher: Do you feel there have been changes in the frequency or manner of your use of silence within your cross-cultural marriage? How does the present compare to when you first started?

Interviewee: So, today’s topic is entirely about silence, right? There has been a huge change in the frequency of my silence. At the very beginning—before I met my partner—my behavioral and communication patterns were entirely Chinese-style; they were essentially copies of my parents’ ways. But after living in Thailand for so many years and being married to Jie-ge, I’ve undergone a lot of subtle, gradual changes. I’ve learned how to communicate, yet I don’t obsess over it. If something can be discussed, I’ll talk it over with you. However, regarding deep, inner communication, I’ve always felt that, ultimately, you live with yourself. Communicating with yourself—finding inner consistency and balance—is what truly matters. Communication with others, specifically with a partner, is simply a means to live together better and more harmoniously.

If a person truly wants peace and joy from within, it really comes down to how well they get along with themselves. That said, my communication with my partner, parents, and children has improved significantly compared to when I first arrived in Thailand eight years ago. I talk to my parents now; I express thanks, love, gratitude, and longing to friends—things I wasn’t very good at expressing before. Eight years ago, I really didn’t know how to express these feelings. Now, when I return to China and leave again, I hug my parents and friends, tell them I miss them, and make frequent video calls to say “I miss you”—things I never would have said in the past. I’ve come to realize that expression is crucial; both feeling loved and expressing love are vital. That’s something I’ve learned over these past few years.

Researcher: Regarding your use of silence in a cross-cultural family, is there anything we haven’t asked about that you feel is important and would like to add?

Interviewee: I think silence... why is it called “silence” [in Chinese, the character implies “sinking”]? Because if you remain silent for too long, things really do sink down and get buried. That happens in any relationship if you stay silent for too long. It’s the same with friends, isn’t it? As we grow up, if two friends are apart and go a long time without contact or checking in, the silence eventually sets in, and that silence often signals the end of the connection. Yet, intimate relationships are even more vital. I believe that timely expressions of affection can boost the other person’s happiness, and that applies to our parents, too, doesn’t it? Whenever I express my feelings to my parents, I see them smiling—smiling continuously. That’s when I realized just how important this is. It’s the same with having children of our own; we all love our kids—that goes without saying, right? “Of course I love you”, but you still need to actually tell them you love them, or give them a kiss. It really makes a difference. If you haven’t been influenced by a culture that encourages this, or if you’ve simply stayed within a domestic context, why not give it a try starting today?

Break the silence with your partner. Tell them, “I love you so much,” “You’re amazing,” or “I’m so happy, so incredibly happy, to be with you.” Sometimes, this is simply an act of kindness. When you say these things often enough, you start to truly believe them yourself, and your partner does too. Your relationship will become increasingly harmonious; let’s not be stingy with our praise.

**I-18: (Male, Australian partner, with children, married 4–10 years)**

Researcher: Looking back at your time together, what do you think were the most significant impacts of your differing cross-cultural backgrounds, such as values, mindsets, and communication habits, on your intimate relationship?

Interviewee: The most obvious difference was in how we handled conflict. It took some getting used to at first. My wife is the type of person who, when a problem arises, wants to sit down, talk it through clearly, and come up with a plan. She tends to ask things like, “What exactly is the problem? How do we solve it? What should we do next time?” She shifts into problem-solving mode quite quickly. I wasn’t like that initially. I felt that when a conflict occurs, the first priority should be addressing emotions. For instance, if you’re exhausted or already angry, I don’t think it’s the right time to rush into a rational discussion. I wanted to let the other person know, “I understand why you’re upset,” before discussing the issue itself. There was one instance involving who would pick up the kids. We both had work that day, and I had to work late unexpectedly, so I was absolutely exhausted when I got home. She immediately took out a piece of paper to map out everyone’s availability and responsibilities for pick-ups and drop-offs, wanting to set a fixed schedule. I was so tired that my first reaction wasn’t to look at the plan; I just wanted her to ask, “Are you really tired today?” She, however, thought I was being too emotional—wondering why I kept talking about my feelings when the issue could simply be solved with a plan. Later, I realized she didn’t actually disregard my emotions; she was simply accustomed to expressing care by solving problems. She felt that by fixing the issue, she was showing she cared. My understanding of care, on the other hand, was more about companionship and listening. After we’d adjusted to each other over time, she would sometimes start by asking, “Are you really tired today?” and I would say, “Tell me your plan first, and I’ll listen.” That worked much better.

Researcher: So, you essentially developed a new way of communicating?

Interviewee: Exactly. It wasn’t really a case of one person changing the other; it was more about mutual adjustment. She learned that I sometimes need an emotional buffer, and I learned that she needs a clear, concrete response. I used to think she was too rational, but now I see that her rationality actually helps me. She used to think I was being evasive or beating around the bush, but she eventually realized I wasn’t trying to avoid the issue. I just needed a little time to process things clearly. Researcher: Do you ever go silent during your daily interactions with your partner or when a conflict arises? If so, could you describe one or two instances of silence that left a particularly strong impression on you? For example, what happened, what the atmosphere was like, how long the silence lasted, and what was going through your mind when you chose to remain silent?

Interviewee: Yes, it happens quite often, actually. I didn’t really think of it as “going silent” back then; I just felt like I needed some quiet time. There was one instance where we had a significant disagreement about parenting styles. Specifically, our child was going through a phase of being hyperactive at night, and their sleep schedule was inconsistent. My view was that we shouldn’t have a rigid daily schedule; instead, we could observe the child’s state and make gradual adjustments. Her perspective was different. She wanted to establish a stable routine and believed the child should know exactly what to do and when.

We ended up arguing about it. She felt I was too casual and thought that if we based everything on the child’s mood at the moment, it would lead to a lack of rules. I felt she placed too much emphasis on rules. After all, a child isn’t a machine. Things got pretty heated, and I realized I didn’t know how to continue the conversation. It wasn’t that I lacked ideas, but I felt that if we kept going, it would devolve into personal attacks—things like “you don’t understand the child” or “you’re being too domineering.” So, I went to the study and spent about half an hour by myself.

Researcher: Why did you choose to go to the study instead of continuing the discussion with her?

Interviewee: Because I knew my emotions were running high. I tend to get anxious easily, especially when it involves our child. If I had kept talking, I likely would have just vented my emotions. I also felt that cultural differences were being magnified in that moment; we likely had different underlying ideas about what makes a “good parent.” Trying to convince each other right then and there would have been pointless. I just wanted to calm down first and reflect on why she was so insistent, what she was worried about, and what my own thoughts were.

Researcher: What exactly were you thinking about during that half-hour in the study?

Interviewee: At first, I wasn’t thinking very rationally. I was still a bit angry when I first went in, thinking, “Why doesn’t she understand me?” It took about ten or fifteen minutes before I slowly started to consider what she was truly worried about. I later realized that her insistence on a routine wasn’t simply about controlling the child; rather, she believed that structure provided the child with a sense of security. Once I saw it that way, my emotions subsided. I also re-examined my own stance. I didn’t entirely disagree with having a routine; I just didn’t want it to become a rigid requirement. So, later on, I told her that I wasn’t against structure. I just wanted to allow for some flexibility. She, in turn, explained that she didn’t want to control the child, but was simply worried that we were being too casual.

Researcher: So, in the end, that silence actually had a positive effect?

Interviewee: Yes. We fell out over parenting styles. I then stayed alone in the study for half an hour. Cultural divergence calls for calm reflection. Silence gives both sides breathing space and prevents hurtful words spoken out of impulse, which makes subsequent communication much smoother. If I hadn’t walked away and had kept arguing, I think the conversation would have likely shifted away from parenting styles and turned into issues like “You don’t respect me” or “You always think you’re right.” I think this is a form of adjustment.

Researcher: In addition to parenting issues, are there any other silence experiences that particularly impressed you?

Interviewee: There is one thing I remember clearly. That time, it was about money. My parents gave me a sum of money at that time. They gave it to me on their own initiative and said they could use it to buy some things for their children. I felt at the time that this was my parents’ intention for their children, so I kept it to myself without telling my wife immediately. Later, when she found out by chance, she had a huge reaction. She felt that I had not kept the family finances transparent and believed that even if the money at home was given to me by my parents, I should explain it to her. I felt very aggrieved at that time. Because in my understanding, it was not me secretly taking the family’s money, but a little kindness from the parents to their children. I think she took this too seriously. She asked me why I didn’t tell her and then said things like, “Why do you think you don’t need to tell me this kind of thing?” and “If we are a family, why do you need to hide things from me?” In fact, her core may be “transparency,” but what I heard at the time was “don’t trust me.” I wanted to explain at the time, but I felt that the more I explained, the more confusing it would be. Because the matter itself is actually not complicated, but what I want to express is: first, I have no malice; second, my parents just want to express their feelings for their children; third, I understand why you feel that I should tell you. Putting these three meanings together, I found it difficult to explain them in Chinese, let alone English. So I just sat on the sofa and was silent for about 40 minutes without saying anything.

Researcher: Were you completely unresponsive during those 40 minutes?

Interviewee: It’s not like I didn’t respond at all. She asked me, and I might say, “I know,” or “That’s not what I meant,” but I didn’t elaborate. She was also in a bad mood at the time, and I don’t think she could listen to the explanation at that time. If I keep emphasizing “You misunderstood” at this time, she may think that I am shirking responsibility. So I thought let her release her emotions first. After she calms down, I will explain everything from beginning to end. Because I didn’t explain it in time, she became more sensitive to some of my expenses for a while. For example, when I buy something, she will ask what it is; if it involves a relatively large expenditure for the family, she will make a point of discussing it first. I actually felt aggrieved at the time and thought, “Do you really not trust me anymore?” Later, when my parents came to our house and mentioned the money while chatting, my wife realized that it was indeed given to me by them on their own initiative and their original intention was to buy things for their children. She later apologized to me and said that she had indeed misunderstood it at the time. After that, I just felt that I couldn’t remain silent when I was misunderstood. Silence can sometimes relieve emotions, but if the other party has formed a wrong judgment and you don’t explain it, it will become a fact. After that time, I paid more attention. If she clearly misunderstood me, I would still wait for her to stabilize, but I wouldn’t leave it at all without explaining.

Researcher: In what specific situations do you tend to remain silent? For example, when facing accusations from your partner, discussing sensitive topics, conflicting cultural concepts, feeling tired, or expressing feelings of grievance, helplessness, sadness, anger, etc.

Interviewee: I think the most obvious thing is when the emotion is particularly strong but you can’t explain it clearly. For example, I feel aggrieved and angry, but I don’t know how to express this emotion accurately, so I won’t say it for now. One time, my child encountered something unpleasant in kindergarten. I was very distressed and angry at the time. I actually want to discuss this with my wife, but I don’t know how to express it. Because if I say, “I think you should pay more attention to the kids,” it sounds like I’m blaming her. If I say “I’m angry,” she might ask “Why are you angry?” I don’t know how to explain all the feelings of “distressed, worried, angry, and a little helpless.” So I didn’t say much that time. I was afraid that my expression would end up being an accusation. Because in a cross-cultural family, sometimes what you are thinking about is very complicated, but when you say it out, there may be only one sentence left. Especially since English is not my native language. If I were in a Chinese environment, I might be able to say a lot of subtle things. But when expressing myself in English, I worry that my word choice is not accurate. There are many similar situations. For example, when she makes a mistake at work. Sometimes she encounters unsatisfactory things at work and is in a bad mood when she comes home. I actually wanted to comfort her, but I didn’t know what to say. Chinese people’s habit may be to say “It’s okay, it will pass” and “Don’t think too much.” But sometimes she doesn’t like this too-quick comfort, and she wants someone to really listen to her. If I didn’t know how to respond, I would sit next to her and keep her company. She later learned that I was actually accompanying her by sitting there quietly, not being indifferent.

Researcher: You just mentioned the issue of language expression many times. How much of a role do you think language barriers play in your silent behavior?

Interviewee: I think it plays a big role, especially when the emotions are complex. Of course, it’s okay to do simple things; they’re all simple. But if it involves emotions, such as “Why do I feel wronged?” “I actually understand you, but I feel uncomfortable,” and “I don’t disagree with you, I just feel that the way you speak puts me under pressure,” this kind of thing is more difficult. There are many expressions in Chinese that cannot be matched by one word. So sometimes I would rather stay silent first, because I feel that speaking out now may cause greater misunderstandings. One time she was very busy at work and forgot to bring a file for me. I actually felt a little aggrieved at the time, because that document was quite important to me. She immediately apologized and said she was busy and forgot. Logically speaking, I should say “it doesn’t matter,” but in my heart, it doesn’t really matter. I understood that she was very busy, but I also felt that I was quite disappointed at the time. It’s hard to express this emotion clearly in Chinese. Because if I say, “Why did you forget again?”, she will feel that I am blaming her; if I say “It doesn’t matter”, it is not entirely true. So I was silent for a while. Later, when I thought about it clearly, I told her: “I know you didn’t mean it. I was just a little disappointed at the time. I didn’t want to blame you.” Then she understood. For me, silence often means not not having what I want to say, but rather sorting it out in my mind. Especially when it comes to feelings, I will first think about “what do I want to express” and “how to express it without causing her to misunderstand.”

Researcher: What is the main reason why you choose to remain silent? For example, not wanting to quarrel, feeling that the explanation is unclear, maintaining relationship harmony, protecting oneself or the other party’s face, the impact of cultural concepts, pressure to express language, not knowing how to respond temporarily, etc.

Interviewee: Actually, there are several reasons. The first is not wanting to argue. I don’t like that kind of situation where the more you talk, the more intense you get. The second is that the explanation is unclear. Especially when it comes to cultural differences, I sometimes need to sort out my thoughts first. The third is to protect both parties. I feel that if you say something in anger, you may regret it later.

The fourth is the education received since childhood. My mother has told me since I was a child that I should speak in a measured manner and not say everything in front of people I don’t know well. This habit has actually been formed. So now, when I have a conflict with my wife, I will subconsciously consider, “Would it hurt someone if I say this?” But I also know now that excessive silence is not good either. I used to think that I could avoid problems by not saying anything, but now I know that some things must be said, and I just have to choose the right time and method.

Researcher: What impact does your silence usually have on your communication or relationship?

Interviewee: Some are good and some are bad. The good thing is that it can cool down. For example, on the parenting issue I just mentioned, if I had continued to argue with her, we might have ended up having a serious argument. After half an hour of silence, everyone calmed down and was able to discuss the matter itself. The bad thing is that if the silence is too long, the other party will guess. Especially my wife, who is not as accepting of long periods of non-response as her Chinese friends. She will think, “Why don’t you speak?” “Are you angry with me?” I used to think she thought too much, but now I know it’s a cultural difference.

Researcher: Can you give me another specific example of misunderstanding caused by silence?

Interviewee: She once discussed with me whether to move. She said that although the place where she lives now is good, she wants to change to one with more space. I actually think my current house is pretty good, and I don’t have a particularly strong interest in moving. But at that time, I didn’t directly say “I won’t move”; I just said “It’s pretty good to live here now.” She later felt that I might not like the house, or that I might not want to discuss it with her. Some time later, she asked me again. I just said, actually, I am not against you. I just think the cost of moving is too high, and there is no big problem with this house now. She later said: “Then why didn’t you tell me then?” I said: “Because I don’t think this matter is urgent and I want to think about it again.” Then she understood. There was a lot of misunderstanding about my silence in the beginning. She used to think that silence meant I was angry, or that I just didn’t want to communicate with her. Later, she gradually learned that I was influenced by Chinese culture and was relatively reserved, so my silence did not necessarily mean rejection. Now she usually doesn’t push me right away. For example, when I am silent, she may ask: “Do you need some time, or do you not want to talk about this?” If I say “I need some time,” she will let me stay by myself for a while. This helped me a lot because if she kept asking, I wouldn’t know what to say. I think this is good because she’s actually adapting to my culture as well. She may have thought before that “everything between husband and wife should be discussed immediately,” but now she has accepted that some things need to be buffered. Of course, I am also adapting to her. Now I will not be silent for hours like before, but I will take the initiative to tell her: “I’m not angry, I just don’t know how to express it now.” This way she won’t be anxious.

Researcher: From a cultural perspective, what concepts in Chinese culture do you think of, such as “Everything thrives when everything is at home”, “Think before you act”, “Silence is golden”, “Too many words lead to mistakes”, “Be careful with your words and actions”, etc.Which will affect your choice of silence in cross-cultural intimate relationships?

Interviewee: It definitely has an impact. The education I received since childhood is to speak in a measured manner and respect others. Especially in front of unfamiliar people, you can’t say everything. My mother often tells me to think before speaking and not to overestimate what I say out of impulse. This habit has actually entered my personality now. When getting along with my wife, I sometimes wonder if this statement is sensitive to her. I’m afraid of touching her cultural taboos or making her think I don’t respect her, so when I’m not sure, I might not say anything. Once, she was talking to me about an issue in Australian political history and asked me what I thought. I actually don’t have a particularly clear view, and I don’t know much about Australia’s historical background. I was afraid that I would say the wrong thing, so I didn’t say much. She later asked me, “Why are you so quiet?” I said, “Because I don’t know what to say.” She told me that it didn’t matter, I could just tell her whatever I thought, and there was no need to hold back because I was worried about saying something wrong. After that time, I slowly discovered that she might be more accepting of different opinions than I thought.

Researcher: Do you feel that the frequency or manner in which you use silence in cross-cultural marriages has changed? What’s the difference between the beginning and now?

Interviewee: It has changed a lot. When I first got married, my silence was more like an escape. For example, if I discuss some issues that I don’t want to face, I won’t talk about them. Especially when it comes to future planning, I may find it troublesome, so I don’t respond for now, hoping that things will go away on their own. It’s different now. Now, if I am silent, I usually do it to think.

For example, when we discuss which school our children will choose in the future, I won’t answer immediately. I might consider the distance, teaching quality, and family schedule first, and then consider how to discuss it with her. So the current silence is more like an intermediate stage than an end point. It used to be “I don’t want to talk about it, so I won’t talk about it.” Now it’s “I need to think about it, and then we’ll talk.” I think this change is actually quite important.

Researcher: As a male, do you think your gender role will affect your silence in marriage?

Interviewee: I think a little bit. Chinese men may be taught from an early age to take responsibility and not to show too much vulnerability. Sometimes you feel really uncomfortable, but you think, “What’s there to say?” For example, when I’m under high pressure at work, I won’t take the initiative to tell her a lot because I think I should handle it myself. But sometimes she would ask: “Are you under a lot of stress recently?” I would say “no” at first. Later, as she got to know me better, she realized that when I said “no”, I might actually mean yes.

Researcher: Comparing the communication styles between you and your same-sex Chinese friends, especially male friends with whom you have a good relationship, what do you think is the difference between your silent behavior in a cross-cultural family?

Interviewee: The difference is quite obvious. When I have a conflict with a Chinese friend, if I don’t say anything, he can usually understand that I am trying to calm down. Especially for male friends, everyone may not keep asking. For example, if we argue about something and I stop talking later, he may send me another topic a few hours later, and then come back to talk about the previous matter two days later. But my wife is different. She will feel that silence itself is a message. She may be thinking: “Are you angry?” “Do you not want to talk to me?” “Do you have a problem with me?” So she needs an explanation. In the past, I would think, “Why must I explain?”, but now I think these are two different understandings of silence. To me, silence can mean “I won’t say anything yet.” To her, silence may mean that “something is wrong.” So I tell her now: “I need a moment, but I’m not saying no to you.”

Researcher: Will you take the initiative to tell your partner why you are silent now?

Interviewee: Yes, and much more than before. For example, if I’m very tired from work today, I might just say: “My mind is a little confused today, and I want to be quiet for a while.” If it’s because we just had an argument, I will say: “I’m a little angry now, and I don’t want to say hurtful things when I’m angry. Let’s talk about it after I calm down.” After she hears this, she won’t think that I’m trying to bully her.

Researcher: Do you think this is a common rule formed after your cross-cultural adjustment?

Interviewee: Yes, I think so. We have actually formed a habit now: silence is okay, but there must be an explanation. For example, “I need half an hour,” “I’m too tired today,” “I need to think.” This way, the other person knows what’s going on.

Researcher: If you were asked to differentiate now, what kind of silence do you think is an escape, and what kind of silence do you think is a positive communication strategy?

Interviewee: I think the key is to see whether you come back after being silent. If you remain silent and leave the problem there without facing it again, that is escaping. If you stay silent to calm yourself down and then come back to talk, that’s strategy. Another difference is whether you let the other party know. Of course she would be worried if I left without saying anything. But if I say, “I’m in a bad mood right now. Give me some time and we’ll talk about it tonight,” then she knows I’m not throwing the issue away.

Researcher: Do you think if you were both Chinese, your silence would still be as frequent and obvious as it is now?

Interviewee: I think probably not. Because there are many things among Chinese people that don’t need to be explained. For example, I told my friend, “Forget it.” He might realize that when I say “forget it,” I don’t mean it literally, or perhaps he understands that I simply don’t want to keep arguing at that moment. But my wife might not necessarily see it that way. That’s the thing about cross-cultural marriages: they turn things that wouldn’t normally require an explanation into things that do. It made me realize that while I used to view my silence as perfectly natural, for her, it might have seemed like something else entirely.

Researcher: How do you think the process of adjusting to each other over the years has affected the stability of your relationship?

Interviewee: I think the biggest change is that we no longer insist on the other person communicating exactly the way we do. When we first got married, I felt she should understand my silence, while she felt I should state things clearly right away. Now, we both know that the other person’s style might differ from our own. Sometimes she wants to discuss something immediately, and I can tell her I’m not ready yet; other times I need silence, and she knows that doesn’t mean I don’t love her. I feel this kind of understanding actually makes the relationship more stable.

Researcher: Do you view your silence more as a form of emotional management or relationship management?

Interviewee: It’s a bit of both. First and foremost, it’s about managing my own emotions. If I’m angry, I need to calm myself down first. But at the same time, it’s about managing the relationship. I know that if I say something harsh in the heat of the moment, I might hurt her. Couples, in particular, know exactly what matters most to each other. When people are angry, it’s easy to deliberately target those sensitive points. I don’t want to do that. So, sometimes silence acts as a way for me to hit the brakes.

Researcher: How do you usually resume the conversation after a period of silence?

Interviewee: It’s simple; it doesn’t have to be anything formal. Sometimes I’ll say, “Let’s go back to what we were talking about earlier,” or I might start by asking, “What was it that worried you the most just now?” “I let her speak first, and then I share my side. I’ve realized that if I try to explain myself right away while she’s still angry, it doesn’t work well. So, I listen to her first before speaking about myself. That’s something I learned along the way.

Researcher: Have these experiences changed your understanding of marriage?

Interviewee: Yes. I used to think marriage meant two people becoming more and more alike. Now, I don’t think so. Marriage is actually about two people coming to understand why the other person is different from them. Especially in cross-cultural marriages, it’s impossible to completely eliminate cultural differences. Sometimes, the most important thing isn’t erasing those differences, but knowing when to explain things and when to give your partner space.

Researcher: If you were to redefine ‘silence’ now, how would you define it?

Interviewee: I don’t think silence necessarily means refusing to communicate. It could be a way to avoid conflict, process emotions, or protect the relationship. However, silence on its own can’t solve every problem; it only creates a space. What really matters is whether you come back after the silence and actually say what needs to be said. If you stay silent indefinitely, the problem remains unresolved.

Researcher: Regarding your silence within a cross-cultural family, is there anything else, something we haven’t asked about but you feel is important—that you’d like to add?

Interviewee: I don’t think silence is something to be afraid of; what matters is explaining the reason for the silence afterward so the other person doesn’t have to guess. In the past, when I went silent without explaining, my wife would always assume I was angry. Sometimes, she would even let her imagination run wild. Now, whenever I go silent, I tell her whether I’m tired, deep in thought, or just dealing with some emotions. Once she knows the reason, she stops guessing. Another crucial point in cross-cultural marriages is understanding that a partner might not grasp the nature of “Chinese-style silence” due to cultural differences. I used to think, “She should know I’m not angry,” but I later realized she has no obligation to instinctively understand that.

That’s why it’s important to explain things proactively. For instance, I’ve told her that in Chinese culture, silence is sometimes a way to calm down and avoid conflict. Once she understood that, she began to grasp it better. At the same time, I’m learning her way of doing things, too. Sometimes she needs me to explicitly say “I agree” or “I disagree,” so I try to do that now.

I feel that our current dynamic isn’t about one person changing to become like the other; rather, we’ve developed a new way of communicating. I used to view silence simply as not wanting to speak, but now I see it as a way to communicate more effectively. Sometimes, when emotions are running high, what you need most isn’t to speak immediately, but to pause first. However, you have to come back to the conversation after that pause. This is especially true for couples: if you just pause and never return, that’s avoidance; but if you pause to gather your thoughts before sharing them with your partner, I consider that a mature form of communication.

**I-19: (Female, UK partner, without children, married < 3 years)**

Researcher: Looking back at your time together, what do you think were the most significant impacts of your differing cultural backgrounds, such as values, mindsets, and communication styles, on your intimate relationship?

Interviewee: The impact was mainly evident in a few areas. First, regarding communication styles: he had a very typical, direct approach. He was polite but straightforward when expressing needs, saying things like “I need some time,” “What you said upset me a bit,” or pointing out exactly what was wrong. I, on the other hand, was more reserved; when conflicts arose, I hoped he would sense how I felt rather than having to state it explicitly, so I was less proactive in that regard. There were also differences in our views on relationships. In China, there’s a strong emphasis on responsibility, planning, and loyalty; selfishness carries a stigma and is seen as a negative trait, so one is expected to consider others’ feelings in everything. Abroad, however, the focus is more on individual feelings, asking whether the relationship makes both parties happy, feeling free to voice one’s own needs, and accepting a certain degree of selfishness as normal. The emotional well-being of both partners within the relationship takes center stage.

Researcher: Do you ever go silent during your daily interactions or when conflicts arise with your partner? If so, could you describe one or two instances of silence that left the deepest impression on you (e.g., what happened, the atmosphere, how long the silence lasted)? What was going through your mind when you chose to remain silent? Interviewee: Yes, it happened. The most memorable instance was last winter; we had finished dinner and were sitting at the dining table in our rental apartment, discussing where to settle down and our future plans. We were considering moving to a different city, a topic that involved weighing numerous practical factors—career opportunities, room for personal growth, and future arrangements for our parents’ elderly care. He was using a notebook to run the numbers while he spoke, remaining completely calm and objective throughout. He broke down the cost of living, the financial pressure of a down payment, and the likelihood of career advancement for each city—his logic was as clear and precise as if he were conducting a project evaluation. Yet, I had sat down that day with the mindset that “it doesn’t matter where we go, as long as we’re together.” What I wanted wasn’t a meticulously calculated balance sheet; I wanted an emotionally reassuring statement, a promise that “we are definitely going to walk this path together.” The more rationally he analyzed the pros and cons and calculated the return on investment, the greater the sense of disappointment I felt. Eventually, I developed a strong, unsettling impression that our relationship, and the very idea of us walking through life together, had been plugged into his cost-benefit formula, turning us into a commodity with a price tag that could be weighed for value. A wave of hurt and resentment washed over me, making my nose sting, but I didn’t want to cry in front of him, so I simply let the conversation trail off and fell silent. That silence lasted about three or four hours, stretching from just past eight in the evening until nearly midnight. At first, he looked up a couple of times to ask, “What do you think?” or “Do you think this plan works?” but seeing me only shake my head or offer a vague murmur, he realized something was wrong with my mood and stopped discussing the plans. We sat on opposite sides of the table—he flipping through industry materials while I clutched my water glass, lost in thought. Later, we simply moved to opposite ends of the sofa and scrolled through our phones; the room was dead silent, the air thick with a tense, awkward stiffness.

Researcher: When you chose silence, what was the primary thought driving that decision?

Interviewee: My main thought was simply to end the conversation. I didn’t want to keep talking. If I had continued, I would have just gotten more agitated. It likely would have spiraled from a discussion about the future into a blame game, leading me to blurt out hurtful things like, “You don’t care about my feelings at all.” I didn’t want to lash out in the heat of the moment and say things that would damage the relationship—things I’d regret later. On the other hand, I felt that instead of clashing while emotions were running high, it was better for both of us to cool off. Once we were calmer, we could revisit these practical issues; that way, I could clearly articulate my true needs, and we could discuss solutions more rationally.

Researcher: You mentioned earlier that his rationality made the relationship feel like a “commodity with a price tag.” Could you elaborate on that? Was there a specific remark or action that triggered such a strong reaction in that moment?

Interviewee: It was when he said, “If we move to a ‘new first-tier’ city, even combining our salaries and deducting rent and daily expenses, the cost-benefit ratio of saving up for a down payment in three years isn’t actually very good.” When I heard the phrase “cost-performance ratio,” my heart sank. Of course, I knew he was calculating the cost of living and addressing practical realities, but in that moment, I couldn’t help but get hung up on the wording. Did the very idea of us settling down together in the same city have to be measured by “cost-performance”? It felt as if our relationship and the meaning of our life together were being reduced to numbers to determine if it was “worth it.” That one phrase really struck a nerve; a sense of grievance instantly overwhelmed my rationality, and I just didn’t want to speak anymore.

Researcher: After that silence, which lasted several hours, how did you break the deadlock and start communicating again? Did the way you interacted change when discussing similar practical issues afterward?

Interviewee: It was nearly midnight when he came over, nudged my arm, handed me a cup of hot milk, and said softly, “Stop thinking about it for now; let’s get ready for bed.” I took the opportunity to let the tension go and didn’t bring up the topic again that night. The next morning at breakfast, I finally told him that it wasn’t that I disagreed with his plan; it was just that he was so calm and focused on calculating the pros and cons that I didn’t feel like he cared about my feelings. He was taken aback for a moment; he said he thought that by calculating all the risks and arrangements, and presenting me with a solid, secure plan. Later on, whenever we discussed such practical matters, he would start by saying, “I definitely want to be with you; we’re just choosing the best plan.” That reassurance put my mind at ease before we got into the specific costs and logistics. For my part, I tried to listen patiently to his analysis instead of letting my emotions flare up the moment I heard rational calculations. That period of silence actually wasn’t a bad thing; it helped us realize that we cared about different aspects of the situation.

Researcher: In what specific situations are you more inclined to remain silent (e.g., facing accusations from your partner, discussing sensitive topics, cultural clashes, feeling exhausted, or expressing emotions like grievance, helplessness, sadness, or anger)?

Interviewee: I tend to go silent when I feel aggrieved or down; I simply don’t have the energy to argue or explain myself. For instance, when my partner’s words or actions hurt my feelings, but he thinks I’m making a big deal out of nothing. I also go silent when discussing sensitive topics regarding our future or his past relationships. And when I’m exhausted—feeling drained and unable to think clearly, I tend to go silent. Since I’m not Chinese, communication often requires explanation; when I’m tired, I don’t have the energy to explain, so I stay silent while searching for the right words. If the other person doesn’t understand, especially if it’s due to a clash in cultural values where explaining would be too taxing, I’ll just remain silent.

Researcher: What is the main reason you choose silence (e.g., avoiding arguments, feeling it’s impossible to explain, maintaining harmony, protecting your own or the other person’s “face,” cultural influences, language barriers, or simply not knowing how to respond)?

Interviewee: It’s mainly to maintain harmony in the relationship. In certain situations, if an argument continues while emotions are running high, it can easily escalate into a heated quarrel without actually solving the problem. So, I choose silence to avoid saying something rash during an argument that might damage the relationship. Secondly, sometimes I simply don’t know how to respond; I need to pause and use silence as a buffer to gather my thoughts before explaining the situation. There’s also the issue of “face”. I don’t want to show an overly emotional or negative side of myself to the other person; I want to preserve the image I have in their eyes. Finally, I dislike arguing; I find it exhausting and emotionally draining for both parties, and since it rarely solves the actual problem, I feel it’s pointless.

Researcher: What impact does your silence have on your communication or relationship (e.g., maintaining harmony, easing conflict, escalating tension, confusing or hurting your partner, allowing time to cool off, leaving unresolved emotional issues, etc.)? Could you give an example?

Interviewee: I think silence has a few different effects. For instance, it allows both parties to cool down and acts as a buffer, making it easier to resolve the issue when we revisit it later. However, in negative situations, it can also exacerbate the conflict. While I might view silence as a way to de-escalate and calm things down so we can solve the problem later, my partner might perceive it as a form of “cold violence.” Interpreting it as a sign of resentment, neglect, a refusal to communicate, or a negative coping mechanism. Consequently, this can actually prolong the tense atmosphere; if the other person feels the issue remains unresolved or has been ignored, it can lead to lingering resentment. Take, for instance, the earlier discussion about where to settle and build a career—a decision involving many factors. If I choose to remain silent at such a moment, the other person becomes confused.

Researcher: Might your partner misunderstand your silence? How do they typically react to it (e.g., by asking questions, responding with silence, showing impatience, or trying to draw you out)? What is your perspective on these reactions?

Interviewee: There are times when misunderstandings arise, possibly due to cultural influences. From his perspective, silence during a disagreement is tantamount to the silent treatment—a form of punishment directed at the other person. As for his reactions, he tends to take the initiative to ask questions, trying to encourage me to share my views, opinions, and feelings. He is quite patient, asking things like, “Can you tell me what’s on your mind?” or “How are you feeling deep down?” I view this reaction as a sign that he truly values the relationship and is committed to resolving issues through active communication; therefore, I try to understand and appreciate his communication style.

Researcher: From a cultural perspective, which concepts within Chinese culture (such as “harmony in the family leads to success in all endeavors,” “think twice before acting,” “silence is golden,” “careless talk leads to trouble,” or “be cautious in speech and action”), or perhaps your upbringing. Influence your choice to remain silent in an intercultural intimate relationship?

Interviewee: First, there is the concept that “harmony in the family leads to success in all endeavors.” Relational harmony is often closely linked to individual emotions; silence can be a way to temporarily avoid conflict and preserve that harmony, so this concept certainly influences my tendency toward silence. Then there is “think twice before acting.” Many relationship breakdowns stem from impulsive words or actions, so I often weigh whether something should be voiced and reflect carefully before speaking; this cautious attitude is actually beneficial for maintaining an intimate relationship. Of course, the influence of one’s family of origin plays a role as well. For instance, when parents are in conflict, if they keep trading barbs and arguing back and forth, the situation often escalates; both parties become emotionally charged, the actual problem remains unresolved, and the relationship suffers. However, if they take some time to cool off, spending a moment apart in quiet reflection before resuming the conversation actually facilitates a better resolution. Therefore, I believe that silence serves as a highly effective buffer in intimate relationships, especially during times of conflict.

Researcher: Do you feel that the frequency or manner in which you use silence in your cross-cultural marriage has changed? How does the present compare to the early days?

Interviewee: There has been a significant shift. In the beginning, I often used silence passively; I would retreat into my own emotions and wait for my partner to soothe me. Now, however, as our relationship has deepened, I communicate openly. For example, I might say, “I need a few minutes to calm down and gather my thoughts; can we pause this and talk later?” By directly expressing my feelings and giving a clear explanation, I help him understand that my silence isn’t the silent treatment or a hostile standoff, but rather my way of processing emotions within the relationship. Also, we agreed to break the ice actively if silence lasts over ten minutes after quarrels, so as to eliminate misunderstandings and prolonged cold wars. This approach reduces any resistance or negative reaction he might have toward the silence.

Researcher: Comparing your communication style with that of your Chinese friends of the same gender who are not in cross-cultural relationships, do you think your silence within your cross-cultural relationship differs from the silence they experience in their intimate relationships? What are the main differences?

Interviewee: I think there are some differences. For instance, with Chinese friends in same-culture relationships, it’s easier for their partners to understand the meaning behind their silence because they share the same cultural framework. A close friend’s boyfriend, for example, can more accurately grasp what her silence signifies—whether it stems from feeling aggrieved, angry, or exhausted, or if it’s a way of acting coy or playful. However, in a cross-cultural relationship, interpreting that silence can be more of a struggle. The partner might tend to view it negatively, assuming I’ve given up on communicating, am giving them the “silent treatment,” or don’t value the relationship. Consequently, silence carries certain risks and costs; I have to patiently explain the underlying reasons for my silence—clarifying whether I’m feeling aggrieved, exhausted, or sad.

Researcher: Is there anything else regarding your silence in this cross-cultural relationship, something we haven’t asked about yet—that you feel is important and would like to add?

Interviewee: I used to rely heavily on silence as a default response, especially when the relationship was just forming. Now, however, I make an effort to explain my internal thoughts and feelings during those silent moments, leading to a gradual, reciprocal influence. We are influencing each other; he teaches me to be more direct about valuing my own feelings within the relationship, while I can articulate the emotions hidden behind my silence—whether it’s a sense of grievance, hurt, or sadness. Through building this intimate relationship, we both grow: I become better at expressing my emotions verbally, and he learns to give me the space I need, allowing us to find a balance.

**I-20: (Female, Australian partner, with children, married < 3 years)**

Researcher: To start, I’d like to ask for a general overview. Looking back at your journey from dating to marriage and having children, what do you think were the most significant impacts of your differing cultural backgrounds, such as values, mindsets, and communication styles, on your relationship?

Interviewee: Well, there were quite a few impacts. After all, we grew up in completely different environments, so we often approached things differently. The most obvious difference was definitely in communication styles. He has a typical Western mindset. He speaks his mind and is very straightforward; you can see exactly how he feels on his face, and he says it like it is. Even when we argued, he’d get it out of his system right then and there and move on. He wouldn’t hold a grudge. I’m the complete opposite; I tend to bottle things up at first and sort out my thoughts before speaking. Often, if I felt something wasn’t worth saying or might hurt feelings, I’d just stay silent.

Researcher: Could you give a specific example of a small, everyday occurrence—a common situation in your life that illustrates this difference?

Interviewee: For instance, if I did something wrong, he’d say so directly; if the food didn’t taste right, he’d say that straight out, too. He was incredibly blunt—no sugar-coating at all. At first, that made me really uncomfortable. I’d think, “You don’t even compliment me, and on top of that, you’re nitpicking.” If the roles were reversed, say, if he messed up a meal. I’d never tell him to his face that it tasted bad. I might just eat a little less of it. If I really had to say something, I’d phrase it like, “The flavor is quite unique today.” I’d never point it out directly because I wouldn’t want to hurt his pride or dampen his mood.

Researcher: Beyond communication styles, were there significant differences in your values—regarding things like family boundaries, personal space, or attitudes toward spending?

Interviewee: The difference regarding personal space was huge. He places a lot of importance on personal boundaries. I eventually learned that this is how they’re raised. Even between parents and children, everyone has their own independent space. It’s not that they aren’t close; it’s simply about wanting to relax on their own without being glued together every moment. I’ve gotten used to it now; if he wants some alone time, he takes it, and I can just scroll through my phone or watch a show. It’s actually quite comfortable. Then there’s the matter of spending habits. When we first started dating, he insisted on splitting everything, even the cost of a coffee or a meal. I really wasn’t used to that at the time; I thought it was normal for the guy to pay. Splitting the bill down to the last penny felt so impersonal. It didn’t feel like dating, but more like sharing a table with a stranger. After a long period of adjustment, we settled into a routine: we share household expenses, like the mortgage, utilities, and costs for the kids, but handle personal spending (like him buying sneakers or me buying skincare) independently. Surprisingly, this has actually reduced a lot of conflict.

Researcher: Did these differences cause frequent friction when you first got together?

Interviewee: Oh, definitely. During the first six months, we’d bicker over these little things all the time. I’d get upset without saying why, leaving him completely baffled and thinking I was being irrational. He was too blunt, which made me feel he was inconsiderate and lacked emotional intelligence, like he didn’t know how to be mindful of others’ feelings. One incident that really stands out happened three months into the relationship: for my birthday, he gave me an electric iron. He said it was super practical since I wore shirts to work that needed ironing. My face fell the moment I saw the gift, but I didn’t say anything; I just stayed silent all evening. He thought I didn’t like the color. He completely missed the point that I felt he hadn’t put any real thought into it. It’s actually pretty funny to think about now.

Researcher: Now that you’ve been married for over two years, have the effects of these differences softened, or do they remain a major source of conflict?

Interviewee: They’ve definitely softened. After all, we’ve been together nearly four years, so we’ve gradually gotten to know each other’s temperaments. It’s not that the differences have disappeared, but rather that we’ve learned how to live with them. For instance, he’s learned to be a bit more tactful when he speaks, and I try to voice my thoughts instead of bottling everything up.

Researcher: Right. Let’s move on to talk about specific instances of silence. Do you ever go silent during your daily interactions or when a conflict arises? If so, could you describe one or two specific scenarios that left the strongest impression on you? For example, what happened, what the atmosphere was like, how long you stayed silent, and what was going through your mind when you chose silence?

Interviewee: Oh, absolutely, and it happens quite often, too. Especially after having the baby, there were many times when I really didn’t want to say a word. My child had just turned six months old; I had done my homework by reading numerous parenting books, attending a hospital class on introducing solids, and consulting experienced friends. Everyone agreed that the first solid food should be iron-fortified rice cereal, followed by a gradual introduction of vegetable and fruit purees, and—crucially—absolutely no salt or sugar before the age of one. He, on the other hand, asked his friends in Australia and his mother. They told him that children over there start solids as early as four months and even eat meat purees and egg yolks; they said there was no need to be so meticulous—that raising a child too delicately actually results in a weaker immune system. One night, we were sitting at the dining table, staring at the baby’s bowl of food, and an argument broke out. He accused me of being overly anxious and coddling the child, claiming that he ate whatever was available growing up and still grew to be over 1.8 meters tall. I was instantly furious. I spent my nights staying up late researching and taking notes, and my days looking after the baby until my back ached—wasn’t I doing it all for the child’s well-being? How could he dismiss all my effort with just one sentence?

Researcher: So, you chose silence? How long did that silence last?

Interviewee: Right. After he said that, I wanted to argue back, but the words died on my lips; I was suddenly speechless. I just sat there staring at the baby’s little silicone bowl—not looking at him, not saying a word. We sat there in a tense standoff for about twenty minutes. Eventually, I got up and went to the kitchen to wash the dishes, without saying a single word to him the whole time.

Researcher: What exactly was going through your mind during those twenty minutes? Were you seething with anger, thinking of how to refute him, or did you simply not want to argue anymore?

Interviewee: For the first few minutes, I was definitely angry and felt terribly aggrieved; tears were welling up in my eyes. But the more I thought about it, the more I realized it was pointless to try to explain things to him. He was set in the mindset he’d grown up with; no matter how much scientific evidence I presented, he would just think I was being uptight or overly anxious. Besides, an argument would inevitably get louder, and the baby was sleeping in the bedroom. If the noise startled the baby awake or made him cry, it just wouldn’t be worth it. Eventually, I just decided not to say anything at all. I felt that talking was pointless; it was better to calm down and wait until my anger subsided. Besides, there was no way to determine who was right or wrong in the argument, so why bother?

Researcher: What was the atmosphere like at home at that time? How did he react when he saw you go silent?

Interviewee: The atmosphere was incredibly stifling. At first, he kept rambling on about his point of view, but when he saw I had suddenly stopped speaking, he fell silent too. He just sat on the sofa playing on his phone, stealing glances at me every now and then. I could sense he was a bit flustered and at a loss for what to do, but he was too proud to be the first to speak to me. Then there was the last Spring Festival—the second one after we got married. We had originally agreed that since we’d spent the previous Christmas and New Year in Australia, we would spend the next Spring Festival in my hometown. I had already made arrangements with my parents and grandparents; the older folks were really looking forward to us bringing the child back for a visit. But just a week before the holiday, he suddenly told me his parents had booked flights to China to spend the festival with us. He suggested my parents come over too, so we could all celebrate together at our place. My heart sank instantly, but I didn’t argue with him or say I was upset. From that evening on, I hardly initiated any conversation with him. I stayed silent for nearly a whole day; from that night until the following afternoon, I barely said a few complete sentences.

Researcher: When you chose silence on that occasion, what was the main thought going through your mind?

Interviewee: First, I felt disappointed; I thought he had gone back on his word and didn’t care about my family at all. But then I thought, his parents were making a long, difficult journey from Australia. If I started a fight over this, I’d look immature and petty, and his parents might form a bad impression of me. It was the Spring Festival, after all, fighting would just make everyone miserable, so what was the point? Plus, I felt that bringing it up would make me seem stingy or unfilial toward his parents. I also harbored a bit of wishful thinking, hoping he’d notice I was unhappy and figure it out on his own. But he didn’t pick up on it at all; the next day, he even asked if I had a cold because I seemed so listless. Looking back on it now, it’s actually both infuriating and funny. Researcher: So, regarding those two instances of silence—one was to avoid conflict because you felt arguing wouldn’t lead to a resolution, and the other was to keep the peace and avoid the embarrassment of speaking up?

Interviewee: Right, that’s pretty much the mindset. Often, silence doesn’t mean I have nothing to say; it’s just that I feel speaking up would do more harm than good.

Researcher: Looking at your daily life, in what specific situations are you more inclined to stay silent? For instance, when facing accusations from your partner, discussing sensitive topics, dealing with clashes in cultural values, feeling exhausted, or experiencing emotions like feeling wronged, sad, or angry?

Interviewee: Actually, it happens in quite a few situations. First, when an argument gets really heated, I’ll definitely stop and go silent; I don’t want to keep fighting and risk making things worse.

Researcher: Aside from the peak of an argument, do you also stay silent when facing his accusations or criticism?

Interviewee: Yes, absolutely. For example, there are times when I’ve spent the whole day looking after the kids and am dead tired, and the house is a bit messy because I haven’t had time to tidy up. If he comes home from work and casually remarks, “Why is the place such a mess?”, I instantly lose the desire to speak. I feel deeply aggrieved. Like, looking after the kids is exhausting enough; not only do you fail to acknowledge my effort, but you also nitpick. But I won’t argue with him; I just stay silent and go about my business.

Researcher: What about when there are clashes in cultural values? For instance, when discussing matters involving both families or social obligations. Do you choose silence then, too?

Interviewee: All the time. Take gift-giving during holidays, giving red envelopes to kids, or contributing money for colleagues’ and friends’ weddings. He just doesn’t get it at all. He says we’re all adults, so why exchange money and gifts? He thinks it’s just empty formality. I usually don’t argue with him in those moments; there’s no point. He didn’t grow up with these customs, so no matter how much I explain, he just sees it as a waste of money. I just stay silent and listen to him, then do what I was going to do anyway. I can’t be bothered to explain.

Researcher: When you’re feeling exhausted, say, from childcare or work, do you also find yourself not wanting to talk and choosing silence?

Interviewee: Oh, that happens all the time. For instance, if the child fusses all night and I don’t sleep at all, and then the next day he tries to talk to me about work or weekend plans, I just don’t want to engage. It’s not that I’m angry; I’m simply so exhausted that I don’t have the energy to open my mouth, and my brain isn’t functioning well either. In moments like that, I fall silent. At first, he thought I was angry again, but over time he learned that if I’m listless and quiet, it means I’m tired. He’ll let me catch up on sleep while he takes care of the child.

Researcher: So, when you experience negative emotions, like feeling aggrieved, sad, or angry, are you more inclined to express that through silence rather than speaking up directly?

Interviewee: Yes, I think that’s just been my personality since childhood. When I felt wronged at school, I wouldn’t tell my parents; I’d just hide in my room and cry. It was the same in my romantic relationships later on: if I was upset, I’d go quiet and wait for the other person to notice and comfort me. I always felt that if I actually said, “I’m angry” or “I feel wronged,” it would make me seem overly sensitive or like I cared too much—almost like I was “losing” the situation. Especially when I’m angry, I worry that if I open my mouth, I’ll say something harsh or hurtful that can’t be taken back and might damage the relationship. So, I just keep quiet and calm myself down first.

Researcher: Are there any situations where you absolutely wouldn’t stay silent and would speak up directly?

Interviewee: I absolutely won’t stay silent when it comes to the child’s safety. For example, if he’s holding the child while playing on his phone and his grip slips, or if he feeds the child the wrong thing, I’ll call him out on it right then and there, and quite sternly, too. Then there are matters of principle, like if he lies or fails to follow through on a promise. I’ll definitely confront him about those things; I won’t bottle it up. As for other trivial, day-to-day matters, I can’t be bothered to argue; I usually just turn a blind eye and let it slide.

Researcher: Next, I’d like to ask: what are the main reasons you choose silence? Is it to avoid arguments, a feeling that things can’t be explained clearly, a desire to maintain harmony, a need to save face (for yourself or the other person), cultural influences, the pressure of verbal expression, or simply not knowing how to respond in the moment? Please feel free to share based on your own experience.

Interviewee: Actually, it’s a mix of several reasons; it’s not just one single factor. The main reason is simply that I don’t want to argue; I feel it damages the relationship too much. Besides, a lot of arguments don’t lead to any resolution anyway, so it’s better not to say anything.

Researcher: Why do you feel arguments don’t lead to a resolution? Is it due to cultural differences or a fundamental mismatch in your underlying logic, like you’re just not on the same wavelength?

Interviewee: That’s a big part of it. Many issues don’t have an absolute right or wrong answer; it often comes down to different upbringings and mindsets. You can’t convince him, and he can’t convince you. Take things like exchanging gifts during the Lunar New Year or deciding whether to raise a child with a meticulous or a more laid-back approach. You could argue about these until dawn without settling anything. Talking too much just gets exhausting and hurts the relationship, so it’s better to stay silent and both take a step back.

Researcher: So, does the desire to maintain harmony—that idea of “harmony in the family leads to prosperity in all things”—have a significant influence on you?

Interviewee: It does. It’s deeply ingrained in me. My parents taught me from a young age that when living as a family, you shouldn’t nitpick; taking a step back opens up a world of possibilities. You shouldn’t make a scene over trifles and let the neighbors laugh at you. So, when I encounter a conflict, my first reaction isn’t to rush in and fight to win; instead, I step back and stay silent to keep the situation under control and avoid disrupting the family harmony. I’ve always felt that home is a place for love, not for arguing about who is right. Winning the argument but losing the relationship isn’t worth it.

Researcher: What about protecting each other’s “face”? Do you ever choose silence, giving him a way to save face, even when you’re unhappy, especially when you’re out in public or with his friends?

Interviewee: Absolutely. For instance, there was a time he brought colleagues home for dinner. In front of them, he joked that my cooking was mediocre and claimed I was lazy, saying he was the one who cleaned the house. That wasn’t the case at all; I actually did most of the housework. But I didn’t say a word at the time; I just smiled, poured their drinks, and served the food without showing any sign of displeasure. I only brought it up with him after the friends had left, telling him he shouldn’t have said those things in front of outsiders. Men need to save face when they’re out; if I’d argued with him right then and there, it would have been incredibly embarrassing for him, and awkward for his colleagues, too. Things like that are best discussed in private; there’s no need to air them publicly. Researcher: Is there any pressure regarding language expression? For instance, are there times when you can’t quite articulate your emotions in English, or worry about not expressing yourself well?

Interviewee: Oh, we usually communicate in Chinese; his Chinese is actually quite good. He handles daily conversations and even arguments without any issues. It’s just that he might not fully convey particularly complex or nuanced emotions. But my silence really isn’t due to language barriers; I’d act the same way even if I were dating a Chinese man. It’s just my personality and habit. It’s just that in an intercultural marriage, silence is more easily misunderstood.

Researcher: Are there many situations where you don’t know how to respond right away? Like when he suddenly asks a major question and you can’t make up your mind immediately, so you stay silent to think it over?

Interviewee: Yes. For example, sometimes he’ll suddenly want to have a serious discussion about whether we should immigrate to Australia in the future, or whether we should have a second child. With big issues like that, I can’t make a decision on the spot—my mind gets a bit jumbled, so I stay silent first to weigh the pros and cons carefully before answering him. It’s not that I don’t want to speak; I just need time to organize my thoughts. I can’t just blurt something out carelessly.

Researcher: Is there also an element of self-protection? Like, are you afraid that if you voice your emotions, he might dismiss them or think you’re being overly sensitive, so you simply choose not to say anything?

Interviewee: Hmm. There is a bit of that, especially when we first started dating. Back then, if I told him I felt aggrieved, he’d say things like, “What is there to be upset about?” or “You’re overthinking it.” I felt like my emotions weren’t being accepted or understood. Voicing it actually makes me feel worse, so I’d rather just bottle it up. Like you said, it’s a form of self-protection; if I don’t say anything, I won’t face rejection, and I won’t get hurt any further.

Researcher: So, what kind of practical impact does your silence have on your communication or relationship? Does it help maintain harmony or defuse conflict? Or does it escalate tensions, leave your partner confused or hurt, or create lingering resentment? Could you give some specific examples?

Interviewee: It has both positive and negative effects, depending on the stage of the situation. On the positive side, it gives everyone a chance to cool down and eases the conflict.

Researcher: Could you give a specific example of how silence actually helped defuse a conflict?

Interviewee: Well, one time we were arguing about whether to enroll our child in an early education class. He thought those classes were a waste of money—a “scam” targeting parents’ anxiety, and believed we could just teach the child ourselves at home. I, on the other hand, felt it was good for the child to interact with peers and develop social skills. The argument got heated; his voice rose, and his face turned red. Seeing the situation spiraling, and fearing that the shouting would scare the child or lead us to say hurtful things we couldn’t take back, I simply stopped talking and went to the bedroom to play with the child. About forty minutes later, he gently opened the door, came in, and apologized. He admitted he shouldn’t have raised his voice or dismissed my ideas. We sat down and talked again—our tone was much calmer this time, and eventually, we both compromised by agreeing to sign up for a short-term course first to see how it went. If I had kept arguing right then, things would have definitely escalated, and we might have even started bringing up old grievances, which wouldn’t have solved the problem at all.

Researcher: What about the negative effects? Have there been times when silence deepened misunderstandings or escalated the conflict?

Interviewee: Oh, plenty of times, especially when we first got together. He couldn’t understand at all why I’d suddenly go silent; he’d assume I was giving him the silent treatment or that I didn’t love him anymore. There was one time he forgot my birthday, so I stayed silent and barely acknowledged him all day. That evening, he actually asked me, with teary eyes, if I wanted to break up with him.

Researcher: Have there been instances where prolonged silence left lingering resentment—where you didn’t speak up at the time but kept it in your mind afterward?

Interviewee: It happened once, back when we were newlyweds. Once, he went out drinking with friends and didn’t get back until past 2 a.m., reeking of alcohol. I was furious and deeply worried, but instead of arguing, I just gave him the cold shoulder for three days—hardly speaking a word to him. He thought I was just throwing a tantrum and would get over it in a couple of days, so he didn’t take it seriously or offer a proper apology. But I kept it bottled up; over two months later, during another argument, I brought it up. He was shocked. He had no idea I’d been that angry; he’d thought I was just a little displeased. He told me that if I didn’t speak up at the time, he’d never know what actually bothered me. That experience made me realize you really can’t keep things bottled up. You assume the other person understands, but often they don’t “get it” at all. Over time, it turns into a lingering grievance that actually damages the relationship more.

Researcher: So, looking at the big picture—up to this point—has silence had a more positive or negative impact on your relationship?

Interviewee: It’s definitely more positive now. In the beginning, it was mostly negative because he didn’t understand and I wouldn’t speak up, so misunderstandings just kept piling up. Now, we know each other’s temperaments inside out and have a real rapport; when there’s silence, we generally know what state the other person is in, so we don’t let our imaginations run wild. Plus, I don’t stay silent for too long anymore—usually, I just need a few dozen minutes to cool off, rather than the days-long silent treatment I used to give.

Researcher: I’d like to ask next: does your partner ever misunderstand your silence? How does he usually react to it? Does he ask what’s wrong, go silent himself, get impatient, or try to draw you out? What do you think of those reactions?

Interviewee: Oh, there were definitely misunderstandings, almost daily at the start. Initially, he couldn’t grasp that kind of Eastern reticence at all; he always felt that silence meant a refusal to communicate, or even a form of emotional abuse, like the “silent treatment.”

Researcher: When he misunderstood you early on, was there anything he said that really stuck with you?

Interviewee: Yes, he said, “Every time you suddenly go silent, I feel completely helpless.” That really hurt to hear. I wasn’t trying to emotionally shut him out; it was just my habitual way of processing emotions, and I assumed he’d understand.

Researcher: So, how did he usually react when he saw you go silent back then? Interviewee: At first, he was just anxious—really anxious. He’d keep pressing me: “What’s wrong? Say something. Are you mad? What do you actually want?” He’d ask so urgently, firing off question after question. The more he asked, the less I wanted to speak; I found it annoying and felt like he didn’t understand me at all. Then, after asking so much, he’d get impatient and angry himself, and eventually stop talking too. We’d both fall silent, giving each other the silent treatment, and creating a vicious cycle.

Researcher: What about now? Has his reaction to your silence changed?

Interviewee: It’s changed a lot. We actually had several specific conversations about silence later on, and after one major argument, we really laid everything out in the open. We’ve made a mutual pact: brief silence simply means I’m processing my thoughts, whereas prolonged silence paired with a dark expression indicates bad moods. Now, he rarely misinterprets my reflective silence as indifference or emotional evasion.

Researcher: After reaching that understanding, how did his actual reactions change?

Interviewee: Now, when he sees me suddenly go quiet, he doesn’t immediately lean in and press for answers. For instance, if we’re discussing something and I pause to think, staying silent, he’ll just go get a drink of water or play with the kids, waiting for me to speak when I’m ready. If I’ve been silent for nearly an hour and look upset, he’ll come over, give me a hug, and ask softly, “Are you unhappy? Do you want to talk about it?”

Researcher: How do you feel about these changes in him?

Interviewee: I’m really touched. After all, he grew up in a Western culture and originally didn’t understand this kind of indirect emotional expression at all. The fact that he’s willing to learn, adapt, and even remember the different meanings behind my silence. I think that’s really impressive. Of course, I’m changing too; I try to be direct and not make him guess. It’s a two-way street; you can’t expect just one person to make all the compromises.

Researcher: Does he ever respond to you with silence in return?

Interviewee: Very rarely; he’s the type of person who can’t keep things bottled up. When he’s truly angry, he’ll say straight out, “I’m in a bad mood right now and need to cool off,” then go to the study for a bit. He’ll come out ten minutes later and be fine. We can communicate normally again. He doesn’t go silent for half the day like I do.

Researcher: Moving on from reactions, after a period of silence, how do you usually “break the ice” and get back to normal communication?

Interviewee: It depends on the stage of the relationship; it’s completely different now compared to when we first started dating.

Researcher: How did you usually break the ice back then?

Interviewee: When we first started dating, he was usually the one to make the first move to soothe things over. He’d come over, talk to me, and try to cheer me up, or just say, “I’m sorry, I was wrong,” even if he didn’t actually know what he’d done wrong; he’d apologize first anyway. He said the mindset where he’s from is that you shouldn’t give each other the silent treatment after an argument—you have to resolve it quickly, and there’s no shame in a guy being the first to back down.

Researcher: How would he usually try to soothe you? Sweet talk, or actions?

Interviewee: He’s not really one for sweet talk; he’s very straightforward. For instance, he might come over and give me a hug, or bring me something to eat, and then say, “Please don’t be mad anymore, okay?” I don’t actually want to stay angry forever; I’m just waiting for a way to back down gracefully. Once he coaxes me a bit, I’m happy to let it go.

Researcher: Has the way you break the ice changed after marriage, especially after having children?

Interviewee: It’s changed a lot; nowadays, there’s hardly any need for deliberate coaxing. Often, after a period of silence, the baby might cry or get hungry, and we’ll both lean in to check on the child. We start chatting about the baby, one thing leads to another, and we naturally make up without needing a formal apology or ice-breaking moment. We’re an old married couple now; the issues are usually trivial, so there’s no point in staying at odds.

Researcher: Are there times when you take the initiative to break the ice?

Interviewee: Yes, there are, like when I’m clearly in the wrong. For example, I once wrongly accused him of going out for drinks after getting off work without telling me. In reality, he had told me earlier that day, but I was so frazzled from looking after the baby that I’d forgotten. I stayed silent all afternoon, but that evening, I made his favorite dish, brought it to him, and quietly said, “I’m sorry about earlier; I remembered it wrong.” He smiled immediately and said it was fine; he even comforted me, saying it was perfectly normal to get things mixed up when I was exhausted from childcare. Actually, taking the initiative isn’t that hard. It’s just that, at first, you might feel too proud or embarrassed to do it, but you get used to it.

Researcher: Have you ever had a “cold war” situation where the silence lasted a really long time, like a day or two without speaking?

Interviewee: No. We made a pact: never let an argument drag on overnight. We resolve the day’s conflicts that same day. We absolutely refuse to go to sleep angry. So now, the silence lasts a few hours at most; we always clear the air before bed.

Researcher: Do you ever explain to your partner why you went silent in the first place? Does that kind of retrospective explanation help reduce misunderstandings?

Interviewee: In the beginning, absolutely not. Back then, I really believed in the idea that “those who understand you don’t need an explanation, and for those who don’t, why bother?” I felt that explaining it ruined the moment, thinking that if he loved me, he should understand why I was upset. Later, I realized that approach didn’t work at all. He genuinely didn’t understand; there’s a cultural gap there, and if you don’t say it out loud, he’ll never guess it.

So, I eventually learned to take the initiative and explain things.

Researcher: When do you usually choose to explain? Does this happen the same evening, or a day or two later?

Interviewee: It’s almost always the same day. After the kids are asleep and we’re lying in bed, we have a quiet chat. For instance, if we’d argued during the day and I’d gone silent, I might say to him later, “When you said the house was messy today, it really hurt my feelings. I’d been looking after the kids all day and simply didn’t have the energy to tidy up, which is why I didn’t want to talk.”

Researcher: Do you think explaining things after the fact helps much in clearing up his misunderstandings?

Interviewee: It helps a huge amount, really. Once you talk things through, a lot of issues turn out to be no big deal. For example, he used to think my silence was a form of the silent treatment or a way of punishing him. Later, I explained that I went silent because I didn’t want to fight. I wanted to calm down first because I was afraid I’d say something harsh and hurt our relationship. He understood immediately. I also go silent when I’m thinking things through; he used to assume I was ignoring him or didn’t want to talk, but now that he knows I’m just organizing my thoughts, he doesn’t overthink it. In fact, he gives me space.

Researcher: How does he react when you explain things? Does he listen carefully, or does he push back?

Interviewee: He listens very carefully and tells me what he was thinking at the time. There was one instance where I went silent because he said my cooking wasn’t good. Later, I told him that after working hard to make a meal, having it criticized directly hurt my feelings. It made me feel like my effort was being dismissed. He explained that he was just casually voicing his honest opinion; where he’s from, it’s normal to express preferences directly. It wasn’t about being picky or dismissing my effort. He changed his approach after that; now, if he wants to say something, he’ll start with a compliment before offering a small suggestion, like, “This dish is delicious today; it would be perfect if it were just a little less salty.”

Researcher: Are there times when you explain things but he still doesn’t understand?

Interviewee: Yes, there are. Take social obligations or gift-giving during the Lunar New Year, for example. I’ve spent ages explaining that these are the customs where I’m from—a way to maintain ties with relatives, but he still feels it’s unnecessary, just empty formality. Still, at least he understands why I go silent over these things; he doesn’t think I’m getting angry for no reason. Understanding is one thing, but respect is another. Even if he doesn’t agree with my perspective, he respects the way I handle things and doesn’t try to force me to change; for me, that’s enough.

Researcher: Let’s talk about this from a cultural perspective. What concepts within Chinese culture, or aspects of your upbringing, do you think influence your choice to remain silent in cross-cultural romantic relationships?

Interviewee: I think the primary factor is reticence. We Chinese tend to be reserved when expressing feelings or emotions; we aren’t as direct or passionate as Westerners. Whether it’s love or unhappiness, we don’t talk about it constantly; we prefer to show it through actions or let the other person sense it for themselves.

Researcher: Could you elaborate on that based on your upbringing? For instance, was that the atmosphere in your family when you were growing up?

Interviewee: Yes, my parents were typical Chinese parents. They spent their whole lives together without ever saying “I love you,” yet they took care of each other and always kept the other’s needs in mind, like bringing tea or water when one was sick, which spoke volumes more than words ever could. If I cried because I felt wronged as a child, my mom wouldn’t come over to hug or comfort me; instead, she’d say, “Stop crying. What is there to cry about over such a small thing?” or “You’re a girl; don’t cry at the drop of a hat. People will laugh at you.” So, I learned early on to keep my emotions hidden rather than showing them openly; otherwise, I’d be seen as immature or overly dramatic. I felt I had to process negative emotions on my own rather than dumping them on others.

Researcher: Aside from that sense of reticence, does the concept of “valuing harmony above all else” play a role as well?

Interviewee: Definitely. “Harmony in the family leads to prosperity in all things” and “A moment of forbearance brings calm; a step back opens up a vast horizon.” I heard sayings like these all the time growing up. My dad always said that when a family lives together, you shouldn’t nitpick; winning an argument but losing the relationship isn’t worth it. So, when I encounter conflict, my first reaction isn’t to rush in and argue, but to step back, using silence to avoid confrontation and preserve family harmony. There’s also the idea that “family scandals shouldn’t be aired in public.” Even if a couple has a conflict, it should be resolved behind closed doors; you shouldn’t let everyone know about it or give neighbors and relatives something to gossip about. That’s why, often, I’d rather stay silent and bottle things up than get into a loud argument where others might hear. Researcher: What about the concept of “face”? Did it influence your tendency to stay silent?

Interviewee: It did—whether it was my own “face” or the other person’s. For instance, if I made a mistake, I’d feel too embarrassed to admit it, so I’d use silence to cover it up; I was afraid that speaking up would make me lose face. If he made a mistake, especially in front of others, I felt I had to save his face. I couldn’t point it out right then and there, so I’d just stay silent. Also, I always felt that proactively saying “I’m angry” or “I feel wronged” was a sign of weakness, like begging for attention, and that it was really undignified. It felt like whoever spoke first was the one who “lost.” Looking back, it seems pretty childish, but that’s exactly how I thought at the time.

Researcher: Were there any other cultural beliefs at play? Like “speak less, do more” or “the more you say, the more likely you are to slip up”?

Interviewee: Yes. My dad taught me from a young age that people who talk too much lack composure—that saying too much leads to mistakes, so I should speak less and do more. That’s why I’ve always been someone who acts rather than talks. For example, when I wanted to show I cared, I’d cook, do the laundry, and take care of his daily needs, but I wouldn’t constantly go around saying, “Look how good I am to you.” It was the same when I was upset; I’d just process it silently rather than complaining to everyone.

Researcher: So, do you think these deeply ingrained cultural beliefs are an asset or a liability in a cross-cultural marriage?

Interviewee: It’s a bit of both. On the positive side, I’m quite tolerant and easygoing; I don’t dwell on little things, which keeps the home atmosphere relatively peaceful. The downside is that I’m too reserved—my partner often doesn’t pick up on what I’m feeling, which easily leads to misunderstandings. If I’d married a Chinese man, there might have been a tacit understanding where we knew what the other was thinking without needing words. But in a cross-cultural relationship, that doesn’t work; if you don’t say it, he genuinely won’t know, and he might even assume the worst.

Researcher: Looking back to now, has the frequency or way you use silence in your cross-cultural marriage changed? How does it compare to when you were first together?

Interviewee: During the first six months of dating, I’d get upset over the slightest thing and just bottle it up without saying a word. Nowadays, it might not even happen once every two weeks. Even when it does, the silence lasts only a few tens of minutes—nothing like the old days, when we’d go half a day or even a full day without speaking.

Researcher: What about the way it happens? Has the nature of the silence changed?

Interviewee: It used to be sudden. I’d stop talking without any warning or explanation, leaving him to guess the reason. Now, if I need to cool off or take time to think things through, I’ll tell him something like, “I need a moment to calm down” or “Let me think about it and get back to you,” so he isn’t left confused. It’s shifted from that passive, emotionally charged silence to an active, agreed-upon cooling-off period. The nature of it is different: before, it was a silent treatment born of resentment; now, it’s a pause intended to facilitate better communication.

Researcher: What do you think is the main reason for this change?

Interviewee: First, our bond has deepened, and our trust has grown. I’m no longer afraid that expressing my true feelings will make him laugh at me or stop loving me. Then there’s the fact that we communicate more and understand each other better; we know each other’s boundaries and what makes for the most comfortable dynamic. Also, having a child shifted our focus. We pour our energy into the kid, so we don’t sweat the small stuff anymore. There’s simply no time to throw tantrums.

Researcher: Was there a specific turning point or an event that made you determined to change this habit of silence?

Interviewee: It was that one really heated argument—the time we felt like we couldn’t go on—which was also when we reached that consensus on how to handle silence. After that, I realized that a cross-cultural marriage couldn’t operate on my old, ingrained patterns. I couldn’t keep expecting him to guess what I was thinking; otherwise, the relationship really wouldn’t last. Since then, I’ve forced myself to voice my emotions whenever possible; even if I feel awkward, I try to express them.

Researcher: Do you view this change as a positive thing? Do you feel like you’ve lost anything in the process?

Interviewee: It’s definitely a good thing. We have far fewer conflicts now, and our relationship is much more stable. I used to think true love meant you understood me without me having to say a word. Now I realize that kind of innate rapport is rare, especially between people from different cultures, and that understanding is actually forged through repeated communication. I may have lost that romanticized expectation, but I’ve gained a solid, stable relationship. It’s been well worth it. Researcher: One final question—regarding your tendency toward silence in your cross-cultural family, is there anything we haven’t asked about that you feel is important or would like to add?

Interviewee: Actually, I want to say that silence isn’t a universal trait among Chinese women, nor is it a flaw; it’s simply a different way of expressing emotions and handling conflict. Many people assume that when a Chinese partner stays silent in a cross-cultural marriage, it’s a sign of weakness or avoidance, but that’s not the case. Sometimes, silence doesn’t mean I can’t win an argument or that I’m afraid of my partner; I just don’t want to damage the relationship over something trivial or turn the household into a scene of chaos. Especially after having children, I really want to avoid arguing in front of them; silence is actually a way of protecting the family and the children’s environment. Also, cross-cultural communication shouldn’t require change from only one side. It shouldn’t just be about the Chinese partner learning to be direct; the Western partner needs to learn to understand silence and be accepting of a more reserved style. Relationships are a two-way street, and the process of adjustment goes both ways. It’s about me learning to voice my thoughts while he learns to read my silence; that’s how you find a way of relating that works for both of you. Cultural differences are truly greater than you might imagine. If you don’t speak up, he might never guess what you’re thinking. But you don’t have to force yourself to become exactly like a Westerner, either; the goal is simply to find a rhythm that feels comfortable for both of you.

**I-21: (Female, US partner, with children, married 4–10 years)**

Researcher: Let’s start with a broad overview. Looking back at your time together, what do you think were the most significant impacts of your differing cultural backgrounds, such as values, mindsets, and communication habits, on your intimate relationship?

Interviewee: The most obvious impact was having to consider the underlying reasons behind each other’s actions. Take the boundaries between family and the individual, for instance. I grew up in an environment where family was a tight-knit, interdependent system. Parents’ opinions mattered, and frequent interaction and mutual support among relatives were the norm. He, on the other hand, a typical American, placed a huge emphasis on independence and personal choice. Early in our marriage, my mother would call often to check on us and even offer advice on decisions like when to have children or how to decorate our home. To me, that was just a mother’s love and care. It felt warm. But to him, it felt a bit... intrusive. He would ask in bewilderment, “This is our life; why is your mom making decisions for us?” I had to put a lot of effort into explaining that in Chinese culture, this doesn’t signify interference, but rather deep concern and a sense of “oneness.” Conversely, it took me a long time to adjust to his family’s dynamic—calling only on holidays while everyone lived their own separate lives. I used to wonder if he had a bad relationship with his family or if he was just cold. Then there’s the understanding of conflict versus harmony. In my cultural background, maintaining outward harmony and avoiding direct confrontation is seen as a sign of maturity and social wisdom. Often, you don’t spell things out completely; you leave some room for maneuver. But he’s different; he believes in direct communication—laying issues out on the table—thinking that’s the only way to truly solve problems. That difference is precisely the backdrop for my tendency to go silent. Whenever a situation forced things into the open in a way that made me uncomfortable or even hurt me, I would often choose silence to protect the harmony I was used to, or, perhaps, to protect myself.

Researcher: In what specific situations are you most inclined to remain silent? For example, when facing accusations from your partner, discussing sensitive topics, clashing over cultural values, feeling exhausted, or expressing emotions like grievance, helplessness, sadness, or anger?

Interviewee: Yeah. I might fall silent in almost all the situations you mentioned, though the triggers differ.

The first type is undoubtedly a clash of cultural values—specifically, the kind where both of us feel we are right. Think of issues like parenting, money, socializing, or how we define success and happiness. Once we touch upon these fundamental matters, I tend to retreat into silence. I know it’s not just a disagreement between the two of us; it’s a clash between two different value systems, which makes it incredibly difficult. The second type is when I’m simply exhausted. It’s not just physical fatigue; it’s more of a mental or emotional drain. Maybe I’ve had a long day at work, or the kids have been acting up for hours, and I just don’t have the energy left to organize my thoughts or figure out how to articulate complex emotions in a second language. If a conflict requiring discussion arises at a moment like that, I honestly just want to “power down.” For me, silence is like “power-saving mode.” Then there are those “sensitive” topics, like minor friction involving his family. You know how it is. Some things, once said, can’t be unsaid. I’d rather stay silent than have him think I’m criticizing his family; that would put him in a really awkward position.

Researcher: What is the primary reason you choose silence? For instance: avoiding arguments, feeling it’s impossible to explain clearly, maintaining harmony, saving face (for yourself or the other person), cultural influences, the pressure of expressing yourself in a foreign language, or simply not knowing how to respond in the moment?

Interviewee: The reasons you listed all play a part to some extent. But if I had to pinpoint a core reason, I think the fundamental cause is a combination of the difficulty in explaining things clearly and the influence of cultural values. Deep down, I always feel there is a natural barrier between us. Some feelings, like the sense of insecurity about the future or the complex understanding of family obligations, are intuitions I developed after being immersed in Chinese society for over twenty years. How could I possibly make him fully understand them in just a few sentences, using my second language? Even if I did explain, he might grasp the literal meaning without truly feeling what I feel. He tends to analyze my emotions using his own logic and then offer solutions. But often, I don’t want a solution; I just want to be understood and validated. When I feel that explaining is futile, or that it will only invite more follow-up questions I have to answer, I’d rather not start at all. It’s a sense of powerlessness stemming from the inability to make myself understood. Maintaining harmony in the relationship is, of course, a major factor. As I mentioned earlier, avoiding direct conflict is instinctive for me. Growing up, whenever my parents argued or criticized me harshly, the best strategy was to keep my head down and stay silent until they had vented their anger. I was conditioned to view this as an effective way to let things blow over. I carried this habit into my marriage; when tensions rise, I automatically choose silence to cool things down. I feel that as long as I don’t speak up, the conflict won’t escalate, and we can maintain a semblance of peace. The pressure of verbal expression is very real, too. Especially in the early years of our marriage, my English wasn’t strong enough; in the heat of an argument, I’d get flustered. I might have had a host of logically sound points in my head, but when I tried to speak, only a few dry, awkward words would come out. I felt incredibly foolish, and my confidence would instantly deflate. That sense of frustration made me feel it was better not to say anything at all. So, my silence is a choice driven by multiple factors: I know I can’t explain things clearly; a lack of clarity leads to arguments; arguments disrupt harmony; and on top of that, I may lack the linguistic ability to “win” an argument. Thus, the safest option is simply to keep my mouth shut.

Researcher: What impact does your silence have on your communication or relationship? For instance, does it maintain harmony, defuse conflict, escalate tension, leave your partner feeling confused or hurt, provide time to cool off, or create lingering emotional issues? Could you give an example?

Interviewee: It can do all of those things; the impact is complex. In the short term, it certainly helps defuse conflict and gives us both time to cool down. When it comes to parenting, if I had exploded in the moment, we definitely would have had a huge argument, and the children would have been frightened. My silence stifled the immediate flare-up and gave us space to calm our emotions. I remember one instance where I walked away; he didn’t follow me to argue further, and we each went about our own business, allowing a tense evening to pass without a blow-up. In that sense, silence seemed to maintain a superficial harmony; at least we didn’t lose control in front of the kids. However, staying silent for too long is definitely not good. The issue becomes a “no-go zone” that no one wants to touch, yet the problem still needs to be resolved. He later admitted to me that my silence really confused him and even hurt his feelings a bit. He would rather we talk things out than have me bottle everything up inside; he felt like I didn’t trust him.

Researcher: How does your partner usually react to your silence? Do they ask you what’s wrong, choose to stay silent themselves, show impatience, or try to draw you out? What is your take on these reactions?

Interviewee: They usually try to get me to talk. After I’ve gone silent, he’ll come over a little while later and ask what’s wrong in a very caring tone. His intentions are good. He wants to solve the problem and connect with me. But because I’m often still immersed in the emotion of the moment, I sometimes feel pressured by his attempts to draw me out. Especially when I haven’t sorted out my thoughts yet, his questions can feel like a rapid-fire barrage... It puts a lot of pressure on me; it feels like an interrogation. I might just need a moment to process my emotions, but his persistent questioning makes me feel like I don’t even have the right to be silent. That just makes me withdraw further. Sometimes, my silence is a way of saying “no”—a rejection of what he’s saying or doing. He rarely chooses silence himself because he’s a problem-solver; silence simply isn’t an option for him. He’ll sometimes say in frustration, “I don’t get what you’re thinking. It drives me crazy.” I understand his reactions. I know they come from a place of care and love; we just have different styles of handling conflict. He values transparency, while I prioritize a sense of safety. Now, we’re both learning to understand each other’s needs. He tries to give me a bit more space and time when I go quiet, and I try, when I really can’t find the words, to at least give him a signal, like squeezing his hand or saying, “Give me a few minutes to calm down, and then I’ll talk to you.”

Researcher: After a period of silence, how do you usually “break the ice” and resume communication?

Interviewee: It took us a long time to figure out that “ice-breaking” process. At first, he was always the one trying to break the ice while I responded passively. Now, I’m a bit more proactive. The most common way we break the ice is through non-verbal, everyday actions that don’t carry any pressure. For instance, after a period of silence, I might pour him a cup of coffee or ask if he wants a snack. He’s learned, too. Instead of hounding me with questions while I’m silent, he’ll wait a bit and then break the tension with something completely unrelated, like saying, “Hey, look at this video. It’s hilarious,” or just coming over to pat my head or give me a hug. These actions send a signal: “I’m not angry anymore; we’re safe with each other.” If the other person picks up on that, they usually respond warmly, with a smile or a “thank you.” Then, real communication usually resumes when the atmosphere is more relaxed. He might bring up the topic again, but his tone is much softer than before. He might say, “Can you tell me what you were thinking about what happened yesterday? I just want to understand you.” By that point, having had a moment to cool off, I’ve calmed down and gathered my thoughts, so I can speak more composedly. I tried to explain the situation and let him know that my earlier silence wasn’t because of anything he did wrong, but simply because I needed time for myself. So, our “breaking the ice” doesn’t mean jumping straight back into the issue at hand; instead, we first re-establish a connection through signals of reconciliation, and only then reopen the conversation in a safe atmosphere.

Researcher: From a cultural perspective, what concepts within Chinese culture, or aspects of your upbringing, do you think influence your tendency to choose silence in an intercultural intimate relationship?

Interviewee: That question really gets to the heart of the matter. There are so many factors, honestly. First, there are the concepts that “harmony is precious” and “family scandals shouldn’t be aired in public.” Growing up, I was instilled with the idea that family conflicts should be resolved internally. Ideally, without telling outsiders, and certainly without arguing in front of them. In our marriage, “outsiders” sometimes even includes our children. So, when a conflict arises, my instinct is to suppress it and prevent it from exploding; I feel that kind of scene would be unsightly. Then there are the concepts of “hierarchical order based on age” and “authority.” Although my husband and I are equals in our marriage, the habit of deferring to parents and teachers while growing up left a deep mark on me. When my husband speaks in a very direct manner, almost as if he’s instructing me, that ingrained mindset of “obeying authority and avoiding challenging it” kicks in. I feel that contradicting him would be disrespectful. So, I choose silence, adopting an attitude of acceptance to maintain a veneer of respect. There’s also the issue of how emotions are expressed. I feel that Chinese culture has a certain taboo against the direct, intense expression of negative emotions, like anger or a sense of grievance, especially for women. Expressing such emotions is often viewed as being “immature” or “overly emotional.” We are taught to “endure” and remain “restrained.” Consequently, when I experience strong emotions, I’m used to suppressing and processing them internally rather than expressing them outwardly. My silence is a form of internal processing, not a method of communication. My husband taught me the exact opposite. He believes emotions should be voiced, and that’s something I struggled with for a long time. Regarding the concept of family, I view maintaining family stability and harmony as my responsibility, a duty that even supersedes the expression of my personal emotions. I feel that an emotional outburst on my part could disrupt this stability, so I choose to contain it through silence. I sacrifice my own self-expression for the sake of peace among us. In a cross-cultural marriage, this mindset can easily lead my husband to think I am being dishonest or hiding something, simply because he does not share this logic or way of thinking. Growing up, whenever I voiced a dissenting opinion or expressed strong emotions that my parents deemed unreasonable, their standard response was “Stop talking” or “Children shouldn’t interrupt when adults are speaking.” Over time, I learned that expressing disagreement or intense emotion was unacceptable, while silence was the expected behavior. I carried this pattern into my marriage; whenever my husband and I had a disagreement, that silenced child within me would resurface.

Researcher: Do you feel the frequency or nature of your silence in this cross-cultural marriage has changed? How does the present compare to the early days?

Interviewee: We’ve been married for many years, and I’ve grown as a person. In those early years, my silence was defensive. It happened frequently, lasted a long time, and was absolute. Whenever a conflict arose, I would immediately cut off verbal communication, shut myself in a room or the kitchen, and refuse to talk. I used silence as both a shield and a weapon. Back then, my silence was charged with a sense of grievance, anger, and helplessness. He felt hurt, and I felt incredibly lonely.

Now, my silence is more strategic. It happens less often and doesn’t last nearly as long. I view it as a tool to pause and gather my thoughts, rather than an end to the conversation. The biggest difference is that I now “label” my silence. Before, I would go silent without any warning. Now, if I feel the need for silence, I try to tell him: “I need a moment to figure out how to say this,” or “I’m too emotional right now; can we talk about this later?” This small change has had a revolutionary impact on our communication. It transformed my silence from a signal of refusal to communicate into a signal that I need a moment to compose myself before continuing the conversation. He is much more accepting of this and willing to cooperate. Another difference is that, after the silence, I always take the initiative to explain myself. In the past, after a period of silence, I would pretend nothing had happened and simply gloss over the issue. Now, I realize that sweeping things under the rug doesn’t work; once I’ve calmed down, I take the initiative to approach him and explain why I went silent and what I was thinking. For me, this evolution represents a journey of personal growth and cultural adaptation, as well as the maturing of our relationship.

Researcher: Regarding your tendency toward silence within this cross-cultural family, is there anything else, something we haven’t asked about, that you feel is important and would like to add?

Interviewee: Silence isn’t actually just the absence of sound. In a cross-cultural family, especially after having children, silence can serve as a form of communication in itself. We respect one another and avoid verbal attacks. I might disagree with him, but I can choose to remain silent and avoid an immediate argument; in this way, silence helps maintain a safe, harmonious family atmosphere. Sometimes, my silence is actually an invitation. After he says something, I might fall silent to give him space, allowing him time to reflect on and process his own words. In the context of Chinese-style communication, these are moments of unspoken understanding; it’s a way of offering the other person a graceful way out or gently guiding them toward reflection. Although my husband doesn’t always pick up on this, sometimes assuming I’m angry instead, he is gradually learning to understand this silent language. Furthermore, as a mother, especially when the children were young, I experienced exhaustion and anxiety that were difficult to put into words. The fatigue of late-night feedings or the pressure regarding the children’s future education were things my husband couldn’t fully grasp. Sometimes, I fell silent not because I was dissatisfied with him, but simply because the thoughts weighed too heavily; voicing them would have turned them into mere complaints. My silence holds so much left unsaid regarding the complex roles and emotions I navigate as a mother and wife in a cross-cultural family.

**I-22: (Female, UK partner, with children, married 4–10 years)**

Researcher: Let’s start with a broad overview. Looking back at your time together, what do you think were the most significant impacts of your differing cultural backgrounds, such as values, mindsets, and communication habits, on your intimate relationship?

Interviewee: Hmm, if I had to sum it up, the impact was quite substantial. It wasn’t just an issue early in the marriage that eventually faded away; it’s still there, though we’re better at handling it now. In the beginning, I think the biggest issue was that we had different understandings of what “communication” actually means. I used to feel that if a couple is close, you don’t necessarily have to spell everything out. For instance, if I was upset, I expected him to be able to tell. I might not say directly, “What you did hurt my feelings,” but I thought my facial expressions, tone of voice, or even my silence should make it obvious that I was unhappy. But my husband isn’t like that. He felt that if I didn’t say it, how could he possibly know? He used to tell me all the time, “Just tell me what you need.” At first, I really couldn’t grasp that. I wondered, “Why do I have to say everything out loud? Can’t you just sense it?”

Researcher: So, you feel there was a fundamental difference in how you each understood the concept of “mutual understanding” or “being on the same wavelength”?

Interviewee: Exactly. That was very apparent. In Chinese marriages, we often place a lot of emphasis on that kind of unspoken understanding, especially after living together for a long time. If I was angry and stayed silent, he might ask, “Are you okay?” and I’d say, “I’m fine,” even if what I was really thinking was, “Please, ask me again.”

But he would take “I’m fine” at face value and believe it.

Researcher: So, in those situations, you felt he didn’t really understand what you meant by “I’m fine”?

Interviewee: Right. I later realized that he tends to interpret language quite literally. If you say “I’m fine,” he genuinely believes you are. If you say, “I don’t want to talk about this,” he thinks, “Okay, you don’t want to talk now, so we’ll discuss it later.” But for me, saying “I don’t want to talk” doesn’t necessarily mean “we don’t need to talk.” It might mean “I’m too angry right now and don’t know how to discuss it,” or “I need some space first.” That distinction is actually quite significant. Another factor was how we viewed conflict; in my family, we were always taught to avoid conflict. When my parents argued, they often wouldn’t do it in front of us kids; they might just stop speaking to each other, or one of them would go out for a walk. Growing up, I thought that was just how you handled conflict. But my husband’s family environment was likely different. He believes in speaking up when there’s a problem; he even says that arguing doesn’t mean the relationship is bad. At first, I was actually really afraid of that. I wondered, “Why do you have to be so blunt? Doesn’t saying it out loud just hurt people more?”

Researcher: You mentioned earlier that you’re more accustomed to dealing with uncomfortable emotions by staying silent. So, in your daily interactions with your partner or when a conflict arises, do you tend to go noticeably silent?

Interviewee: Yes. It happened more often in the past. It’s less frequent now, but it still happens.

Researcher: Could you describe one or two specific instances that really stand out in your memory? For example, what happened, what the atmosphere was like, roughly how long you stayed silent, and why you chose not to speak up at the time?

Interviewee: The one that sticks out most in my mind was when our child had just started kindergarten. I was busy with work, the child was still little, and I was basically handling most of the household matters. One evening, my husband mentioned—quite last-minute—that he was going to an event with friends the next day, from the afternoon into the evening. But I had already made plans: I was taking the child to the doctor the next day and had work tasks to handle as well. He said it so casually, like, “Oh, I forgot to tell you. I’m going tomorrow.” I got angry instantly.

Researcher: Did you tell him right then that you were angry?

Interviewee: No. I just said, “Okay,” and then stopped talking.

Researcher: Just a very brief “Okay”?

Interviewee: Right. Then I started getting the child’s things ready. I remember it vividly: a little while later, he asked, “Are you upset?” I said, “No,” and he actually didn’t ask any further. But inside, I was furious. I was thinking, “How could you do this without consulting me? Why do you treat this as just your business? The child is yours too, and the family belongs to both of us.” But I didn’t say any of that at the time.

Researcher: Why didn’t you say anything then?

Interviewee: One reason was that I was just too tired. I felt that if I brought it up right then, it would definitely turn into an argument. Because I already had so much on my mind, I was afraid that if I opened my mouth, I might say some very harsh things. Another reason was that I found the prospect of explaining things too troublesome. I knew he might say, “I didn’t know you had plans,” or “Why didn’t you tell me?” Then I’d have to explain, and that could lead to a conversation like, “So, what exactly do you want me to do in the future?” I didn’t have the energy for that at the time. There was also a deeper reason: I felt like, “Forget it, saying anything probably wouldn’t make a difference anyway.”

Researcher: So, how long did this silence last?

Interviewee: About an evening, I suppose. Strictly speaking, I didn’t stop talking completely, because we still had to take care of the kids. For instance, if they needed a bath or dinner, I’d still speak up. But regarding that issue, I wouldn’t say a word.

Researcher: So, functional communication remained, but emotional communication ceased?

Interviewee: Yes, I think that’s a fair way to put it. At night, he’d say “Good night” and I’d say “Good night,” but that was it. It was the same the next morning. I’d still make breakfast and look after the kids, but deep down, I had shut myself off.

Researcher: During that silence, did you want him to take the initiative and ask you what was wrong, or did you want him to leave you alone?

Interviewee: It was actually a contradiction. On the surface, I wanted him not to ask because I didn’t want to argue; but deep down, I wanted him to notice what was going on.

Researcher: So, you expected him to intuitively understand your emotions?

Interviewee: Yes. Looking back, it was actually a bit unfair, since he didn’t know what I was thinking. But that’s how I felt at the time. I wanted him to say, “I think you’re upset. Did I do something wrong?” If he had said that, I probably would have cried and opened up about it. But if he just asked, “Are you okay?” and I said “Yeah,” and then he just walked away, I’d get even angrier.

Researcher: Why would that make you angrier?

Interviewee: Because I thought, “See? You don’t actually care.” Although, thinking about it now, he probably did care; he just didn’t know what I wanted him to do.

Researcher: Aside from that specific scenario, in what other situations are you likely to go silent?

Interviewee: When I’m really angry. The angrier I get, the less I want to speak. A second scenario is when cultural differences are involved. For instance, issues like child-rearing, spending habits, or the appropriate boundaries between parents and adult children; I tend to be cautious when these matters touch upon both our families. Then there are times when I feel I can’t express myself clearly in English.

Researcher: Do language factors have a significant impact on you?

Interviewee: They used to. My English is fine now, and I use it constantly at work, but it’s different when we’re arguing. Arguments require quick responses. I might have a complex thought in my head, but I can’t immediately find the precise English words to convey it. Take the Chinese concept of weiqu (feeling aggrieved or unfairly treated). It’s not simply “sad” or “angry.” It carries a sense of, “I’ve done so much for this family, yet you haven’t noticed or acknowledged it.” Explaining that nuance would require a long, drawn-out explanation.

Researcher: So, do you sometimes choose silence because the effort required to explain yourself feels too great?

Interviewee: Yes, especially in the past. It’s also the case when his parents are involved; I’m reluctant to be too direct. For example, I might feel uncomfortable when his mother interferes too much in our lives, but I rarely say something like, “Your mother is interfering with our marriage.” I wouldn’t say that. Instead, I might say, “Maybe we need a little more privacy,” or “I think we should have some boundaries.” I’m actually being quite tactful.

Researcher: What do you consider the primary reason for choosing silence?

Interviewee: First, to avoid conflict. Second, I need time to process my emotions. Third, I feel that saying certain things might hurt the other person. And finally, I don’t want to blow things out of proportion.

Researcher: What exactly do you mean by “blowing things out of proportion”?

Interviewee: Well, an issue might originally involve just one specific problem, but if I speak up, it can end up dragging in a lot of past grievances. Take my husband forgetting to pick up the kids, for instance. If I simply said, “I’m angry that you forgot to pick up the kids today,” the matter might end there. But if I were really furious, I might say, “You’re always like this; you never put the family first—you were like this back then, too.” That changes the whole situation. So, sometimes I choose not to speak because I know that once I start, I might lose control. Researcher: So, is your silence sometimes a form of self-control?

Interviewee: Yes. I think it’s more apparent now. In the past, my silence meant “I don’t know how to put it into words,” whereas now, it sometimes means “I know what to say, but I don’t feel it’s the right moment.” I think that’s a significant difference.

Researcher: What kind of impact does your silence generally have on your communication or your relationship?

Interviewee: It cuts both ways. On the positive side, it can indeed prevent an immediate flare-up. For instance, when I’m furious, speaking right away might lead me to say hurtful things. By staying silent for ten minutes, half an hour, or even the whole night until I’ve calmed down, I’m able to speak more rationally. The downside, however, is that if the silence drags on too long, the other person has no idea what’s going on. Sometimes my husband feels I’ve suddenly turned cold, but he doesn’t know why.

Researcher: Have there been instances where the silence actually caused the conflict to escalate?

Interviewee: Yes. There was one time we argued about money. It was actually a trivial matter. He’d bought something expensive without telling me beforehand. I didn’t say anything at the time, but I remained distant for the next two or three days. At first, he’d ask, “What’s wrong?” and I’d reply, “Nothing.” Eventually, he grew impatient. He said, “If something is wrong, please just tell me. I can’t read your mind.” That really upset me. I knew he was right, but the way he said it made me feel like he was blaming me. My thought process was: I’m already hurting so much. Why do I have to be the one to explain everything clearly? So, I just stayed silent. Finally, one night after the kids had gone to sleep, he sat next to me and said, “I know you’re upset. I’m not going anywhere. You can take your time.” I burst into tears right then and there. I opened up about the money issue, and it turned out he hadn’t realized the real reason I was angry. He thought I was mad about the purchase itself, but I explained that what really hurt was the feeling that, even though we’re a family, he still made decisions as if he were an independent individual. So, if he hadn’t been patient and waited for me, I might have stayed silent indefinitely.

Researcher: Do you think your husband understands your silence now?

Interviewee: He failed to understand my silence at first, but over time, he came to realize that it’s just my unique way of communicating. We’ve both learned to put ourselves in each other’s shoes, deeply embracing and respecting each other’s cultural backgrounds and communicative styles.

Researcher: So, when you go silent now, how does he react?

Interviewee: Usually, he gives me a little space first and asks again after a while. Sometimes he’ll say, “Do you want space or do you want me to stay?” I really like that question because it puts the choice in my hands. If I say “space,” he lets me be by myself for a bit. If I say “stay,” he might just sit nearby.

Researcher: What does that mean to you?

Interviewee: It means I don’t have to use silence to control the distance between us. Before, silence was the only way I could tell him, “Don’t come near me,” but now I can just say, “I need an hour.” I think it shows that our relationship has matured.

Researcher: How do you usually resume communicating after a period of silence?

Interviewee: One of us usually speaks up first. Sometimes it’s me, sometimes it’s him. If it’s me, I might say, “Can we talk about what happened yesterday?” I try to avoid starting with accusations now. For instance, I used to say, “You never listen to me,” but now I say, “I felt like my opinion wasn’t considered.” That’s something I’ve learned over time.

Researcher: Was this change something you actively worked on?

Interviewee: Partly. Also, after living together for so many years, I understand how he interprets things. I’ve found that being direct is actually more effective. I used to view directness as a form of conflict, but now I realize that being appropriately direct can actually reduce misunderstandings.

Researcher: Do you explain to your husband why you went silent after the moment has passed?

Interviewee: Yes, I do now. I didn’t used to. I used to think, “If he really understood me, he’d know.” Later, I realized that wasn’t a very realistic expectation.

Researcher: How do you usually explain it now?

Interviewee: I’ll say, “I wasn’t ignoring you. I was trying not to say something I would regret,” or “I needed time because I was overwhelmed.” I think offering an explanation puts him more at ease. He didn’t know what my silence meant before, but now he understands that sometimes my silence isn’t a rejection of him. It’s just me needing to process my emotions.

Researcher: Does that kind of explanation help reduce misunderstandings?

Interviewee: It really does. Especially since we’ve developed a habit of doing it. If I really don’t want to talk, I’ll tell him, “I don’t want to talk right now, but I will talk about it tonight.” That way, he knows I’m not just avoiding the issue.

Researcher: From a cultural perspective, what concepts within Chinese culture, or aspects of your upbringing, do you think influence your choice to remain silent in your cross-cultural marriage?

Interviewee: I think there are quite a few. I was raised to avoid being too direct, especially when speaking to elders. You can’t just come out and say, “You’re wrong.” Even if you think the other person is mistaken, you have to find a different way to express it. Then there’s the concept of “forbearance” or “endurance.” A phrase I heard often growing up was, “Just endure it, and it will pass.” So, when I encounter a conflict, my first reaction often isn’t “I need to resolve this,” but rather “Let it go.” There’s also the desire to avoid making family relationships look bad. My parents’ generation placed a huge premium on “harmony.” If there was an argument at home, people felt it was a bad look. So, I subconsciously came to view spousal arguments as something negative.

Researcher: How does this upbringing manifest most clearly now that you’re in a marriage?

Interviewee: It means I’m very quick to just endure things. For instance, if my husband says something that makes me uncomfortable, my initial reaction isn’t to immediately say, “That made me uncomfortable.” Instead, I think, “Let it go; he probably didn’t mean it that way.” It’s fine once or twice, but over time, the emotions build up.

Researcher: So, how do you see the relationship between “endurance” and “silence”?

Interviewee: I think silence can sometimes be a form of endurance. But now, I feel they aren’t exactly the same thing. In the past, my silence was more about suppressing things and leaving them unaddressed. Now, some of my silence is about pausing temporarily before eventually dealing with the issue. So, I make a distinction now.

Researcher: Could you give an example?

Interviewee: Well, in the past, if my husband said something that upset me, I might stay silent all night and then act as if nothing had happened the next day. Now, I might still hold off on speaking immediately, but I’ll tell him, “Yesterday you said something that bothered me. I wasn’t ready to talk about it.” That’s still silence, but it’s no longer avoidance.

Researcher:

You’ve already mentioned some changes. Comparing the early days of your marriage to now, how has the frequency or nature of your silence in this cross-cultural marriage changed? Interviewee: There’s been a huge change. In the first few years of marriage, I was often silent, and usually passively so. It was because I didn’t know what to say. It was a mix of language and culture. I’d often wonder, “Is what he’s saying a typical American way of expressing things? Am I just being too sensitive?” So, I’d frequently second-guess myself.

Researcher: So, beneath that silence lay an uncertainty about whether your feelings were valid?

Interviewee: Exactly, very much so. For instance, if he told me, “You’re being too emotional,” I might just go silent. I’d wonder if Americans were simply more direct or if I was overreacting. But later, I realized that cultural differences don’t explain everything. Some things are just hurtful. Now, I’m more confident about asserting my boundaries.

Researcher: Do you still go silent these days?

Interviewee: Of course. But the nature of it has changed. Now, I often use silence to pace the conversation, rather than because I’m afraid to speak up.

Researcher: You have children now. Has their presence changed how you use silence in your marriage?

Interviewee: Yes, significantly. When we have a conflict now, we try to avoid arguing in front of the kids. So, sometimes I choose not to say anything right away. I used to feel that staying silent meant bottling things up, but now I realize that with children, context matters. For example, if the kids are around, I won’t argue with my husband about parenting issues. I might just say, “Let’s talk about this later,” and wait until the kids are asleep.

Researcher: You mentioned feelings of grievance, anger, and exhaustion. Which of these emotions makes you most likely to fall silent?

Interviewee: A sense of grievance. Anger is actually easier to deal with because it’s easier to express. Grievance is harder. It’s a complex feeling—you find yourself thinking, “I’ve done so much; why can’t you see that?” “I didn’t use to say things like that. I felt that speaking up about it would look like I was fishing for credit.”

Researcher: Why did you feel that “expressing your own efforts” was like fishing for credit?

Interviewee: It probably has to do with how I was raised. We tend to emphasize that you shouldn’t always talk about what you’ve done. For instance, my mother did a lot of housework, but she never said, ‘Look how much I’ve sacrificed for this family.’ But if she never says anything, others might not realize it. I think that might be an issue now.

Researcher: So, do you feel that silence can sometimes make your own needs invisible?

Interviewee: Yes. I think that’s been one of my biggest realizations over the years. Just because you don’t say anything doesn’t mean others know. Sometimes, they genuinely don’t know.

Researcher: If you were to re-examine your silence now, would you see it not just as a passive reaction, but perhaps as an active strategy?

Interviewee: I think so. Especially now. For example, when my husband is very emotional, I might not argue back immediately. I’ll stay quiet first. It’s not because I’m afraid, but because I feel there’s no point in continuing the conversation right then. I’ll speak up after he’s calmed down. Also, for issues involving both our families, I’m more cautious. I won’t discuss them when emotions are running high. So, I view my silence now more like a ‘pause button.’

Researcher: But is it possible for the other person to interpret that ‘pause button’ as a refusal to engage?

Interviewee: It is. That’s why I try to give a timeframe now. For instance, I’ll say, ‘I need thirty minutes.’ That makes it clearer. If I say nothing and just go silent, he might still feel like I’m refusing to communicate.

Researcher: You’re quite fluent in English now, but do you think language factors still play a role in your silence?

Interviewee: They do. Especially when things get very emotional. Since Chinese is my first language, sometimes the thoughts that pop into my head first are in Chinese. For example, if I want to say, ‘You’ve made me feel deeply disheartened,’ it’s really hard to find an exact English equivalent. I might end up just saying, ‘I’m really...’“ ...disappointed.”

But “feeling disheartened” and “disappointed” aren’t exactly the same thing. So sometimes I feel it’s actually easier to say nothing than to use an inaccurate word.

Researcher: Do you experience a sense of “loss of expression” because of this?

Interviewee: Yes. Especially with very subtle emotions. That’s why we sometimes mix the two languages now. For instance, I might tell him, “I’m not angry; I feel deeply aggrieved,” and then explain further. He’s gradually learned some Chinese emotional terms, too. That’s actually been a big help.

Researcher: You’ve changed over the years; has your husband’s reaction to your silence changed as well?

Interviewee: Yes. At first, he found the silence terrifying. Now he knows I need time. He’s started to change, too. Before, whenever there was an issue, he wanted to solve it immediately. Now he’s willing to accept the idea of “let’s not solve this today.” I think that’s a huge shift for us. Because before, he used to think, “If we don’t talk about it now, the problem will get bigger,” whereas I felt, “If we talk about it now, the problem will get bigger.” We were essentially pulling in opposite directions. Now we’ve found a middle ground: we can pause, but we can’t avoid the issue indefinitely.

Researcher: Are there things that passed at the time but—because you didn’t express yourself—later turned into a lingering emotional knot?

Interviewee: Yes. I think there are quite a few instances like that. For example, shortly after we got married, my husband made a joke about me in front of some friends. Everyone laughed, and I laughed too. But deep down, I felt really uncomfortable. I didn’t say anything when we got home; I felt it would be too awkward to bring it up. It wasn’t until much later—when we were discussing the topic of joking in front of friends—that I finally told him. He said he had no idea. He said, “I thought you were laughing.” I replied, “I was laughing because I didn’t know what else to do” Then, we both fell silent for a while.

Researcher: Did that instance make you realize that a smile on the surface and silence within can actually coexist?

Interviewee: Yes. I think this is a crucial aspect of cross-cultural marriages. Sometimes I might appear perfectly normal, even smiling, on the outside, but internally, I’ve already disengaged from the interaction. If he judges the situation solely by my facial expressions, he’s bound to misunderstand.

Researcher: If you had to define your silence now, how would you describe it?

Interviewee: I don’t think silence is simply the absence of speech. For me, at least, it can take many forms. Sometimes it stems from fear. Sometimes it’s exhaustion. Sometimes I simply don’t know how to express myself. Sometimes I don’t want to hurt the other person. At other times, I’m actually observing—waiting to see if the other person will realize there’s an issue on their own. And then there are times when silence serves as a way for me to protect myself for the moment.

Researcher: Do you feel you can distinguish between these different types of silence?

Interviewee: I can now. I couldn’t before. In the past, all silence felt the same to me. It was just “not speaking.”

Now, I ask myself, “Why am I not speaking?” “If the answer is ‘I’m afraid he’ll get angry,’ then I remind myself that this is likely an issue I need to face. If the answer is ‘I’m too angry right now; anything I say might be hurtful,’ then I feel that silence is valuable. If the answer is ‘I feel like speaking up won’t make a difference,’ then I become particularly wary, because that kind of silence can easily build up over time.

Researcher: Finally, I’d like to ask a more personal question. After so many years in a cross-cultural marriage, how do you feel you’ve changed?

Interviewee: I think I’ve become more willing to speak up. But that doesn’t mean I’ve turned into an incredibly blunt person; I still prefer to express myself calmly. It’s just that I no longer equate maintaining the relationship with staying silent. I used to think that, for the sake of the relationship, I should say less. Now, I realize that sometimes, for the sake of the relationship, mutual integration, and living together harmoniously, speaking up is actually necessary.

Researcher: Do you still retain any Chinese-style communication habits?

Interviewee: Of course. I still dislike heated arguments. I still consider the other person’s face and try to avoid embarrassing anyone. However, I now distinguish between being considerate of others and sacrificing myself. That distinction is very important to me.

Researcher: Regarding your silence within this cross-cultural family, is there anything else, something we haven’t asked about yet, that you feel is particularly important or would like to add?

Interviewee: I think one important point is that I used to view my silence as a personal flaw. I’d wonder, ‘Why can’t I just speak my mind directly like Americans do?’ But later, I realized it’s not that simple. Marriage involves two people building a communication style together. It’s not about Chinese people needing to become like Americans, or Americans needing to adopt Chinese ways entirely. We eventually developed a style that is uniquely ours. For instance, if I need some quiet time, I tell him. If he needs to discuss something immediately, he tells me. We even ask each other things like, ‘Is this a Chinese silence or a real silence?’

Researcher: What does that question mean?

Interviewee: It’s a bit of a joke. Sometimes, if I suddenly stop talking, he’ll ask, “Is this your ‘I’m thinking’ silence, or your ‘I’m really angry’ silence?” I would tell him which kind it was. I think that’s the shared language we eventually developed.

Researcher: So, looking at your experience, silence didn’t disappear completely. It just changed form?

Interviewee: Right. I don’t think it disappeared, nor could it ever fully vanish, because everyone has moments when they don’t want to talk. It’s just that, in the past, my silence was mostly due to not knowing what to say, fearing conflict, or worrying that speaking up would damage the relationship. Now, my silence is more intentional. For instance, I might say, “I need some time,” but I always come back to the conversation. I think that’s the biggest change.

Researcher: If you had to summarize your current understanding of silence in a single sentence, what would you say?

Interviewee: I’d say that silence doesn’t necessarily mean a refusal to communicate; sometimes it’s just another form of communication. However, if that silence remains unexplained, it can gradually turn into genuine emotional distance. I don’t think I realized that before. I used to think that staying silent was a way to avoid conflict. Now, I see it as merely setting the conflict aside temporarily. What really matters is whether you come back later to address it. I think that when studying silence in cross-cultural marriages, one shouldn’t simply view it as negative, because silence can actually protect the relationship at times. But if one person remains silent for a long time while the other is constantly left guessing, both partners end up exhausted. Ultimately, the key isn’t really “silence versus speaking,” but whether the couple can understand why the other person chose silence at a particular moment. This is especially true in cross-cultural marriages; often, it’s not a lack of love or a refusal to communicate, but rather that the way we learned to communicate growing up is fundamentally different. So, sometimes we have to relearn—learning not just how to speak, but also how to listen to what remains unsaid.

**I-23: (Male, UK partner, with children, married 4–10 years)**

Researcher: First question, looking back at the seven or eight years you’ve spent together, from dating to marriage, what do you think were the most obvious impacts of your differing cultural backgrounds, such as values, mindsets, and communication styles, on your relationship?

Interviewee: Well, it’s a bit of a double-edged sword; both the good and the bad aspects are very pronounced. Let’s start with the differences—the most immediate one is that our communication styles are worlds apart. She’s the type of person who is incredibly direct; she speaks her mind. If she’s happy, she shows it; if she’s dissatisfied, she says so. She never beats around the bush. As for me—perhaps due to my upbringing, I tend to be more indirect. I’m used to reading the room and am afraid that speaking too bluntly might hurt someone’s feelings or cause the other person to lose face if I refuse them directly. It took some getting used to when we first got together. Later, after spending more time together, I realized she wasn’t actually criticizing me; she was simply stating facts. There was no emotional baggage attached. Conversely, she found my approach exhausting. For instance, if she asked where I wanted to go on the weekend, I might not want to visit an exhibition, but instead of saying so directly, I’d say, “Whatever works for you. It’s up to you.” That would really confuse her. She’d say, “If you want to go, say so; if you don’t, say you don’t. What does ‘whatever works’ even mean?” When she makes a decision, she considers whether she likes it or if it will make her happy; when I make a decision, I first consider what works best for the household as a whole. Of course, there are positive effects, too. We complement each other. She helps me break out of rigid conventions. I used to think life had to follow a set script—get a job, save money, buy a house, raise kids, but she’d tell me, “That’s not the only way to live.” She’d drag me along to try new things I’d never even considered before. As for me, I help her handle practical, real-world issues. For example, if she struggles to communicate with clients back home, I step in to coordinate and manage the interpersonal dynamics so she doesn’t have to deal with the headache.

Researcher: From what I gather, the difference in communication styles is the most striking aspect. Was there a specific, small incident that made you realize for the first time, “Wow, our mindsets are actually worlds apart?”

Interviewee: Yes, there was. It left a really deep impression on me. In our second year of marriage, we were living in the UK. Her younger brother came to visit once, and after the meal, he started to clear the dishes to wash them. I instinctively stood up and said, “Let me do it; you’re a guest.” He looked really surprised and said, “I wash my own dishes. Why would you do it?” I was taken aback. Later, my wife explained that in her family, there’s no such thing as “guests must be waited on”; everyone is equal and handles their own affairs. If I insisted on washing up for him, he might actually feel disrespected, as if I thought he wasn’t even capable of washing a dish properly. That’s when I truly realized how cultural differences permeate even the smallest things: what you intend as politeness, the other person might perceive as an offense.

Researcher: So, does this kind of difference make you unconsciously more cautious when communicating?

Interviewee: Yes, absolutely. During the first two years of marriage, I would mentally rehearse everything I said, afraid I might say the wrong thing and step on a cultural landmine. Often, I’d just lose the desire to speak altogether; fearing a slip-up, I’d simply stay quiet.

Researcher: Right, let’s move on to the next question. Could you describe one or two specific instances of silence that left the deepest impression on you? For example, what happened, what the atmosphere was like, and how long the silence lasted? What was going through your mind when you chose to remain silent?

Interviewee: Last year, we moved from a two-bedroom apartment to a three-bedroom house. While we were sorting out the study, she dug up a bunch of my things from college—major-specific textbooks, an old MP3 player, sports trading cards I’d collected over a decade, and a handmade soccer ball model a roommate had given me as a graduation gift. She piled them all on the floor, pointing out that I hadn’t touched these items in five or six years. She suggested either donating them to a second-hand shop or just throwing them away to make room for our child’s picture books and toys, especially since we might move back to the UK for a couple of years and couldn’t haul all that clutter along. I sat there on the floor, flipping through the books, saying, “These are memories; I can’t bear to part with them.” She just laughed and said, “Memories belong in your head, not taking up physical space. Besides, you haven’t opened these books since graduation; keeping them just means they gather dust.” She got more and more animated as she spoke, even picking up that soccer model and shaking it. “The paint is chipping off this thing. Why keep it?” At that moment, I suddenly lost the desire to speak. I just squatted on the floor, holding an old textbook and flipping through the pages one by one; I didn’t respond to anything she was saying. We sat in silence like that for maybe twenty minutes. She kept muttering at first, but when she saw I wasn’t saying a word, she stopped and went to sort through other boxes herself. The whole study was incredibly quiet—just the sound of turning pages and the movers hauling things outside, and the atmosphere was pretty tense.

Researcher: When you heard her say things like “clutter” or “the paint is chipping, why keep it,” what was your immediate reaction? Why did you choose silence instead of arguing about what these items meant to you?

Interviewee: My first reaction was actually a mix of anger and a sense of being misunderstood. I felt like she had no idea what these things meant to me. That textbook was a gift from my university advisor. It even had his signature on the flyleaf. My best friend had stayed up all night making that soccer model. We live in different cities now. These weren’t useless pieces of junk; they were mementos of my youth. But why didn’t I argue? Because I felt it would be pointless. Even if I explained it all, she’d probably just think I was being overly sentimental, thinking I was picking a fight over a pile of trash. Plus, moving is exhausting and makes everyone irritable; the more we talked, the more likely an argument would break out. In the end, the stuff would get thrown out anyway, and it would just hurt our relationship. So, I decided not to say anything and just sat there to compose myself. My main thought was, “Forget it, I can’t explain this, so I won’t bother.” I also felt a bit sad, thinking she might never truly understand the life I had lived before.

Researcher: Are there any other examples?

Interviewee: Well, there was also the time earlier this year when I talked to her about quitting my job at a major tech company to work on a small project with a friend, sort of a semi-startup venture. I brought it up over dinner. I had barely started explaining when she immediately objected. She said the economic climate was bad and starting a business was too risky. She pointed out our fixed monthly expenses—mortgage, car loan, the kids’ tuition—and insisted we couldn’t afford to be without a stable income. I explained to her that I already had a plan: I wouldn’t need to quit my job right away. I’d start part-time and only consider going full-time once the project generated stable income. Plus, the startup costs were low, so I wouldn’t be touching our family savings. She still disagreed, saying I was being too idealistic—focusing only on the upside while ignoring the worst-case scenario. She asked what would happen to the family or the kids if I failed. We went back and forth for nearly an hour, but we just couldn’t get on the same page. She kept talking about risks and reality, while I kept talking about the project’s significance and my vision; we were completely out of sync. Eventually, I just felt utterly exhausted and stopped talking. That silence lasted about ten or fifteen minutes. She kept talking, saying she wasn’t against me doing what I loved, but just wanted me to be prudent. I just listened without responding or even looking at her. The only sounds at the dinner table were her voice and the noise of the kids playing with toys in the living room. It was all very stark.

Researcher: What exactly was going through your mind during those ten or fifteen minutes? Did you feel a sense of grievance at not being understood?

Interviewee: I definitely felt aggrieved. I didn’t make this decision on a whim; I’d been secretly preparing for nearly six months, doing extensive research and writing a thick business plan. Yet, she dismissed me outright without even looking at it. I felt a tightness in my chest, like I was stifled, feeling that she only cared about money and couldn’t see the value in what I wanted to do. But on the other hand, I knew she was right; she had the family’s best interests at heart. She isn’t an unreasonable person, and the risks she mentioned were very real. So, I couldn’t really argue with her; if I did, it would look like I was being irresponsible or neglecting the family. During those minutes, I was essentially wrestling with myself, feeling like I wasn’t wrong, but acknowledging she wasn’t wrong either. Not knowing what to say, I just shut my mouth to try and sort out my thoughts.

Researcher: I see. Let’s talk next about the specific situations where you’re more inclined to stay silent. For instance, when facing accusations from your partner, discussing sensitive topics, dealing with a clash of cultural values, feeling exhausted, or expressing emotions.

Interviewee: It happens in all those situations.

Researcher: Could you elaborate on that a bit?

Interviewee: It definitely happens when facing accusations from my partner. There are times when she is extremely agitated and speaks very rapidly. Whenever she gets angry, her words come out like machine-gun fire—a rapid-fire mix of Chinese and English. In those moments, I usually stay silent because I know she won’t listen to anything I say; for every word I utter, she’ll fire back ten, only adding fuel to the fire. It’s better to wait until she’s vented and calmed down before trying to talk things through. Then there are times when I’m in a bad state of mind myself, like when I’m furious, feeling deeply aggrieved, or just exhausted. I’m afraid that if I open my mouth, I’ll say something harsh and hurtful. Even though I’m frustrated, I know she’s tired too and I shouldn’t take it out on her, so I’ll say less, or nothing at all, and just give myself a moment to cool off. Another situation is when it involves her family or friends. For instance, when her parents came to stay in China, there were certain lifestyle habits I wasn’t quite used to; but since they were elders and foreigners, I felt awkward bringing it up, so I just stayed quiet and put up with it. It’s the same when her friends visit. If they do something that makes me uncomfortable, I won’t say anything to their faces because I don’t want to make her lose face.

There’s also the issue of expressing very subtle or complex emotions. Even though my English is decent and her Chinese is pretty good, when it comes to really deep feelings—the kind that might seem a bit overly sentimental or embarrassing to voice. I feel like I can’t quite find the right words. I’d feel awkward saying them in Chinese, let alone in a foreign language, so I just don’t say anything at all.

Researcher: You mentioned earlier that you choose to stay silent when her family visits; could you give a specific example? What kind of small things made you feel inconvenienced but kept you from speaking up?

Interviewee: Last year, her parents stayed in Shanghai for a month, and one thing really stuck with me. They had a habit of showering every morning, and they took a long time, over forty minutes each time. I had to leave for work at 8:30 AM, and the time around 7:00 AM was the morning rush; the bathroom was constantly occupied. Sometimes, when I was in a hurry, I’d pace around outside anxiously, but I never said a word. Eventually, I started getting up half an hour earlier, at 6:30 AM, to finish my morning routine before they woke up, just to avoid the conflict. My wife eventually found out and asked why I was getting up so early; I mentioned it casually, and she then spoke to her parents, asking them to hurry up or shower in the evening instead.

Researcher: Why didn’t you say something directly at the time? Were you afraid of offending your elders, or did you feel it wouldn’t make a difference?

Interviewee: A bit of both, I suppose. On one hand, they had traveled a long way to visit China, and as the host, I felt that criticizing their habits would make me look petty or impolite. On the other hand, I felt these were habits they’d had for decades, things that couldn’t really be changed, and bringing it up would just cause awkwardness for everyone. It was only for a month, after all; I could just put up with it. There was no need to create bad feelings over such a small matter.

Researcher: Aside from that, are there situations, like when discussing your child’s education, where you choose to remain silent?

Interviewee: Child-rearing is actually fine; we’re pretty much on the same page regarding the big picture. Neither of us wants to be the type of parent who obsessively pushes their child to excel academically. We do have occasional disagreements. For instance, she wants the child to run around outdoors, climb trees, and play in the mud, while I worry about safety, but I usually speak up directly rather than staying silent. After all, matters concerning the child are important; you can’t just bottle things up. We rarely argue about the child; we actually argue more about our own personal issues or cultural differences.

Researcher: I see. Let’s talk about the reasons behind this. What is the main reason you choose silence? Is it to avoid arguments, a feeling that things can’t be explained clearly, a desire to maintain harmony, a need to save face, the influence of cultural values, language barriers, or something else?

Interviewee: The main reason is simply that I don’t want to argue; I feel arguments damage the relationship. I’ve always believed there’s no need for a husband and wife to fight over who is right and who is wrong. This is especially true for cross-cultural couples—differences are inevitable. Arguments can easily escalate into “You Chinese people are just like that” or “You Westerners are just like that.” Once it reaches the cultural level, it becomes much harder to resolve. So, I prefer not to say anything; taking a step back often clears the air. Sometimes, I genuinely feel things can’t be explained clearly. So many things are deeply ingrained, like why I’m reluctant to throw away old items, why I give hongbao (red envelopes) to relatives, or why I dislike being criticized in public. These aren’t things you can explain in a few words. If you talk about your upbringing or cultural background at length, she might think you’re making excuses; if you don’t say enough, she won’t understand. Why go through all that trouble? Silence is easier. Subconsciously, I felt that as long as we avoided arguments and kept the home atmosphere pleasant, it didn’t matter if I had to swallow a bit of grievance. Silence, at least, maintained a semblance of peace. There was also the pressure of verbal expression. As I mentioned earlier, while everyday communication was fine, my native language felt more natural when it came to actual arguments or discussing deep emotions. Sometimes I’d have a head full of thoughts that I could articulate clearly in Chinese, but if I tried to switch to English, or even simplified Chinese, I couldn’t quite convey the nuance. I was afraid she might misunderstand, so I’d just stay silent.

Researcher: You mentioned the idea that “harmony in the family leads to prosperity,” implying that maintaining harmony is more important than determining who is right or wrong. Did you hold this view from childhood, or did it become more pronounced within your cross-cultural marriage?

Interviewee: I’ve held it since childhood; my parents were like that. However, this mindset was amplified in my cross-cultural marriage. I knew her culture was different. She didn’t share the philosophy that “harmony is paramount.” She believed in voicing problems openly, even if it led to an argument. So, I became extra cautious, afraid that saying the wrong thing might shatter that harmony, and I relied on silence to keep the peace. After all, coming from different countries, I felt I needed to be more accommodating and not act too self-indulgently.

Researcher: Have there been times when you knew silence might make your partner uncomfortable, yet you chose it anyway because you felt it was better than arguing?

Interviewee: Yes, far too many times. Often, I knew that my silence would make her overthink things or get upset, but I chose silence regardless. I felt that her unhappiness would be temporary, whereas an argument could cause deeper hurt. I’d rather she be upset for a while than get into a huge fight. I was unhappy in those moments too, of course, so naturally, my first instinct was to manage and soothe my own emotions.

Researcher: What impact does your silence have on your communication or relationship? For instance, does it maintain harmony, defuse conflict, escalate tension, leave your partner confused or hurt, or create lingering resentment? Could you give an example?

Interviewee: Well, for example, when she’s really worked up, if I stay quiet, she’ll vent for a bit, but with no one to engage or argue back, she eventually calms down on her own. It prevents the argument from spiraling out of control. If I were to argue back while we were both angry, we’d almost certainly say hurtful things we couldn’t take back, and things would get out of hand. Staying silent also buys me a bit of breathing room. When people are angry, their thoughts tend to be extreme; if you stay quiet for a while and calm down, you realize, “Oh, it’s actually not that big a deal.” Of course, if the silence drags on too long, it can leave her confused and hurt. Silence doesn’t actually solve the problem. It just suppresses it temporarily. The issue remains, and it can even grow bigger. I thought I was keeping the peace, but I was actually letting resentment build up.

Researcher: So, has there ever been a time when silence caused a minor issue to escalate into a major conflict?

Interviewee: It happened once. We were living in the UK at the time. I was in a bad mood because of work, so I didn’t say much when I got home. She thought I was angry with her because she’d accepted an invitation to a friend’s party that morning without consulting me. She didn’t ask me about it and bottled up her own frustration, so we ended up giving each other the silent treatment for two days. Eventually, I couldn’t stand it anymore and asked her why she was upset, and we realized it was all a misunderstanding. The atmosphere at home was incredibly heavy during those two days. We didn’t speak during meals and slept with our backs to each other. Looking back, it seems ridiculous; it was such a small thing, yet because we both stayed silent, we ended up at odds for two whole days.

Researcher: So, after a period of silence, how do you usually “break the ice” and resume communication?

Interviewee: When we first got married, I was usually the one to break the ice. I knew that since she was a foreigner, she didn’t understand the Chinese social convention of “taking the offered way out” [accepting a gesture to de-escalate a conflict]. If I didn’t take the initiative to speak, she could just keep the standoff going indefinitely. I’d usually start a conversation with something trivial, like pouring a glass of water and handing it to her while asking, “Do you want some water?” or offering her fruit and saying, “I just bought these strawberries; they’re really sweet,” or asking, “What do you want for dinner? I’ll make it.” By starting with these insignificant daily matters, she’d usually take the cue, answer me, and then we’d gradually open up and start talking. Then we’d sit down and review the conflicts or arguments we’d had. It wasn’t about debating who was right or wrong; I’d just explain to her why I hadn’t wanted to speak, why I was upset, and so on. That way, we could understand each other, and if we ran into similar issues later, we’d know how to handle them.

Researcher: When you explain things, do you usually use Chinese or English? Do you ever run into issues where you can’t express yourself clearly?

Interviewee: I usually use Chinese. She has no problem understanding spoken Chinese, though she has a bit of an accent when she speaks it herself. When it comes to emotions and cultural nuances, it’s still clearer to speak in one’s native language. For instance, if I explain the concept of “valuing harmony” (yi he wei gui), I can make it clear in Chinese, but the meaning gets lost if I switch to English. Still, sometimes I can’t explain things clearly. Like when I told her, “I stayed silent to save you some face,” she was really confused and asked, “Why save face? Isn’t it more respectful to just say what you mean directly?” Concepts rooted in such fundamental beliefs are really hard to explain; I have to let the ideas sink in gradually. I explain them a few times, and over time, she comes to understand.

Researcher: Let’s talk about the cultural aspect now. What concepts from Chinese culture, or aspects of your upbringing, do you think influence your choice to remain silent in an intercultural intimate relationship?

Interviewee: The influence is actually quite deep, and much of it is subtle—things I hadn’t even realized about myself until I married a foreigner and saw the contrast. It was only then that I thought, “Oh, so that’s how I am.” Growing up in my environment, I felt that arguing was bad—that it disrupted harmony, so I avoided it whenever possible. Silence was the most direct way to avoid an argument. There’s also the Chinese saying, “Silence is golden.” Growing up, we were taught to speak less and do more—that talkative people are unreliable, while a composed, reticent nature signifies maturity. I used to believe that men should speak sparingly and focus on action; chattering away all day seemed frivolous to me. Consequently, when faced with a problem, my first instinct wasn’t to voice it but to bottle it up and process it internally, viewing this as the mature approach. I felt one should always leave some leeway in conversation to avoid putting others in an awkward spot. For instance, if my partner made a mistake, I wouldn’t point it out directly for fear of hurting her self-esteem; if I disagreed with her, I wouldn’t openly object, lest she lose face. Many things I wanted to say would die on my lips, turning into silence instead. My parents argued quite a bit, but the aftermath was usually a “cold war;” neither side would speak to the other. When I was young, whenever they were at odds, the house would go deathly quiet; the atmosphere was stifling. After a day or two, once tempers cooled, they would naturally reconcile without ever sitting down to talk things through openly. Watching them back then, I came to believe that this was simply how a marriage worked: when conflicts arose, you stayed silent and waited for the moment to pass.

Researcher: Do you feel you can perceive the distinction between the collectivism of the East and the individualism of the West? Does this affect you? Interviewee: I think I can certainly sense it; we are definitely more influenced by collectivism. In our culture, personal emotions take a back seat to collective harmony. For the sake of the family’s overall harmony, it is considered right, and a sign of responsibility, to endure a little grievance or bottle up one’s emotions. So, when I’m upset, I don’t just vent; I bottle it up and process it in silence to avoid disrupting the family atmosphere.

Researcher: Do you feel, then, that this pattern of silence—learned in childhood—clashes with the reality of an intercultural marriage?

Interviewee: It clashes completely. In a Chinese marriage, everyone understands the rules: you stay silent, I stay silent; there’s a tacit understanding, and things blow over in a couple of days. But in an intercultural marriage, the other person doesn’t understand those rules at all. The moment you go silent, she panics and lets her imagination run wild, which often ends up blowing the issue out of proportion. It’s like playing a game where everyone follows different rules; if you play by your rules and she plays by hers, you’re bound to clash. So, over the past few years, I’ve been trying to change—slowly learning to voice my thoughts instead of bottling everything up.

Researcher: Do you think there’s been a change in how often or in what way you use silence in your cross-cultural marriage? How does the situation now compare to when you first got married?

Interviewee: When we first married, we were living in the UK; the environment was unfamiliar, we had few friends, and work stress was high. I’m naturally a bit introverted and guarded, so whenever an issue arose, I’d tend to clam up and avoid talking. Back then, my silence was really about not wanting to communicate. It was a form of avoidance. I felt that speaking up was pointless, so I thought it was better to say nothing. Often, there was a lot of pent-up emotion behind it. Now, it happens much less frequently. Even when I do go silent, it doesn’t last long—maybe ten or twenty minutes at most—before I take the initiative to start a conversation.

Researcher: What do you think caused such a significant change? Was it simply a result of spending time together and getting to know each other, or was it a conscious effort on your part?

Interviewee: It’s a bit of both. After living together for a while, we’ve come to understand each other—knowing each other’s boundaries and temperaments—which has created a sense of mutual understanding. I know she isn’t unreasonable; she’s just direct. She doesn’t target me intentionally, so I don’t have to worry about saying the wrong thing; I can just speak my mind. She also understands that my silence isn’t a form of the silent treatment or emotional abuse. I just need time, so she doesn’t pressure me. I’ve also made a conscious effort to change because I know silence hurts her and damages our relationship. In those early years, my silence caused so many misunderstandings and led to tears; it was heartbreaking to see. Since we’ve built a family together, I can’t just stick to my old habits; we have to accommodate each other. She’s learning to understand my silence, and I’m learning to open up and communicate more. Having a child also brought about a huge change. I didn’t want our child to see his parents stop talking whenever there was a conflict, or to feel an oppressive atmosphere at home. I want him to grow up in a happy family where we speak openly and honestly. So, for the sake of our child, I’ve pushed myself to change, trying to communicate whenever problems arise instead of resorting to the silent treatment.

Researcher: Has she changed as well? For instance, has her reaction to your silence shifted?

Interviewee: She’s changed a lot, too. When we first got married, she would get really agitated the moment I went silent, but now she stays incredibly calm. Whenever I say, “I need to cool off,” she simply replies, “Okay, let me know when you’re ready to talk.” She doesn’t pester me or let her imagination run wild; she trusts that I’ll be fine once I’ve had a moment to compose myself. She’s also learned to be mindful of saving face. For instance, she won’t criticize me directly in front of my friends; she’ll wait until we get home to bring it up. She says she learned that from me, realizing how much importance Chinese people place on it. It’s actually quite touching. We’re both making an effort to move toward each other’s cultures for the sake of this marriage.

Researcher: Right, one last question. Regarding your tendency toward silence in this cross-cultural family, is there anything we haven’t asked about that you feel is important or would like to add?

Interviewee: The thing is, in a cross-cultural marriage, there’s no right or wrong, and no culture is inherently better than the other. Directness has its merits, and so does indirectness; speaking openly has its advantages, and so does silence. It’s not about changing the other person or turning them into someone just like you; it’s about mutual understanding and accommodation, finding a balance somewhere in the middle. Take us, for example: I’m learning to speak my mind directly, while she’s learning to give me space. I’m learning not to bottle everything up, and she’s learning not to press for answers on every little thing. As we gradually adjust to each other, things have become much more comfortable. Another point is that silence has actually decreased significantly since we had children. The kids act like a bridge; often, when we’re in the middle of the silent treatment, a child will chime in, shifting the conversation and helping the anger dissipate. Plus, to set a good example for the kids, we both subconsciously keep ourselves in check, trying to communicate constructively and avoid the silent treatment. In a way, the children have helped us grow, too.

**I-24: (Female, UK partner, with children, married 4–10 years)**

Researcher: Looking back on your time together, what do you think were the most significant impacts of your differing cultural backgrounds, such as values, mindsets, and communication habits, on your intimate relationship?

Interviewee: I think the impact was actually quite profound, and it wasn’t just a simple matter of having an argument because of cultural differences. More often, it permeated the smallest details of daily life. Things like how we express displeasure or care, or deciding what should be voiced versus what should be processed internally. I think the differences there were quite noticeable. When I first got together with him, what I struggled with most was his directness. British people, or at least the version of him I know, tend to be accustomed to speaking their minds openly. If he didn’t like a plan, he might immediately say, “I don’t think this is a good idea” or “I’m not comfortable with that.” He wouldn’t perceive those words as particularly heavy or harsh. But for me, coming from the background I did, we often wouldn’t be that direct. For instance, if an elder suggested something and I felt it wasn’t right, I might say, “Hmm, let’s see when the time comes,” or “Could we try a different approach?” I might actually mean “I don’t really want to do this,” but I wouldn’t say “no” outright.

Researcher: So, would you say the most obvious difference between you lies in how you interpret “direct expression”?

Interviewee: Yes, and there’s a deeper issue, too: we have different understandings of the relationship versus differing opinions. He feels that a difference of opinion is just that, a difference of opinion, and has no direct bearing on the relationship itself. For example, if we were discussing a family decision, he might firmly oppose my view, yet ten minutes later, he’d be joking with me or cooking together. He’d think, “I just disagreed with your point; I wasn’t attacking you.” But at first, I really felt hurt. I’d wonder, “You refuted me so strongly. Do you not understand me at all? Do you think my idea is stupid?” It took time for me to realize that wasn’t what he meant at all.

Researcher: So, are you able to distinguish between “disagreeing with an idea” and “rejecting the person” now?

Interviewee: It’s much better now, though not 100%. Especially when I’m tired, I still sometimes interpret his directness as an emotional attack. And then there’s the language barrier—English. This is actually quite important. I have no trouble working in English, but the situation is completely different when my spouse and I argue. During an argument, it’s not just about whether I can get the words out; it’s about whether I can precisely convey my emotions, sarcasm, sense of grievance, or boundaries. For instance, in Chinese, I might say, “Fine, whatever makes you happy.” That single phrase can carry multiple layers of meaning—anger, disappointment, or simply “I don’t want to argue with you anymore.” But if I translate it directly into English, he might take it literally to mean, “Okay, if you’re happy, that’s fine.” So, there are times when I have a lot to say, but in the heat of the moment, I don’t know how to express it accurately.

Researcher: Does this kind of language barrier further contribute to your silence?

Interviewee: Yes, very much so. It’s not that I lack thoughts; it’s just that I feel if I speak up, I might have to spend ten minutes explaining what I mean. Then he’d ask more questions, and I’d have to keep explaining. It feels exhausting. So, sometimes the easiest solution is just to stay silent.

Researcher: Do you experience moments of silence during your daily interactions or when conflicts arise with your partner? If so, could you describe one or two scenarios that stand out most in your memory? For example, what happened, what the atmosphere was like, how long you remained silent, and why you chose silence?

Interviewee: Yes, and looking back, I realize there are actually different types of silence. They aren’t all the same. One instance that really sticks in my mind involved our family privacy. My husband likes sharing our life on social media. He thinks it’s perfectly normal—posting travel photos, family gatherings, or snapshots of our child’s daily routine—because he believes friends naturally want to know what’s going on in our lives. I, however, am not keen on making our family life public. There was a time he was about to post a photo; there wasn’t anything particularly sensitive in it, but I felt uncomfortable when I saw it. I said to him, “Can we not post this?” He asked, “Why?” I actually had plenty of reasons in my head. I felt that family life didn’t need to be shared with others, and I disliked the idea of people observing our lives via social media. But he kept asking, “What’s the problem with the picture?” and suddenly, I just didn’t know how to explain it. Researcher: So, he was asking, “What’s wrong with this photo?” while you were concerned with the deeper issue of “family privacy boundaries”?

Interviewee: Exactly. But at the time, I didn’t know how to articulate that. I just said, “Nothing. It’s fine,” and then I stopped talking. After a while, he noticed something was off and asked, “Are you upset?” I said, “No.” But of course, I was actually a bit angry.

Researcher: Why did you say “No” when you were actually angry?

Interviewee: Because I didn’t want to make a scene. Another reason was that I felt I didn’t have a particularly convincing argument. I knew I didn’t like it, but “not liking it” didn’t seem like a strong enough reason to stop him. So, I chose silence.

Researcher: How long did that silence last?

Interviewee: About an hour, I think. We had a meal together afterward, and I hardly said a word.

Researcher: What was going through your mind during that hour?

Interviewee: I felt he should have realized I was uncomfortable. But at the same time, I was blaming myself, wondering if I was being too sensitive. It was a real conflict. I didn’t tell him, “You’ve crossed my boundaries,” yet I hoped he would realize where those boundaries were on his own.

Looking back now, that was actually quite unfair.

Researcher: So, was there an element of “hoping the other person would understand you through your silence” in that moment?

Interviewee: Yes. I hadn’t realized that before. I used to think silence just meant “I don’t want to argue,” but thinking about it now, silence sometimes carries an expectation, like, “You ought to understand me.”

Researcher: Is there another typical scenario involving silence?

Interviewee: There was a time regarding my own work. I had a career opportunity that required a quick decision. It meant I might have to spend some time in another city. My husband’s first reaction was that this arrangement would put a lot of pressure on the family. He brought up practical concerns, like who would look after the kids, how we’d manage family life, and the financial costs. What he said actually made sense. But I felt terrible at the time. Because what I heard wasn’t “this arrangement is difficult,” but rather “your career isn’t as important as the family.” He never actually said that; it was just my own interpretation. He asked me, “So what do you think?” and I said, “I don’t know.” He said, “We need to make a decision.” I still said, “I don’t know,” and then I fell silent. Actually, I felt deeply aggrieved at that moment. I even felt like crying, but I didn’t. I didn’t know how to explain it. Because if I said, “You don’t support my career,” he might immediately say, “I never said that,” and we’d get stuck in a loop. I’d be talking about my feelings, while he’d be quoting his exact words. I felt that the more I tried to explain, the less clear things became. So, in the end, I just didn’t say anything. That sense of “not being able to explain it clearly” is a major reason why I choose silence, especially in a cross-cultural marriage, where I find some things incredibly hard to articulate. For instance, if I say, “I felt like what you just said made me feel undervalued,” he might reply, “But that’s not what I meant.” I know that wasn’t his intention. But the problem is, just because he didn’t mean it that way doesn’t mean my feelings simply vanish. So sometimes, I just think, “Forget it.”

Researcher: In what specific situations are you more likely to remain silent? For example, when facing accusations from your partner, discussing sensitive topics or cultural clashes, or when you are simply exhausted?

Interviewee: I think it happens in all those situations, but it’s most pronounced when the other person is angry or when I’m extremely tired.

Also, when I know an issue is complex but lack the capacity to explain it clearly in the moment. Then there are times when cultural differences come into play. And there’s another specific scenario: when we’re in front of other people.

Researcher: What exactly do you mean by “in front of other people”?

Interviewee: Like when we’re having a meal with his friends. If he says something I disagree with in front of them, I usually won’t contradict him right away. I feel like doing so would make him lose face, especially regarding private matters. Once, we were dining with some of his friends, and the conversation turned to the division of household chores. He said, half-jokingly, “She is much more organized than me, so she basically runs the household.” Everyone laughed, and I laughed too. But deep down, I didn’t feel great about it. I felt that statement oversimplified things. I actually wanted to say, “It’s not that I like managing all this; it’s just that if I don’t, nothing gets done.” But I didn’t say it. I felt there was no need. Besides, if I had spoken up right then and there, people might have thought, “Oh, they’re arguing.” I didn’t want others to think there were issues in our marriage. So, I just smiled at the time. I didn’t bring it up immediately after getting home, either; I waited until the next day. I told him, “Yesterday, when you said I run the household, I didn’t really like that.” He was surprised at first and said, “Really? I thought it was a compliment.” I replied, “I know, but it didn’t feel like one.” We ended up talking about it for quite a while. Looking back now, I feel that this kind of silence is sometimes just a delayed expression of feelings, rather than a refusal to express them at all.

Researcher: If you had to summarize, what would you say are the main reasons you choose silence?

Interviewee: Not wanting to argue. Feeling that the effort required to explain is too high. I need a little time to sort out my thoughts. I don’t want to say hurtful things when my emotions are running high. Also, it’s about preserving the relationship. Sometimes, I feel I lack the language skills to articulate my emotions clearly. And then, there’s simply a personal habit of mine. For certain things, I could explain myself in just two sentences in Chinese. But in English, I might have to explain the context, the tone, and why I felt hurt. Plus, my husband is the type who prefers logical analysis. He often asks, “What exactly do you mean?” I know he wants to understand me, but sometimes I really don’t want to analyze things; I just feel sad. I feel that while his follow-up questions are well-intentioned—aimed at communication. They actually add to my stress. For instance, if I say, “I’m upset,” he might ask, “Why?” I’d say, “Because of what you said.” He’d ask, “Which part?” Then I’d have to recall the specific words, and I’d just feel exhausted. Sometimes I really wish he would just hug me instead of making me explain why I need a hug.

Researcher: You mentioned “preserving the relationship” earlier; do you think silence really helps maintain harmony in the relationship?

Interviewee: In the short term, yes, but not necessarily in the long run. That’s been a major shift in my perspective over the last few years. When we first got married, I thought that avoiding arguments was a good thing. Later, I realized that the absence of arguments doesn’t mean problems don’t exist. If you leave certain issues unaddressed, they accumulate. For example, we once discussed household finances. He believed that a couple should remain financially independent, each keeping their own separate accounts. I, on the other hand, preferred having a joint account where we’d both contribute a portion of our money for household expenses. When we discussed it, he felt his approach was perfectly reasonable. I didn’t strongly object at the time; I just said, “Okay, that’s fine.” But deep down, I didn’t fully agree.

Researcher: Did problems arise later?

Interviewee: Yes. It wasn’t about the money itself, but rather a feeling that gradually crept up on me—a sense that I was shouldering the family’s responsibilities all by myself. I had thought I could accept that arrangement, but in reality, I had never truly voiced my expectations. So, my silence didn’t solve the problem; it only postponed it.

Researcher: Do you think your silence sometimes serves as a form of emotional regulation?

Interviewee: Definitely. I’m increasingly realizing that silence isn’t necessarily negative. When I’m furious, if I speak immediately, I might say something really harsh. So now, I’ll tell him, “I need some time,” and then I stop talking. The silence now is different from before. In the past, it was “I’m not speaking because I don’t know how to.” Now, sometimes it’s “I’m holding off on speaking because I know I need to calm down.”

Researcher: Does your husband ever misunderstand your silence?

Interviewee: He does, or at least he used to, quite often. He would think I was punishing him or sulking. Once, after an argument, I was sitting in the living room and he came over and asked, “Are you giving me the silent treatment?” I was actually really surprised. In my mind, I just needed some quiet. But he interpreted it as the “silent treatment.”

To me, those two things are completely different. When he asked, “Are you giving me the silent treatment?” I immediately felt accused. That made me want to speak even less. It turned into a cycle: he thought I was deliberately staying silent, and I felt misunderstood, so I withdrew even further into silence. Later, I explained to him that I needed him to understand: “My silence doesn’t necessarily mean I’m rejecting you.” Sometimes, it just means “I don’t have the capacity to communicate properly right now.” We eventually settled on a simple system. If I say, “I need quiet time,” he generally understands that I need to cool off. If I say, “I don’t want to talk about this,” it usually means I genuinely don’t want to discuss it. So, we gradually developed our own “language of silence.”

Researcher: How does he usually respond to your silence?

Interviewee: In the past, he would keep asking questions. “What happened?” “What’s wrong?” “Why aren’t you talking?” Sometimes he would even follow me around. That used to really annoy me, because the more I was pressed, the less I wanted to talk. Later, he learned to give me some space. But sometimes, he goes silent too.

Researcher: Is his silence the same as yours?

Interviewee: No, it’s different. His silence is usually more like, “I don’t know how to handle this.” My silence is sometimes a form of emotional self-protection. Sometimes he goes silent because he feels there’s no point in continuing the discussion. So, when we’re both silent, I actually get really nervous. I start wondering if he’s given up on communicating altogether.

Researcher: So, “mutual silence” means something different to you than “unilateral silence”?

Interviewee: Exactly. If it’s just me being silent, I’m actually hoping he’ll understand me. But if he goes silent too, I sometimes feel like the relationship has suddenly become distant.

Researcher: After a period of silence, how do you usually break the ice and resume communicating?

Interviewee: Usually, one of us starts by saying something very ordinary, like “Do you want some tea?” or “Are we still going out tomorrow?” It sounds strange, but that’s often how it happens. We don’t just jump straight into saying, “Let’s resolve our conflict”—that feels too formal. We usually return to a normal state of daily life first. Then, after a while, someone might say, “About earlier” I think that “about earlier” part is really important. It signals that we know the issue is still there, but we’re willing to reopen the conversation.

Researcher: Who usually speaks up first?

Interviewee: It used to be him most of the time. Now, sometimes it’s me. Especially since I’ve become more aware that if the silence drags on, the problem just gets bigger. So, if I feel I’ve calmed down, I’ll take the initiative to speak.

Researcher: Do you explain to him afterwards why you went silent?

Interviewee: I do now. I didn’t used to. I used to think, “He should just know.” But now I realize he genuinely doesn’t. For instance, there was a time I suddenly stopped talking, and he thought I didn’t want to continue the discussion. Later, I told him that I was actually afraid I might say something too harsh. After hearing that, he said, “I wish you had told me that.” I found that remark quite interesting at the time, because I had always thought silence minimized conflict, whereas he felt it increased uncertainty.

Researcher: So, did explaining it afterwards help?

Interviewee: It did, especially after we started discussing the silence itself. For instance, now I’ll tell him, “When I go quiet, it doesn’t mean I don’t care.” Sometimes I’ll even say outright, “I’m quiet because I’m overwhelmed.” That really helps him.

Researcher: From a cultural perspective, what concepts within Chinese culture, or aspects of your upbringing, do you think influenced your choice to use silence in an intercultural relationship?

Interviewee: I think the most obvious one is the idea of “not making a big deal out of things.” That really left a deep impression on me. Growing up, adults would often say, “Just endure it and it’ll pass.” There’s also the saying, “Harmony in the family leads to prosperity in all things.” I don’t think these ideas are necessarily bad; they make me value relationships. The problem is, sometimes I conflate maintaining the relationship with suppressing my own feelings. Another factor is that I’m not used to expressing negative emotions directly. When I’m angry, for example, I don’t naturally say, “I’m angry with you.” I’m more likely to say, “It’s okay,” even when it’s really not.

Researcher: What kind of issues do you think this way of expressing yourself causes with your British husband?

Interviewee: He takes me literally. That’s a real problem. If I say “It’s okay,” he might genuinely believe everything is fine. Then I’d find myself thinking, “How can you not tell?” Later, I realized it wasn’t his fault, because I hadn’t actually said it. I was expecting him to understand my meaning—the way Chinese people do—through my tone, facial expressions, or pauses. But he doesn’t necessarily interpret things that way.

Researcher: Do you think there have been changes in the frequency or nature of your silence compared to when you first got married?

Interviewee: There’s been a huge change. At first, my silence was mostly passive. It was a case of not knowing what to say, feeling like speaking up wouldn’t help, or worrying he’d think I was being too sensitive. Now, my silence is more intentional. I assess the situation: Is this the right time to speak? Should I say anything? How should I say it? When? So, I feel like my silence has become more of a strategy. For instance, if a conflict arises now, I don’t blurt everything out immediately. I might stay silent for ten minutes first—not to punish him, but to observe my own emotions. Then, I might only bring up the single most important point. Instead of saying, “You always do this; you never consider my feelings,” I’d say, “When you said that in front of your friends, I felt uncomfortable.” That makes communication easier. So, I feel my silence has shifted from a form of avoidance to a way of pausing before I speak.

Researcher: Aside from maintaining the relationship, do you think silence also serves to protect you?

Interviewee: Yes, especially when I feel I’m in a vulnerable position. That vulnerability isn’t necessarily about power dynamics; sometimes it’s simply because I feel I lack sufficient information. For example, when we discuss social issues in the UK or China, he might be familiar with the local context while I don’t have as much background knowledge. If he suddenly asks, “What’s your view on this?” I might not answer right away. I’d say, “I don’t know enough about it.” I used to worry that admitting this made me look ignorant, but I’ve come to realize that silence, or holding back an immediate answer, is a form of self-protection. I don’t need to speak instantly just to prove I have an opinion.

Researcher: Do you think “saving face” is still important in your cross-cultural marriage?

Interviewee: It is, but my understanding of “face” has changed. I used to be very concerned about not embarrassing my husband in front of others. If he stated a fact incorrectly, for instance, I wouldn’t correct him immediately. Now, however, if the error could actually cause problems, I will speak up. I just change how I do it. Instead of saying, “No, you’re wrong,” I might say, “I think it might be slightly different.” I’m still disagreeing, but it doesn’t sound as confrontational.

Researcher: So, you haven’t completely abandoned the Chinese style of indirect communication; you’re adapting it?

Interviewee: Exactly. I feel I’ve adopted a hybrid approach. I don’t stay silent like I used to, nor do I adopt his style of speaking bluntly. I adjust based on the situation.

Researcher: Is there a difference in how you use silence when you’re with your husband in public versus in private?

Interviewee: A huge difference. I’m more likely to remain silent in public because I don’t want others to witness our conflicts, especially when the issue is personal. For instance, if a friend asks, “Are you planning to move?” and my spouse and I haven’t reached a consensus yet, I usually won’t state my true thoughts directly. I might say, “We’re still discussing it.” That is actually a form of silence—not total silence, but withholding full information.

Researcher: What do you think are the positive and negative effects of silence on marriages between people from different cultures?

Interviewee: There are both. I’ve come to feel that a marriage without any silence isn’t necessarily healthy. People really need a bit of space sometimes. If every single emotion had to be voiced immediately, I think it would be exhausting. Sometimes, silence allows you to gather your thoughts and rephrase things, especially in a cross-cultural marriage. I need to translate my feelings into a way he can understand, and that process takes time. The biggest negative impact, however, is the lingering emotional baggage. There are things left unsaid at the time; even after the event passes, the feeling remains. Take the issue of career opportunities we faced earlier: we eventually reached an arrangement that I accepted, but long afterward, I would still recall the feeling of not being understood. So, I feel that silence can end an argument, but it doesn’t necessarily resolve the underlying emotions. Ending an argument and resolving the emotional issue aren’t the same thing. I think my biggest mistake in the past was equating “stopping the argument” with “solving the problem.” I don’t think that way anymore.

Researcher: Do you think the balance of power—who has more say, in the marriage affects your tendency to stay silent?

Interviewee: It does. But I think our situation is complex. It’s not always about who is more dominant, but rather who is better at articulating their point of view. My husband has lived in an English-speaking environment for a long time, so expressing complex ideas comes naturally to him. Sometimes, he isn’t intentionally trying to overpower me, but because he’s more skilled at verbal debate, I gradually find myself on the defensive. When I can’t win an argument, I tend to go silent. That inability to “win” the argument can be frustrating. I know I’m right, but I can’t express it quickly enough. He keeps talking, and eventually, I just stop speaking. So, language proficiency can actually become a form of power within a relationship. I used to be terrible at saying what I needed; I would only point out what others were doing wrong. Now, I try to say things like, “I need some reassurance,” or “I need you to listen rather than solve the problem.” That has really helped us. Because the British communication style often leans towards problem-solving, whereas sometimes I just want to be understood. If I don’t speak up, he immediately starts trying to solve the problem.

That just makes me angrier. So now, I tell him directly.

Researcher: If you were to summarize the changes over the years, how has your understanding of silence evolved?

Interviewee: I used to think silence was just about avoiding conflict. Now, I see it as a way of managing conflict—provided, of course, that communication follows. If the silence just drags on, that’s avoidance. But if I say, “I don’t want to talk right now; let’s discuss it tonight,” and then we actually do talk later, I think that’s healthy.

Researcher: So, what do you think distinguishes “healthy silence” from “unhealthy silence”?

Interviewee: I think there are three factors. First, is there an intention to communicate? Second, is there a clear end point? Third, is there a reconnection afterwards? If I stay silent just to make him feel bad, that’s unhealthy. But if I do it to protect myself or sort out my emotions before coming back to the conversation, then I think it’s okay. I don’t think I’m the typical “Chinese-style silent type” anymore. I’m not the same person I was when I first arrived in the UK. Yet, there are still traces of Chinese culture in my silence. For instance, I still care deeply about maintaining stable relationships. I still dislike direct confrontation in front of others, and I still consider the other person’s feelings. But I’ve also learned to express myself more directly. So, I feel like I’m a blend of two cultural logics; sometimes I evaluate myself using both sets of standards. My silence used to stem from not knowing how to say something. Now, it’s more about knowing what I want to say but choosing the right time and way to say it. I used to be more passive, but now I’m increasingly proactive, though not entirely so, because sometimes culture, language, and emotions push me toward silence. It works both ways. Sometimes I choose silence. Other times, I find myself already silent and then realize, “Oh, I’m doing it again.” After a period of silence, I reflect on my behavior. I ask myself: “Do I really not want to speak? Or am I afraid to? Am I doing this to calm down, or to avoid the consequences of conflict?” These are actually very different things. I couldn’t distinguish between them before, but now I can. If it’s just fear holding me back, then I feel I really ought to speak up after all. If I really need to calm down, staying silent for a while is an option. However, there is a risk that silence can become a habit, as it’s easy to feel that “staying quiet is safer.” After doing it once or twice, you realize that nothing terrible happens immediately if you don’t speak up. But in the long run, it might make you increasingly reluctant to express yourself.

So now, I remind myself not to treat silence as the default choice, especially regarding recurring issues. If the same problem arises a third time, I feel I have to speak up.

Researcher: You have children now. How has your communication with your husband changed since the children joined the family?

Interviewee: It has changed. But it’s not a simple case of “we don’t argue because of the kids.” In fact, having children has made us more aware of how our communication style affects the family atmosphere. When we disagree, I might have previously chosen to simply say nothing. Now, I’m more inclined to tell him, “Let’s discuss this later,” because I don’t want our children to think that “going silent when angry” is the only way to handle conflict. Of course, we can’t always get it right. We do make a conscious effort to avoid acting out in front of the kids. But I don’t think avoiding it entirely is necessarily good, either. Children need to see that adults can have differing opinions and can also repair relationships. So, we try to avoid having heated arguments in front of them, but we don’t go out of our way to hide simple disagreements.

Researcher: After all these years, do you feel you’ve developed a communication style that is neither typically Chinese nor typically British?

Interviewee: Yes, I think we’ve created something of our own. For instance, he used to prefer solving problems immediately, whereas I liked to take a breather first. Now, we combine the two approaches. He knows I need time, and I know I can’t put things off indefinitely. In the past, when I went silent, he interpreted it as rejection. Now, if I go silent, he might ask, “Do you need space?” and I’ll say “yes,” so he stops pressing me. I’ll come back to the conversation on my own later. I feel this is our own unique culture—neither Chinese nor British, but something that has evolved between the two of us over years of living together.

Researcher: If you had never resorted to silence. If you had always spoken your mind directly about everything—do you think your relationship would be better?

Interviewee: Not necessarily. I might have thought so in the past, but I don’t anymore. Direct expression can be hurtful, too, especially when emotions are running high. Sometimes, silence actually gives me the chance to avoid saying things I would later regret. So, I don’t think the issue is simply whether silence is good or bad. The key is why you are silent. Often, I used to stay silent because I wanted the other person to understand me without me having to say it. For instance, hoping he would realize I was hurt when I didn’t speak. But I later discovered that a truly mature relationship isn’t about “you should understand me,” but rather “I am willing to tell you.” That has been my biggest shift. I no longer expect him to guess what I’m thinking; I try to articulate it. Yet, I still hold onto a bit of silence, because I believe it’s impossible for people to ever fully explain themselves to one another. Some emotions simply need to be processed internally. I used to think of myself as a gentle person, but I realized that my so-called ‘gentleness” was partly just conflict avoidance. I used to equate maturity with never getting angry or arguing, but I’ve learned that isn’t necessarily true. Sometimes, having the courage to say no is a sign of maturity, too. So, I feel that this cross-cultural marriage has revealed things about myself that I might never have discovered had I lived with someone who communicated exactly the way I do.

Researcher: Do you think your silence will evolve further as the marriage progresses?

Interviewee: I think so. I hope to express myself more clearly in the future, but I don’t want to become someone who feels compelled to voice everything immediately. I still value silence, especially as a way to give myself space. I hope that, moving forward, my silence will be a choice rather than a reaction born of fear. In the past, my silence often stemmed from fear—fear of arguments, of being misunderstood, of my English not being good enough, or of complicating matters by speaking up. Now, I hope that if I am silent, it is because I recognize a need for quiet, not because I am afraid to express myself.

Researcher: Regarding your silence within this cross-cultural family, is there anything else you’d like to add, something we haven’t asked about but you feel is important?

Interviewee: I think it’s important not to view silence as inherently wrong; sometimes, you genuinely need time. However, you shouldn’t expect the other person to always understand you through silence, as people from different cultures may interpret silence in completely different ways. If you need space, tell your partner—don’t just withdraw or disappear. If something is truly important, you must speak up, even if you don’t phrase it perfectly. Relationships aren’t built on guesswork. I believe Chinese-style reticence and British-style directness can coexist; it doesn’t have to be an “either-or” choice regarding culture. I can retain my reticence while learning to be more direct, and my husband can learn to understand my pauses, hesitations, and emotions. What ultimately emerges is something else entirely. Nor should the silence of Chinese women be simply dismissed as weakness.

Sometimes I stay silent because I am observing, protecting the relationship, or safeguarding myself. Or perhaps I am searching for a more appropriate way to express myself. There is a great deal contained within that silence. For instance, when discussing an issue, I might realize that arguing right then could cause me to be swept away by emotion, so I pause. It isn’t that I am abandoning my point of view; quite the opposite. I value my perspective so much that I don’t want to speak rashly when emotions are running high. I might rethink the matter overnight and speak to him the next day. In this context, silence acts as a form of active control, a tool for self-regulation. In cross-cultural marriages, we often attribute conflicts to cultural differences. He might say, “This is the British way of communicating,” and I might say, “This is the Chinese way.” Yet, after years of marriage, I feel that not everything can be blamed on culture. Sometimes it comes down to personality, upbringing, the emotions of the moment, or simply who is more exhausted. At other times, it is simply a genuine difference of opinion on a specific issue. Culture provides the backdrop, but it isn’t the whole story. The same applies to silence. I cannot simply say, “I am silent because I am Chinese.” Often, it is because within that specific relationship and at that specific moment, I simply do not know how to express myself. Silence is not merely “not speaking.” It can mean holding back temporarily, speaking sparingly or vaguely, waiting for a different time, or simply listening. For me, it can be an act of avoidance or protection; a sign of resignation or a deliberate choice. Most importantly, it evolves alongside the relationship. When I was newly married, my silence often stemmed from not knowing how to communicate with someone from such a vastly different cultural background. Now, my silence is more often a choice I make myself. I feel that while I used to be driven by silence, I now sometimes choose when to be silent.

**I-25: (Male, US partner, with children, married > 10 years)**

Researcher: To start, I’d like you to look back on the past decade or so you’ve spent together. What do you think were the most noticeable impacts on your intimate relationship resulting from cross-cultural differences, such as values, mindsets, and communication styles?

Interviewee: The most obvious one has to be our way of thinking. She has a very typical linear mindset. She deals with issues one by one, treating each matter as a separate entity with very clear boundaries. I, on the other hand, have the holistic mindset typical of Chinese people; when considering a problem, I like to take the broader context—past and future, social connections, and human dynamics. Into account. Then there are the differences in communication habits. She is incredibly direct; you can see exactly how she’s feeling on her face. If she has an opinion, she voices it immediately without beating around the bush. Sometimes her tone comes across as quite blunt, but she doesn’t see it that way. She just thinks, “I’m telling the truth.” I’m different; I tend to leave some room for maneuver when I speak. Even if I disagree with someone, I won’t snap back directly. I always feel it’s important to give the other person a way out. There’s no need to be so absolute or harsh.

Researcher: Beyond mindset and communication, are there any areas regarding lifestyle or family values where the differences really stand out to you?

Interviewee: Our understanding of personal boundaries differs significantly, too. She places a huge value on personal space; she believes that even a married couple needs completely independent spheres. I can’t just walk into her study whenever I want, and if she’s in a bad mood, she might simply close the door and say, “I need two hours alone. Don’t disturb me.” At first, I really struggled to adjust to that. I felt that a married couple should share everything. If you’re upset, you talk about it and face it together. Shutting the door like that felt... distant. Conversely, she felt I had weak boundaries. For instance, sometimes when I’d call a childhood friend and mention what was going on at home, she wouldn’t understand it at all. She’d ask, “That’s our private family business. Why are you telling an outsider?” She couldn’t grasp that Chinese sentiment of “close friends are like family.” In her view, friends are friends and family matters are family matters—the two are kept strictly separate. Then there’s the social aspect: she loves hosting house parties. Birthdays, holidays, a promotion—any excuse will do to invite twenty or thirty people over for a barbecue and drinks. I’m naturally introverted; at parties, I have to keep a smile on my face and entertain guests the whole time, and then clean up until midnight. It completely drains me. For her, though, it’s a joy—a normal way to maintain relationships.

Researcher: Was this difference more pronounced when you were first married?

Interviewee: Definitely. The first two years were a constant process of adjustment; we’d disagree over the smallest things. Take squeezing toothpaste, for instance: she’d squeeze from the middle, while I’d push from the bottom up. She’d argue with me about it, calling me “OCD” or “controlling.” Or doing the dishes—she felt the job was done once the plates were clean, whereas I thought wiping down the stove and sink was just common sense. She’d say I was doing unnecessary extra work. Now, after more than a decade, we’ve gotten used to it. She remembers to squeeze from the bottom, and I don’t insist she wipe the stove every time; there aren’t really any “must-do-it-this-way” issues anymore.

Researcher: Let’s talk about silence. Could you describe one or two specific instances of silence that left the deepest impression on you? For example, what happened, what the atmosphere was like, how long the silence lasted, and what was going through your mind at the time?

Interviewee: I’m not a particularly talkative person to begin with, and I’m even less inclined to speak up when there’s a conflict. There was a time when she was offered a transfer to her company’s Shanghai headquarters. Her salary would double, and there was great room for career growth; she was incredibly excited. One Friday night, after the kids had gone to sleep, she sat me down to talk about future plans: renting a two-bedroom apartment there first, then moving the kids to an international school in Shanghai after six months—better educational resources, and it would make going abroad for college easier later on. But at the time, my own startup was only two years old. I had a team of a dozen people, guys who had stuck with me through the grind, and my core clients were mostly in the Southwest region. If the whole family moved to Shanghai, it would basically mean starting my company over from scratch: I’d lose my client base and my team, and all those sleepless nights and the heart and soul I’d poured into it would go to waste. I did share my concerns with her at first, but she’d say things like, “Why not just move the company to Shanghai? The market is bigger there,” or “Everyone works online these days. What’s so hard about remote management?” She spoke so lightly about it, showing she didn’t understand our industry at all. Our client relationships are built on repeated face-to-face meetings and shared meals—personal bonds that fade if you leave; you can’t maintain them just by hopping on a few video calls.

That night, she got increasingly worked up, going on about what a rare opportunity it was. She accused me of being complacent, shortsighted, and afraid to step out of my comfort zone. She ranted for over twenty minutes without letting me get a word in; every time I tried to explain my difficulties, she’d cut me off, saying, “You’re just making excuses.” Eventually, I just gave up. I sat in silence for about forty minutes while she kept going, losing steam as she spoke. Finally, she stared at me and demanded, “What exactly is your point? Say something!” I still didn’t say a word. She shoved her chair back, stormed into the bedroom, and slammed the door hard.

Researcher: What exactly was going through your mind during those forty minutes? Were there moments when you wanted to speak up? What made you hold back in the end?

Interviewee: My mind was a chaotic mess; I was thinking about a lot of things. At first, I was angry. I felt she was being incredibly selfish, focused only on her own career advancement while completely ignoring the effort I’d put in. My business isn’t a standard nine-to-five job I can just walk away from; I built it through sheer hard work. Did she really think she could just breezily dismiss it all with a casual “just move over there”? Then I felt a sense of grievance, realizing she had no desire to understand me. I’d explained the realities of my industry to her so many times, but it never sank in; she always assumed the problem was my lack of ability or my fear of taking risks.

Later on, I gradually calmed down and decided it wasn’t worth saying anything. She was completely fixated on that job offer and riding high on excitement; nothing I said would get through to her. She’d just think I was being a killjoy or holding her back. Explaining myself would only lead to an argument, which would hurt our relationship without actually solving the problem. It was better to stay quiet and let her cool off; maybe then she’d see things clearly. There were several times the words were right on the tip of my tongue. I wanted to ask, “Have you ever considered me?”, but I swallowed them back. For one thing, blurting it out would mean a falling-out; once harsh words are spoken, you can’t take them back. For another, I knew it wouldn’t help. It would only make her more agitated. I just kept it bottled up; after holding it in for so long, I really didn’t want to talk about it anymore.

Researcher: At the time, did you ever consider saying something like, “I don’t want to talk about this right now”? Why did you choose not to say anything at all?

Interviewee: How should I put it? When you’re in that kind of emotional state, anything you say feels superfluous. Plus, I was in a bit of a muddle myself; I didn’t know how to articulate my difficulties without dampening her enthusiasm. If I spoke too bluntly, I was afraid I’d hurt her feelings or make her think I didn’t support her; if I was too gentle, she wouldn’t take it seriously. So, I just kept my mouth shut, thinking I’d let things cool off and talk when everyone was calmer. Looking back, that wasn’t the best approach, but silence was my immediate, instinctive reaction.

Researcher: I see. Could you tell me about the second scenario that left a strong impression on you?

Interviewee: The second one involves her birthday party this year. She throws a big family party every year. Inviting colleagues, friends, and neighbors, totaling twenty or thirty people—for a home barbecue with drinks and conversation that lasts until midnight. I’m naturally somewhat socially anxious, so during these parties, I have to play host the whole time, serving drinks, coordinating logistics, and then cleaning up the mess afterward. It completely drains me physically and mentally; it’s more exhausting than working overtime for a week straight. Around that time last year, my company was having some trouble; a major project I’d been working on for six months fell through, and we lost a lot of money. I was overwhelmed and frazzled every day. When I got home, I just wanted to sit quietly; even talking felt like too much effort. A week before her birthday, she started dragging me into making guest lists and buying themed decorations. She wanted a Hawaiian theme this year and even wanted to hire a professional bartender. I tried to discuss it with her then, saying, “Could we keep it simple this year? Just a meal for the four of us? I’m really exhausted lately and don’t have the energy to host guests.” Her face fell immediately. She said, “The birthday party is an annual tradition—my friends are all expecting it. What would people think if we didn’t hold it this year? I’ve already made plans with everyone; how am I supposed to tell them now that we’re calling it off?” Then she started bringing up old grievances, claiming I’m always like this—antisocial, disliking her friends, unwilling to fit into her social circle. She accused me of caring only about work and not giving a damn about her happiness. She got more and more worked up as she spoke. Sitting there beside her, I just lost the desire to say anything. The project falling through was already frustrating enough; instead of offering comfort, she was digging up the past to blame me. I wanted to tell her how much pressure I was under, but I felt she wouldn’t understand. She’d just think I was using work as an excuse to dodge socializing. I thought about arguing back, but I was afraid the fight would escalate and the neighbors might hear. So, I just fell completely silent.

Researcher: During that hour or so, what was the strongest emotion you felt? Was it more a sense of grievance or a sense of powerlessness?

Interviewee: Probably a sense of powerlessness. It’s that feeling where you’re bursting with emotions and reasons, but you know voicing them is useless—the other person can’t really process or understand them. Besides, there’s no right or wrong here; her love for a lively atmosphere and socializing stems from her upbringing, while my preference for quiet and finding socializing exhausting is just my personality. It’s not like anyone is intentionally targeting the other. That’s the nature of cultural differences. There are no standard answers. You can’t convince the other person, nor can you fully compromise. You get stuck in the middle, feeling utterly powerless, so silence becomes the only option.

Researcher: Thinking about your day-to-day interactions, in what specific situations are you most inclined to remain silent? For instance, when facing accusations, cultural clashes, exhaustion, or heightened emotions, which of these most frequently triggers your silence?

Interviewee: There are a few main scenarios. I actually reflected on this before the interview. The first, and most common, is when the other person is extremely agitated and keeps accusing me of things. People aren’t rational when they’re angry; they won’t listen to any explanations. For every word I say, she has ten retorts ready, and the more I try to explain, the more the anger flares up. In those moments, I definitely keep my mouth shut and let her vent; I wait until her anger has subsided and she’s ready to listen before I slowly explain my side. The second scenario involves deep-seated cultural differences—situations where I feel I simply can’t explain things clearly. Take the concept of renqing (social reciprocity and relationship-building), for instance. In our line of work, giving small gifts to business partners during holidays to maintain relationships is completely normal in China. It’s just a matter of social etiquette. But she views it as unprofessional and unethical; we argue every time the topic comes up. Eventually, I stopped bringing it up. After all, if you try to explain the logic of a “relationship-based society” to someone who grew up in the U.S., they might grasp the literal meaning but fail to appreciate the nuances of social boundaries involved. Then there are concepts like “saving face” or “giving someone a graceful way out.” For example, if I insist on paying the bill when dining with friends, she finds it baffling and insists that splitting the cost is the only fair way. I try to explain that it’s a gesture of courtesy and etiquette, but she sees it as hypocrisy. There’s no point in belaboring these issues since she doesn’t get it, so I just stay silent rather than arguing. The third scenario is when I’m completely drained of energy, like when I’ve just returned from a string of business trips or spent the whole day in meetings. My brain feels foggy, and even speaking takes effort. If she tries to discuss something complex or picks a minor fight at times like that, I just don’t want to talk. It’s not directed at her; I simply don’t have the energy to deal with emotions. The fourth scenario is when the other person refuses to listen and keeps interrupting me. If I get cut off every time I open my mouth and have no space to express myself, what’s the point of talking? I just stop and wait until she’s actually willing to listen.

Researcher: So, looking at it from the other side. Are there situations where you absolutely wouldn’t stay silent and would insist on making your point clear?

Interviewee: I certainly wouldn’t stay silent if a bottom line was crossed. For instance, if the kids were lying, bullying others, or doing something dangerous. If she thought, “Oh, they’re just young, it doesn’t matter,” I would definitely argue the point to the end. I’d also speak up directly if she were doing something that could harm herself. But when it comes to differences in values or trivial daily matters, I generally prefer not to argue.

Researcher: What do you think is the core reason behind your choice to remain silent? Is it avoiding conflict, the difficulty of explaining things, maintaining harmony, saving face, cultural habits, or something else? Which factors are the most significant? Interviewee: The main reason is definitely that I don’t want to argue; I want to keep the relationship harmonious. Then there are cultural nuances that are really hard to explain. The more you try to explain, the deeper the misunderstanding becomes. Concepts like social obligations, “face,” and proper etiquette are ingrained in Chinese people; if you explain them to an American, she might grasp the literal meaning but fail to understand the underlying boundaries and context. Also, sometimes I just don’t react immediately and need time to gather my thoughts. For instance, if she suddenly announces a major decision or says something hurtful, my first reaction isn’t to retort but to feel stunned. I need a moment to pause and figure out the right way to speak, something that solves the problem without hurting her. That’s when I fall silent; it’s not that I don’t want to speak, but that I haven’t figured out how to say it yet, and I’m afraid saying the wrong thing will make matters worse. The language barrier isn’t really the issue. Her Chinese is decent enough for daily communication; she might be a beat slow on deep cultural references, but there are no obstacles to ordinary conversation, so language isn’t the main cause.

Researcher: So, what impact do you think your silence has had on your communication and relationship? There are both positive and negative aspects, right? Could you give some specific examples?

Interviewee: Silence helps me hold my tongue and avoid saying hurtful things. Plus, we always talk things over afterward; once we open up and understand each other’s perspectives, we get to know one another better, and the marital relationship becomes more harmonious.

Researcher: Have there been times when silence actually made the conflict worse?

Interviewee: Yes, during our second year of marriage. One weekend, I had to meet an important client and couldn’t go hiking with her. She was unhappy and complained a bit. I was in a rush and a bit annoyed, so I didn’t say anything. I just grabbed my bag and walked out the door. When I came back that evening, she had moved into the guest room and didn’t speak to me for three days. I thought she was just in a bit of a huff and would get over it in a couple of days; I didn’t realize she was so angry. I found out later that she thought I was using silence to protest against her—deliberately giving her the cold shoulder, so she gave me the silent treatment in return. Things got pretty tense, and in the end, I had to be the one to apologize and explain. Since then, I’ve learned not to use silence indiscriminately; especially when the other person doesn’t know why you’re silent, it can easily add fuel to the fire. Researcher: How does your partner usually react when she misunderstands your silence? For instance, does she press you for answers, go silent herself, or get impatient? What is your take on these reactions?

Interviewee: She used to ask me right away what was wrong and keep asking until I answered. If I stayed silent, she’d get increasingly agitated and loud; she might even cry, feeling like I didn’t care about her. If pestering didn’t work, she’d switch to the silent treatment, refusing to speak or acknowledge me, moving to the guest room, or going out to see friends, all to deliberately give me the cold shoulder. It became a battle of wills to see who would back down first, and usually, I was the one to yield. Later, after we’d been married a while and she understood my temperament better, she realized my silence didn’t mean I was angry. It just meant I needed time to process my emotions or wanted to avoid an argument. Her reaction changed; she stopped hounding me to speak. Instead, she’d pause, go about her own business, and give me some space. An hour or two later, she’d come over and ask, “Do you feel like talking now?”

Researcher: How did you view those reactions? How did you feel about the initial pestering versus the later approach of giving you space?

Interviewee: When she kept pressing me at first, I found it really annoying. I didn’t want to talk, yet she kept pushing; I might have only been a little upset initially, but the constant questioning made me even angrier. I felt misunderstood and a bit aggrieved. Later, when she started changing, giving me space and waiting for me to speak up on my own, I was actually quite touched. I knew it wasn’t easy for her; in her culture, people speak their minds directly. Her willingness to hold back her curiosity and anxiety to wait for me was a huge concession. Conversely, I knew I couldn’t expect her to accommodate me all the time; I had to change, too. So now, even if I want to stay silent, I try to say something like, “Let me calm down for a bit,” instead of just ignoring her without a word.

Researcher: After a period of silence or the silent treatment, how do you usually “break the ice” and resume communication? Is there a set pattern or a particular trick you use?

Interviewee: If it’s just a minor tiff—a brief spat followed by ten or twenty minutes of silence. There’s no need for a formal ice-breaker; a casual, unrelated remark is enough to move past it. If it’s a bit more serious, say, an hour or two of silence, usually one of us will make the first move to extend an olive branch. I’m usually the one to do it; after all, I’m a man. There’s no shame in being the first to back down. But if the conflict is major and leads to a “cold war” lasting several days, we have to sit down and have a proper talk. I usually wait until we’ve both cooled off, maybe the next evening or the one after, once the kids are asleep, and then I’ll initiate the conversation by saying, “Let’s talk,” and lay everything out clearly. We even established a rule later on: the silent treatment can’t last longer than 24 hours. Regardless of who’s right or wrong, we have to speak up within that timeframe; we can’t just let it drag on. That rule has been really helpful. It saves us from a lot of pointless emotional friction.

Researcher: Do you explain to your partner afterwards why you went silent in the moment? Does that kind of explanation help reduce misunderstandings?

Interviewee: Most of the time, yes, but never while the silence is actually happening. I wait until the incident has passed and we’re both calm before bringing it up. For instance, if I go silent during an argument, I certainly wouldn’t say, “I went quiet because you were getting too worked up”, saying that would just add fuel to the fire. Instead, I’ll mention it casually after we’ve made up: “You were so angry earlier that I was afraid anything I said would fall on deaf ears and just make the fight worse, so I stayed quiet,” or “That whole thing caught me off guard; my mind was a mess and I didn’t know how to respond, so I didn’t say anything.” It definitely helps a lot. When we were first married, she had no idea why I went silent and had to guess, and nine times out of ten, she guessed wrong, which led to a lot of misunderstandings. Later, by explaining my thoughts and reasons for staying quiet after the fact, she gradually came to understand me; she stopped jumping to conclusions like “he doesn’t love me anymore” or “this is emotional abuse.” For example, I told her about my childhood: whenever my parents fought, I’d get terrified and hide in my room, not daring to make a sound, thinking that if no one spoke, the fight couldn’t escalate. So now, whenever a conflict arises, my immediate reaction is to clam up. It’s not directed at her personally; it’s an instinctive response ingrained in me since childhood. She was deeply moved when she heard this; she had never considered that background before and had simply assumed I didn’t want to communicate with her. Since then, she doesn’t panic as much when I go silent; she knows I just need a little time to process things.

Researcher: So, do you think it’s more effective to say “I need to cool off” beforehand, or to explain things after the fact?

Interviewee: Saying something beforehand is definitely far more effective. I’m consciously trying to change that now. If I need a moment to pause, I’ll tell her, “My thoughts are a bit jumbled right now; let me think for half an hour, and then we can talk.” That way, she knows I’m not ignoring her. I just need time to organize my thoughts, so she doesn’t jump to the wrong conclusions. But sometimes, emotions flare up quickly or something happens unexpectedly, and I forget to say anything and just go silent. In those moments, explaining afterward becomes crucial. You can’t just let things slide without addressing them, or misunderstandings will keep piling up.

Researcher: From a cultural perspective, what concepts within Chinese culture, or aspects of your upbringing, do you think influence your tendency to go silent in a cross-cultural marriage?

Interviewee: Chinese culture has emphasized “harmony above all” and the idea that “a harmonious family leads to prosperity” for thousands of years. Harmony is the top priority in a family; arguments are seen as damaging to that harmony and generally negative. I was raised with this mindset, whether at home or out in the world, I dislike conflict. I tend to be patient and willing to make concessions whenever possible. This is especially true in marriage; unless it’s a matter of principle, there’s no need for a heated argument. A moment of silence lets the issue pass, and keeping things harmonious is better than anything else. We Chinese tend to be reserved when expressing feelings and emotions; we don’t like being too direct. We don’t go around saying “I love you” every day; we show it through our actions. That’s exactly the kind of personality I have. I’m not good at directly expressing my emotions, especially negative ones—because I feel like talking about them is overly sentimental or might hurt feelings. I’m used to processing things internally, and silence is part of that process. My father was a man of few words, very quiet, but he was incredibly resilient and capable of shouldering heavy burdens. My mother has a quick temper; whenever she lost her temper, my father would just sit nearby and smoke silently, never talking back or arguing. Once her anger subsided, he would simply go about his business and get on with life as usual.

Researcher: You mentioned your father’s influence. Could you elaborate on that? Was there a specific incident from your childhood that left a deep impression on you regarding the idea that “silence can resolve conflict?

Interviewee: It was probably around the time I was in junior high. My father lost a huge sum of money in a business venture, an astronomical amount for our family, and the atmosphere at home was terrible during that period. When my mother found out, she cried and berated him, calling him impulsive and unreliable, saying life was impossible. She said some really harsh things. My father just sat on the sofa, smoking cigarette after cigarette without saying a word, letting her vent. She scolded him all night until she was exhausted from crying and went to bed. The next morning, he got up and made breakfast as usual and spoke to her; although she didn’t look happy, she did respond to him. A few months later, after exploring every avenue, he managed to gradually cover the financial loss, and life returned to normal. At the time, I felt my father showed great responsibility; when faced with a crisis, he didn’t make excuses or argue. He just quietly shouldered the burden and solved the problem once the storm had passed. I thought then that when I started my own family, I wanted to be that kind of man, someone who didn’t bicker with his wife. Looking back now that I’m older, I realize that approach isn’t necessarily always right; communication is actually very important.

Researcher: Do you think this kind of Chinese-style silence is more likely to cause misunderstandings in cross-cultural marriages?

Interviewee: It doesn’t work with a foreigner; she lacks that cultural context. If you stay silent, she interprets it through her own cultural logic, assuming you are being indifferent, hostile, or engaging in the silent treatment. We often say “silence speaks volumes,” believing that a thousand words are contained within that silence and the other person should understand. But Americans don’t think that way; to them, if you don’t say it, it doesn’t exist. How are they supposed to know what you’re thinking if you don’t speak? They aren’t used to guessing, and they view guessing as a waste of time. So, in cross-cultural marriages, silence really easily leads to misunderstandings; that’s why I eventually learned to change my approach. You can’t always expect the other person to understand your culture; you have to take the initiative to speak up.

Researcher: Over the dozen or so years of your marriage, has the frequency or nature of your silence changed? How does the present compare to the early days?

Interviewee: It’s different now. For one thing, after more than a decade of adjusting to each other, we’ve reached a consensus on many issues. We don’t clash over trivial matters anymore, so naturally, there are far fewer instances of silence. Even when I do go silent, it doesn’t last long; usually, within an hour or two, I’ll take the initiative to communicate. I’d never give her the silent treatment for days on end. If I’m too emotional to say the right thing, I’ll calm down for ten minutes and gather my thoughts before talking. The goal is better communication, not avoiding the problem. Before, my silence was a passive withdrawal; now, it’s an active pause. It’s a completely different dynamic.

Researcher: What prompted this change?

Interviewee: Getting older and more mature, I realized the silent treatment was pointless. It hurts the relationship without solving anything; it’s just a waste of emotional energy. There was also a mutual willingness to accommodate each other: she tried to understand my silence, and I tried to step out of it. It was a two-way change. Of course, the most practical reason was simply that I’d learned the hard way about the downsides of silence. In the early years of our marriage, my silence caused so many unnecessary misunderstandings and pointless arguments. After a while, I realized this approach wasn’t working and I had to change.

Researcher: Did your partner’s reactions influence your behavior regarding silence?

Interviewee: Yes, significantly. For instance, whenever she cried because of my silence, it actually pained me; I’d wonder if I was being too harsh. Later, she told me that my silence made her feel insecure and led her to imagine the worst. That made me realize I couldn’t keep doing it. I needed to put her mind at ease. Also, she stopped pressuring me to speak immediately and started giving me space to open up on my own terms; ironically, that made me more willing to speak sooner. Before, the more she pressed me, the less I wanted to talk. It was almost like a rebellious phase. Now that she respects my pace, I actually make an effort to pull myself together quickly so I don’t keep her waiting too long.

Researcher: Looking back now, do you think this change is positive? Do you feel you’ve lost anything?

Interviewee: It’s definitely positive. The atmosphere at home is much more relaxed, there are fewer misunderstandings, and we understand each other better. Lost anything? I wouldn’t say that. Perhaps we really do lack that kind of unspoken rapport? But in a cross-cultural marriage, it’s inherently difficult to achieve that level of wordless understanding; it’s actually less stressful to just speak your mind. Besides, true rapport isn’t built on guesswork; it’s forged through repeated communication. We actually have a stronger connection now. We don’t need words; a single glance tells me if the other person is tired or upset. But that’s the result of over a decade of communication, not something that came from bottling things up in silence.

Researcher: Finally, regarding silence in cross-cultural families, is there anything we haven’t asked about that you consider important and would like to add?

Interviewee: Silence has its place, but it shouldn’t be overused. You can’t use silence as a weapon or as a way to perpetually avoid issues. Silence should serve as a buffer zone in communication, not the final stop. You have to circle back and resolve the problem after the silence; otherwise, emotions build up and will inevitably explode. I’ve seen plenty of cross-cultural couples where one partner talks incessantly while the other remains perpetually silent. Eventually, the talker gets exhausted, the silent partner feels stifled, and the relationship falls apart. Balance is key: stay composed when silence is called for, but speak up when it’s time to talk. In a cross-cultural marriage, effective communication is truly paramount. Effective communication isn’t about winning or losing an argument; it’s about understanding the other person from the perspective of their cultural background. That’s about it.
